# SEIS: Insight’s Seismic Experiment for Internal Structure of Mars

**DOI:** 10.1007/s11214-018-0574-6

**Published:** 2019-01-28

**Authors:** P. Lognonné, W. B. Banerdt, D. Giardini, W. T. Pike, U. Christensen, P. Laudet, S. de Raucourt, P. Zweifel, S. Calcutt, M. Bierwirth, K. J. Hurst, F. Ijpelaan, J. W. Umland, R. Llorca-Cejudo, S. A. Larson, R. F. Garcia, S. Kedar, B. Knapmeyer-Endrun, D. Mimoun, A. Mocquet, M. P. Panning, R. C. Weber, A. Sylvestre-Baron, G. Pont, N. Verdier, L. Kerjean, L. J. Facto, V. Gharakanian, J. E. Feldman, T. L. Hoffman, D. B. Klein, K. Klein, N. P. Onufer, J. Paredes-Garcia, M. P. Petkov, J. R. Willis, S. E. Smrekar, M. Drilleau, T. Gabsi, T. Nebut, O. Robert, S. Tillier, C. Moreau, M. Parise, G. Aveni, S. Ben Charef, Y. Bennour, T. Camus, P. A. Dandonneau, C. Desfoux, B. Lecomte, O. Pot, P. Revuz, D. Mance, J. tenPierick, N. E. Bowles, C. Charalambous, A. K. Delahunty, J. Hurley, R. Irshad, Huafeng Liu, A. G. Mukherjee, I. M. Standley, A. E. Stott, J. Temple, T. Warren, M. Eberhardt, A. Kramer, W. Kühne, E.-P. Miettinen, M. Monecke, C. Aicardi, M. André, J. Baroukh, A. Borrien, A. Bouisset, P. Boutte, K. Brethomé, C. Brysbaert, T. Carlier, M. Deleuze, J. M. Desmarres, D. Dilhan, C. Doucet, D. Faye, N. Faye-Refalo, R. Gonzalez, C. Imbert, C. Larigauderie, E. Locatelli, L. Luno, J.-R. Meyer, F. Mialhe, J. M. Mouret, M. Nonon, Y. Pahn, A. Paillet, P. Pasquier, G. Perez, R. Perez, L. Perrin, B. Pouilloux, A. Rosak, I. Savin de Larclause, J. Sicre, M. Sodki, N. Toulemont, B. Vella, C. Yana, F. Alibay, O. M. Avalos, M. A. Balzer, P. Bhandari, E. Blanco, B. D. Bone, J. C. Bousman, P. Bruneau, F. J. Calef, R. J. Calvet, S. A. D’Agostino, G. de los Santos, R. G. Deen, R. W. Denise, J. Ervin, N. W. Ferraro, H. E. Gengl, F. Grinblat, D. Hernandez, M. Hetzel, M. E. Johnson, L. Khachikyan, J. Y. Lin, S. M. Madzunkov, S. L. Marshall, I. G. Mikellides, E. A. Miller, W. Raff, J. E. Singer, C. M. Sunday, J. F. Villalvazo, M. C. Wallace, D. Banfield, J. A. Rodriguez-Manfredi, C. T. Russell, A. Trebi-Ollennu, J. N. Maki, E. Beucler, M. Böse, C. Bonjour, J. L. Berenguer, S. Ceylan, J. Clinton, V. Conejero, I. Daubar, V. Dehant, P. Delage, F. Euchner, I. Estève, L. Fayon, L. Ferraioli, C. L. Johnson, J. Gagnepain-Beyneix, M. Golombek, A. Khan, T. Kawamura, B. Kenda, P. Labrot, N. Murdoch, C. Pardo, C. Perrin, L. Pou, A. Sauron, D. Savoie, S. Stähler, E. Stutzmann, N. A. Teanby, J. Tromp, M. van Driel, M. Wieczorek, R. Widmer-Schnidrig, J. Wookey

**Affiliations:** 1 Institut de Physique du Globe de Paris-Sorbonne Paris Cité, Université Paris Diderot (UMR 7154 CNRS), Planetology et Space Science Team, 35 Rue Hélène Brion, Paris, 75013 France; 2 Jet Propulsion Laboratory, California Institute of Technology, Pasadena, CA 91109 USA; 3 Institut of Geophysics, ETHZ, Sonneggstrasse 5, 8092 Zurich, Switzerland; 4 Department of Electrical and Electronic Engineering, Faculty of Engineering, Imperial College London, London, UK; 5 Department of Planets and Comets, Max Planck Institute for Solar System Research, Göttingen, Germany; 6 Centre National d’Etudes Spatiales, 18 av. Edouard Belin, 31401 Toulouse Cedex 9, France; 7 Atmospheric, Oceanic, & Planetary Physics, University of Oxford, Parks Road, Oxford, OX1 3PU UK; 8 Clarendon Laboratory, University of Oxford, Parks Road, Oxford, OX1 3PU UK; 9 ISAE-SUPAERO, Toulouse University, 10 Avenue E. Belin, 31400 Toulouse, France; 10 LPG Nantes, UMR6112, CNRS-Université de Nantes, 2 rue de la Houssinière, BP 92208, 44322 Nantes cedex 3, France; 11 NASA Marshall Space Flight Center, 320 Sparkman Drive, Huntsville, AL 35805 USA; 12 Institut de Recherche en Astrophysique et Planétologie, UMR5277 CNRS - Université Toulouse III Paul Sabatier, 14, avenue Edouard Belin, 31400 Toulouse, France; 13 RAL Space, STFC Rutherford Appleton Laboratory, Harwell Science and Innovation Campus, Didcot, OX11 0QX UK; 14 Kinemetrics, 222 Vista Av., Pasadena, CA 91107 USA; 15 Cornell Center for Astrophysics and Planetary Science, Cornell University, Ithaca, NY USA; 16 Centro de Astrobiologia, Madrid, Spain; 17 Earth, Planetary and Space Sciences, University of California, Los Angeles, Los Angeles, USA; 18 Swiss Seismological Service, ETHZ, Sonneggstrasse 5, 8092 Zurich, Switzerland; 19 Geoazur, University Cote d’Azur, 250 rue Einstein, 06560 Valbonne, France; 20 Département de Sismologie, Institut de Physique du Globe de Paris-Sorbonne Paris Cité, UMR 7154 CNRS - Université Paris Diderot, 1 Rue Jussieu, Paris Cedex, 75238 France; 21 Royal Observatory of Belgium, 3 avenue Circulaire, 1180 Brussels, Belgium; 22 Laboratoire Navier (CERMES), Ecole des Ponts ParisTech, Marne la Vallée, France; 23 Institut de Minéralogie et de Physique des Matériaux et de Cosmochimie, Case courrier 115, 4 Place Jussieu, 75252 Paris Cedex 05, France; 24 University of British Columbia, Vancouver, BC Canada; 25 Planetary Science Institute, Tucson, AZ USA; 26 SYRTE, Observatoire de Paris, Université PSL, CNRS, Sorbonne Université, LNE, 61 avenue de l’Observatoire, 75014 Paris, France; 27 School of Earth Sciences, University of Bristol, Wills Memorial Building, Queens Road, Bristol, BS8 1RJ UK; 28 Department of Geosciences, Princeton University, Guyot Hall, Princeton, NJ 08544 USA; 29 Observatoire de la Côte d’Azur, Boulevard de l’Observatoire, CS 34229, 06304 Nice Cedex 4, France; 30 Black Forest Observatory, Karlsruhe Institute of Technology and Stuttgart University, Heubach 206, 77709 Wolfach, Germany; 31 Present Address: Institut d’Astrophysique Spatiale, Université Paris-Sud, Bâtiment 121, 91405 Orsay Cedex, France; 32 Present Address: Laboratoire de Mécanique et d’Acoustique, LMA - UMR 7031 AMU - CNRS - Centrale Marseille, 4 impasse Nikola Tesla, CS 40006, 13453 Marseille Cedex 13, France; 33 Present Address: Advanced Technology and Research, Arup, 13 Fitzroy Street, London, W1T 4BQ UK; 34 Present Address: Center for Gravitational Experiments, Huazhong University of Science and Technology, 1037 Luoyu Rd, Wuhan, 430074 P.R. China

**Keywords:** Mars seismology, InSight

## Abstract

By the end of 2018, 42 years after the landing of the two Viking seismometers on Mars, InSight will deploy onto Mars’ surface the SEIS (**S**eismic **E**xperiment for **I**nternal **S**tructure) instrument; a six-axes seismometer equipped with both a long-period three-axes Very Broad Band (VBB) instrument and a three-axes short-period (SP) instrument. These six sensors will cover a broad range of the seismic bandwidth, from 0.01 Hz to 50 Hz, with possible extension to longer periods. Data will be transmitted in the form of three continuous VBB components at 2 sample per second (sps), an estimation of the short period energy content from the SP at 1 sps and a continuous compound VBB/SP vertical axis at 10 sps. The continuous streams will be augmented by requested event data with sample rates from 20 to 100 sps. SEIS will improve upon the existing resolution of Viking’s Mars seismic monitoring by a factor of $\sim 2500$ at 1 Hz and $\sim 200\,000$ at 0.1 Hz. An additional major improvement is that, contrary to Viking, the seismometers will be deployed via a robotic arm directly onto Mars’ surface and will be protected against temperature and wind by highly efficient thermal and wind shielding. Based on existing knowledge of Mars, it is reasonable to infer a moment magnitude detection threshold of $M_{{w}} \sim 3$ at $40^{\circ}$ epicentral distance and a potential to detect several tens of quakes and about five impacts per year. In this paper, we first describe the science goals of the experiment and the rationale used to define its requirements. We then provide a detailed description of the hardware, from the sensors to the deployment system and associated performance, including transfer functions of the seismic sensors and temperature sensors. We conclude by describing the experiment ground segment, including data processing services, outreach and education networks and provide a description of the format to be used for future data distribution.

**Electronic Supplementary Material:**

The online version of this article (10.1007/s11214-018-0574-6) contains supplementary material, which is available to authorized users.

## InSight’s SEIS: Introduction and High Level Science Objectives

The InSight mission will deploy the first complete geophysical observatory on Mars following in the footsteps of the Apollo Lunar Surface Experiments Package (ALSEP) deployed on the Moon during the Apollo program (e.g. Latham et al. 1969, 1970; Bates et al. 1979) It will thus provide the first ground truth constraints on interior structure of the planet.

The InSight spacecraft was launched on May 5, 2018 and landed on Mars on November 26, 2018 in Elysium Planitia (Golombek et al. 2017). The three primary scientific investigations are the Seismic Experiment for Interior Structure (SEIS), the Heat Flow and Physical Properties Package (HP^3^, Spohn et al. 2018), a self-hammering mole that deploys a tether with temperature sensors to a depth of 3–5 m and the Rotation and Interior Structure Experiment (RISE; Folkner et al. 2018, an X-band precision tracking experiment which will follow the motion of the lander over a Martian year to determine the precession and nutation of Mars).

In addition, there is a set of environmental sensors grouped as the Auxiliary Payload Sensor Suite (APSS; Banfield et al. 2018). This set of instruments includes a pressure sensor, wind sensors and a magnetometer. It was primarily included to decorrelate seismic events from atmospheric effects or lander and planetary magnetic field variations and ensure that putative seismic signals are not mistaken for wind activity. It is notable that the magnetometer might potentially be used to perform crustal and lithosphere magnetic sounding and the pressure sensor has a sensitivity compatible with infrasound detection.

Finally, the lander also has an Instrument Deployment System (IDS; Trebi-Ollennu et al. 2018) with a robotic arm (Instrument Deployment Arm, or IDA) and set of cameras (Maki et al. 2018) which will deploy SEIS and HP^3^ to the surface of Mars. The camera will also be used to determine the azimuth of SEIS with respect to Geographic North Pole (Savoie et al. 2018) and to better understand the geology and physical properties of the local surface and shallow subsurface (Golombek et al. 2018).

The InSight mission goal is to understand the formation and evolution of terrestrial planets through investigation of the interior structure and processes of Mars and secondarily to determine the present level of tectonic activity and impact flux on Mars.

More specifically, the payload is targeted to determine through geophysical measurements the fundamental planetary parameters that can substantially contribute to these goals. Thus, in order to address these goals, InSight has the following science objectives:

- Determine the size, composition and physical state of the core.
- Determine the thickness and structure of the crust.
- Determine the composition and structure of the mantle.
- Determine the thermal state of the interior.
- Measure the rate and distribution of internal seismic activity.
- Measure the rate of impacts on the surface.

These goals have all been quantified as listed in Table 1 and have defined the InSight mission requirement (Level 1 or L1). Their rationale in terms of knowledge of Mars’ interior structure and evolution is described in detail in Smrekar et al. (2019, this issue). All of these goals were defined before InSight was selected in 2012. In the ensuing six years, some of these have benefited from advances in knowledge from ongoing orbiter and lander measurements, but most are even more worthy of pursuit in view of recent findings. We illustrate this point for two examples, the core size and the crustal thickness

**Table 1:** L1 Mission Requirements of the InSight along with their associated science objectives. When instrument name in the last column is in bold, the requirement is considered as threshold while it is baseline otherwise. All threshold goals (*dark grew*) are associated with internal structure while the baseline (*light grew*) are associated with heat flow, seismic activity and impact rate. Only the Mission requirements related to SEIS are numbered

Science objectives	Mission requirements	2012 knowledge	Experiment
Determine the thickness and structure of the crust	L1-1 Determine the crustal thickness to $\pm 10~\mbox{km}$	$\pm 35~\mbox{km}$	**SEIS**
	L1-2 Detect any regional-scale crustal layering with velocity contrast $\geq 0.5~\mbox{km}/\mbox{s}$ over a depth interval $\geq 5~\mbox{km}$	No information	**SEIS**
Determine the composition and structure of the mantle	L1-3 Determine the seismic velocities in the upper 600 km of mantle to within $\pm 0.25~\mbox{km}/\mbox{s}$	$\pm 1~\mbox{km}/\mbox{s}$ (inferred)	**SEIS**
Determine the size, composition and physical state of the core	L1-4 Positively distinguish between a liquid and solid outer core	None (likely liquid)	**RISE+SEIS**
	L1-5 Determine the core radius to within $\pm 200~\mbox{km}$	$\pm 450~\mbox{km}$	**RISE+SEIS**
	*Determine the core density to within* $\pm 450~\mbox{kg}/\mbox{m}^{3}$	$\pm 1000~\mbox{kg}/\mbox{m}^{3}$	**RISE**
Determine the thermal state of the interior	*Determine the heat flux at the landing site to within* $\pm 5~\mbox{mW}/\mbox{m}^{2}$	$\pm 25~\mbox{mW}/\mbox{m}^{2}$	HP3
Measure the rate and geographic distribution of seismic activity	L1-6 Determine the rate of seismic activity to within a factor of 2	Factor of 10 (inferred)	SEIS
	L1-7 Determine epicenter distance to ±25% and azimuth to ±20^∘^	No information	SEIS
Measure the rate of meteorite impacts on the surface	L1-8 Determine the rate of meteorite impacts to within a factor of 2	Factor of 6	SEIS

For the core and in the same way that Jeffreys (1926) demonstrated the liquid state of the Earth’s outer core using tidal measurements, the range of $k_{2}$ values observed for Mars at the Solar tidal periods may only be explained by a core in a primarily, if not entirely, liquid state (Yoder et al. 2003). The most recent determinations of the tidal Love number from orbiters have furthermore narrowed our estimation of the Mars core. The last proposed value for $k_{2}$ ($0.163 \pm 0.008$, based on the estimates of Konopliv et al. 2016 and Genova et al. 2016) ruled out earlier results in the range of $k_{2}=0.12\mbox{--}0.13$ by Marty et al. (2009). It implies a core radius in the range of 1710–1860 km for the SEIS reference models (Smrekar et al. 2019, this issue) and in an even smaller range as proposed by Khan et al. (2018) (1720–1810 km). These two ranges are smaller than the $\pm 200~\mbox{km}$ expected originally through either SEIS tidal or RISE geodetic measurements. But as shown by Panning et al. (2017) and Smrekar et al. (2019, this issue) more than 150 seconds of difference is predicted between the SEIS reference models for the arrival at the InSight station of shear waves generated by quakes and reflected by the core. InSight should thus be able to use core reflected waves to determine the core radius with much better resolution, perhaps a few tens of km. This is important because core size controls the maximum mantle pressure, which can have a significant influence on mineralogy and potential mantle convection regimes.

Our second example is the crust. It appears to be very far from the homogeneous crust assumed in most geophysical models and the Martian lithosphere might also be far from thermodynamic and mechanical equilibrium. Goossens et al. (2017) suggested for example a very low average bulk density of $2582\pm 209~\mbox{kg}/\mbox{m}^{3}$ which is significantly less than the $2660\mbox{--}2760~\mbox{kg}/\mbox{m}^{3}$ range assumed by Khan et al. (2018). This suggests a mean crustal thickness of about 42 km, very well outside the 55–80 km range of Khan et al. (2018). The mean crustal thickness proposed by Goossens et al. (2017) is moreover based on the assumption than some crust remains even beneath the largest impacts, which remains to be proven. In addition, higher densities for the volcanoes (e.g., Belleguic et al. 2005), the discovery of feldspar-rich magmatic rocks analogous to the earliest continental crust on Earth (Sautter et al. 2015) and possible large temperature variations in the lithosphere (Plesa et al. 2016) indicate the possibility of significant lateral density variations which make gravity constraints weaker. Knowledge of the crustal thickness has therefore arguably not improved when one takes into account these unknowns, largely as a consequence of the non-uniqueness of any gravity interpretation and the lack of penetration for other geophysical observations (e.g., ground penetrating radars). Seismic measurements are mandatory for any significant new step in our knowledge of the Martian mean crustal thickness.

The SEIS goals can also be considered in another context and compared to historical achievements in terrestrial and lunar seismology (see, e.g., Ben-Menahem 1995; Agnew 2002; Dziewonski and Romanowicz 2015; Schmitt 2015; Lognonné and Johnson 2015).

After having located seismic activity on the Earth from human reports (Mallet 1853), instrumental seismology grew rapidly following the first remote observation of a quake from Japan in Potsdam (von Rebeur-Paschwitz 1889) and the first observation of the solid Earth tide with a gravimeter (Schweydar 1914). Subsequently seismology on the Earth was able to rapidly decipher the interior details of our planet. Table 2 provides a comparison between SEIS goals for Mars and some key discoveries made on the Earth in the period from 1850 to 1926 and on the Moon following the Apollo seismometer deployments in the early 1970s. Of course, such early observations always triggered alternative interpretations and multiple controversies before reaching consensus.

**Table 2:** Comparison of the mission science objectives with achievements made in terrestrial and lunar seismology. The suggested references are those corresponding to the first observation reported either in historical reviews or in the literature for the Moon. Many more studies were of course done after

Science objective	Earth analogue	Lunar analogue
Determine the thickness and structure of the crust	Mohorovičić (1910, 1992)	Toksöz et al. (1972)
Determine the composition and structure of the mantle	Jeffreys and Bullen (1940)	Nakamura et al. (1973) Toksöz et al. (1974) Nakamura et al. (1976)
Determine the size, composition and physical state of the core	Oldham (1906) (body waves) Jeffreys (1926) (solid tide)	Williams et al. (2001), from LLR Nakamura et al. (1974), from far impact Weber et al. (2011), from ScS Garcia et al. (2011), from ScS
	Oldham (1906) (body waves)	
Measure the rate and geographic distribution of seismic activity	e.g. Mallet (1853)	Latham et al. (1971), DMQ Nakamura et al. (1974), HFT
Measure the rate of meteorite impacts on the surface	N/A (due to atmosphere shielding)	Duennebier and Sutton (1974) Dainty et al. (1975)

The major challenge of InSight SEIS, with its first non-ambiguous detection of marsquakes and solid tides, will be to implement a third planetary seismological success story. The single-station character of the mission will limit its scope compared to the 4-station Lunar passive seismology network (plus a partial fifth station consisting of the Apollo 17 gravimeter, Kawamura et al. 2015) and the current very dense network on Earth. This is among the reasons why InSight has chosen not to target the interpretation of any seismic observations deeper than the core-mantle boundary, likely leaving observation of any possible inner core phases, as made on Earth by Lehmann (1936) and proposed by Weber et al. (2011) for the Moon, a possible goal for future Mars geophysical networks.

## Mars Seismology Background

### Summary of Past Missions and InSight Pre-selection Efforts

Seismology on Mars started with the seismometers on the Viking landers. This first attempted seismic exploration of Mars (Anderson et al. 1977a, 1977b) was unfortunately much less successful than the seismic exploration of the Moon. On Mars only the Viking 2 seismometer was operational, as the seismometer on the Viking 1 lander failed to unlock. The sensitivity of the Viking 2 seismometer was an order of magnitude less than the sensitivity of the Lunar Short Period (SP) seismometer for periods shorter than 1 s and five orders of magnitude less than the Lunar Long Period (LP) seismometer for periods longer than 10 s. No events were convincingly detected during the seismometer’s 19 months of nearly continuous operation, with the possible exception of one event on sol 80 (Anderson et al. 1977a). The event occurred when no wind data were recorded but recent analyses (Lorenz et al. 2016) have shown that the local time excludes, with a better than 95% probability, wind-induced lander noise with such a high amplitude level. Nevertheless, the absence of other recorded events, as shown by Goins and Lazarewicz (1979), was probably related to the inadequate sensitivity of the seismometer in the frequency bandwidth of teleseismic body waves, as well as the device’s high sensitivity to wind noise (Nakamura and Anderson 1979). On the other hand, this sensitivity provided a means to monitor the wind and atmospheric activity in a quite different way than with classical weather sensors (Lorenz and Nakamura 2014).

The next mission for Mars seismology was the ambitious Russian 96 mission with a very large orbiter, two small autonomous stations (Linkin et al. 1998) equipped with the short-period OPTIMISM (from the French “**O**bservatoire **P**lané**T**olog**I**que **M**agnét**I**sme et **S**ismique sur **M**ars”) seismometers (Lognonné et al. 1998) and 2 penetrators (Surkov and Kremnev 1996) with the Kamerton short-period (SP) seismometers (Khavroshkin and Tsyplakov 1996). After a successful initial launch, it failed to insert into a trans-Mars trajectory and fell into the Pacific.

More than 2 decades of efforts will have therefore been spent in proposal formulations and instrument development between the collapse of Mars 96 and the selection of InSight. See the summary provided by Lognonné (2005), including efforts related the NetLander project, a network of 4 landers cancelled at the end of its phase B (Harri et al. 1999; Sotin et al. 2000; Lognonné et al. 2000; Banerdt and Pike 2001; Marsal et al. 2002; Dehant et al. 2004). We will describe only those directly related to the conception of InSight and SEIS.

The collapse of NetLander marked indeed the end of near terms perspectives for a Mars Seismic Network mission and implied focus on a single-station seismic pathfinder mission (Banerdt and Lognonné 2003; Lognonné and Banerdt 2003). The first attempts were made jointly in the USA and in Europe, respectively with the GEMS (Geophysical and Environment Monitoring Station) NASA Mars Scout Program proposal (based on a Mars Pathfinder-like lander; Banerdt and Smrekar 2007) and with the Humboldt package (Lognonné et al. 2006; Biele et al. 2007, Mimoun et al. 2008) onboard the ExoMars lander and later, the MarsTwin proposal to ESA (Dehant et al. 2010). Although none of these projects were selected, they all paved the way for the InSight/SEIS design (Lognonné et al. 2011), which was proposed in 2010 to the NASA Discovery Program of the GEMS proposal (Banerdt et al. 2011). The latter was finally selected and renamed InSight in 2012. Thus some 40 years after the Viking landings, InSight will explore a virgin planet for seismology, armed only with a sparse set of a-priori constraints on the internal structure derived from other types of orbital or rover investigations and measurements on Martian meteorites.

### Mars Interior Structure Before InSight

While the expected data returned from SEIS will allow for a much-improved knowledge of the interior structure of Mars, it is possible to use existing geophysical observations, combined with geochemical analysis and mineral physics experiments and modeling, to estimate possible domains of internal structure. We review briefly in this section our knowledge before Insight’s seismic data and refer to Smrekar et al. (2019, this issue) for a more in-depth discussion.

Even without seismic observational data, many estimations of the internal elastic and compositional structure of Mars have been made in the last 40 years. The first were based on the knowledge on the seismic structure of the deep Earth, transposed to the pressure conditions inside Mars (Anderson et al. 1977a; Okal and Anderson 1978; Lognonné and Mosser 1993; Mocquet and Menvielle 2000). Fundamental constraints from planetary mass, moment of inertia and gravimetric tidal Love number $k_{2}$ (for the latest assessment of $k_{2}$, see Genova et al. 2016 and Konopliv et al. 2016) combined with some assumptions on bulk chemistry based on constraints primarily from Martian meteorites have then be used. See reviews in, e.g., McSween (1994) and Taylor (2013). A chronological evolution of the proposed models can be appreciated through recently published reviews in Mocquet et al. (2011), Dehant et al. (2012), Lognonné and Johnson (2015), Panning et al. (2017) and Smrekar et al. (2019, this issue).

**Fig. 1:**
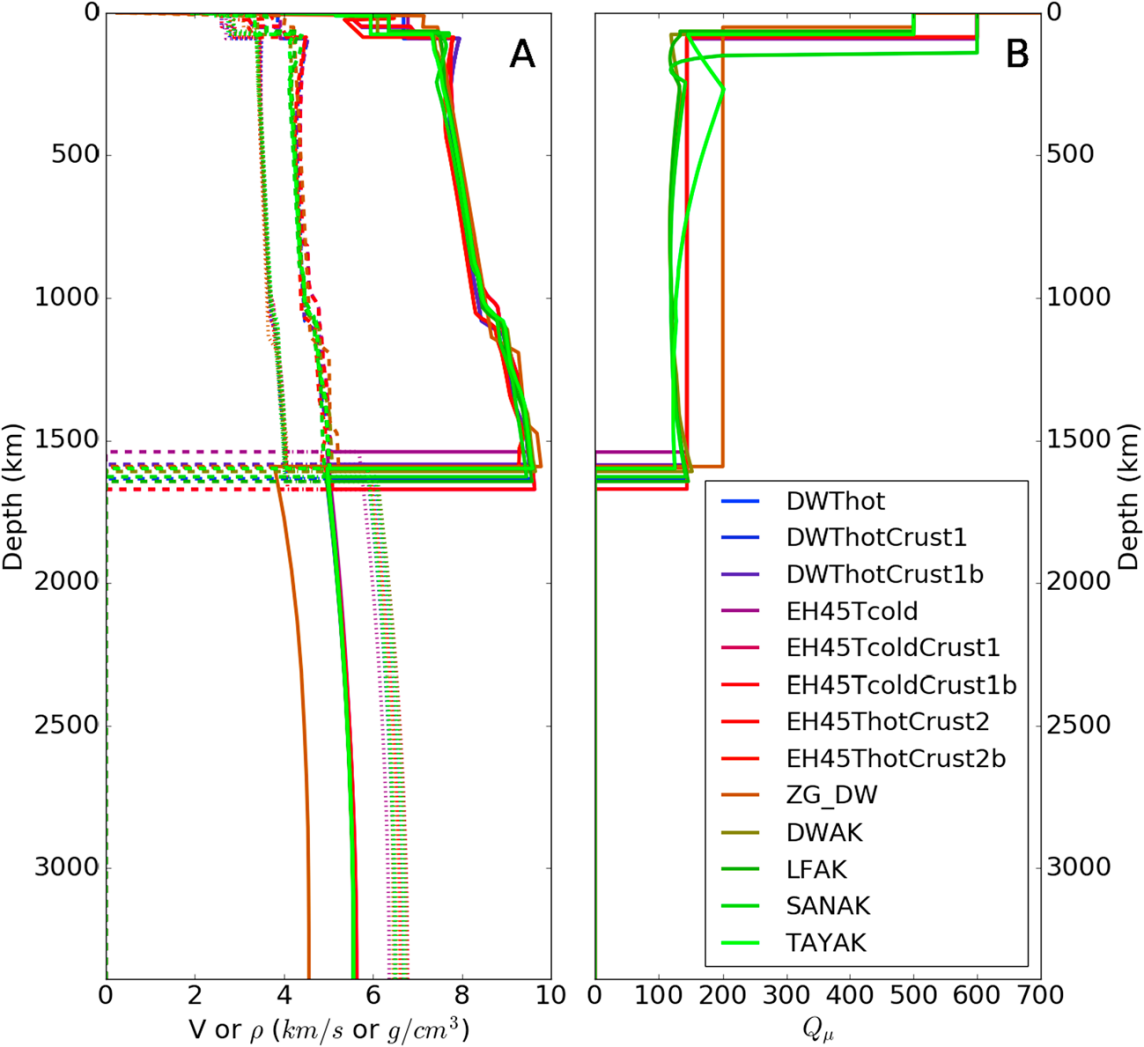
Sample suite of 13 models (color-coded as in legend in lower right). (**A**) $V_{P}$ (solid lines, in km/s), $V_{S}$ (dashed, in km/s) and density (dotted, in $\mbox{g}/\mbox{cm}^{3}$) as function of depth (km). (**B**) Shear quality factor ($Q$) as a function of depth. Models DWThot through EH45ThotCrust2b are from Rivoldini et al. (2011), ZG_DW is from Zharkov et al. (2009) and models DWAK through TAYAK are from Khan et al. (2018). Figure updated from Panning et al. (2017) with models available at: 10.5281/zenodo.1478804

The current restricted set of geophysical and geochemical constraints allow for a wide variety of theoretical models. As part of the preparation for InSight, we have defined a sample range of candidate models (Fig. 1) similar to the collection created for a community blind test of Marsquake location approaches (Clinton et al. 2017). Eight of the models in the set (those with model names beginning with DWT or EH45T) are based on Rivoldini et al. (2011) and described in Panning et al. (2017). The model labeled ZG_DW is model M14_3 of Zharkov et al. (2009), based on the Dreibus and Wänke (1985) chemical model. The ZG_DW model has been corrected for the larger $k _{2}$ value in Zharkov et al. (2017). The family of “AK” models (Khan et al. 2018) are constructed assuming 4 different bulk mantle compositions (the preface to “AK” with DW, LF, SAN and TAY referring to Dreibus and Wänke (1985), Lodders and Fegley (1997), Sanloup et al. (1999) and Taylor (2013), respectively) and therefore mineralogy. Models vary due to different assumed bulk silicate and core compositions, crustal models, thermal profiles and depths of first order interfaces (i.e. crust-mantle and core mantle boundaries). The largest differences between the models are primarily due to the trade-off between the core radius, the spherically averaged thickness of the crust and of their corresponding densities. Seismological constraints on the depth of the Moho and hopefully on the core radius, will be extremely useful to resolve these ambiguities. Waiting for these data, the mean density and moment of inertia already provide rather precise values for the gradients of pressure and adiabatic increase of temperature inside the mantle ($0.12~\mbox{K}/\mbox{km}$).

In the mantle, relatively shallow variation arises primarily due to a wide range of possible thermal profiles (Plesa et al. 2016). In the bulk of the mantle, however, velocity and density variations between possible models are smaller. For example, when a suite of models was calculated in the study of Panning et al. (2017) with varying published Martian mantle compositions, either enriched in olivine or pyroxene and temperature profiles (Plesa et al. 2016) using a consistent equation of state approach based on the code PerpleX (Connolly 2005) with thermodynamic data from Stixrude and Lithgow-Bertelloni (2011) and Rivoldini et al. (2011), shear velocity varied only within a band of $\pm 0.15~\mbox{km}/\mbox{s}$. Some mid-mantle variation between models can be seen, however, near $1100 \pm 200~\mbox{km}$ depth, where phase transitions between olivine, wadsleyite and ringwoodite are expected. The depth and sharpness of this transition, which is critical for determining whether seismic energy reflecting from such a transition can be observed, are primarily governed by the iron content and the temperature of the mantle (Mocquet et al. 1996). Estimate vary depending on the composition and temperature distribution used in the models (e.g. Sohl and Spohn 1997; Gudkova and Zharkov 2004; Verhoeven et al. 2005; Zharkov and Gudkova 2005; Khan and Connolly 2008; Zharkov et al. 2009; Rivoldini et al. 2011; Khan et al. 2018).

### Expected Seismic Activity on Mars from Quakes and Impacts

We refer the reader to Clinton et al. (2018) for a more detailed discussion on internal seismic activity, Daubar et al. (2018) for impacts and summarize below the key points in term of targeted quake and impacts. Mars is expected to be seismically more active than the Moon, but less active than the Earth, based on the relative geologic histories of the terrestrial planets (Solomon et al. 1991; Oberst 1987; Goins et al. 1981). The total seismic moment release per year is $\sim 10^{21}\mbox{--}10^{23}~\mbox{N}\,\mbox{m}/\mbox{yr}$ on the Earth (Pacheco and Sykes 1992) and $\sim 10^{15}~\mbox{N}\,\mbox{m}/\mbox{yr}$ on the Moon (Goins et al. 1981). This would suggest a total moment release on Mars to be midway between the Earth and Moon or somewhere between $10^{17}~\mbox{N}\,\mbox{m}/\mbox{yr}$ and $10^{19}~\mbox{N}\,\mbox{m}/\mbox{yr}$ (Phillips 1991; Golombek et al. 1992; Golombek 1994, 2002; Knapmeyer et al. 2006; Plesa et al. 2018). An average seismicity could therefore generate per year 2 quakes of moment larger than $10^{17}~\mbox{N}\,\mbox{m}$, 10 quakes with moment larger than $10^{16}~\mbox{N}\,\mbox{m}$ and 50 quakes with moment larger than $10^{15}~\mbox{N}\,\mbox{m}$. This leads us to design SEIS with a performance compatible for the surface wave detection of a quake with moment larger than $10^{16}~\mbox{N}\,\mbox{m}$ every were on the planet and the detection of high signal to noise body waves of the latter if occurring outside the core shadow zone. Although the landing site was mostly chosen with landing safety and long-term operations considerations. Cerberus Fossae is only $\sim 1500~\mbox{km}$ to the east-northeast from the InSight landing site and is one of the youngest tectonic features on Mars. It has been interpreted as a long graben system with cumulative offsets of 500 m or more (Vetterlein and Roberts 2010) and it contains boulder trails young enough to be preserved in eolian sediments (Roberts et al. 2012), indicative of large and perhaps very recent marsquakes large enough, if occurring again, to be recorded by the InSight instruments (Taylor et al. 2013).

Meteorite impacts provide another potential source of present-day seismic activity and are discussed in detail in Daubar et al. (2018), with a prediction of about 5 detectable events per year. The cratering rate on Mars can be estimated by extrapolating lunar isochrones to Mars (Hartmann 2005) or more directly from new impact craters detected using before and after orbital imagery (Malin et al. 2006; Daubar et al. 2013, 2016; Hartmann and Daubar 2017). Despite an orbital incomplete coverage, the agreement between estimates of the crater production function from these studies is typically within a factor of 2 or 3, with the Mars-observational studies suggesting fewer impacts. Larger uncertainties are in the estimation of the seismic signal amplitude generated by an impact. The conversion of the impactor momentum or energy is subject to several hypothesis. It is discussed in detail by Daubar et al. (2018) and significant differences exist between approach based on seismic efficiency (e.g. Teanby and Wookey 2011; Teanby 2015) and those computing directly the seismic equivalent source (e.g. Lognonné et al. 2009; Gudkova et al. 2011; Lognonné and Johnson 2015; Karakostas et al. 2018).

## SEIS Requirements

### Overview

SEIS requirements (e.g. the Instrument Level 2 requirements, provided in Table 3) have been designed to meet the InSight mission goal and L1 requirements, as listed in Table 1. Traditional seismic analysis is based largely on arrival times of body waves and direct surface wave acquired by a broadly distributed network of stations. In contrast, SEIS had in contrary to integrate explicitly the constraints of several single station analysis techniques developed for extracting Earth’s interior and seismic sources informations. This, in addition to the expected seismicity and seismic noise on Mars, was integrated in the experiment requirements, especially in the targeted sensitivity.

**Table 3:** SEIS instrument requirement (L2) flow and their link to the InSight mission requirements (L1). The SEIS requirements have led to the VBB performance requirements as indicated in Table 4

Mission requirement	SEIS instrument requirement
L1-1: Determine the depth of the crust-mantle boundary to within $\pm 10~\mbox{km}$	L2-1: Measure Rayleigh wave group velocity dispersion to ±5% for at least 2 quakes with SNR ≥ 3 on R3 wavetrains
L1-2: Detect velocity contrast $\geq 0.5~\mbox{km}/\mbox{s}$ over depth interval $\geq 5~\mbox{km}$ within the crust, if it exists	L2-2: Measure group velocity dispersion to ±4% for at least 3 quakes with SNR ≥ 3 on R3 wavetrains
L1-3: Determine seismic velocities in the upper 600 km of the mantle to within $\pm 0.25~\mbox{km}/\mbox{s}$	L2-3: Measure P and S arrival times to $\pm 2~\mbox{s}$ and R1 and R2 arrival times to $\pm 15~\mbox{s}$ for at least 13 quakes
L1-4: Positively distinguish between liquid and solid outer core^∗^	L2-4: Measure the Phobos tide amplitude to $\pm 2.5\times 10^{-11}~\mbox{m}/\mbox{s}^{2}$
L1-5: Determine the radius of core to within $\pm 200~\mbox{km}$	L2-5: Measure the Phobos tide amplitude to $\pm 3.3\times 10^{-11}~\mbox{m}/\mbox{s}^{2}$
L1-6: Determine the rate of seismic activity to within a factor of 2	L2-6: Measure marsquake signals of P-wave amplitude $\geq 6\times 10^{-9}~\mbox{m}/\mbox{s}^{2}$ with SNR ≥ 3
L1-7: Determine epicenter distance to ±25% and azimuth to $\pm 20~\mbox{degrees}$	L2-7: Measure the horizontal components of P-wave signals from $10^{16}~\mbox{N}\,\mbox{m}$ quakes with a SNR of ≥20
	L2-7b: Detect P and S-wave signals from $10^{16}~\mbox{N}\,\mbox{m}$ quakes at distances up to shadow zone with SNR ≥ 3
L1-8: Determine the rate of meteorite impacts to within a factor of 2	L2-8: Measure the seismic signals from meteorite impacts of P-wave amplitudes $\geq 3\times 10^{-9}~\mbox{m}/\mbox{s}^{2}$ with SNR ≥ 3

In this section, we first provide in Sect. 3.2 a general overview and review of the estimate of amplitude of seismic waves on Mars as a function of epicentral distance and seismic moment. In Sect. 0, we discuss the consequences of the single station approach for SEIS performances. We then present the instrument noise requirement and expected environmental noise (Sect. 3.4). Section 3.5 provides then an estimate of the expected number of quake detections and Sect. 3.6 provides an update and short critical review of new or challenging science goals prior to surface seismic operation. This identify new goals of the experiment which in many cases were considered at risk and not listed in the NASA 2012 non-published concept study report.

### Overview of Seismic Propagation on Mars

As compared with Earth, we expect to observe seismic event with lower magnitudes on Mars. We thus expect on Mars the data with the best signal-to-noise to be found in the bandwidth of body waves and regional surface waves (Lognonné and Johnson 2007, 2015). From seismograms, the most reliable seismological secondary data that could be extracted should be:

- travel times of body waves (in the short period range, 0.1–5 Hz),
- group and phase velocities of Rayleigh surface waves (in the long period range, 0.01–0.1 Hz),
- eigenfrequencies of spheroidal fundamental normal modes in the frequency range of 0.01–0.02 Hz.

Waveform polarization and estimates of azimuth should also be recoverable. Other data such as receiver functions, spheroidal normal modes below 0.01 Hz and overtones above 0.01 Hz, Love surface waves group and phase velocities or Toroidal modes eigenfrequencies might also be extracted but will be more sensitive to thermal or horizontal noise.

The analysis of the short period part of the seismic spectrum will be mainly devoted to obtaining information from the P and S waves that pass through the planet. The P-wave arrival time is the most robust measurement on a seismogram but inevitably, the waveforms to be recorded will look quite different from Earth. Except for quakes located close to the station, the seismic signal will be strongly reduced by the scattering in the crust due to the impacting history and by the attenuation of the planet (Lognonné and Johnson 2007, 2015). The importance of attenuation on Mars was originally pointed out by Goins and Lazarewicz (1979) who have shown that the Viking seismometer with a 4 Hz central frequency was unable to detect remote events due to attenuation.

While surface waves and quakes at small epicentral distances are expected to propagate mostly in the lithosphere, where the shear $Q _{\mu }$ is expected to be large, the deep Martian mantle will therefore likely have a relatively low $Q_{\mu }$, likely comparable or slightly larger that of Earth’s upper mantle one ($Q_{\mu } =140$ in the transition zone following Dziewonski and Anderson 1981) and much less than the $Q_{\mu }$ observed in the deep lunar interior ($Q_{\mu }=300\mbox{--}500$ following Toksöz et al. 1974). Proposed values range from $Q_{\mu } = 140$ (e.g. Khan et al. 2016) to about $Q_{\mu } =250$ from the use of the Anderson and Given (1982) model extended from Phobos’ tidal period to the seismic band (Lognonné and Mosser 1993; Zharkov and Gudkova 1997). We refer the reader to Smrekar et al. (2019, this issue), for more discussions on the a priori Mars intrinsic attenuation and its relation to the Martian mantle and discuss only in this section the implications in terms of seismic signal amplitudes.

This $Q_{\mu }$ is expected to be one of the major parameters influencing the detectability of remote activity. The amplitude ratio of the waves between two models depends on $e^{- \pi fT \Delta ( \frac{1}{Q})}$ where f is the frequency, $T$ the propagation time and $\Delta ( \frac{1}{Q} )$ is the difference of the inverse of $Q$ of the two models. For a 3 s period body wave and at 90° epicentral distance, for which the propagation time is roughly 800 s, this leads to an amplitude decrease by a factor of 3.3 for $Q_{\mu } = 140$ relative to $Q_{\mu } = 175$ and to an amplitude increase by a factor of 4.2 for $Q_{\mu } = 250$ again relative to $Q_{\mu } = 175$. This very high sensitivity of the body waves amplitudes to attenuation at large epicentral distance is a key difference to the Earth, for which the low lower mantle attenuation reduces attenuation loss at large epicentral distances. High sensitivity at long periods, due to its robustness to attenuation, was therefore considered as a critical requirement at the beginning of the OPTIMISM Broad Band seismometer (Lognonné and Mosser 1993; Lognonné et al. 1998) and Very Broad Band one (Lognonné et al. 1996, 2000).

**Fig. 2:**
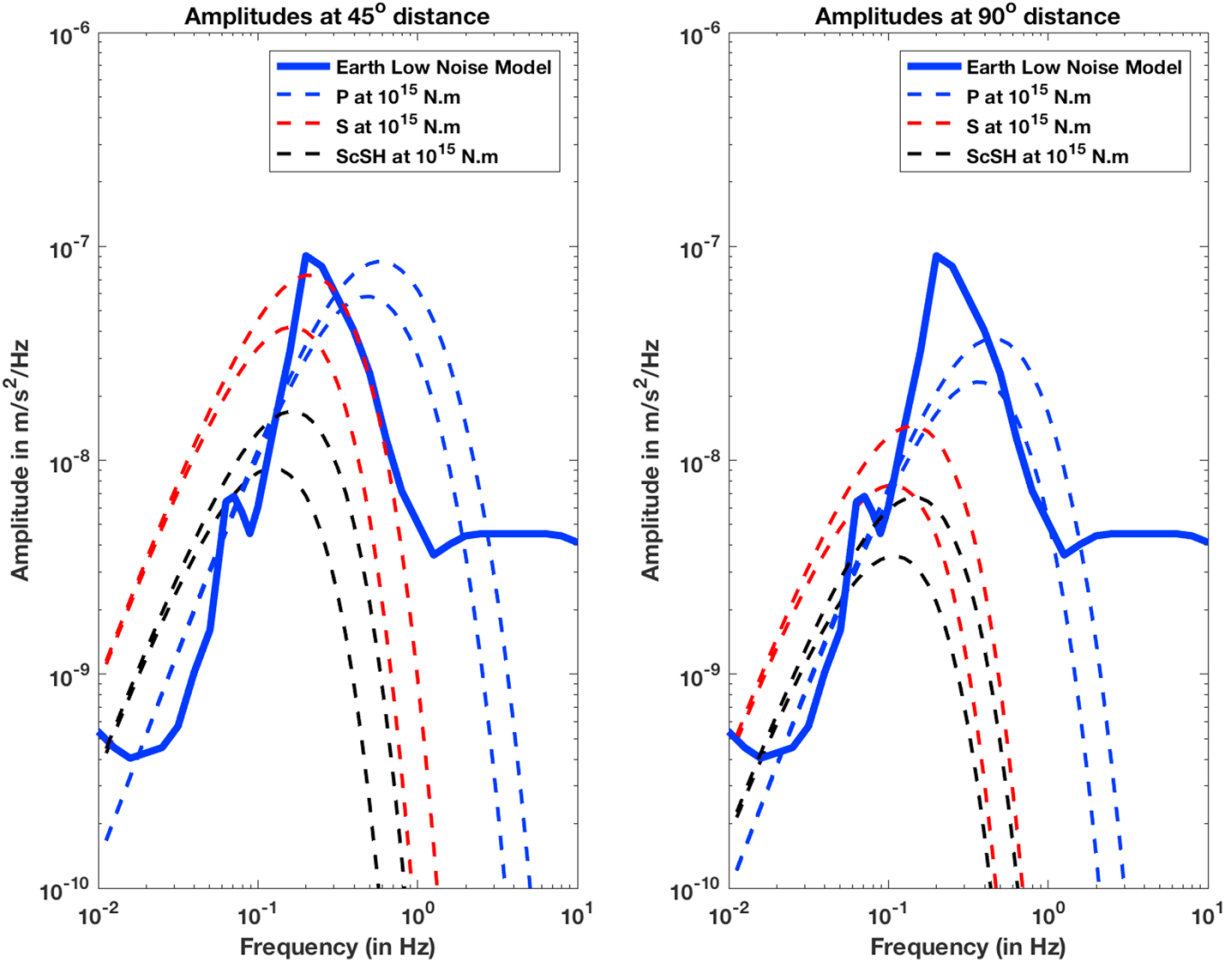
Body waves amplitude spectrum, for a 15 second window, as compared to the Earth Low Noise model (Peterson 1993) and for quakes of Moment $10^{15}~\mbox{N}\,\mbox{m}$ at 45° (left) and 90° (right) of epicentral distance computed with a Gaussian beam method. The two dashed curves are for a shear Q_*μ*_ of 250 (upper curve) and 175 (lower curve) respectively in blue for P waves and red for S waves. On Earth, these body waves signals would be hidden by the micro-seismic peak. Note nevertheless the strong cutoff of amplitude at a few Hz, which shows that most for distant events amplitude will be recorded below 2 Hz for P body waves and below 1 Hz for S body waves

**Fig. 3:**
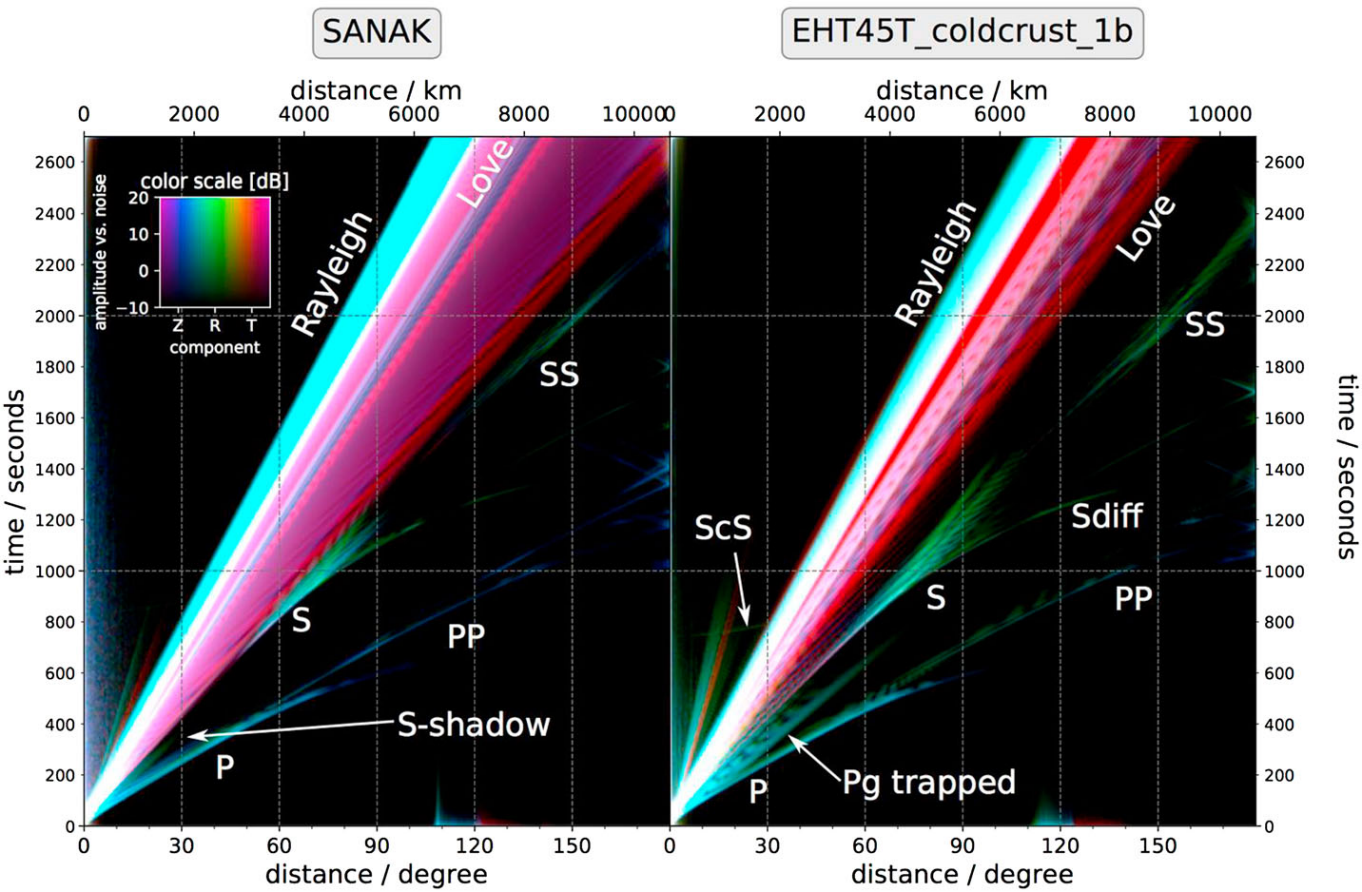
Global stack of synthetic seismogram envelopes for a magnitude 4 (moment $10^{15}~\mbox{N}\,\mbox{m}$) quake for two plausible Mars models, calculated using AxiSEM (Nissen-Meyer et al. 2014; van Driel et al. 2015). The seismograms were filtered with a noise-adapted filter suppressing all phases whose spectral power is below the noise level at all periods. In the plot, this corresponds to an amplitude of 0 dB. Note however, that phases with an amplitude of 0 dB can still be detectable, based on their polarization. Depth of the event is 10 km

Amplitudes of body waves were first estimated by Mocquet (1999), for an isotropic quake located at the surface with a seismic moment of 1015 N m and by Lognonné and Johnson (2015) for 1D models with a method enabling better amplitude modelling. Figure 2 shows expected body waves spectra for direct P, S and core reflected ScS waves, for the model A of Sohl and Spohn (1997) with P and S waves and for two attenuation models ($Q_{\mu } = 250$ and $Q_{\mu } = 175$ respectively, with corresponding $Q_{p} = 625$ and $Q_{p} = 440$). In the 0.5–2.5 Hz frequency band, the amplitudes of the P body waves decrease rapidly with epicentral distance and are smaller than S-wave amplitudes at the longest periods, whereas in the 0.1–1 Hz frequency band, amplitude is relatively independent of epicentral distance only for P waves. On Earth, scattering is very strong in volcanic regions, which suggests that significant scattering may occur in volcanic areas on Mars, particularly in the Tharsis region. Scattering mainly affects P waves and decreases the peak-to-peak amplitudes of body waves by producing conversions (P to SV, P to SH) and spreads this energy in time. For shallow quakes this effect will reduce the amplitude of the P waves near the source and the receiver and can decrease the P-wave energy by a factor of 10 (Lognonné and Johnson 2007). Figure 3 summarizes the amplitude over the full bandwidth of the expected signals as a function of epicentral distance for a magnitude 4 quake (moment $10^{15}~\mbox{N}\,\mbox{m}$), for two plausible Mars models. It can be seen that the SANAK model creates a much more extended surface wave train than the cold crust of the EH45T model. In both models, a broad S-shadow zone exists, but S-energy will come in very focused at distances between 60 and 90 degrees. A strong attenuation related to the low $Q_{\mu }$ factor is assumed for these two models (about 140 for $Q_{\mu }$ which follows models of Khan et al. 2016, 2018).

By sampling the crust, lithosphere and upper mantle, surface waves are the most important source of information to investigate the interior structure of Mars and will propagate mostly in the relatively cold lithosphere, in which attenuation might be much less than in the mantle (Lognonné et al. 1996). For instance, surface wave group velocities are very sensitive to the crustal thickness with 10% typical variations for crustal variations of 20 km (Lognonné and Johnson 2007, 2015). Though no surface waves were recorded on the Moon by the Apollo seismometers (e.g. Gagnepain-Beyneix et al. 2006), SEIS’s improved performance at long period and the expected larger magnitudes of quakes suggest the possibility of such detections on Mars.

In the framework of the InSight mission, Panning et al. (2015, 2017) proposed a single-station technique based on globe-circling surface Rayleigh waves measurements. It requires quakes with moments larger than 1016 N m and enable the location of quakes as well as inversion for crustal and upper-mantle structure. This technique is the key constraint on the SEIS performances as it implies the detection of R3. The consequences are provided in Sect. 3.3. Mars is expected to be less dispersive than the Earth and due to the smaller size of the planet, surface waves should therefore have a larger and more impulsive waveform than on Earth. This amplitude ratio with respect to Earth increases with angular epicentral distance and can reach a factor of 15 at 90° (Okal 1991).

All the modeling techniques used for these amplitudes modeling have of course been benchmarked with different techniques in 1D, such as normal modes summations (Lognonné and Mosser 1993; Lognonné et al. 1996), AxiSEM (Ceylan et al. 2017) and SPECFEM (Larmat et al. 2008; Bozdag et al. 2017). Even if less severe than on the Moon, diffraction of surface waves may however be effective at periods less than 10 s, due to the fracturing of the crust related to meteoritic impacts and might impact these analyses.

### Consequence of the Single Station Approach on the SEIS Performance

As noted above, one of the main drivers on the SEIS instrument requirements is associated with the *global* detection of R3 for $10^{16}~\mbox{N}\,\mbox{m}$ quakes, which are expected to occur at a rate of a few per year to a few tens per year.

**Fig. 4:**
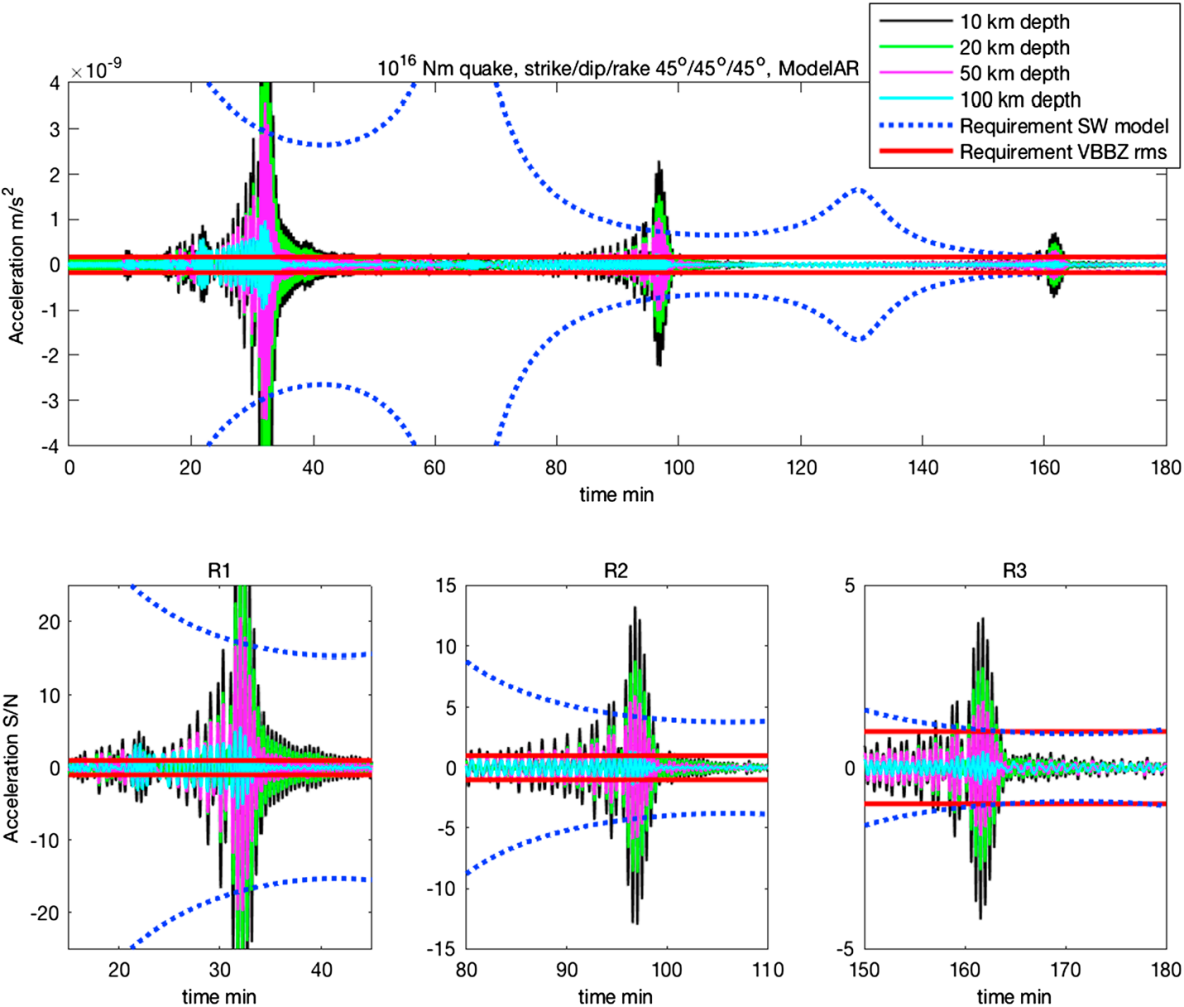
Normal mode summation synthetic seismograms for Mars shows large signals for multiple surface-wave arrivals from a $10^{16}~\mbox{N}\,\mbox{m}$ quake at a distance of $90^{\circ}$ (5500 km). Filtering to isolate the Rayleigh waves suppresses the P and S arrivals around 10 minutes, which are actually quite strong ($\mbox{SNR} >70$ in a 0.1–1 Hz band). Black, green, purple and cyan traces are for source depths of 10, 20, 50 and 100 km, respectively. Red lines denote the RMS noise level for $10^{-9}~\mbox{m}/\mbox{s}^{2}/\mbox{Hz}^{1/2}$ in amplitude spectral density in a bandwidth of 0.02–0.04 Hz, about $1.5\times 10^{- 10}~\mbox{m}/\mbox{s}$. Dashed blue provide the amplitude model which was used in the requirement flow

The consequences of these requirements are illustrated by Fig. 4 which provides an estimate of the amplitude of long period surface waves between periods of 25 s and 50 s, for a 1D model of Sohl and Spohn (1997). They are also listed in Table 4. Practically, the requirement of detecting the R3 surface waves, 10 times smaller than R1, is a major requirement for a single station mission. On the other hand, seismic network missions could indeed focus on the direct waves as soon as a sufficient number of stations are deployed. This was the case for the MESUR (**M**ars **E**nvironmental **SUR**vey, Solomon et al. 1991) and especially for the Impact (Banerdt et al. 1998) concepts were the performance requirement were related to the joint detection of the direct waves at more than 3 stations.

**Table 4:** The SEIS VBB performance requirements

Axis	Bandwidth	Instrument requirements	System requirement
Horizontal	[0.1–1] Hz	$10^{-9}~\mbox{m}/\mbox{s}^{2}/\sqrt{\mbox{Hz}}$	$2.5\times 10^{-9}~\mbox{m}/\mbox{s}^{2}/\sqrt{\mbox{Hz}}$
Vertical	[0.01–1] Hz	$10^{-9}~\mbox{m}/\mbox{s}^{2}/\sqrt{\mbox{Hz}}$	$2.5\times 10^{-9}~\mbox{m}/\mbox{s}^{2}/\sqrt{\mbox{Hz}}$
Vertical and horizontal (SP)	[0.2–15] Hz	$10^{-8}~\mbox{m}/\mbox{s}^{2}/\sqrt{\mbox{Hz}}$	
Vertical and horizontal (SP)	[15–50] Hz	$(f/15)^{2}\times 10^{-8}~\mbox{m}/\mbox{s}^{2}/\sqrt{\mbox{Hz}}$	

This, in addition to early estimates performed on the efficiency of simple surface thermal protection of Broad Band seismometers (e.g. Lognonné et al. 1996), leads to the $10^{-9}~\mbox{m}/\mbox{s}^{2}/\sqrt{\mbox{Hz}}$ requirement in the 0.01–1 Hz bandwidth of the vertical axis.

If this requires obviously electronic sensor self-noise below this noise level, this requirement requests also to mitigate all other source of noise and justified:

- the low temperature sensitivity of the seismic sensors and the significant thermal protection of the housing sphere (2 hours requirement time constant),
- the additional thermal protection and wind shield (5.5 hours requirements),
- the surface deployment of the SEIS sensor assembly with its wind and thermal shield (WTS),
- the minimization of all other sources of noise, including those from the tether, leveling system and packaging of the instruments,
- the inclusion in the payload of environmental sensors aiming to reduce the long period noise, when the latter is associated with either ground deformation associated with pressure fluctuations or magnetic field effects on the sensor (see more in Sect. 3.4).

For a temperature noise spectrum of $30~\mbox{K}/\sqrt{\mbox{Hz}}$ at 100 seconds (Mimoun et al. 2017) based on Viking measurement, the two-stages thermal protection attenuates the temperature by a factor of more than $5.6\times 10^{5}$ at 100 secs but nevertheless necessitates a temperature sensitivity of about $2\times 10^{-5}~\mbox{m}/\mbox{s}^{2}/{}^{\circ}\mbox{C}$ at the instrument level to reach the requirement.

### Instrument Noise

**Fig. 5:**
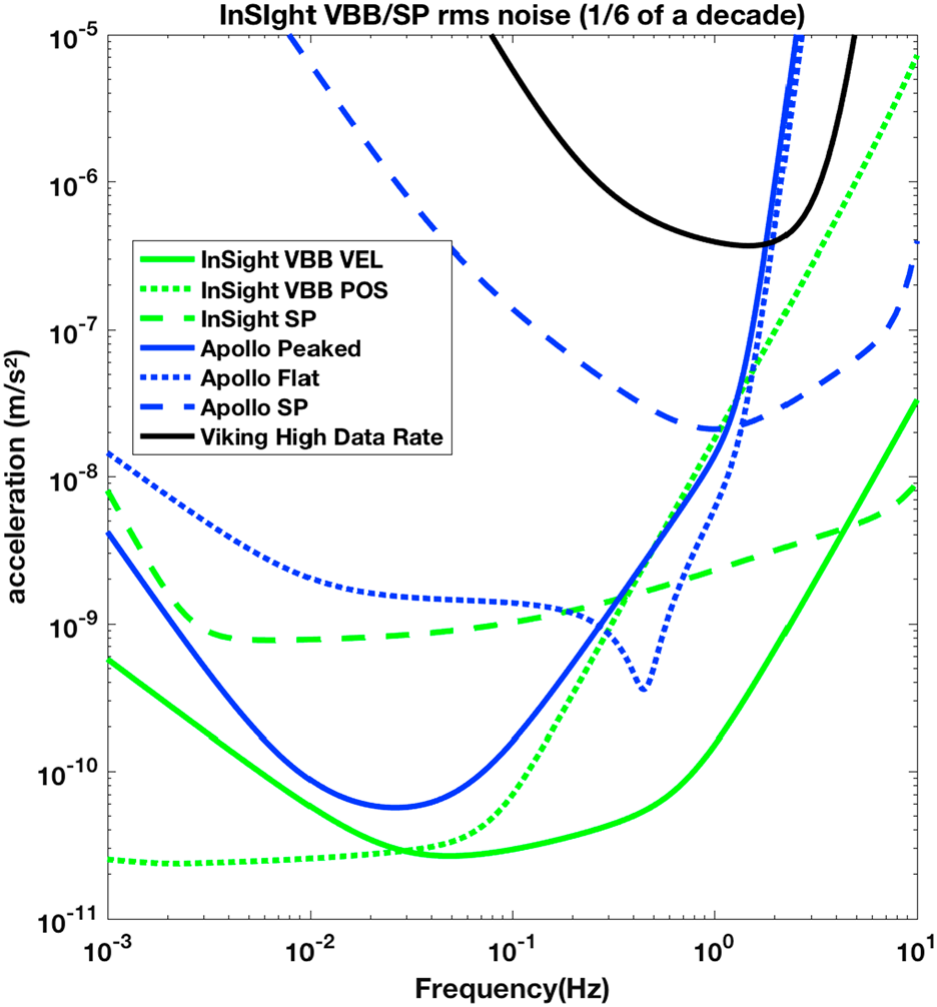
Root mean squared self-noise of the three main outputs of the SEIS instrument (VBB VEL, VBB POS and SP VEL), in acceleration for a $1/6$ of decade bandwidth, as a function of the central frequency of the bandwidth. This is compared to the Apollo and Viking resolution or LSB, as none of these instruments were able to record their self-noise due to limitations in the acquisition system for Apollo and Viking (9 bits plus sign for Apollo, 7 bits plus sign for Viking). SEIS uses acquisition at 23 bits plus sign

As concluded by Anderson et al. (1977a) after the Viking seismometer data analysis: “*One firm conclusion is that the natural background noise on Mars is low and that the wind is the prime noise source. It will be possible to reduce this noise by a factor of* $10^{3}$ *on future missions by removing the seismometer from the lander, operation of an extremely sensitive seismometer thus being possible on the surface*”. As shown on Fig. 5, an improvement of about 2500 at 1 Hz and 200 000 at 0.1 Hz is expected in terms of resolution, which however will likely be limited by the environmental noise associated with the interaction of Mars atmosphere and temperature variations with the SEIS assembly.

A complete noise model of the SEIS instrument has therefore been developed, where all sources of noise associated with the sensor interaction with the Martian environment are added to the self-noise of the SEIS instrument. This noise is extensively developed by Mimoun et al. (2017) and is only summarized in this paper. Specific noise contributions are also described by Murdoch et al. (2017a) and Murdoch et al. (2017b) for the source of wind-induced noise associated with the lander and the ground deformation respectively. With the estimation of the seismic amplitudes described in the previous section, this noise model allowed an estimation of the signal-to-noise ratio of seismic events, as a function of epicentral distance and magnitude (among other parameters) and therefore was vital to assess the success criteria of the experiment with respect to the achievement of its science goals. We have investigated the seismometer performance in three signal bandwidths: very low frequencies (typically $10^{-5}~\mbox{Hz}$ to detect tides), the [0.01–1 Hz] bandwidth to detect teleseismic signals and high frequency signal (e.g. asteroid impacts, local events…) that will be observable in the [1–20 Hz] bandwidth. In this section, we therefore only present the general approach developed by Mimoun et al. (2017) for the SEIS noise model, and discuss the major environmental assumptions and analyze the major and minor contributors to the model.

In the literature, noise analyses for seismometers often focus on the seismometer self-noise. This is due to the fact that most of the very broadband seismometers are operated inside seismic vaults with a very careful installation process (see e.g. Holcomb and Hutt 1993; McMillan 2002; Wielandt 2002; Trnkoczy et al. 2002), with very stable temperature conditions and with magnetic shielding (Forbriger et al. 2010). Despite all these efforts, the detection threshold of body waves on Earth is in addition limited by the minimum ambient Earth seismic noise, known as the low noise model e.g. Peterson (1993) and illustrated in Fig. 2.

The situation for SEIS is different: SEIS will be deployed on the surface of Mars, where the daily temperature variations can be larger than 80 K and the instrument has to integrate this major design constraint from the very beginning. In addition, the instrument will be installed on very low rigidity material and must be protected against all forces, either related to its tether link or to wind stresses, which will induce instrument displacements on the ground. The objective of the SEIS noise model is therefore double: first to provide an estimate of the instrument noise for the various bandwidths of interest and, secondly, to help refine, where necessary, the requirements of SEIS subsystems and of the various interfaces with the lander and HP3, including during the deployment. In some cases, the noise model had led us to consider including additional sensors on the InSight lander to help us decorrelate the seismometer output from the environmental contributions, as already illustrated on Earth for a magnetometer (Forbriger et al. 2010) and micro-barometer (Zürn and Widmer 1995; Beauduin et al. 1996; Zürn et al. 2007). See Murdoch et al. (2017b) for the implementation on InSight.

The first step was to build a seismic noise model identifying and evaluating all possible contributors, including the instrument self-noise and the instrument sensitivity to the external environment. This is described in detail in Mimoun et al. (2017) and is only briefly summarized here. This ensures that a complete estimate of the noise of the instrument in the Martian environment can be made. Then we have followed the performance maturation loop during the mission design and development. As is standard with any design process, all the parts of the system changed in their performance, from estimated values to measured and validated values. The noise model allows the consequences of the evolution of these performances to be tracked throughout the mission design and development process.

The noise requirements (see Table 4) have been defined based on:

- early Earth tests made at Pinion Flat Observatory with installation conditions comparable to those expected for InSight, including a tripod and windshield (Lognonné et al. 1996),
- seismic amplitudes estimation which indicated that these noise requirements are good enough for SEIS to detect a sufficient number of quakes during the operational life of the lander (1 Mars year $\sim 1.88$ Earth years).

The requirements are specified at both instrument and system levels and on both the vertical and horizontal axes. Note that the horizontal requirements extend down to 0.1 Hz and vertical axis requirements extend to 0.01 Hz. The large tilt sensitivity on the horizontal axes was indeed considered as too large to include science associated with Love surface waves without major risks in the threshold and baseline science goals and therefore in the mission requirement flow.

It was important during the process of evaluating the various possible noise sources to be very thorough in order to avoid forgetting an important noise contribution and we separated the source of instrument noise into two categories:

- Instrument noise (self-noise), which includes contributions from the sensor head, electronics and tether and weakly depends on the temperature, although the decrease of the Brownian and Johnson noise and, for the VBBs, the increase of the mechanical gain at cold temperature might slightly reduce the self-noise during winter
- Environmental effects including noise derived from instrument sensitivity to external perturbation sources (temperature variations including thermoelastic effects on the ground and sensor mounting, magnetic field, electrical field) and also the environmental effects generating ground acceleration or ground tilt (pressure signal, wind impact…).

This led to a noise map detailed in Mimoun et al. (2017) in the seismic bandwidth of the VBB (0.01–10 Hz) and in Pou et al. (2018) at very long periods. The quantification of these sources of noise has also been used to define the suites and performance requirements of the APSS sensors, when a source of environmental noise was found larger than the requirement but possibly mitigated by environmental decorrelation. A first example is the magnetic field sensitivity of the VBBs, associated with both micro-motors and spring magnetic properties. Its mitigation was either possible by a mu-metal shielding, too heavy with respect to mass constraints on the deployed sensor assembly, or by a magnetometer decorrelation, which was finally chosen for the implementation. A second example is the pressure decorrelation. In both cases, the admittance between the apparent ground acceleration and the perturbating signals (e.g. in $\mbox{m}\,\mbox{s}^{-2}/\mbox{nT}$ or $\mbox{m}\,\mbox{s}^{-2}/\mbox{Pa}$) was estimated and then used to define the performances of these sensors.

**Fig. 6:**
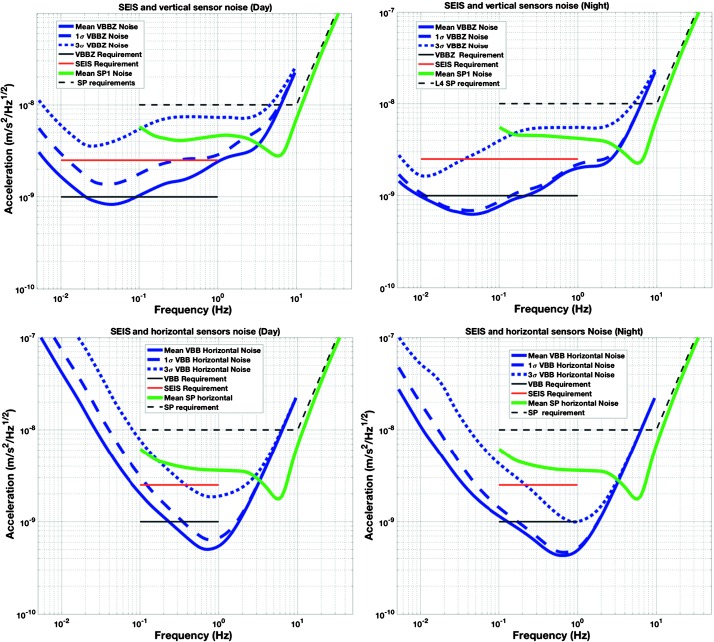
Instrument noise. Vertical (top two figures) and horizontal noises (bottom two figures) for the day (left) and night (right) environmental conditions. Horizontal black and red lines represent the instrument performance requirements for the VBB self-noise and SEIS full noise (with environmental ones). Performances are presented for mean (50%), nominal $1\sigma $ (70%) and worst case $3\sigma $ (95%) conditions, respectively in dashed, dot-dashed and solid lines for the VBBs. Dashed black curve represents the SP sensor requirement while the green continuous line is the expected SP noise with environment for mean conditions. Curves are provided in the VBB and SP bandwidth, respectively [0.01–10 Hz] and [0.1–50 Hz]

The instrument noise summary is depicted in detail in Fig. 6 for both the vertical and horizontal VBBs and SPs, which summarizes the results of Mimoun et al. (2017) for the VBBs and extend the latter to the SPs. During the night, we expect the noise to be below $2.5\times 10^{-9}~\mbox{m}/\mbox{s}^{2}/\mbox{Hz}^{1/2}$ in the body wave bandwidth and to be close to the Earth low noise model down to 0.02 Hz, e.g. 50 s. At longer period and despite the strong thermal protection, thermal noise is expected to grow rapidly, in way very similar to that observed at Pinion Flat Observatory (PFO) during tests made on the Earth’s surface in desert areas by Lognonné et al. (1996). The environmental noise will peak over the self-noise of the SP only at the 3-sigma level and most of the time, we expect the SP to be limited by its self-noise.

### Seismic Event Signal-to-Noise and Frequency

Detailed analyses have shown that all instrument requirements listed in Table 3 and related to quakes can be fulfilled with the Mars activity as described in Sect. 2.3, noise level as predicted by the instrument noise model of Sect. 3.4 and with seismic waves propagation models from expected structure as described in Sects. 2.2 and 3.2. We provide here only two examples and leave others to Fig. 3, which uses synthetics to capture the signal-to-noise estimation of the different seismic phases of an $M=10^{15}~\mbox{N}\,\mbox{m}$ marsquake seismogram.

The frequency of seismic events for the estimation of total seismic moment release per year and the slope of the negative power law that defines the number of marsquakes of any size have been determined for different assumed maximum marsquakes. Intermediate estimates suggest hundreds of marsquakes per year with seismic moments above $10^{13}~\mbox{N}\,\mbox{m}$, which is approximately the minimum magnitude for detection of P waves at sufficient signal to noise ratio at epicentral distances up to $60^{\circ}$ (Mocquet 1999; Teanby and Wookey 2011; Böse et al. 2017; Clinton et al. 2017, 2018). In addition, there should be 4–40 teleseismic events (i.e., globally detectable) per year, which are estimated to have a seismic moment release of $\sim 10^{15}~\mbox{N}\,\mbox{m}$ and 1–10 events per year large enough to produce detectable surface waves propagating completely around the planet, which are suitable for additional techniques in source location (Panning et al. 2015) and fulfill requirements L2-1 with one Mars year of operation.

As shown on Fig. 6, noise in an octave bandwidth around 1 Hz is expected to be in the range of $2\mbox{--}3 \times 10^{-9}~\mbox{m}/\mbox{s}^{2}/\mbox{Hz}^{1/2}$, one order of magnitude smaller than P wave amplitudes at 90° of epicentral distance and for a $M=10^{15}~\mbox{N}\,\mbox{m}$ moment (Fig. 2). For S waves, the amplitude peak will occur at longer period and for one octave around 0.1 Hz, with a ratio of about 5. This illustrates the fulfillment of requirement L2-7b of Table 3.

**Fig. 7:**
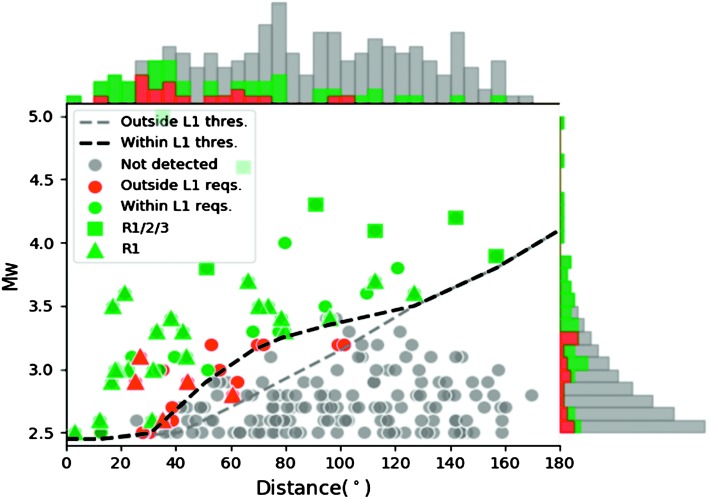
Summary of Marsquake Service performance in the Blind Test. All events included in the one year of data are shown. MQS detected the events shown in red and green, those in green meet L1 requirements. Squares indicate the events located using R1/R2/R3, triangles were located with R1 and circles are for only P and S waves. The grey curve indicates the limit threshold for detection and the black curve the location threshold, as a function of distance. See details in Clinton et al. (2018)

More representative tests were done in the frame of the Blind test proposed by Clinton et al. (2017). Analysis of one (earth) year of data of synthetic quakes with the current best estimate of the noise model was performed with the tools of the MarsQuake Service (MQS) as well as those from other test participants. Figure 7 shows the results from MQS, indicating that 7 quakes were detected and located using R1/R2/R3, with additional 27 quakes with only R1. Practically, the detection of R1/R2 only is most of the time rare as R3 can then be detected even in low signal to noise conditions. Over one Martian year, this is comparable to L2-3 and much more than L2-1. In addition, this test was made without pressure decorrelation, which could significantly improve the number of detected Rayleigh waves at long periods.

Last but not least and as noted in Sect. A.5 of Appendix A, an event large enough to create observable excitation of the planet’s free oscillation (seismic moment of $\sim 10^{18}~\mbox{N}\,\mbox{m}$) may be expected to occur during the nominal mission if the seismic activity level is near the upper bound of the range of reasonable estimates.

### Challenges and New Science Goals

#### Pressure and Environmental Decorrelation

**Fig. 8:**
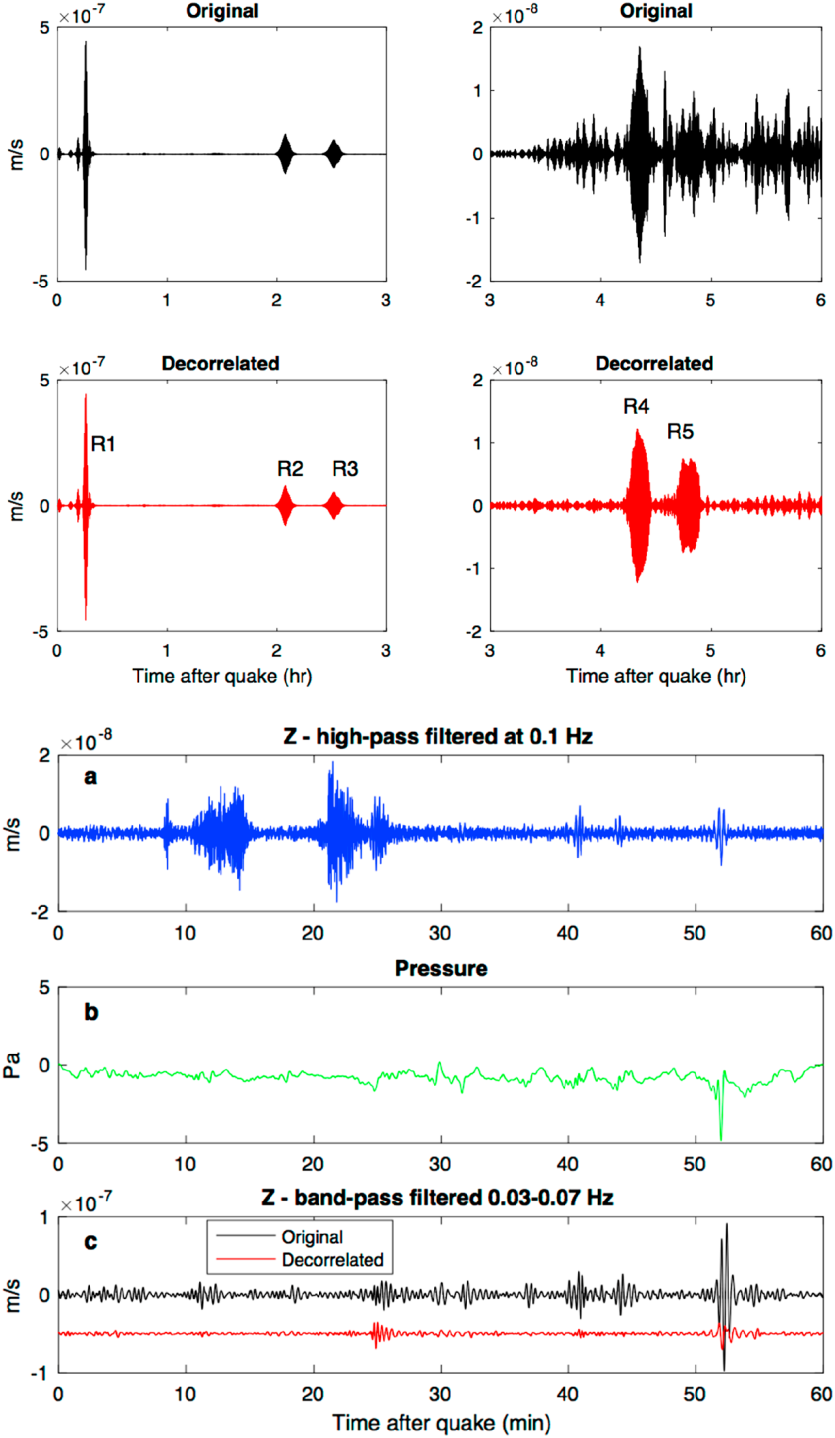
Example of pressure decorrelation efficiency from synthetic tests following the techniques of Murdoch et al. (2017b). Event on the left is the one of the blind test data on 22/09/2019 and pressure decorrelation will enable much better long observations and therefore normal modes. The right example shows the R1 of a smaller quake on 27/03/2019. The bottom trace in black shows a clear detection of R1

As indicated in Sect. 3.4 and described in detail in Mimoun et al. (2017), the pressure noise associated with the low rigidity surface deformation will likely be the limiting factor at both long periods ($T>15\mbox{--}20~\mbox{s}$) and possibly at short period during the day. The efficiency of pressure decorrelation proposed by Murdoch et al. (2017b) will likely improve significantly the detection and analysis of quakes. This is illustrated with synthetic waveforms realized for the Marsquake Service blind test (Clinton et al. 2017), which include realistic pressure noise from Large Eddy Simulation. Figure 8 shows the benefit on the largest quake in the blind test catalog: $M_{w}=5$ quake at $35^{\circ}$ epicentral distance. Since the quake happens during the nighttime, no decorrelation is applied for the first three hours after the origin time. After that, however, an improvement in the SNR of multi-orbiting Rayleigh waves is achieved by decorrelation in the 20–30 s band. A second example for the $M_{w}=3.7$ quake at $66^{\circ}$ epicentral distance is also shown. In this case, the quake occurs during the daytime and pressure decorrelation helps in identifying the surface-wave trains. Indeed, if the body-wave arrivals are clearly visible in the high-pass filtered data, the Rayleigh waves are partially hidden by the background noise. These perspectives explain not only the integration of the APSS suite in the InSight payload (Banfield et al. 2018) but also why APSS data will be distributed by the SEIS ground system to seismological users in the same SEED format as seismic data (see Sect. 9.2).

#### Natural Impacts (L1-8)

Meteorite impacts provide an additional source of seismic events for analysis. On the Moon, impacts constitute $\sim 20\%$ of all observed events and a similarly large number are expected for Mars: for a comparable mass, the frequency of impact is $2\mbox{--}4{\times}$ larger for Mars but the velocity is $\sim 2{\times}$ smaller because of deceleration in the atmosphere (even less for the smallest events). The Apollo 14 seismometer detected about 100 events per year generating ground velocity larger than $10^{-9}~\mbox{m}/\mbox{s}$ and 10 per year with ground velocity larger than $10^{-8}~\mbox{m}/\mbox{s}$ (Lognonné et al. 2009).

Large uncertainties remain prior to landing on the amplitude of impacts signal and on the atmospheric noise in the relatively high frequency bandwidth, were body waves of impacts might peak (0.5–3.5 Hz) but where also the self-noise of the pressure sensor might prevent any good pressure decorrelation.

Most of these uncertainties are related to the impact equivalent sources and are added to those in the impact rate, for which uncertainty of a factor 2–3 remains. Nevertheless, using estimates of impactor flux, seismic efficiency and crater scaling laws, Teanby and Wookey (2011) predict that globally detectable impacts are rare, with an estimate of $\sim 1$ large event per year. Regional decameter-scale impacts, within $\sim 2000\mbox{--}3000~\mbox{km}$ of the lander, are more frequent with $\sim 10$ detectable events predicted per year (Teanby 2015). These estimates are consistent with those by Lognonné and Johnson (2015) using an independent approach based on seismic impulse in the long-period limit. Nevertheless and even if a few impacts can be detected seismically and located from orbital images, the potential for constraining the crustal structure will be much greater than for a single marsquake because the source location will be defined, removing a major unknown. The search in orbital images for fresh impacts after a seismic signal detection associated with shallow sources is thus an important part of the InSight SEIS investigation (Daubar et al. 2018). See also more on impact estimates in Gudkova et al. (2015), Yasui et al. (2015), Lognonné and Kawamura (2015), Güldemeister and Wünnemann (2017) and in airbursts estimates in Stevanović et al. (2017), Garcia et al. (2017), Karakostas et al. (2018).

#### Phobos Tide (L1-5)

**Fig. 9:**
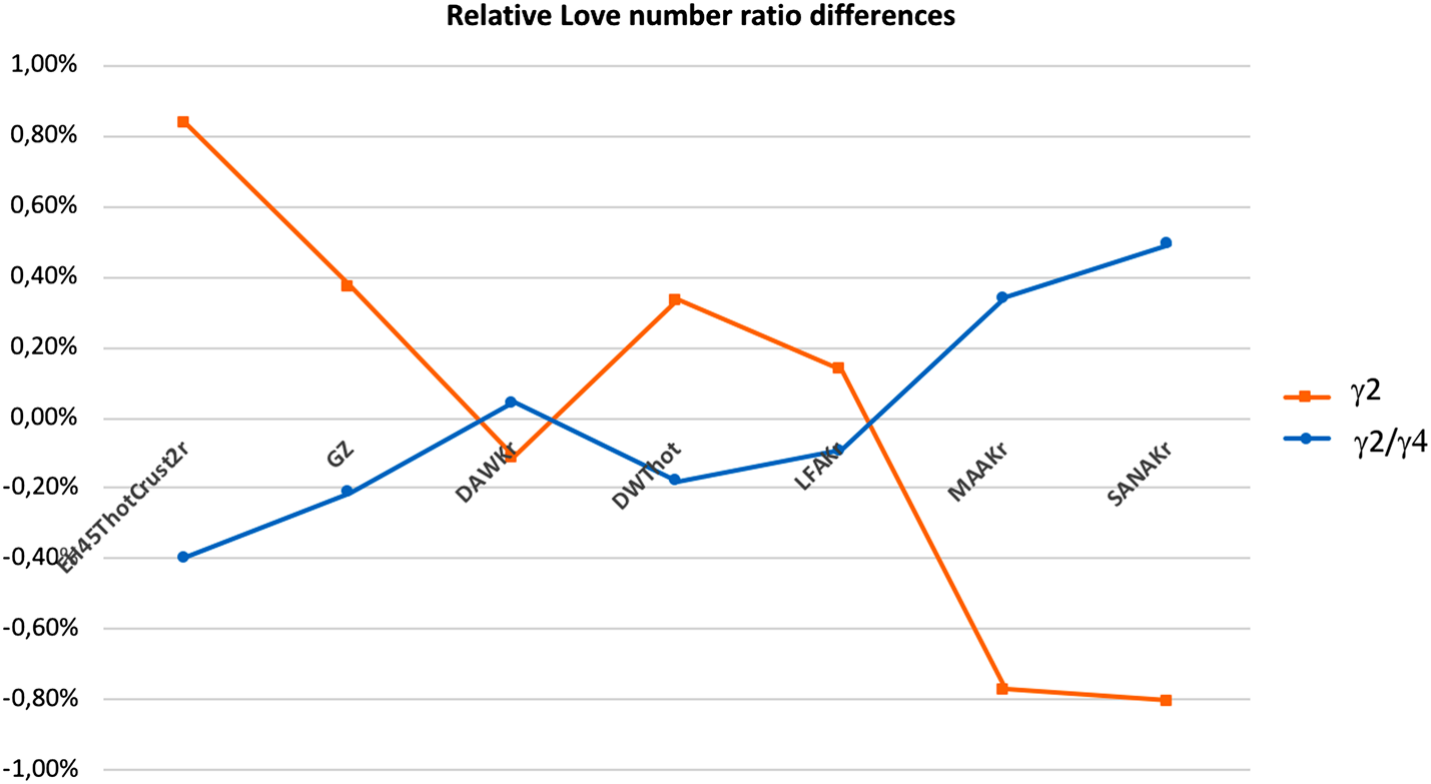
Deviation of the Phobos gravimetric factor l=2, m=2 (in red) and of the ratio between the $l=2$, $m=2$ and $l=4$, $m=4$ factors in blue. The second one varies by about $\pm 0.4\%$ for the range of a-priori models but will not depend on an absolute calibration of SEIS

The Phobos tides, which are $\sim 0.5\times 10^{-8}\mbox{ m}/\mbox{s}^{2}$, are subdiurnal with periods of about 5.5 hr (Van Hoolst et al. 2003). They are thus below the primary seismic frequency range and provide a unique link between high frequency (seismic) and ultra-low frequency (geodetic) observations of Mars’ interior and provides, through their gravimetric factor, constraints on the core (Lognonné and Mosser 1993; Van Hoolst et al. 2003; Panning et al. 2017). The measurement is essentially limited by the temperature noise ($\sim 0.5~\mbox{K}\,\mbox{rms}$ in a bandwidth of 1 mHz around the Phobos orbital frequency) and by calibration precision of the gravimetric output of seismometers. If the first source of noise can be mitigated due to the non-synchronized period of Phobos tide with the sol harmonics, the second is a systematic source of error and will be the limiting factor. Figure 9 illustrates the challenge, by showing the differences in the gravimetric factor associated with the $l=2$, $m=2$ Phobos tide for the different models listed by Smrekar et al. (2019, this issue). Section 7.3.2 and Pou et al. (2018) are reporting the perspective of absolute calibration of the VBBs or SPs output, suggesting a conservative target of 0.5% of calibration error. This only allows distinguishing the extreme sides of the models. But as noted by Lognonné et al. (1996), Van Hoolst et al. (2003), Phobos is close enough to Mars to have a relatively large $l=4$, $m=4$ harmonic with an amplitude 5.5 times smaller for a frequency about 2 times higher. This will provide an alternative for characterization of the core, by using as proxy the ratio between the $l=2$, $m=2$ and the $l=4$, $m=4$, which will be not be impacted by the absolute error in the calibration, as the latter cancels in such a ratio. Although smaller by a factor of two (see Fig. 9), this will provide additional constraints on the interior, independent of the planet seismicity. More physically, this will balance the tidal impact of the upper mantle ($l=4$, $m=4$) with respect to the whole planet tide ($l=2$, $m=2$). Such analysis will be performed in the framework of Mars Structure Service activities (Panning et al. 2017 and Sect. 8.3).

#### New Science Goals

Last but not least, new science investigations on the Martian subsurface, which were not initially integrated in the CSR, will be conducted. The first one will be associated with the monitoring of the HP3 generated seismic signal, when HP3 will penetrate the ground. This will allow both the measurement of body waves (Kedar et al. 2017) and also possibly first attempt in 6 axis seismology by using the 6 SEIS sensors (Fayon et al. 2018), even if this will require a careful processing of the SEIS data during the passive and active cross-calibration phases (see Sect. 7.3).

Other investigations include joint data analysis of the SEIS and APSS data that will use SEIS to investigate signals associated with the lander wind generated noise (Murdoch et al. 2017a), dust devils and atmospheric boundary layer activity generated ground deformation (Lorenz et al. 2015; Murdoch et al. 2017b; Kenda et al. 2017), dust devil detection (Lorenz et al. 2015; Kenda et al. 2017) or short period Rayleigh waves (Kenda et al. 2017; Knapmeyer-Endrun et al. 2017). These signals will not only help us to determine the subsurface structure, but might also provide a new tool for monitoring the atmosphere (Spiga et al. 2018)

## SEIS Description

### SEIS Overall Description

Both the VBB and the SP are feedback seismometers based on capacitive transducers and are inheriting from the development of the very broad band seismometers on Earth since the early 1980 (e.g. Wielandt and Streckeisen 1982). For more information on the broad band seismometers, see e.g. Wielandt (2002) and Ackerley (2014). For a review on past planetary seismometers, see Lognonné (2005), Lognonné and Pike (2015). Due to mass, launch and space environment, space qualification technology limitation and the very large temperature variation on Mars, the SEIS experiment has however been entirely designed for the purpose of planetary seismometry. We provide in the section a first overview, followed by a more detailed description of the different subsystems of SEIS in Sect. 5. Section 6 describes performance and instrument noise and transfer function and Sect. 7 their operation.

#### Overview

**Fig. 10:**
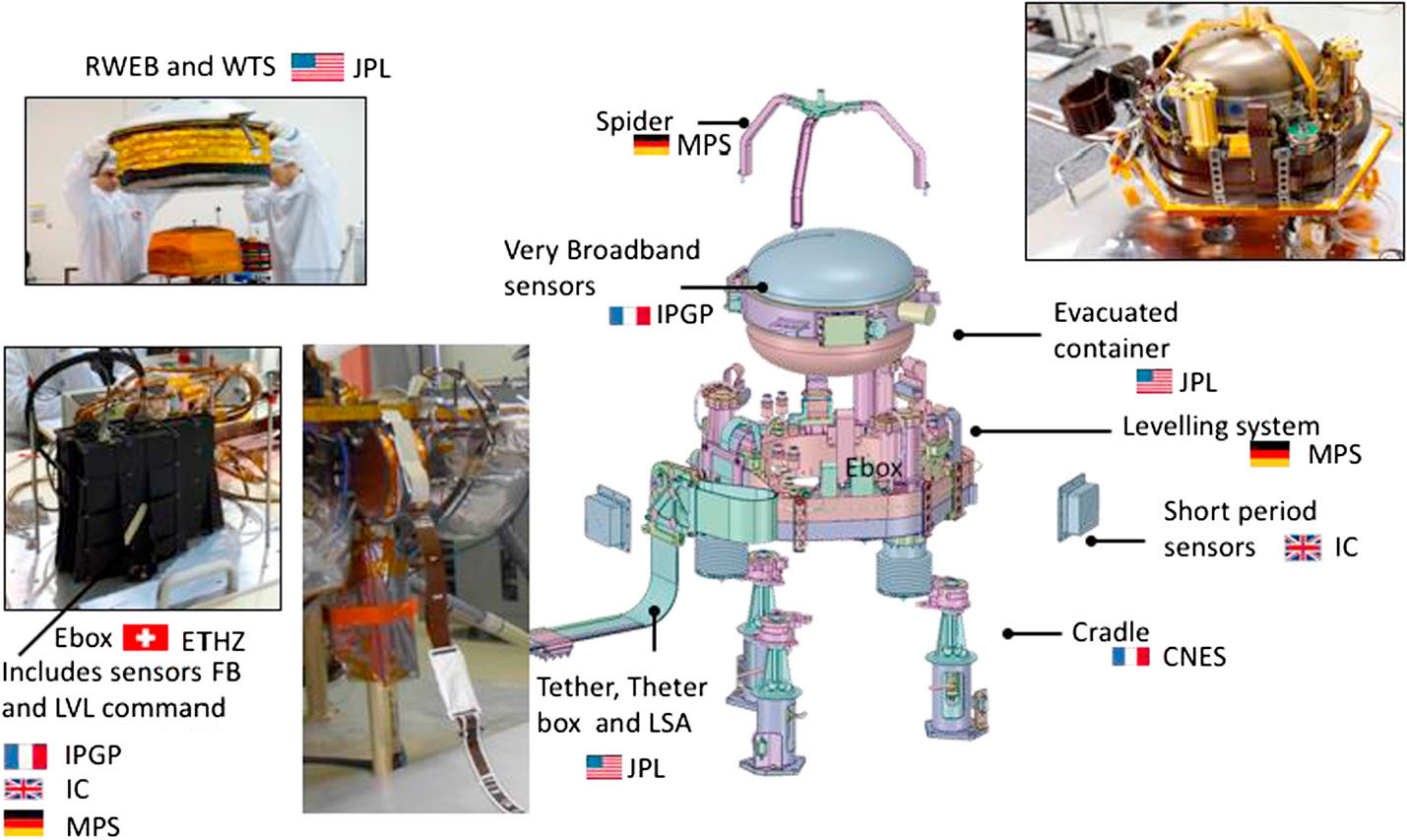
SEIS experiment subsystems, together with the institutions leading the subsystems

**Fig. 11:**
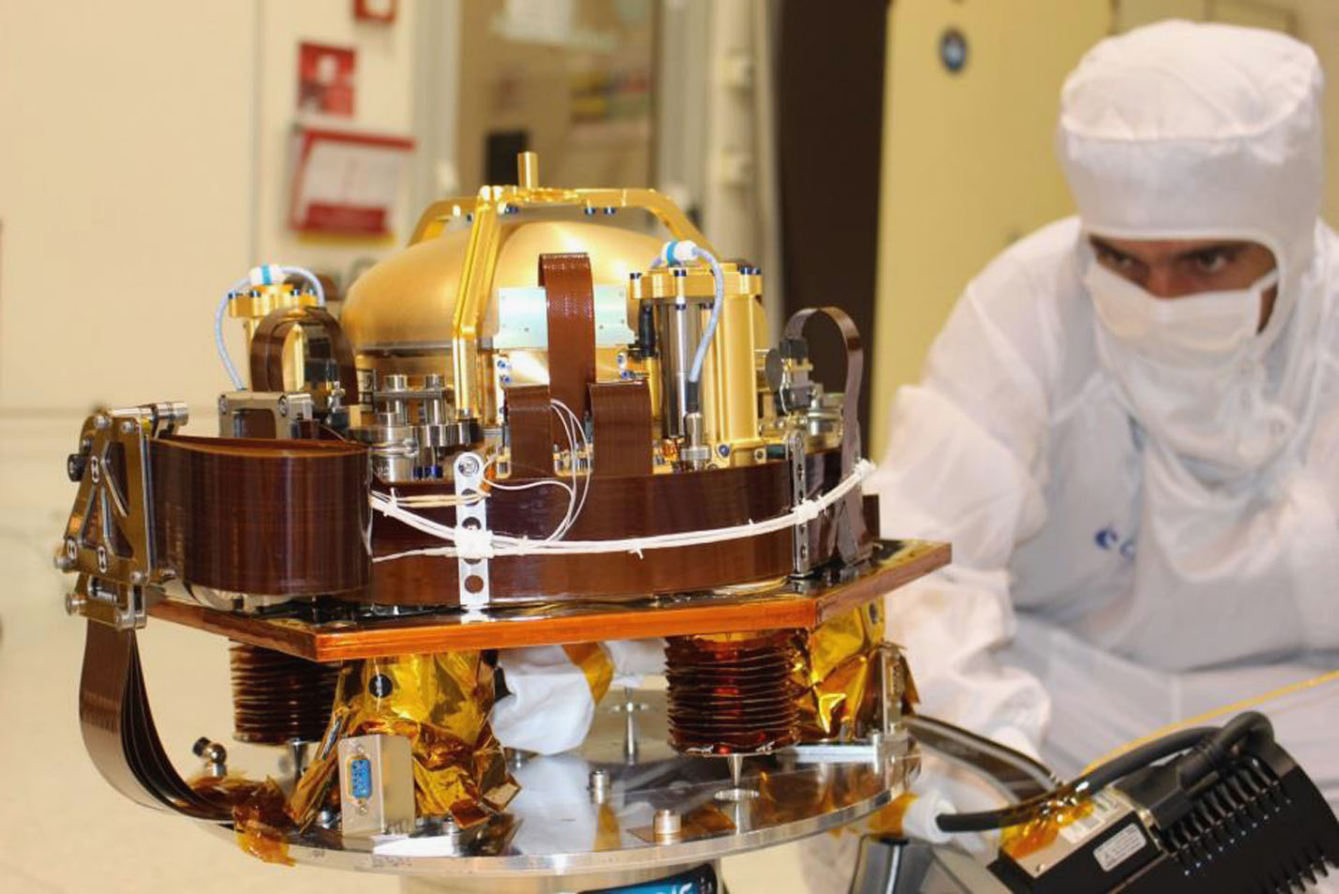
SEIS Sensor Assembly (SA) without the RWEB

**Fig. 12:**
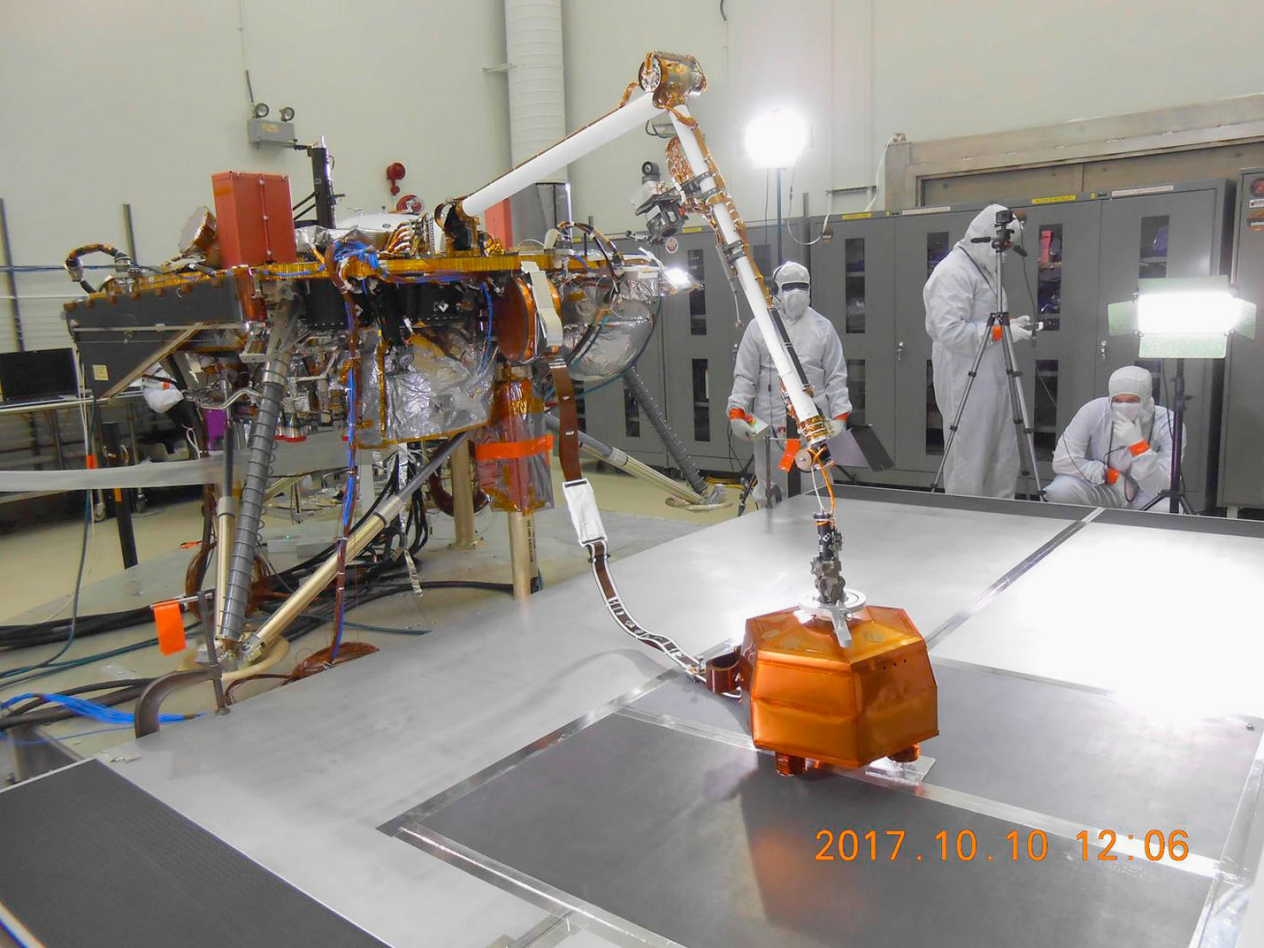
The Sensor Assembly being deployed on the ground during DST#3 (Deployment System Test). Two segments of the tether (TSA-1, part of TSA-), the Tether Storage Box, Field Joint and part of the Load Shunt Assembly are also visible

The SEIS instrument has 4 main components (Fig. 10):

- The Sensor Assembly (SA) (Fig. 11). It accommodates two independent, 3 axes seismometers: a Very Broad Band (VBB) oblique seismometer and a miniature Short Period (SP) seismometer. Both seismometers and their respective signal preamplifier stages are mounted on a common structure which can be precisely levelled thanks to 3 tunable length legs. They are protected against thermal noise by a thermal blanket (RWEB, the Remote Warm Enclosure Box). The Sensor Assembly is stored on the lander’s deck for launch, cruise to Mars, EDL (Entry, Descent and Landing) and the first days of operation on Mars. It is then deployed on the ground of the planet with a motorized arm (Fig. 12). Drawing of the SA is provided in supplementary material 1.
- The EBox (Electronic Box), a set of electronic cards located inside the lander’s thermal enclosure.
- The tether that makes the electrical link between the SA and the EBox.
- The WTS (Wind and Thermal Shield) that is deployed after the SA and over it. It gives an extra protection against winds and temperature variations.

Funding of all subsystems has been made without fund transfer and has been therefore supported by the French (CNES), US (NASA within JPL Discovery contract), German (DLR), Swiss (SSO) and UK (UKSA) space agencies. Additional human resources support has been made by the national academic and research organizations. See details in the Acknowledgement section. CNES has in addition done the overall project management and ESA has managed the Swiss ETHZ contribution through PRODEX.

#### SEIS Seismic Sensors

SEIS has 2 sets of three axis seismometers, the VBB sensors and the SP sensors, each described in more detail in Sects. 5.1 and 5.2. In this section, we focus on comparing the sensors. The VBBs were developed by IPGP since the end of the 1990 following CNES R&D. For the pendulum, early prototypes, InSight qualification, engineering and the flight units were built by SODERN. EREMS was in charge of the InSight VBB feedback cards. SPs sensors have been designed by Imperial College and the electronics are developed by Oxford University and Kinemetrics. Both the VBBs and SPs are inherited from developments initiated in the mid-1990s by the InterMarsnet and Marsnet ESA-NASA projects. The joint VBB and SP configuration was also the baseline for the NetLander project (Lognonné et al. 2003), at that time with 2 VBBs and 2 SPs (Lognonné et al. 2000).

VBBs are oblique sensors, recording U, V, W ground velocity in a non-Galperin configuration and with a tilt with respect to ground horizontal of about 30°, while the SPs are vertical (for SP1) and horizontal (for SP2 and SP3). Both the VBBs and SPs are feedback sensors with their feedback cards inside the lander thermal enclosures, proximity electronics on the sensor assembly and with analogue feedback signals transmitted in the tether. The VBBs however have an increased built-in robustness, as each VBB axis is completely autonomous, including the quartz oscillator driving the displacement sensor. The 3 SP axes on the other hand all share the same oscillator and their 3 feedback circuits are integrated on a single electronic board. In comparison, a failure of any VBB axis prevents the synthesis of a vertical output, while SP1 can provide this irrespective of the failure of SP2 or SP3. Taken together, in their common bandwidth the VBBs and SPs will provide fully redundant 3 axes seismic measurements and any failing axis of the VBB (or SP) can be replaced by any other of the SP (or VBB), as no VBB axis is parallel to any SP axis. This configuration in addition offers the possibility, when all 6 sensor axes operate nominally, to perform 6 axes high frequency seismological measurements, as developed by Fayon et al. (2018).

**Fig. 13:**
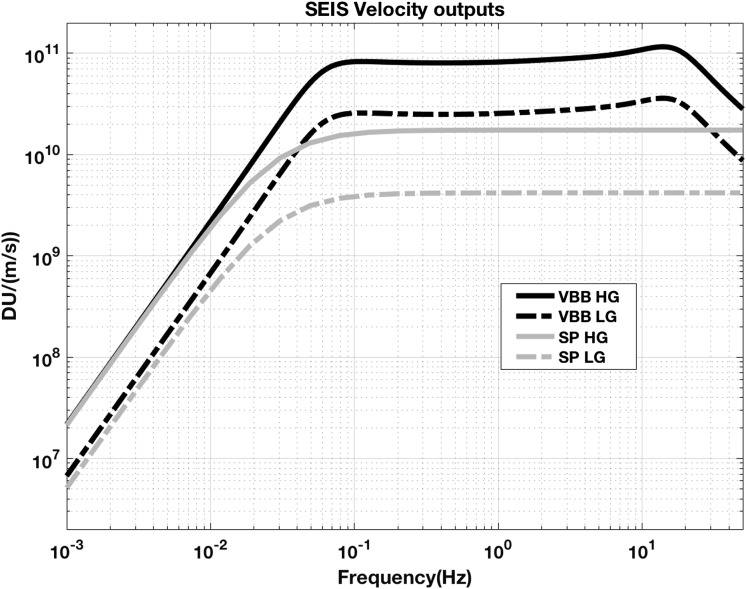
Comparison of the VBBs and SPs transfer functions. Very long period gains are similar while the VBB gain is a factor of 4–5 times larger between 20 s and 10 Hz. All gains are in Digital Unit (DU) per ground velocity ($\mbox{m}/\mbox{s}$)

VBB sensors target the monitoring of the 0.01–5 Hz bandwidth, while the SPs target the 0.1–50 Hz bandwidth. Because of their different natural frequencies (0.5 Hz for the VBBs and 6 Hz for the SPs), VBBs have a larger mechanical gain ($>0.11~\mbox{s}^{2}$) than SPs ($7\times 10^{-4}~\mbox{s}^{2}$), but lower high-frequency cut-off frequencies than the SPs. VBBs therefore demonstrates better performances at long periods than the SPs, while the latter are best at short periods. VBBs were therefore required to meet a self-noise better than $10^{-9}~\mbox{m}/\mbox{s}^{2}/\mbox{Hz}^{1/2}$ between 0.01 Hz and 1 Hz, while the SPs were required to meet a self-noise better than $10^{-8}~\mbox{m}/\mbox{s}^{2}/\mbox{Hz}^{1/2}$ between 0.1 Hz and 1 Hz (see Table 4). Their performances are comparable between 3–5 Hz (depending on tests results) and their transfer functions are compared, for the velocity outputs in Fig. 13.

On the other hand, the factor 100 larger mechanical gain makes the VBBs much more sensitive to installation tilt: VBBs must therefore be levelled and can operate in nominal configuration without saturation up to about 0.25° and 0.02° degrees of tilt in their lowest and highest sensitivity mode, while the SPs can operate under up to 15° of tilt. Each VBB can however still operate in non-nominal conditions for tilts of their sensitivity axis from $-2.8^{\circ}$ to $3.5^{\circ}$, but with a free frequency varying from unstable (about $i\times 0.2~\mbox{Hz}$, where $i$ is the imaginary number such that $i^{2}=-1$) to 0.70 Hz. The feedback is nevertheless strong enough to accommodate unstable free frequencies. The three VBBs can therefore operate in a non-nominal mode within $\pm 2.5^{\circ}$ of tilts of the levelling system (LVL).

The baseline acquisition rate of the VBBs and SPs are 20 and 100 samples per second (sps) respectively, both being acquired with a 24 bit acquisition system. In the common seismic bandwidth (0.1–5 Hz), the output of both VBBs and SPs are flat in velocity in their nominal mode and the low gain of the VBBs is about 55% larger than the high gain of the SPs ($2.8\times 10^{10}~\mbox{DU}\,\mbox{s}/\mbox{m}$ and $1.8\times 10^{10}~\mbox{DU}\,\mbox{s}/\mbox{m}$). The high gain of the VBB is about 5 times larger than the high gain of the SP. As both sensors are feedback sensors, their long period noise in their velocity flat mode is both related to the electronic feedback self-noise and the displacement transducer noise. With their 10 times lower requirement than VBBs at 0.1 Hz but comparable space qualified technologies for the feedback amplifiers, the SPs velocity self-noise at 0.1 Hz is mostly related to the displacement transducer noise, while the VBBs displacement and feedback noise are comparable at 0.01 Hz even with the larger electronic gain. VBBs have therefore, in addition to their velocity output, a DC coupled, flat in acceleration, very long period output (POS), which is much less sensitive to the integrator feedback noise. The SPs have also this output, but the POS SP output is acquired only with a 12 bit A/D converter while the VBB one is acquired with a 24 bit A/D converter.

**Fig. 14:**
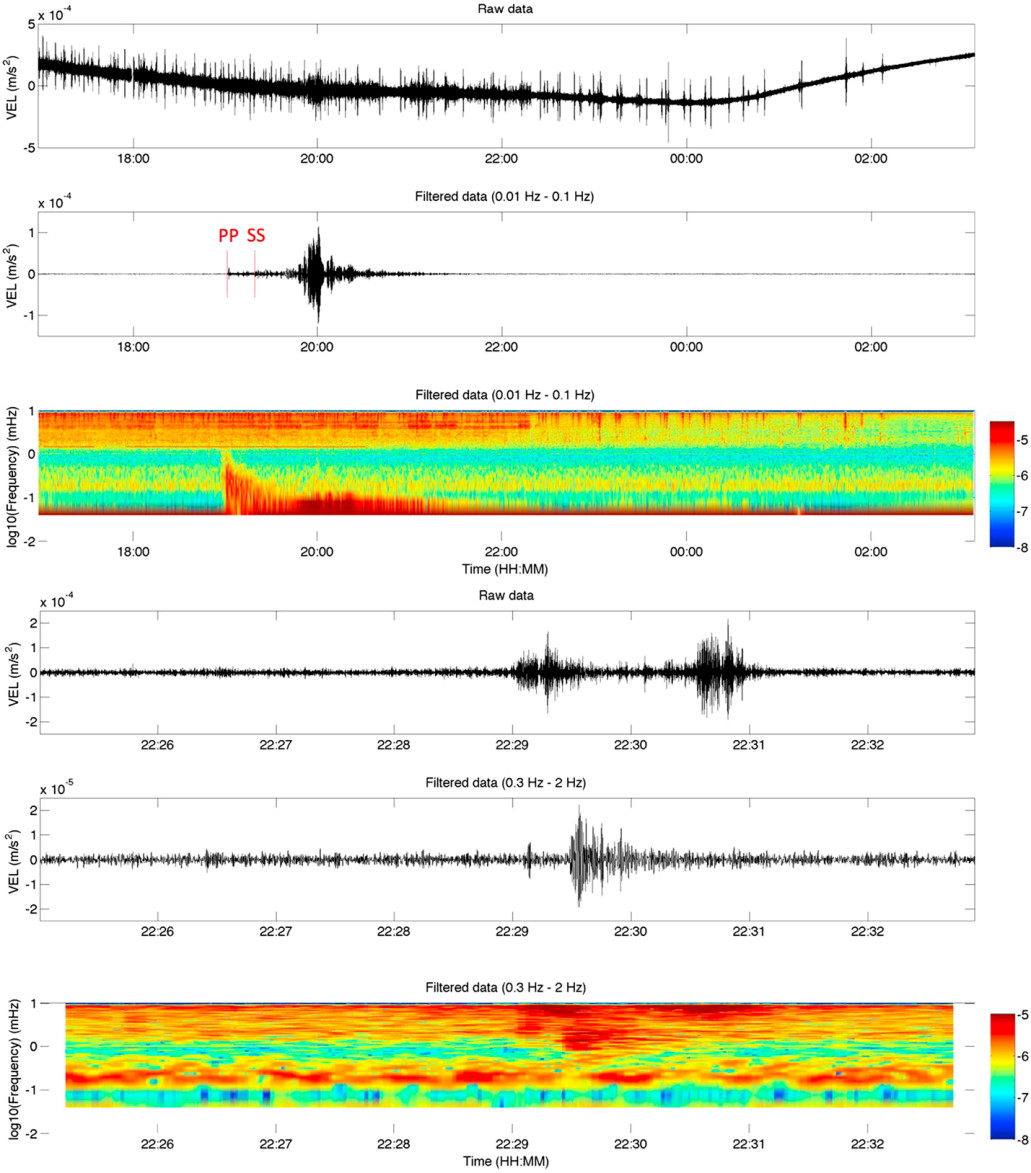
Records of earthquakes detected by VBB14 (**a**) and VBB12 (**b**) in low gain engineering mode during temperature tests when located at IPGP (48.808°N 2.492°E): raw data (top), filtered data (middle) and spectrogram (bottom). (**a**) $M_{w} = 7.8$ Solomon Islands earthquake occurred December 8, 2016, 17:38:46 UTC (epicenter location: 10.681°S 161.327°E, depth: 40 km, epicentral distance: 138.0°). The arrival times of PP and SS waves are indicated in red. P and S waves are not recorded at this epicentral distance. (**b**) $M_{w} = 3.9$ earthquake occurred near Brest (France) December 11, 2016, 21:27:23 UTC (epicenter location: 48.490°N 4.460°W, depth: 2 km, epicentral distance: 4.6 km). Noise levels are all related to the test facility, a site very far from seismic vault conditions

The mission schedule was compatible with only a few days of passive seismic monitoring at the different stages of the VBBs integration, both prior to their integrations in the sphere and after. Nevertheless, several earthquakes were observed by the Flight sensors during testing activities, including cold tests. Figure 14 shows two such earthquakes of $M_{w} = 7.8$ (a) and $M_{w} = 3.9$ (b) recorded by two VBBs located in the IPGP ‘Observatoire de Saint Maur’ facility. After filtering of the $M_{w} = 7.8$ earthquake, we clearly observe the surface wave packet with the highest amplitude at 20h00, as well as the PP and SS phases. A short period filter (0.3–2 Hz) reveals the $M_{w} = 3.9$ earthquake, which was hidden between two signals of suburban commuter trains.

**Fig. 15:**
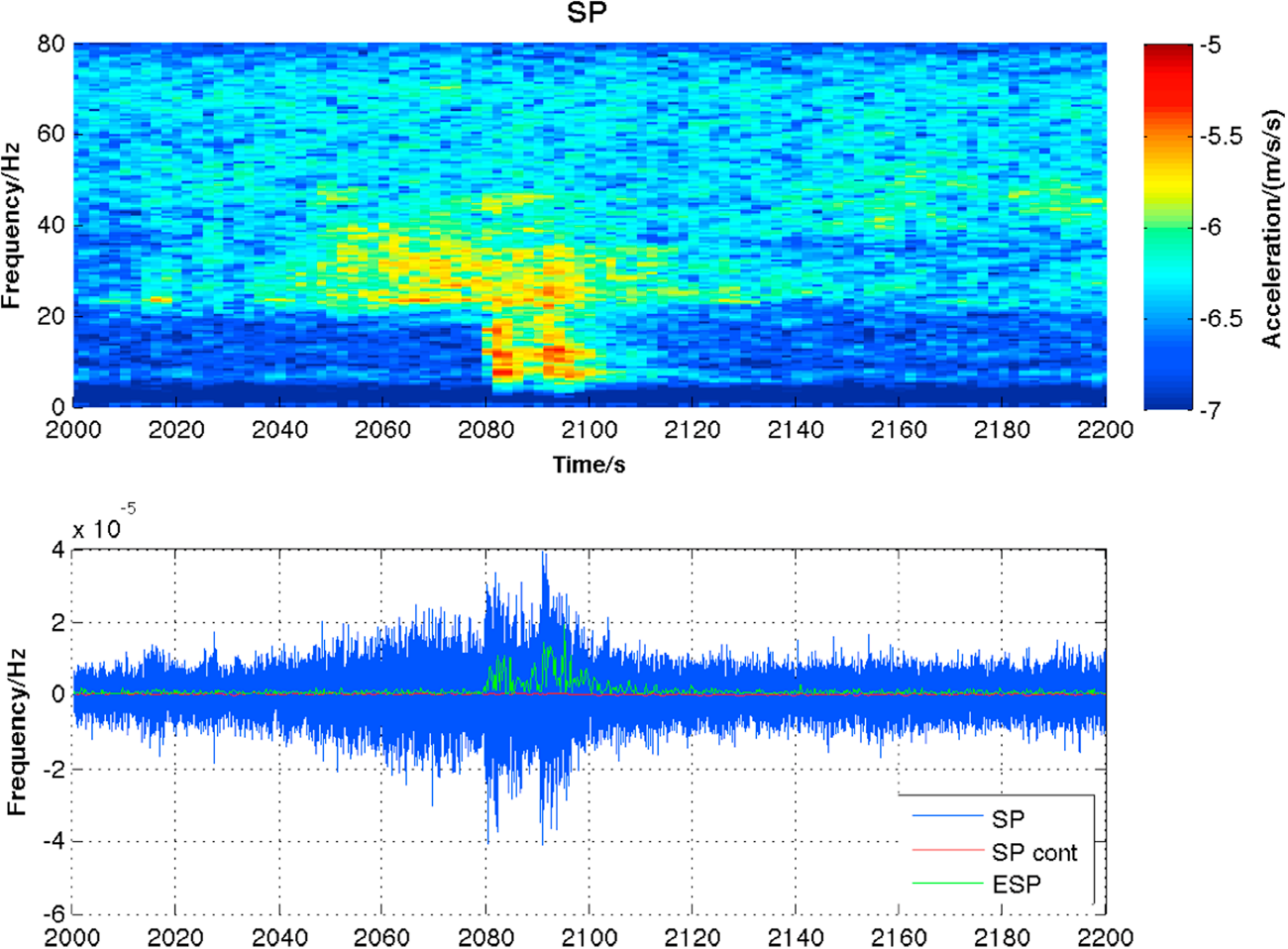
Record of local earthquake detected by QM SP1 in Acton, California: spectrogram (top), time series (bottom) for a $M_{w} = 1.4$ ENE of Colton, California, occurred on July, 17th, 2016, 06:34:11 UTC. The time series show the full 80 Hz bandwidth from 200 sps (labeled SP), downsampled to the continuous stream of 2 sps (cont. SP) and using the energy in a 4 to 16 Hz filter downsampled to 2 sps (ESP)

A non-flight (but similar) model vertical-axis SP was field tested at ambient temperature, inclined to match Mars gravity, over six days in the Kinemetrics test vault in Acton, Southern California. 12 events from $M_{w}$ 1.4 to 6.3 were recorded. Figure 15 shows a low magnitude local event expressed as a spectrogram and as three time series, at the full 80 Hz bandwidth from 200 sps (labeled SP), downsampled to the continuous stream of 2 sps (cont. SP) and using the energy in a 4 to 16 Hz filter downsampled to 2 sps (ESTA). While there is no recognizable event in the continuous data the ESTA time series (labelled ESP in the Fig. 15) correctly identifies the event, validating the approach adopted for InSight’s data downlink at least for local terrestrial events.

**Fig. 16:**
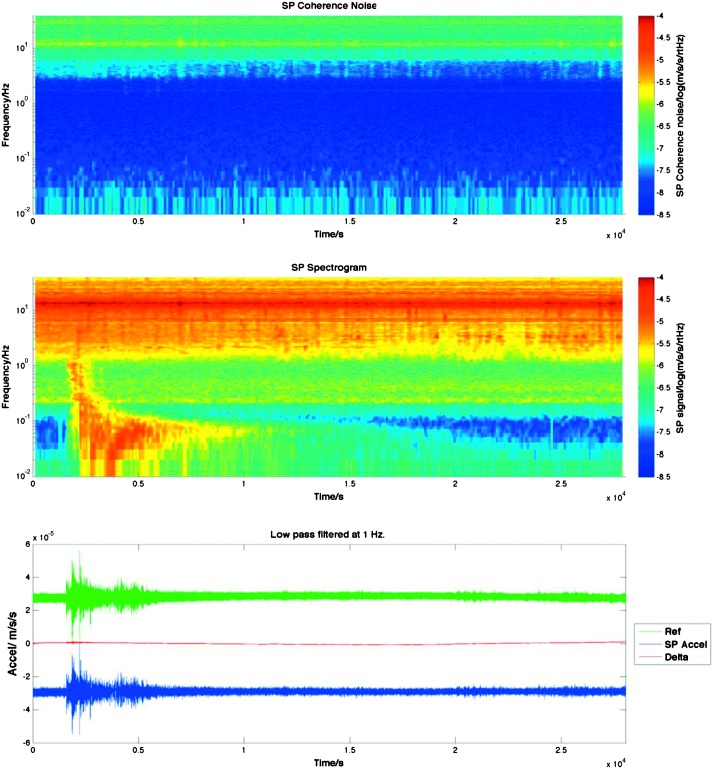
Record teleseismic earthquake detected by QM SP1 in Oxford UK: spectrogram of the incoherent noise with respect to the reference sensor, signal spectrogram (middle) and time series (bottom, for a reference seismometer, green, for the SP in blue with the difference in red) for a $M_{w} = 7.7$, $29~\mbox{km}$ SW of Agriha, Northern Mariana Islands at 2016-07-29 21:18:24 UTC. The red time signal is the one used for the coherence noise spectrogram

A larger, $M_{w} = 7.7$, teleseismic event is shown in Fig. 16. This was also detected during SP testing in Oxford, again at ambient temperature. The source was 29 km SW of Agriha, Northern Mariana Islands at 2016-07-29 21:18:24 UTC. The P and S wave are seen in both the reference and SP time series, with the R1 Rayleigh waves seen most clearly in the spectrogram climbing in frequency to 0.06 Hz. The derived SP sensor noise, which is the incoherent difference with the reference sensor, is stable over time, with no glitches, with a very good match to the reference in the time domain.

#### LVL and Tiltmeters

The SEIS Leveling System (LVL) has been developed by the Max Planck Institute for solar system research (MPS) and is detailed in Sect. 5.3. It has several purposes:

- provide the main structure of the SEIS sensors assembly (SA) and a “rigid” link to the ground,
- allow the precise leveling of the SA on slopes of up to 15° or on rocky ground,
- measure precisely the tilt angle,
- plus other functions, like supporting science temperature sensors, heaters and sensor thermal protection or performing active tilt calibration of the 6 axes on Mars.

The LVL consists of two main parts: a mechanical part, the leveling structure, as the central part of the Sensor Assembly and an electrical part, the Motor Driver Electronics (MDE), integrated in the EBox.

**Fig. 17:**
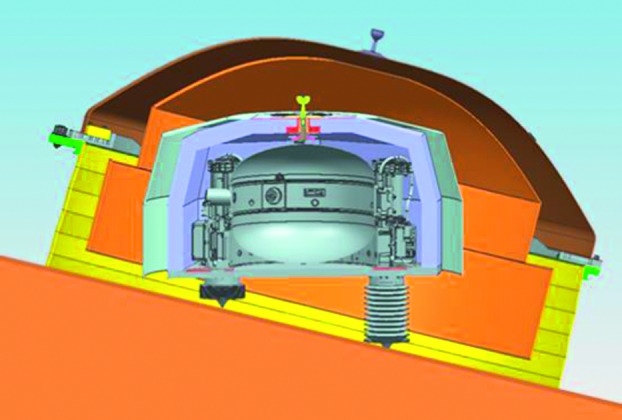
15 degrees tilted configuration for extreme deployment conditions. As a low rigidity regolith is expected at surface, SEIS will however be always in contact with the flat disks of the three feet, in the center of which a spike will penetrate in the ground. The surface will therefore be deformed just beneath each foot

The main part of the leveling structure is a structural ring. The following components are mounted on this structural ring:

- The three expandable legs: driven by stepper motors, those legs are able to compensate tilts of the SA of up to 15° (Fig. 17) and have a displacement resolution of roughly $0.6~\upmu\mbox{m}$. Their geometry has been optimized in order to maximize the stiffness and to minimize any backlash. At the bottom of the legs, there are cone-shaped feet with optimized geometry in order to provide a good interface with the Martian soil and to anchor SEIS against horizontal sliding generated by the tether’s thermoelastic deformations. See Fayon et al. (2018) for more details on the feet.
- The tiltmeters: two types of tiltmeters are integrated on the LVL’s ring. A two-axes MEMS sensor for coarse leveling (resolution better than 0.1°) and two single-axis High Precision (HP) tiltmeter for fine leveling (resolution better than 1 arcsec).
- The heaters: three heaters are serial mounted inside the ring in order to face the cold temperatures during winter time. They provide a heating power of 1.5 W.
- The Science Temperature sensors (SCIT). Two sensors are mounted on the ring.
- The spider structure: it is the mechanical link between the LVL’s ring and the grapple hook, which is the interface that will be grabbed by the deployment arm of the lander.
- The SPs (see Sect. 5.2).
- The VBBs proximity electronics (see Sect. 5.1.4).
- The interface with the cradle (see Sect. 5.7) and the VBB sphere.

**Fig. 18:**
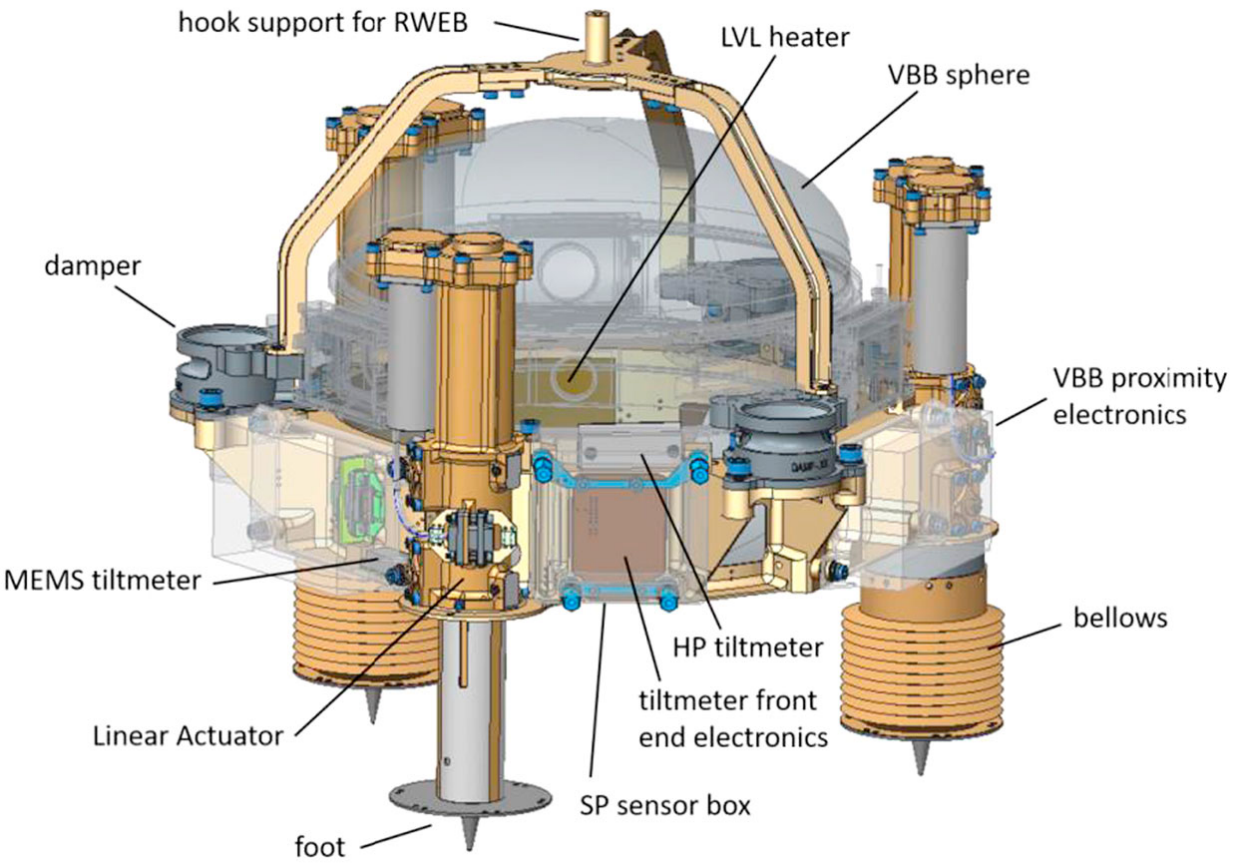
LVL design as well as location of all sensor assembly subsystems

Figure 18 provides the placement of all these subsystems in the sensor assembly. The MDE card controls the LVL from the EBox where it is integrated. It activates the stepper motors of the legs, as well as the heaters and acquires the signals from the tiltmeters. It also provides diagnostics and protection against motor overheating.

#### EBox

**Fig. 19:**
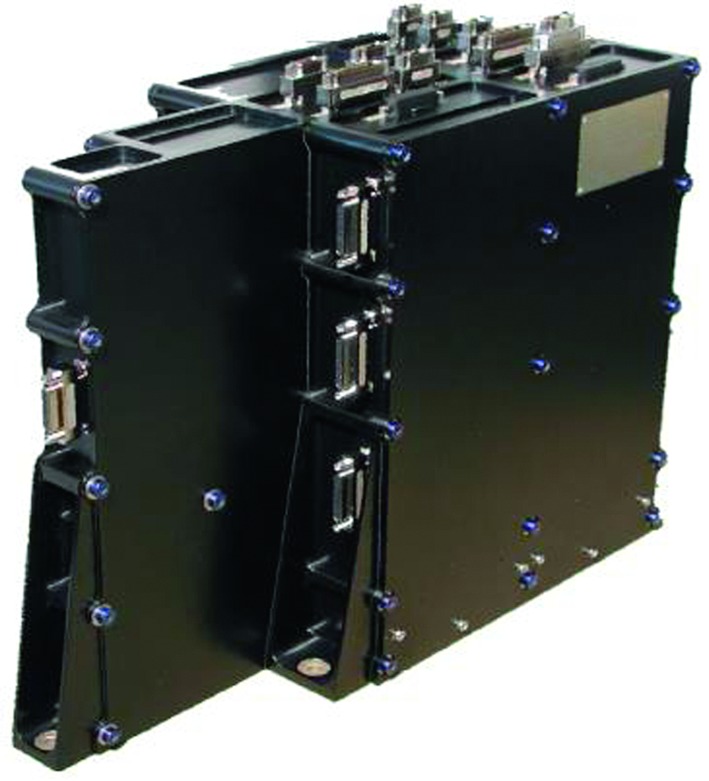
The SEIS EBox. A $\sim 5~\mbox{kg}$ and $\sim 9~\mbox{W}$ electronic box. The E-box is 243.8 mm in height. The top is $303.5~\mbox{mm}\times 125.5~\mbox{mm}$ while the bottom is $343.5~\mbox{mm} \times 169.5~\mbox{mm}$ due to mounting structures

The Electronic Box (EBox, Fig. 19) of SEIS has been developed by ETH Zurich (ETHZ) with the exceptions of the VBBs and SPs sensor feedback cards and the LVL-MDE control card, which are integrated inside the Ebox. See Sect. 5.4 for more details on the power and acquisition parts of EBox and Sects. 5.1, 5.2 and 5.3 for those related to the VBBs, SPs and LVL-MDE cards. Ebox contains the main part of SEIS’ electronics and is located inside the lander’s Warm Electronic Box. Thus, it is not submitted to the same environmental constraints as the SA: temperature will remain within the MIL temperature range, but is nevertheless not stable, with significant changes occurring when the lander operates. Of course, the Ebox stays in the same location while the SA is deployed on the ground.

**Fig. 20:**
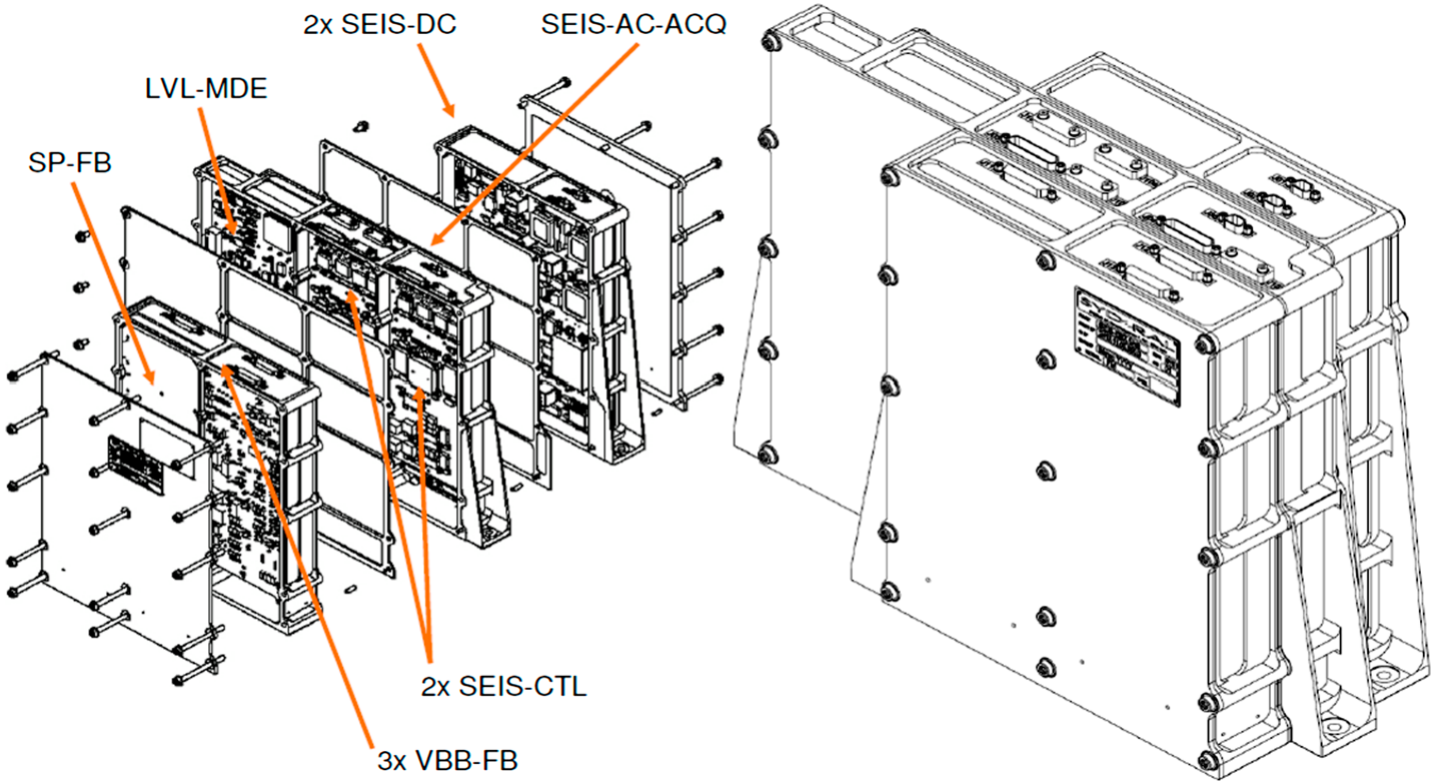
The EBox and its electronic boards. The E-box is 243.8 mm in height. The top is $303.5~\mbox{mm} \times 125.5~\mbox{mm}$ while the bottom is $343.5~\mbox{mm} \times 169.5~\mbox{mm}$ due to mounting structures

Figure 20 shows the electronic boards integrated in the EBox:

- 3 VBB-FB (Feedback), delivered by IPGP for the VBBs,
- 1 SP-FB, delivered by Oxford University for the SP,
- 1 LVL-MDE, delivered by MPS for the LVL,
- 1 SEIS-AC (including 1 ACQuisition and 2 ConTroL boards for redundancy) from ETHZ,
- 2 SEIS-DC modules from ETHZ modules which receive the 28 V primary power line and provide all secondary voltage lines to others sub-systems.

As the electrical interface of SEIS with the lander, it is controlled by the lander’s Command and data Handling (C&DH) and powered by the Power Distribution and Drive Unit (PDDU). When the lander is in sleeping mode, the EBox provides operating power to the sensor units with stabilized voltage. In addition, the SEIS-AC main controller board is in charge of the acquisition of the scientific signals. This board digitizes analog signals and can store up to 65 hours of data. Data are transferred to the lander during its wake-up period and then processed and transmitted to Earth.

#### Tether and LSA

**Fig. 21:**
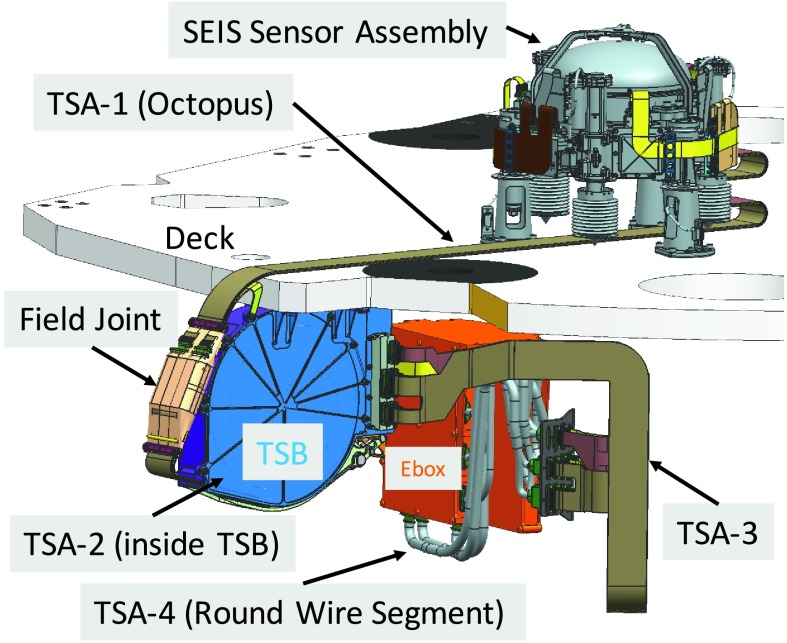
Tether System overview, Stowed Configuration. The Ebox is inside the Thermal Enclosure of the Lander. The Thermal Enclosure is not shown. The height of the Sensor Assembly on the InSight deck is about 33 mm and the distance from the center of the Sensor Assembly to the field joint is about the same

Once on the ground, the SEIS seismometer will remain connected to the InSight lander through a sophisticated umbilical tether in the form of a semi-rigid flat cable, called the Tether. See Sect. 5.5.2 for a more detailed description and Fig. 21 for a general description.

The tether has the following main functions

- Provide an electrical link between the EBox and the SA. This is performed thanks to 4 sub-parts (TSA-1 to 4) connected together.
- Allow for the deployment of the SA on the ground. This is performed in particular thanks to the TSB (Tether Storage Box) that contains the TSA-2 part and releases it just before deployment.
- Decouple the SA from the mechanical noise that could come from the lander. This is performed thanks to the LSA (Load Shunt Assembly) which is an extra loop of the TSA-1 that is released by a frangibolt (Shape Memory Alloy launch lock device).

#### RWEB and WTS

**Fig. 22:**
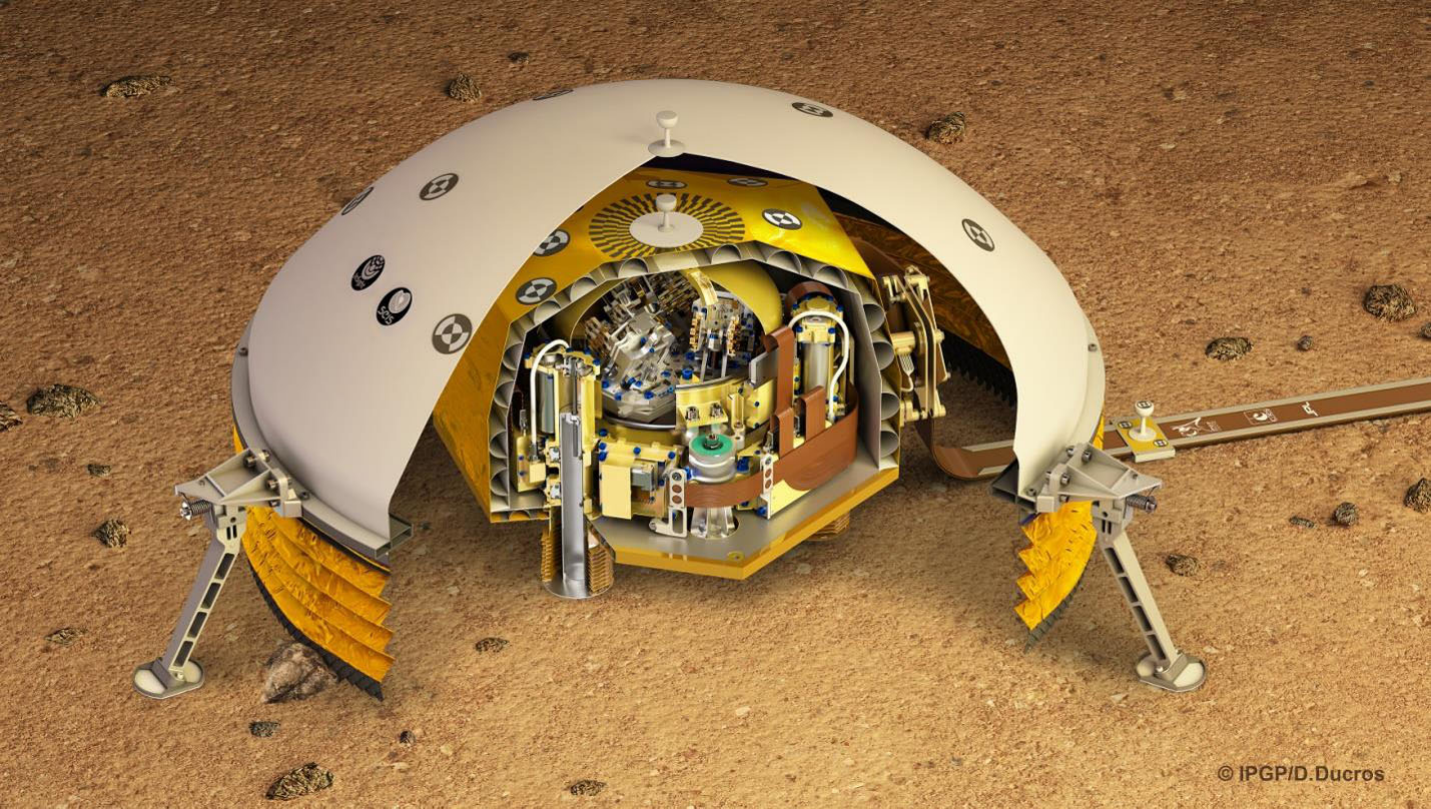
Illustration of the RWEB and WTS configuration after deployment

The RWEB and WTS are the “portable” seismic vault of the instrument and are targeted to provide a strong thermal, wind and sun protection to the instrument, in addition to maintain the ground near SEIS in permanent shadow and to reduce tilts effects associated with ground temperature changes. See Sect. 5.6 for a more detailed description. The Fig. 22 gives an idea of the several barriers in between the seismic sensors and the Martian environment.

The first barrier (RWEB or Remote Warm Enclosure Box) is made of titanium and mylar and uses the Martian atmosphere as an insulator. Thanks to its reduced gaps, it prevents convection from developing. It is part of the Sensor Assembly. The second barrier (WTS or Wind and Thermal Shield) provides an extra protection against the winds and the thermal variations. 3 legs support a dome from which a skirt is hanging. The skirt is able to adapt to the terrain in order to provide a maximum protection.

#### Cradle

**Fig. 23:**
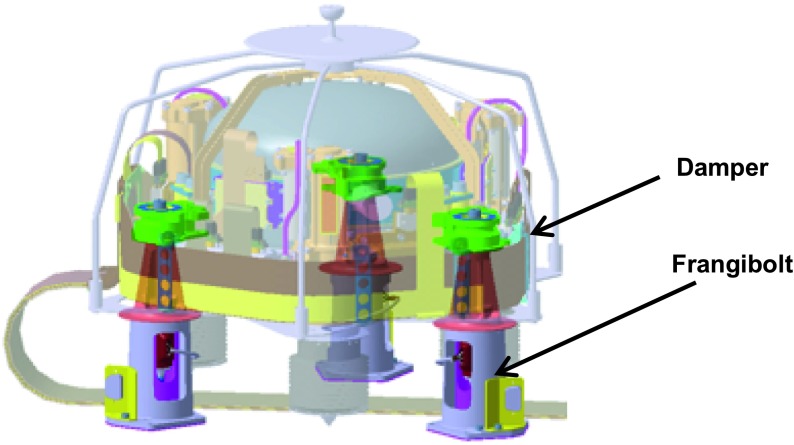
Cradle subsystem overview

The Cradle subsystem (see Sect. 5.7 for a more detailed description) is made of three nearly identical turrets at 120° around the SEIS Sensor Assembly and provides two main functions:

- It connects the SA to the spacecraft until it is deployed on Mars. In particular, each turret is fitted with an elastomer damper that limits the mechanical loads seen by the SA.
- It allows the separation of the SA from the spacecraft in order to start the deployment. To do so, each turret is fitted with an off-the-shelf frangibolt (shape memory alloy launch lock device) that breaks a titanium fastener when heated. Each frangibolt has a nominal and redundant heater circuits that are connected to the unregulated load switches of the spacecraft. See Fig. 23 for their location.

#### Instrument Architecture and Integration Process

**Fig. 24:**
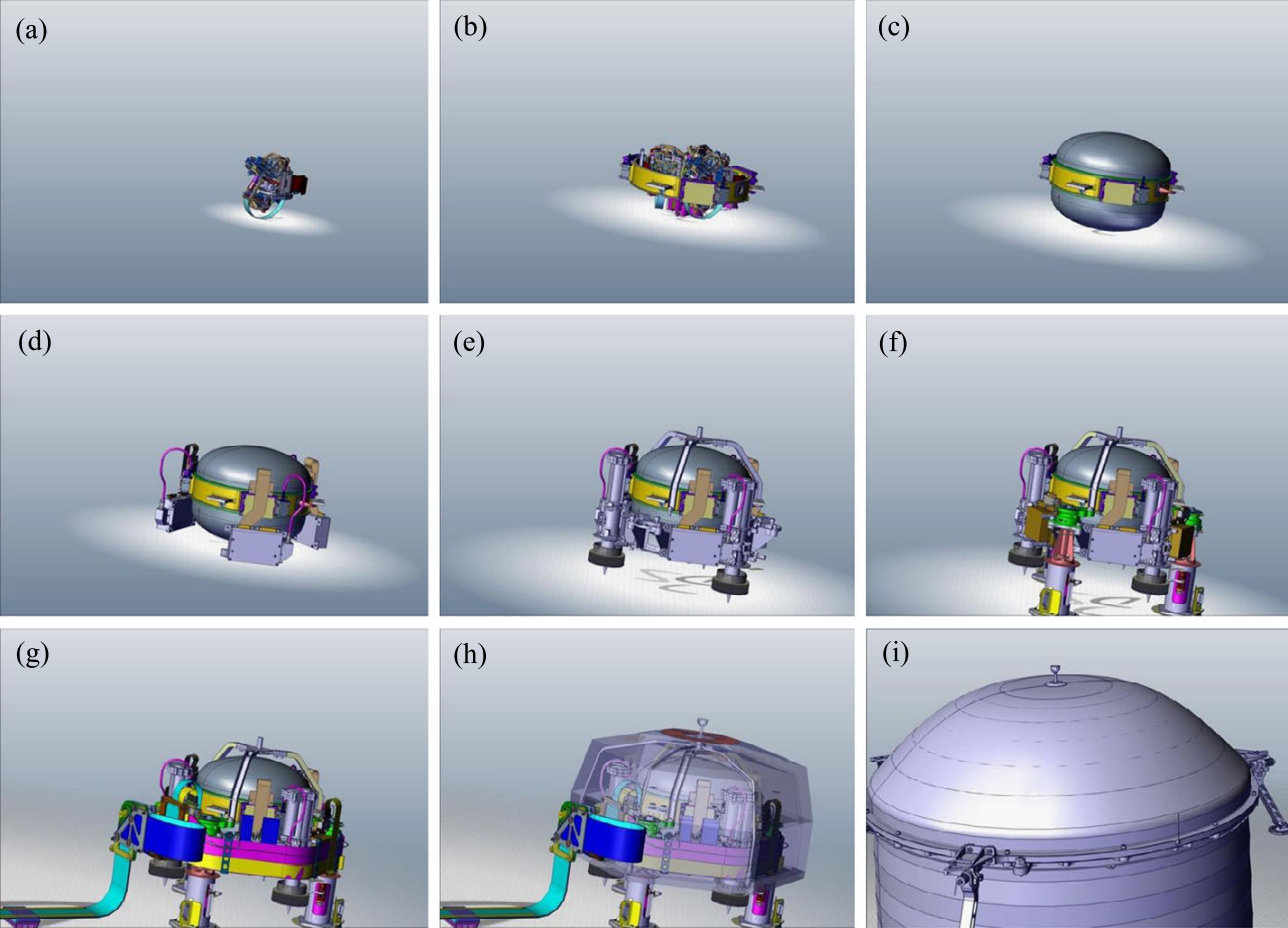
Sensor Assembly integration summary. Each VBB (**a**) is integrated in the crown (**b**). (**c**) Shells are welded on the crown, the vacuum is made and the exhaust tube (queusot) is pinched off. (**d**) VBBs can then be tested when connected to their PE (Proximity Electronics), one PE for each VBB. (**e**) The sphere and then all SPs (Short Period) are added to the LVL ring. (**f**) The cradle makes the mechanical link between the instrument and the lander. (**g**) The tether makes the electrical link between all the Sensor Assembly’s components and the Lander. (**h**) The RWEB provides a first protection, mainly thermal. (**i**) The WTS (Wind and Thermal Shield) is placed on the Sensor Assembly after the SA is deployed

The storyboard shown on Fig. 24 gives a better idea of the sensor assembly organization, even if it is not fully representative of the order in which the components are integrated. The VBB sensors heads are integrated first one after the other in the sphere crown. Each VBB was therefore compatible for either the Flight model or the Spare model, enabling a selection process for the three best VBBs for the Flight model. See movie in supplementary material 2 for the last VBB mounting on the crown. Shells are welded on the crown. The sphere is evacuated and outgassed during a 2 week bakeout process and the exhaust tube (queusot) is pinched off. After thermal and functional tests of the sphere tests, performed at IPGP for all VBBs with their proximity electronics and generic feedback cards, the sphere has been delivered to CNES Toulouse for further integration. The Sphere and VBB proximity electronics are then integrated on the MPS delivered Leveling System (LVL), like the SPs delivered by Imperial College. After connecting of the VBBs, SPs and LVL tether, the RWEB is finally placed to close the sensor assembly.

#### Instrument Budgets

##### Power Budget

The SEIS instrument is powered by the non-regulated primary 28 V bus of the InSight space craft, directly connected to the lander batteries which will be recharged by the solar panels’ generator during day time.

The power of the SEIS Flight Model instrument has been measured during Assembly, Integration and Test (AIT) in a standalone test where the SEIS instrument was powered by a commercial power supply. Additional tests and measurements have been done when the SEIS instrument was connected to the Flight Model lander and powered by the lander power supply subsystem. The Ebox has a power supply subsystem, taking power from primary lines of the lander and powering the Ebox internal cards (3 VBB feedback boards (VBB-FB)), SP feedback board (SP-FB), LVL Motor Driver Electronics card (LVL-MDE) and its internal data acquisition and processing board (SEIS-AC). In normal operating mode, the power of the overall experiment is about 5.9 Watt. All these powers are measured at the non-regulated primary voltage level of the experiment and include losses in the DC/DC converter. They are provided in Table 5 for the different modes of the experiment.

**Table 5:** Power of the different modes of SEIS, at primary 28 Volt level

Mode	Standby	Startup	Nominal	Winter	Re-centering	Leveling	Active cross calibration
Power (mW)	2890	5650	5880	7960	8030	7930	11830

##### Mass Budget

SEIS is carrying to Mars not only the sensor’s heads and their electronics, but also all the installation and multilayer environmental protections necessary to fulfill the mission goals in terms of performance. The full mass of the instrument is therefore large at about 28.8 kg, with about 17.1 kg associated either with the instrument mounting on the lander (1.67 kg for the cradle), the windshield (almost 7.3 kg plus 2.2 kg of launch lock assembly) or the tether and tether box associated with the remote installation (5.9 kg). The remaining 11.7 kg are associated with the sensor assembly (6.5 kg) and the Ebox (5.2 kg). All mass breakdown details are listed in Table 6 and correspond to the weighed mass of the Flight units.

**Table 6:**
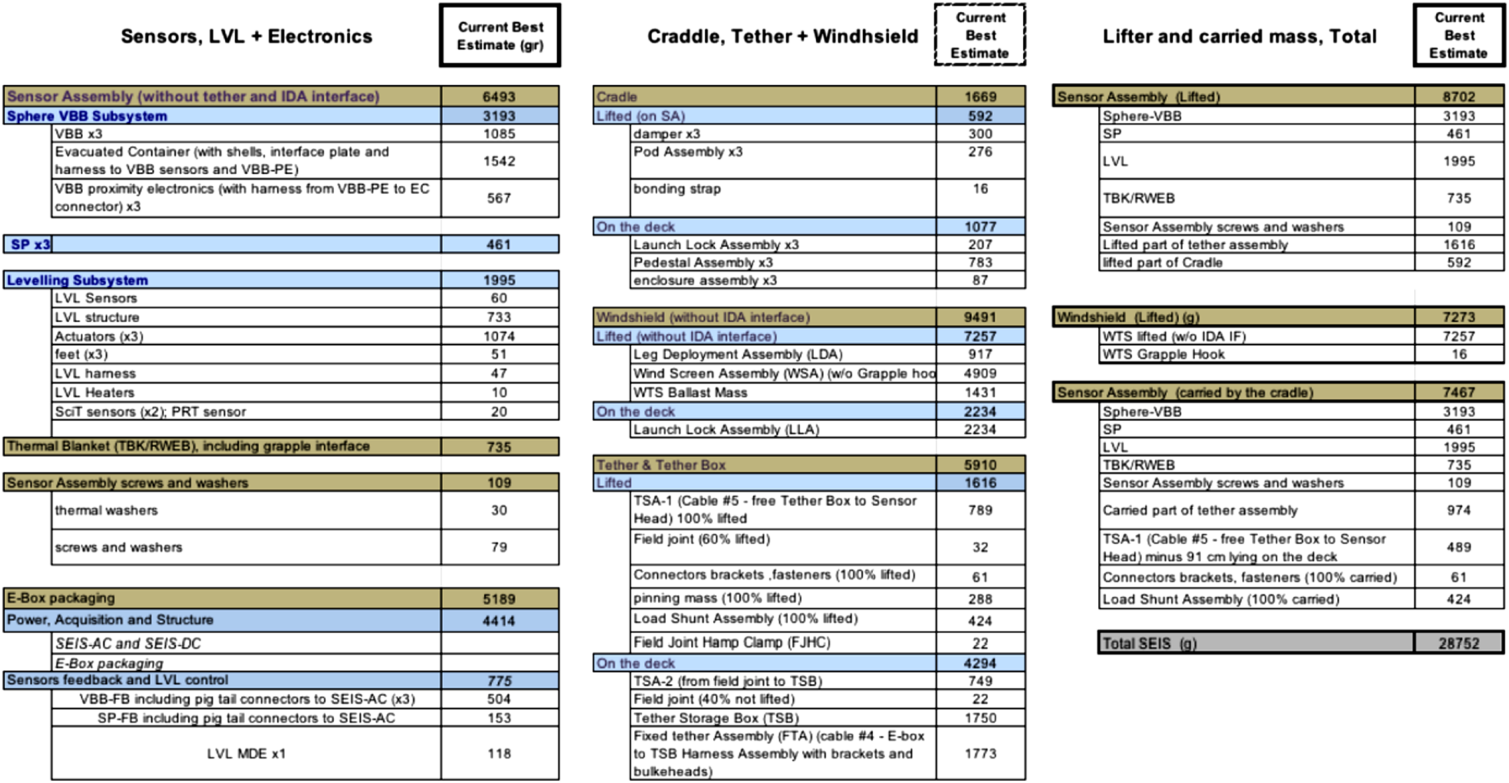
Mass breakdown of the SEIS experiment. The detail of the mass is provided for the Sensor assembly, Electronic box, tether system and Wind Shield. In addition, the mass either lifted by the robotic arm, or carried by the cradles are provided

With the exclusion of the tethers, the 3 SPs sensors encapsulated in their boxes for the sensors and with their feedback card have a mass of 614 g, while the 3 VBBs sensors encapsulated in the sphere for the sensor and with their 3 feedback cards have a mass of 3697 g, about 6 times larger. This is the ratio between the measured performances of the SPs and VBBs in low noise seismic vault condition at 2.5 s ($\sim 3\times 10^{-9}~\mbox{m}/\mbox{s}^{2}/\mbox{Hz}^{1/2}$ for the SPs and $5\times 10^{-10}~\mbox{m}/\mbox{s}^{2}/\mbox{Hz}^{1/2}$ for the VBBs) and illustrates that both sensors fit well along the optimum slope of −1 between performances and mass, as defined by Pike et al. (2016). The mass of the VBB sphere, LVL and VBB feedback cards is about 5.7 kg and can be compared to Earth instruments with built-in feedback but manual LVL like the Trillium compact 120 seismometer (1.2 kg and $-174~\mbox{dB}$ with respect to $1~\mbox{m}/\mbox{s}^{2}$ and about $2\times 10^{-9}~\mbox{m}/\mbox{s}^{2}/\mbox{Hz}^{1/2}$ at 2.5 s, Nanometrics 2018) or the Streckeisen STS-2.5 (12 kg and $-194~\mbox{dB}$ with respect to $1~\mbox{m}/\mbox{s}^{2}$ and about $2\times 10^{-10}~\mbox{m}/\mbox{s}^{2}/\mbox{Hz}^{1/2}$ at 2.5 s, Kinemetrics 2017). The ratio of performances and mass with respect to the VBB/LVL/FB are about 0.85 for both the TC120 and STS-2.5 and therefore close to 1 despite the VBB capability to perform motorized leveling and its space qualified status, including the very efficient evacuated sphere with its high thermal protection efficiency.

If both the SP and VBB sensors are close to the optimum mass, future optimization, if needed for future missions, can nevertheless be made, especially with respect to the tether which is a very complex piece of hardware. SEIS was indeed limited by the capability of the deployment arm, which impacted the maximum lifted mass of the Sensor Assembly and by the very cold temperature encountered by the Sensor Assembly when powered off ($-115^{\circ}\mbox{C}$). This prevented the integration of all feedback cards in the sensor assembly, including the quartz oscillator required for the displacement transducers. If future opportunities allow larger lifted mass, significant optimization will be possible. For Mars missions, other optimization can be made by improving the aerodynamic shape of the WTS (Nishikawa et al. 2014) and/or reducing the maximum wind over which the WTS is authorized to move. This was not performed for InSight and, for static friction larger than 0.2, the mass of the WTS is compatible with a non-displacement of the WTS up to $80~\mbox{m}/\mbox{s}$ of wind, providing therefore a high safety margin, even if a large dust devil is passing over the WTS.

##### Data Budget

The data handling and transmission strategy of the experiment has been designed in order to ensure that seismic data and the APSS data (pressure, wind and magnetometer) are registered continuously during the monitoring mode at the highest data rate and stored in the lander mass memory. This approach is dictated by the need of the full waveform for seismic signals and of a full environmental monitoring for either decorrelation or just confirming that a seismic signal is not related to weather activity. With the sampling rates listed in Table 7, this leads to about $1.03~\mbox{Gbits}/\mbox{sol}$, excluding overheads and secondary data from the SEIS flight software. This is too much for a full transmission.

**Table 7:**
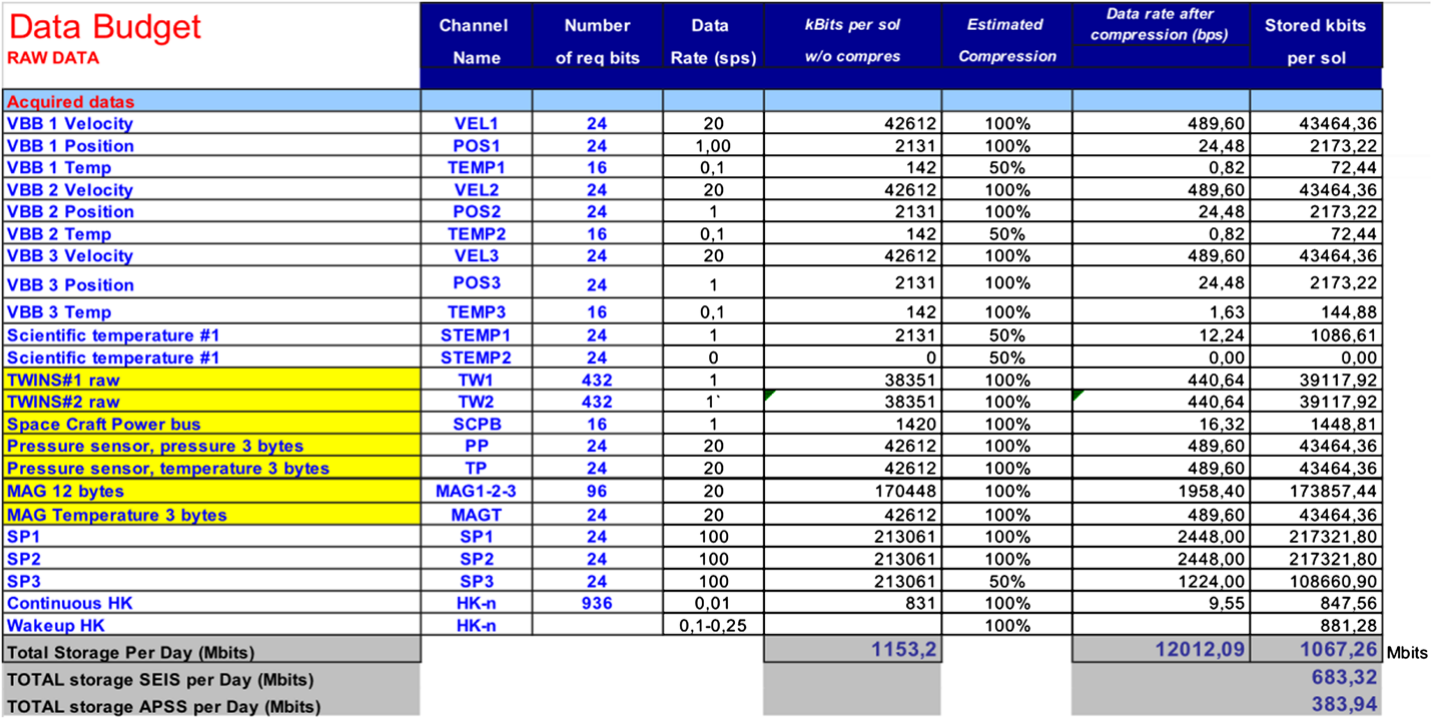
Raw data production of SEIS during one sol, before compression. A minimum of 50% of compression is expected and low noise conditions are expected to reach compression ratio of 40%

The data transmission strategy has therefore been based on (i) a first lossless compression, (ii) the dump of all short period data in a large mass memory, (iii) the transmission of all low pass filtered and decimated signals needed to perform the science goals below 1 Hz and (iv) a post-selection process, where events of interest in the high frequency bandwidth ($> 1~\mbox{Hz}$ for SEIS) will be transmitted as events, with a sampling rate larger than the continuous data, but which nevertheless can be less than the acquired frequency (100 sps for SPs, 20 sps for VBBs, Pressure, IFGs and 1 sps for wind) through a tunable decimation by FIRs in the Flight Software. See Sect. 7.2 for more details on the Flight Software.

All data have been compressed with a lossless STEIM compression (Steim 1994), in which the delta value between two consecutive samples is compressed. This value can also be expressed as

1 $$ d(n) - d ( n -1 ) = \Delta t \frac{d ( n ) - d ( n -1 )}{\Delta t} \approx \frac{ \Delta t}{\mbox{LSB}} \gamma , $$

where $d(n)$ is the velocity flat output signal in count, $\gamma $ is the acceleration, $\Delta t$ the sampling interval in second and LSB the velocity flat output LSB in $\mbox{m}/\mbox{s}$. At their primary sampling rate and in high gain mode, both VBBs and SPs are therefore expected to generate about 9–10 bits per sample due to their self-noise after Steim compression and with overhead. The compression ratio of the raw data has therefore been assumed as 50% with 2 bits of margins. But it is likely, especially for the continuous data at 2 sps during night, that better compression will be achieved. All SEIS data then generate less than $400~\mbox{Mbits}/\mbox{sol}$ of data and less than $550~\mbox{Mbits}/\mbox{sol}$ when the APSS data at high rate (Pressure and MAG at 20 sps and TWINS at 1 sps) are also included.

**Fig. 25:**
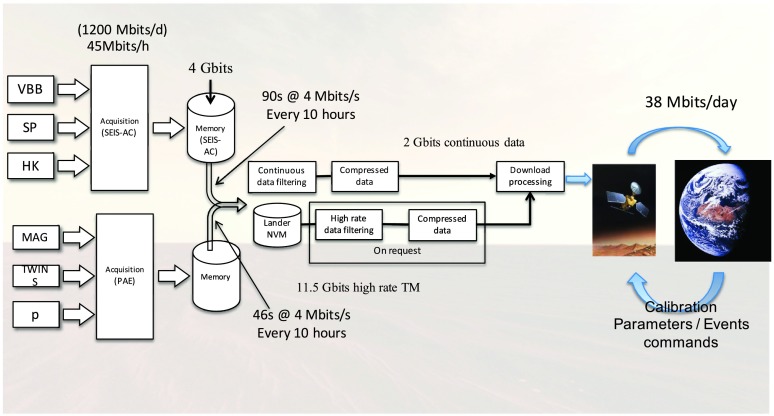
Transmission strategy of the SEIS experiment

Further data compression was therefore mandatory in order to fit the data into the SEIS allocation of $38~\mbox{Mbits}/\mbox{sol}$, including all APSS data requested for seismic analysis and the flight software data. The chosen strategy, illustrated in Fig. 25, is based on the transmission of both low frequency continuous channels and of selected-event high frequency channels.

All data are therefore first acquired at high frequency by the SEIS Acquisition system, with a baseline of 20 sps and 100 sps for the 3 VBBs and 3 SPs respectively and stored in the flash memory inside the Ebox.

##### Continuous Data

About every 10 hours, the SEIS FSW retrieves these data. As the data provided by the SEIS instrument are much larger than the InSight system can accommodate, several pieces of software have been implemented to filter and decimate the input raw data onboard (see Sect. 7.2 on SEIS FSW).

Those data, at full resolution/frequency are first stored in a Full Rate Buffer inside the lander flash memory. They are then filtered and decimated to produce a continuous data flow which is then sent through telemetry to orbiters around Mars, then through the JPL Deep Space Network to Earth.

This sampling allows to fully cover the DC-1 Hz bandwidth for the VBBs and pressure, with VEL and pressure data sampled at 2 sps plus magnetic data sampled at 0.2 Hz as magnetic noise is expected to be possibly significant above 0.1 Hz. In addition, POS is decimated by two with an additional numerical gain of 4, to generate a 0.5 sps time series and additional wind data, at 0.1 sps are transferred in order to discriminate aeolian signals from seismic events.

Some high-frequency data are in addition partially transmitted continuously, with a VELZ output at 10 sps and an ESTA output at 1 sps. VELZ will be a composite output of the 6 channels, defined as

2 $$\begin{aligned} \mbox{VELZ} =& \mbox{FIRVBB}*(\alpha_{\mathrm{vbb}}\mbox{VBB1} + \beta_{\mathrm{vbb}}\mbox{VBB2} + \gamma_{\mathrm{vbb}}\mbox{VBB3}) \\ &{}+ \mbox{FIRSP}*(\alpha_{\mathrm{sp}}\mbox{SP1} + \beta_{\mathrm{sp}} \mbox{SP2} + \gamma_{\mathrm{sp}}\mbox{SP3}), \end{aligned}$$

where FIRVBB and FIRSP are decimating FIRs, performing an equalization of the outputs with respect to their noise and gain, an anti-alias low-pass and a final decimation from their raw sampling rate to 10 Hz. Such filters can for example be used to generate a hybrid output, in a way similar to the one used by Kawamura et al. (2017) for Apollo LP and SP data. The ESTA will be the rms of a band pass filtered data, as defined by

3 $$\begin{aligned} \mbox{ESTASP} = \mbox{rms} \bigl(\mbox{FIRSP}_{\mathrm{esta}}*( \alpha_{\mathrm{sp}}\mbox{SP1} + \beta_{\mathrm{sp}}\mbox{SP2}+ \gamma_{\mathrm{sp}}\mbox{SP3}) \bigr), \end{aligned}$$

for the example of the SP channels. It will capture, typically every second, the high frequency energy in the bandwidth defined by the FIRSP_esta_. Similar ESTA processing is planned for the IGF magnetic data and for the Pressure data, in the latter case with the possibility to implement two types of ESTA channels, one for weather events (e.g. dust devils) and the other for infrasound events.

Together with Housekeeping monitoring (every 100 seconds except during wake-up at higher rate) and with clock synchronization data for the relative drift between APSS and SEIS, a total of about 30 Mbits per sol, corresponding to the SEIS allocation for continuous data are then transmitted. From this allocation, 78% are corresponding to continuous SEIS data and 22% to continuous APSS data and includes 2% of data headers. See the continuous data budget detail for all channels in Table 8.

**Table 8:**
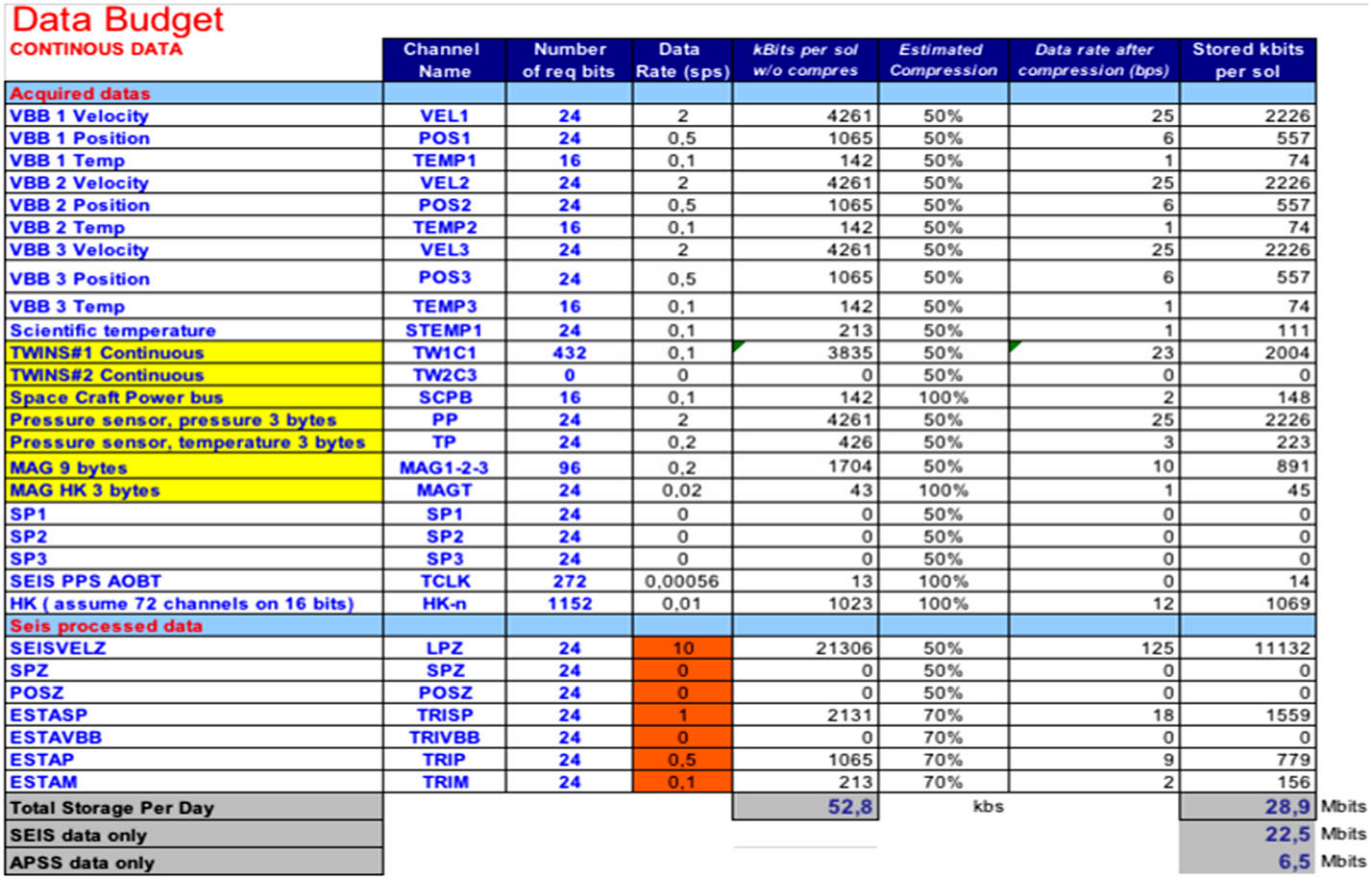
Continuous data budget breakdown. About 30 Mbits of continuous data will be transmitted every sol and will provide all seismic information for a full monitoring of the DC-1 Hz bandwidth

##### Events Data

Continuous data will be distributed regularly to both the Science Team and the Mars Quake Service (see Sect. 8.2) with a latency of less than 2 hours after being received on Earth. If the detection of a seismic event is suspected, the ground segment can send a request to the lander to retrieve buffer data with full sample rate from the lander. Those event request can be for data filtered and decimated from the full rate. However, the full rate data can also be downloaded. 8 additional Mbits have been baselined for the transmission of these events during the nominal monitoring phase. It is planned that transmitted seismic events will be systematically supplemented by high frequency wind and pressure data, which will also be transmitted as event for the same period of time.

##### Data Transmission Update

The previous sections describe only the data transmission for the continuous monitoring phase which is summarized in Fig. 25. Possibly, the compression ratio will be much better for low noise conditions, enabling possible increase of the output sampling rates of the continuous channels or the transmission of new channels, including continuous SP channels.

During the early phase of the mission, including commissioning, different transmission scenarios have been defined, either with increased frequency continuous data or with event requests associated with calibrations, motor activations, etc. For all the operational scenarios several FSW configuration files have been defined, for example for the cruise phase, for the period when the SEIS instrument is still on the deck of the lander after landing, for the commissioning phase and of course for the routine phase, called Surface Monitoring phase and described above in detail.

### SEIS Deployment

**Fig. 26:**
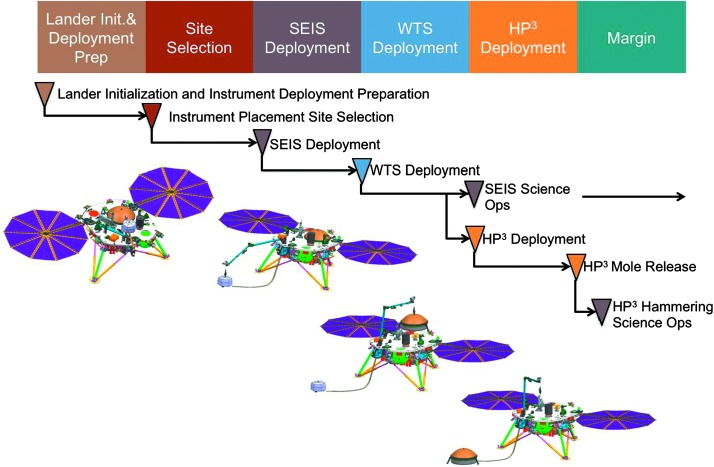
Deployment process of the SEIS experiment following the landing and prior to the HP3. This does not detail the deployment internal to the SEIS sensor assembly after Sensor Assembly deployment on the ground

The deployment of SEIS, illustrated on Fig. 26, is completed once the seismometer is placed on the surface of Mars, is leveled and centered, the service loop is released and the seismometer is covered by the free-standing Wind and Thermal Shield (WTS). The following is a detailed description of the carefully orchestrated deployment and verification steps that take place from the point where a surface deployment site was selected by the InSight Site Selection Working Group (ISSWG) to a fully deployed SEIS system as defined above. Each deployment step is verified on Earth by a set of specific measurements, images and other data, that help determine that its requirements are met and it is safe to proceed to the next deployment step. Several deployment steps are known as “committal events” or events that are not reversible. We elaborate on those as we follow the step-by-step SEIS deployment procedure below.

#### Site Selection

For SEIS to operate on the surface properly a number of deployment requirements must be met. The SEIS requirements as well as some desired characteristics are summarized in Table 9. SEIS has a leveling system that can accommodate up to 15° of tilt so both SEIS and the WTS must be deployed on surfaces with slopes of $<15^{\circ}$. This is nevertheless reduced to $-13^{\circ}$ for tilts lowering the height of the LSA, as the later cannot be deployed successfully for tilts ranging from $-15^{\circ}$ to $-13^{\circ}$. Both the SEIS leveling system and HP^3^ have clearances of $\sim 3~\mbox{cm}$ and so must be placed on surfaces with no rocks or protrusions higher than 3 cm. In addition, the SEIS leveling system can accommodate rocks or protrusions $<2~\mbox{cm}$ and $<1~\mbox{cm}$ high for instrument tilts of $11\mbox{--}13^{\circ}$ and $13\mbox{--}15^{\circ}$, respectively. For stability, foot patch roughness or relief of both instruments must be less than 1.5 cm and for $\mbox{WTS} <3~\mbox{cm}$. The soil beneath both instruments and WTS must be load bearing, as unequal sinkage could lead to additional tilt. This requirement will be integrated in the final site selection of SEIS, after assessment of the soil geomorphology and properties from picture analysis. After deployment, SEIS and WTS must not touch (for noise reasons), so the SEIS foot plane (the plane formed by the SEIS feet) must be less than 1.5 cm higher than the WTS foot plane and the relative tilt between the two must be less than 5°. There are also constraints on the location of the SEIS tether pinning mass (to mechanically isolate SEIS from the tether) and the tether field joint (the connection between the two SEIS tether sections, one from the lander and one from the instrument). The pinning mass and field joint must be free of rocks or other obstructions and on a gentle slope so that if the pinning mass needs to be moved, there will not be obstacles or a tilt hindering the movement.

**Table 9:** SEIS and WTS deployment site requirements

**SEIS & WTS deployment site requirements**
**Requirements**
*Tilt*
SEIS<15^∘^ Tilt (must also be <12^∘^ of negative pitch)
SEIS Footplane<12^∘^ for negative pitch slopes
WTS<15^∘^ Tilt
*Terrain*
No Rocks Under $\mbox{SEIS} > 3~\mbox{cm}$ high for tilts ≤ 11^∘^
No Rocks Under $\mbox{SEIS} > 2~\mbox{cm}$ high for 11^∘^<tilt ≤ 13^∘^
No Rocks Under $\mbox{SEIS} \geq 1~\mbox{cm}$ high for 13^∘^<tilt ≤ 15^∘^
No Rocks under $\mbox{WTS} > 6~\mbox{cm}$ high or $>3~\mbox{cm}$ low under skirt
No Rocks under Load Shunt Assembly
SEIS Footpatch Roughness: $<1.5~\mbox{cm}$
WTS Footpatch Roughness: $<3~\mbox{cm}$
Load Bearing Soil
*SEIS/WTS Relative Placement*
$\mbox{SEIS footplane} < 1.5~\mbox{cm}$ higher than center of WTS footplane
Less than 5^∘^ relative tilt between SEIS/WTS
SEIS not to exceed WTS DNE envelope
**Desired characteristics**
*SEIS*
SEIS Footplane Tilt<11^∘^
All three SEIS feet on same material
SEIS on terrain with positive pitch (uphill from lander along tether)
*Tether*
Place SEIS on the right side of workspace to avoid tether crossing
No rocks under pinning mass or field joint
Field Joint not in hole, in front of hole, or in front of rock
Pinning mass orientation desirable for adjustment with scoop
No obstacles around Pinning Mass
Plane of tether lower than plane of SEIS sensor assembly
*Noise—wind and other noise sources*
SEIS as far as possible away from the lander
$\mbox{SEIS} >= 1~\mbox{m}$ (as far as possible) away from HP3

**Fig. 27:**
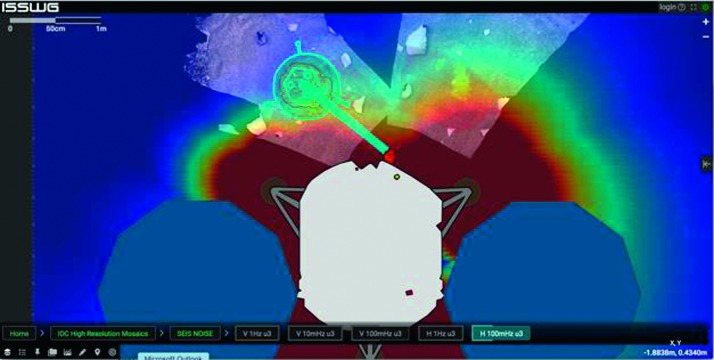
This figure presents the online tool developed by JPL/Caltech to evaluate and compare the performance of tentative deployment sites. The lander is represented in the lower part of the figure. Tentative positions for SEIS (pentagon) and WTS (circle) are figured on the top. The color code goes from blue to red. It represents the percentage of budget allocation for the wind noise on the overall instrument (red equal or superior to 100, deep blue zero percent of the allocation) for the 100 mHz horizontal noise

In addition to these requirements, one of the key contributors to the SEIS noise is expected to be the mechanical noise of the lander transmitted through the ground to the seismometer (lander wind noise). The noise model described in Sect. 3.4 takes into account a typical deployment location within the zone that the Instrument Deployment Arm (IDA) can reach (shown in Fig. 27). Of course, depending on the actual conditions of the Mars landing site, the mission may not be able to deploy the seismometer at its nominal location. Once landed, a whole week is assigned to the selection of the best site for instrument deployment. To select the location where the instrument is deployed, two families of parameters have to be evaluated.

The first parameters are linked to the engineering capabilities of the deployment system: in order to deploy SEIS correctly on its three feet, the underlying terrain needs to have a tilt below 15°, underlying rocks shall not be bigger than 3 cm and the terrain slope shall be compatible with tether and tether loop deployment. JPL has developed a set of tools that evaluates these site geometric properties for all locations within the deployment zone (Abarca et al. 2018). These tools use images, mosaics and digital elevation models generated from the stereo images taken after landing by the camera on the arm. The tools are designed to quickly determine where the seismometer can be deployed that meets the requirements.

The second family of parameters evaluated is related to the lander vibrations induced by the wind. In order to assess the performance impact of the lander motion, the noise model assumes that the ground behaves as an elastic medium. Two major parameters influence this noise contribution: the distance to the lander feet, the mean slope on which the lander is located and the mean wind speed and direction. We have developed a tool, described in Murdoch et al. (2017a) that estimates the noise map at the site depending on the actual landing conditions. The tool takes in account the contribution from HP3 wind noise.

#### Sensor Assembly Deployment

##### Deck to Ground

**Fig. 28:**
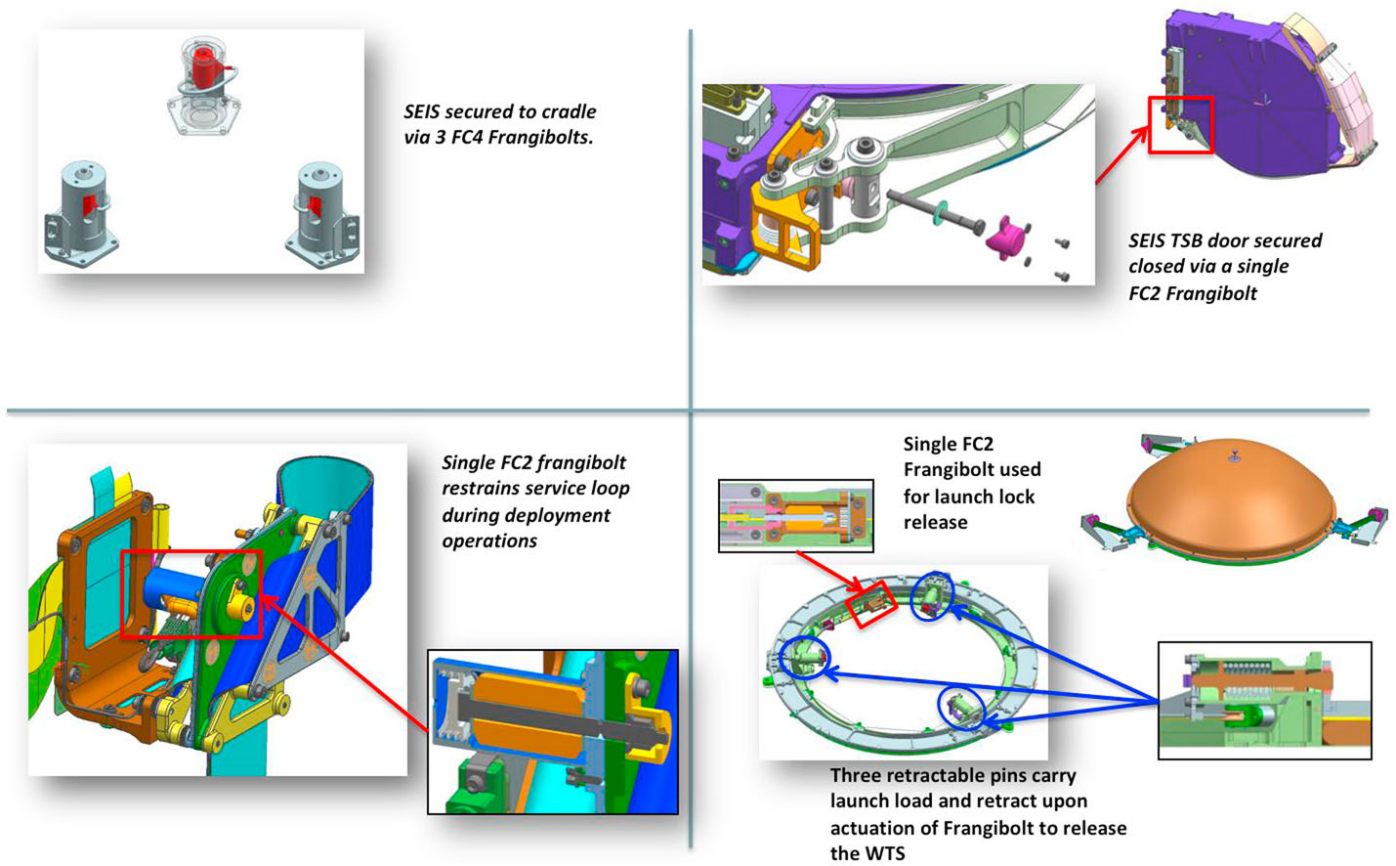
Details of the 4 Frangibolt firings actions, each of them an irreversible action associated with locking systems. Respectively, these are (top left) the locking system of the SEIS SA on the deck, (top right) the Tether box opening for mechanical decoupling of the tether from the lander, (bottom left) the LSA opening for mechanical decoupling of the tether from the SEIS SA and (bottom right) the locking system of the Wind Shield

**Fig. 29:**
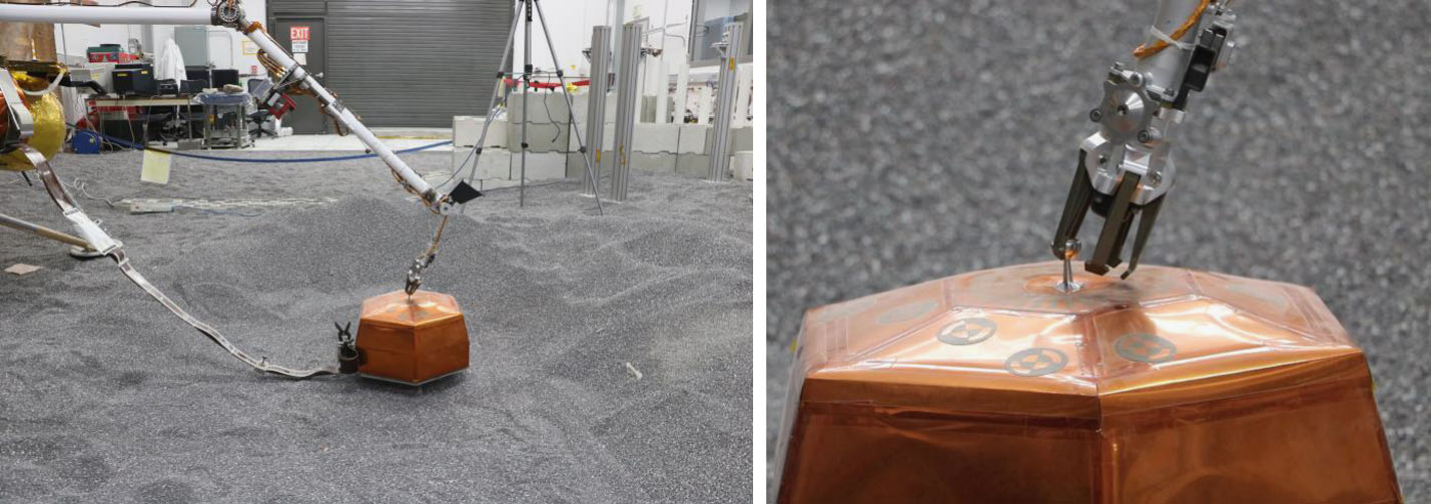
SEIS Deployment (left) and grapple release (right)

After release of the SEIS SA from the deck by the activation of the Frangibolts ((1) in Fig. 28), the Instrument Deployment Arm (IDA) picks up SEIS from the spacecraft deck and places it on the Martian surface (Fig. 12 and Fig. 29) at a pre-selected location determined by the ISSWG team based on analysis of the detailed Digital Elevation Map (DEM) derived from the Instrument Deployment Camera (IDC) mounted on the IDA. At this point it is determined that SEIS is on ground and within recapture constraints using information from the IDA, IDC and the lander-mounted Instrument Context Camera (ICC). At the same time the coarse-tiltmeter on SEIS is used to verify that SEIS is within the constraints at the selected site by verifying that the instrument tilt meets the deployment requirements and in good agreement with the DEM pre-calculated tilt. When all of the above requirements are met, a decision to release the IDA grapple that holds the seismometer is made.

##### Grapple Release

The next step is the verification of the grapple release (Fig. 29). This is done both by information from the IDC.

##### SEIS Placement Imaging

Once the grapple is released an imaging campaign begins to evaluate the state of SEIS on the ground and establish that its location meets the deployment requirements. IDC stereo imaging of SEIS is acquired to localize it in order to evaluate the position and orientation of SEIS in the workspace and confirm that the placement constraints were met. Specifically, the imaging will focus on the configuration of the tether that links SEIS to the lander and on the location of the feet, to determine that it is safe to proceed with leveling. This step includes an analysis to ascertain that the WTS can still be deployed without touching the seismometer.

##### Leveling

SEIS leveling is a two-step process including ‘*Initial Leveling*’ followed by a ‘*Leveling Low*’ step in which the leveled seismometer is lowered so that its center of mass is as close to the ground as possible without touching the surface. The latter step requires some elaboration as it differs from most terrestrial installations. The SEIS LVL system is capable of leveling the seismometer on a surface tilt of up to 15°. This is a factor of $\sim 3$ more than most terrestrial seismometer systems. Therefore, unlike most terrestrial installations, there is a possibility that the seismometer would be levelled while there is a significant bias from the ground that can be trimmed down by evenly lowering the seismometer. This is desirable both for shifting vibrational modes of the LVL system to higher frequencies and for allowing more room between the seismometer and the WTS.

##### Initial Leveling

The SEIS leveling system (LVL) is activated and SEIS is leveled to within its 0.1° requirement. The tilt is verified using both its coarse and precise tiltmeters. Further imaging of SEIS is used to establish that no significant change to the SEIS system has occurred and to determine the lowering distance in the next step.

##### Leveling Low

At this point the SEIS system is evenly lowered to its pre-determined “Low” position and a final tilt of $<0.1^{\circ}$ is established. In order to insure no contact with the ground a Digital Elevation model (DEM) integrating the current location of Sensor Assembly will be used. The latter is assessed from images taken by Instrument Deployment Camera. With the knowledge of the pebbles size underneath the Sensor Assembly reported in this DEM and with a margin of 0.5 cm, the maximum movement on each leveling leg able to lower the Sensor Assembly as much as possible will be computed.

##### VBB Operations

This deployment step is key to determining that it is safe to proceed to the first committal event of releasing the tether from its box on the lander. Until this point, only Short Period (SP) seismometers were turned on providing ancillary non-decisional data. Now that the seismometer is leveled, the Very Broad Band (VBB) sensors are turned on and centered. We proceed with 12-hour period of daytime (since the seismometer cannot be operated at night without the protection of the WTS) monitoring of the VBB in Engineering, low-gain, mode to make sure the sensors do not saturate, as would be the case if the tilt changed by more than 0.25°. Simultaneously, the tilt is monitored for drift by the precise tiltmeter. At this point we are ready to release the Tether Box—the first “committal event”.

##### Tether Box Release

**Fig. 30:**
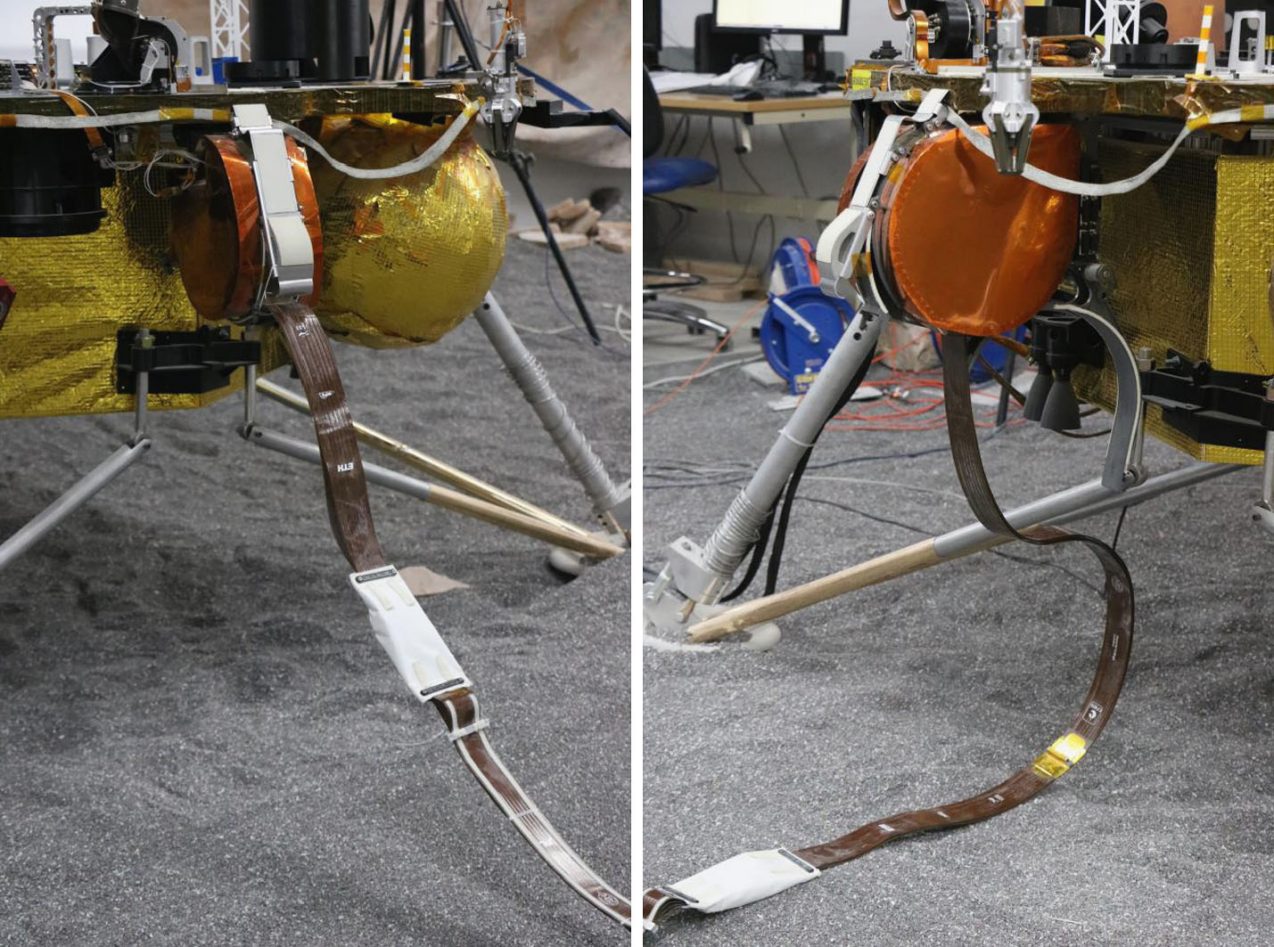
The closed tether box (left) is opened to release the tether onto the surface (right)

At the end of the VBB operations step we are ready to commit to the SEIS location, since after release of the tether from its box below the lander deck the ability to change location of the seismometer would be minimal (a few centimeters). The SEIS tether is released by opening the tether box door (Frangibolt (2) activation, Fig. 28 and Fig. 30) and the tether drops to the Martian surface. Although the team has studied a number of tether configurations based on a range of landing site terrain, slopes and obstacles, it is difficult to predict the precise configuration of the tether once it is released. While minor adjustment of the tether layout near the interface with the Service Loop is possible by shifting the position of a pinning mass (discussed below) with the IDA, once the tether is deployed it is more challenging and therefore unlikely that the seismometer itself will be moved. Once it is confirmed by the Instrument Context Camera (ICC) that the tether box door is fully open and the tether is completely released, SEIS monitoring operations are continued.

##### Polarization Assessment

The next committal event is the opening of the service loop by releasing the tether shunt. The decision to open the service loop is based upon continuing to meet placement, functional and derived performance requirements. This is determined based on the tether configuration, tilt and tilt stability and the VBB ability to re-center. This decision is based on imagery, VBB and tilt data.

##### Tether Shunt Release

**Fig. 31:**
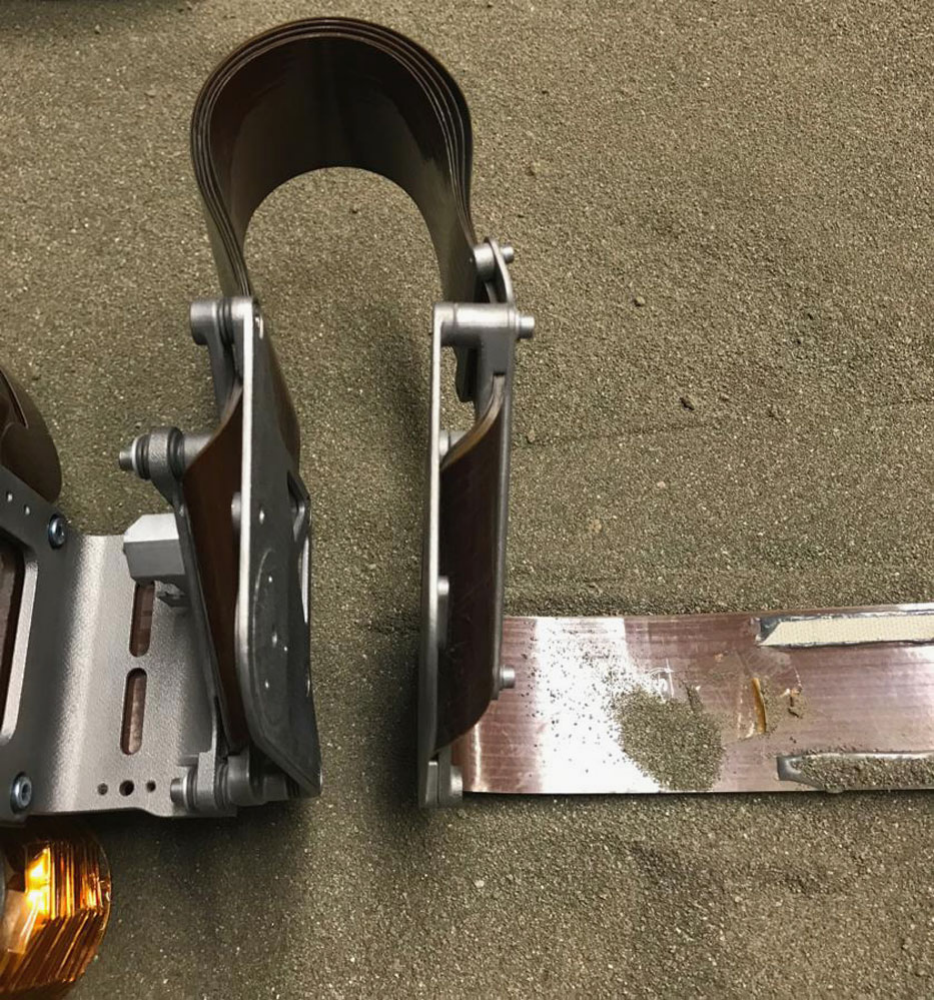
Opening the Load Shunt Assembly (LSA) on the tether. The LSA mechanically decouples the seismometer from thermoelastic expansion and contraction of the tether

The service loop is opened by activating the Frangibolt that keeps it in the closed position (Frangibolt (3) activation, Fig. 28 and Fig. 31). Once this is done, it is necessary to confirm that the service loop is completely open and that there is no contact between the two parts of the Load Shunt Assembly (LSA) previously held together by the Frangibolt. This is confirmed by Instrument Deployment Camera (IDC) image.

##### Service Loop Assessment

At this stage, the seismic monitoring is continued with particular focus on analysis of signal polarization that might indicate that the LSA is shorted. If there is contact between the two LSA plates, the free plate can be shifted by using the IDA scoop to pull the pinning mass away from the seismometer. Another key factor is the ability to deploy the WTS over the seismometer without touching any part of the seismometer including the LSA. Therefore, the final configuration of the seismometer in its “leveled low” position with an open LSA cannot exceed a pre-determined volume which will be encapsulated by the WTS. At this stage, the analysis of the terrain and the final configuration of the seismometer is carried out to ensure that the seismometer is well within its “Do Not Exceed” volume for WTS placement. The WTS placement is selected accordingly. All the while VBB monitoring is continued to confirm that the tilt drift remains within the recentering VBB capability.

##### WTS Deployment

**Fig. 32:**
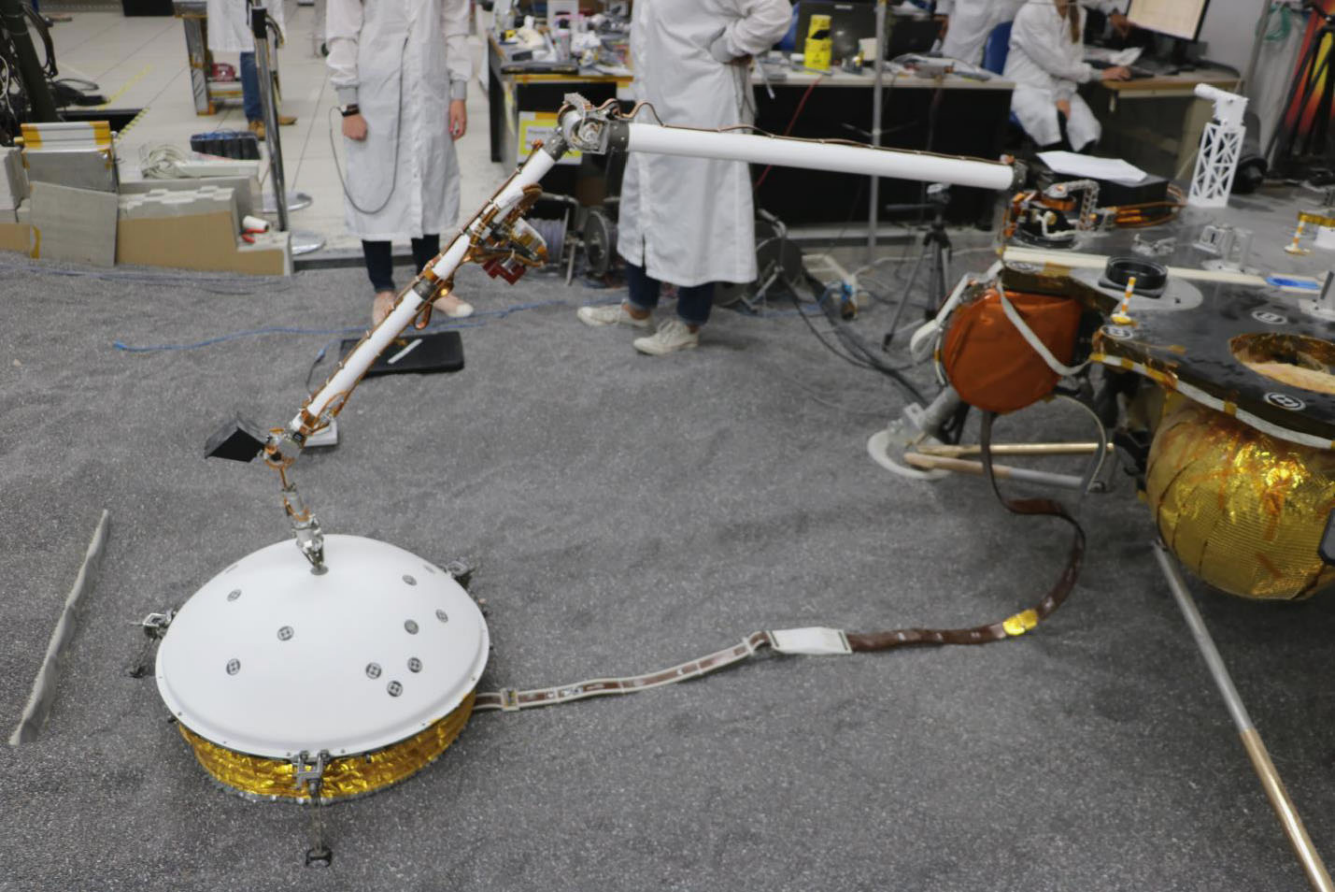
The final stage in SEIS deployment is the placement of the WTS over the sensor assembly

The final committal event is the deployment of the Wind and Thermal Shield (Fig. 32), which is picked up from its stowed position on the lander deck (during low wind conditions) after the last Frangibolt activation (Frangibolt (4) activation, Fig. 28) and placed over the seismometer, then confirming that it is on the ground in its desired position with IDA and IDC data. While grappled, the WTS position is approximated using the ICC image and a single IDC image of the WTS. The final position is determined from IDC stereo imaging. Only then is it determined whether or not it is safe to release the grapple from the WTS. Although the IDA is able to re-grapple the WTS, once the WTS is released it is extremely unlikely to be moved again.

##### WTS Grapple Release

As before, the verification of the grapple release is done by information from the IDC.

##### WTS Imaging

The imaging of the WTS chronicles its final position that may be used in future data analysis. The VBB can now be turned on continuously and a background noise level reduction should be noticeable. With this step completed, SEIS is fully deployed and the mission can proceed with the deployment of HP3.

## SEIS Sub-system Descriptions

### Very Broad Band Sensors

#### Overview

**Fig. 33:**
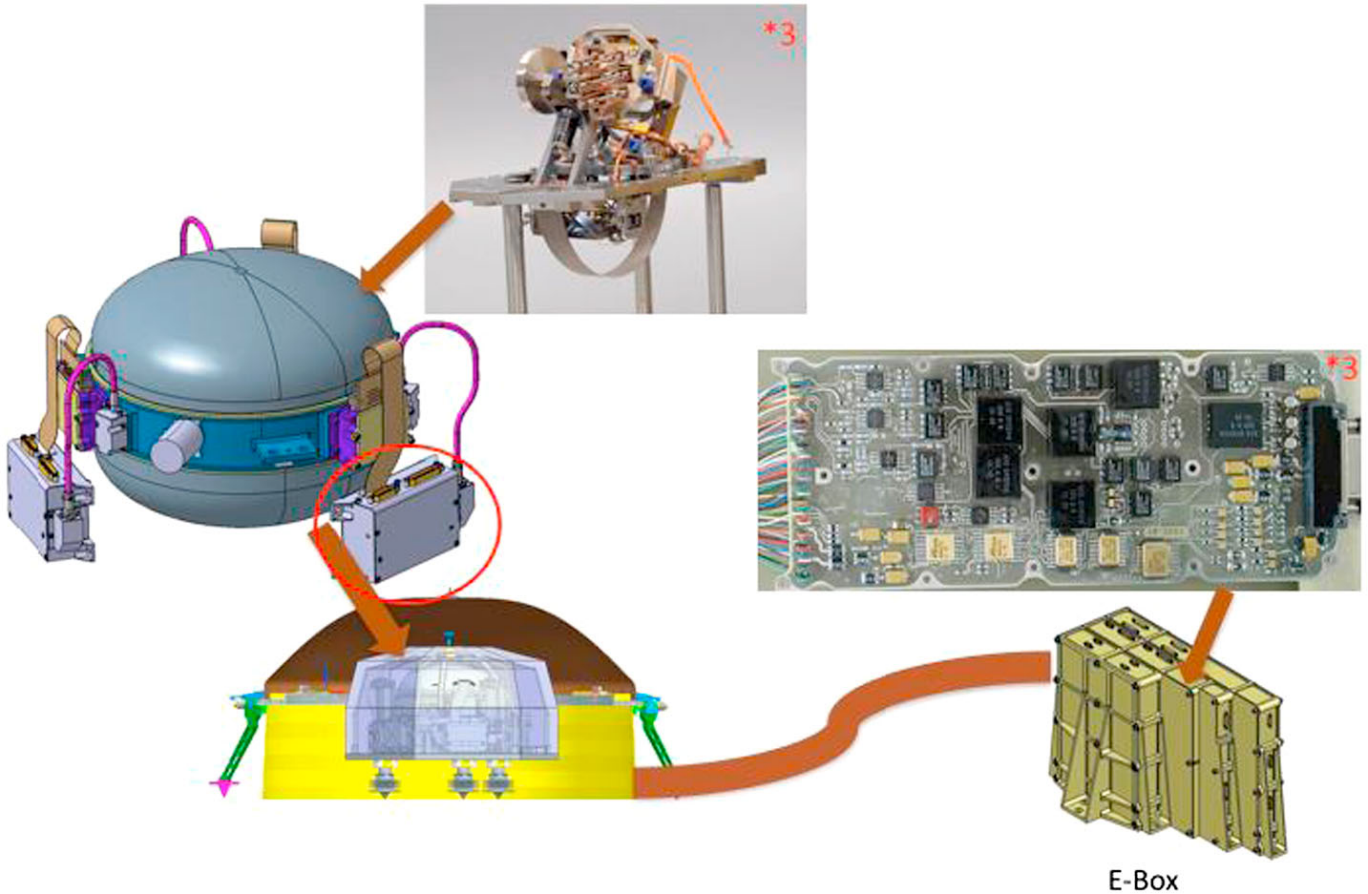
VBBs subsystem overview. It is composed of 3 sensors enclosed in the Evacuated Container (EC), 3 proximity electronics boxes hosted on the LVL and 3 feedback boards located into the E-box. The Tether provides the electrical connection between the feedback board and the PE

The VBB is an ultra-sensitive very broad band (VBB) 3 axis oblique seismometer illustrated by Fig. 33. Its function is to transform the ground motion into analog electrical signals recorded and numerized by SEIS-AC. The VBB has two feedback modes: The first is the engineering (ENG) where the sensor operates as an accelerometer with two outputs flat in acceleration but provided with different gains. The second is the scientific (SCI) mode. The feedback provides then two outputs: a ground acceleration proportional to the position of the proof mass and therefore named as POS and a velocity output named as VEL. The POS output is flat in acceleration from DC to a few tenths of Hz while the VEL is relatively flat in ground velocity from $1/15~\mbox{Hz}$ to 20 Hz. Note that in ENG mode, the two outputs will still be named POS and VEL, even if they are both proportional to ground acceleration.

The VBB sensors are a trio of inverted pendulums stabilized with a leaf spring and tuned for Mars gravity (Sect. 5.1.2 for details on the pendulum including spring and Sect. 6.2.1 for details on their operation on Earth gravity). Each has a dedicated and tunable internal temperature compensation system, activated by micro-motors as well as a re-centering system based also on micro-motors. They are packaged in an evacuated sphere (EC) with internal passive vacuum pumps (getter) and operate in a high vacuum environment. The getters are described in detail by Petkov et al. (2018).

A differential capacitive displacement transducer detects movement of the housing relative to the pendulum and generates an analog output signal amplified by a Proximity Electronic (PE), mounted on the LVL ring. This signal is then sent to the Feedback Boards (FB), located in the SEIS electronics, in the lander warm box and feedback signals are returned to the sensor though the tether, to perform continuous re-centering by a first magnetic-coil actuator for both the ENG and SCI modes and response shaping for a second one in the SCI mode.

In the SCI mode, the velocity output is derived from the differential component of the analog feedback signal prior to amplification by output gain amplifiers. The moving parts of the VBB do not need to be locked for launch or EDL but must be leveled to within $\pm 0.3^{\circ}$ of the gravity vector to operate nominally.

**Fig. 34:**
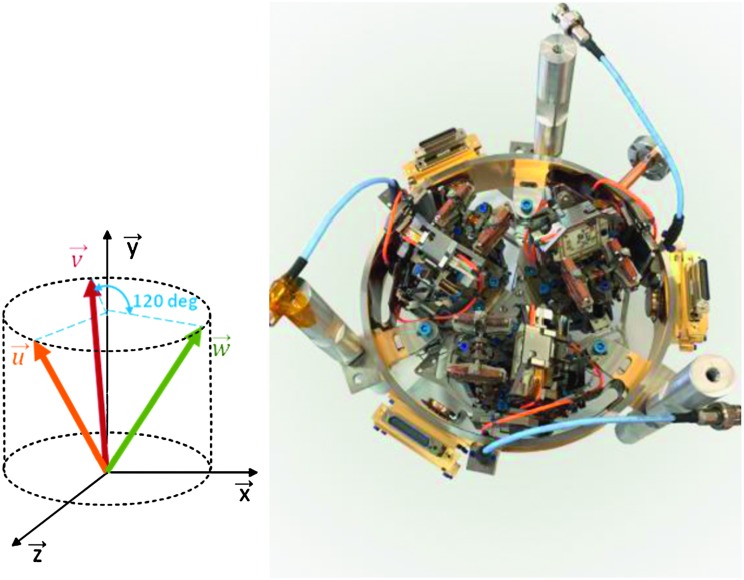
The 3 VBB sensors in the spherical evacuated container (right) which has an outside diameter of 198 mm. Their three sensing directions form the tetrahedron shown on the left

The 3 sensors enclosed in the spherical evacuated container are identical. Each sensor is measuring motion along one axis. They are then oriented in the sphere in order to register three acceleration directions (U, V, W) forming a tetrahedron and therefore measuring seismic motions in 3 dimensions (Fig. 34). Vertical and horizontal are produced by combining the three outputs after transfer function correction.

#### Mechanical Pendulum

**Fig. 35:**
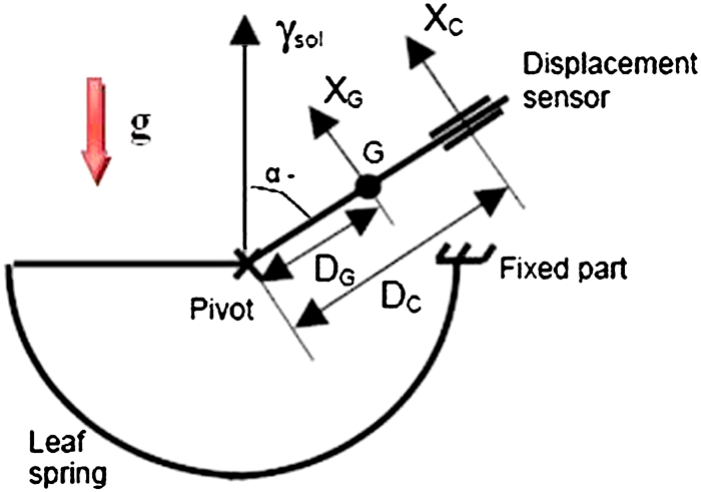
Inverted Pendulum Principle Schematic

**Fig. 36:**
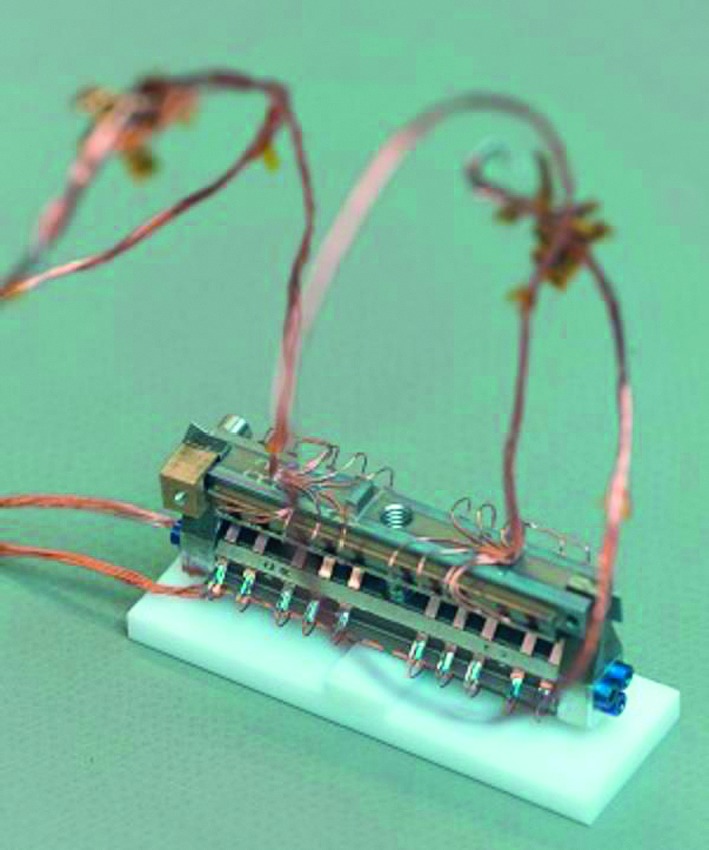
Picture of the pivot of VBB1, including electrical connections. The length of the pivot is 54 mm

**Fig. 37:**
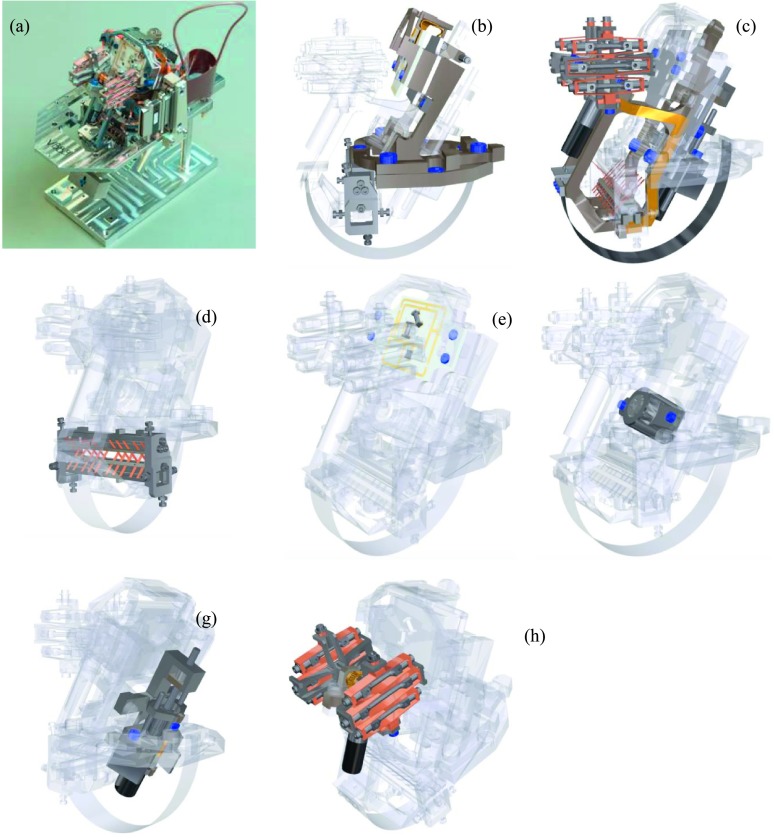
(**a**) One of the VBB sensor with Earth mass and VBB pendulum CAD views, illustrating the different functions of the sensor, (**b**) the fixed part, (**c**) the moving part, (**d**) the pivot, see Fig. 36, (**e**) the displacement Transducer, see Sect. 5.1.4, (**f**) the Feedback coils, see Sect. 5.1.5, (**g**) the re-centering motors, see Sect. 5.1.6 and Fig. 44, (**h**) the Thermal Compensation System, Sect. 5.1.8 and Fig. 45. A VBB pendulum fits in a $65\times 100\times 108~\mbox{mm}^{3}$ volume

**Fig. 38:**
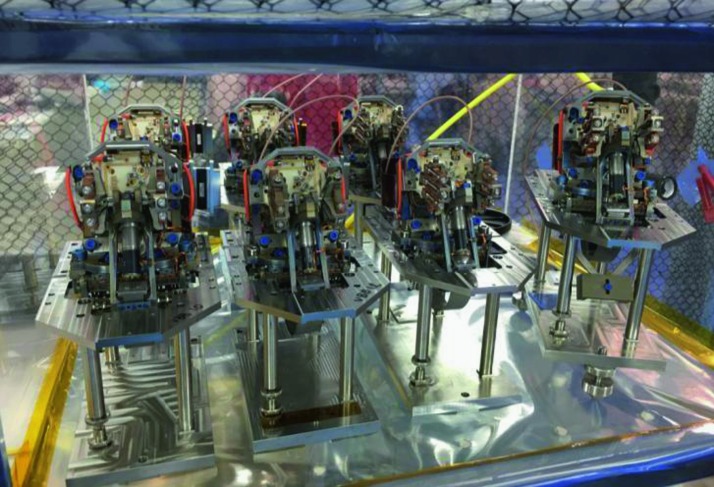
All Flight and spare VBBs prior to the cherry-pick process which lead to the selection of the 3 Flight and the 3 spare units. Each VBB pendulum fits in a $65\times 100\times 108~\mbox{mm}^{3}$ volume

The mobile part of the pendulum is suspended to a fixed frame through a flexure pivot and a leaf spring (Fig. 35). The flexure pivot provides the rotation axis of the pendulum with a very low stiffness (around $0.003~\mbox{N}\,\mbox{m}/\mbox{rad}$) and a very high stiffness in the five other degrees of freedom (above $900~\mbox{N}\,\mbox{m}/\mbox{rad}$). The flexure hinge allows a very low motion damping in the Evacuated Container as there is no sliding, rolling nor friction between parts. Electrical signals between fixed frame and mobile pendulum are transmitted through the pivot’s blades (Fig. 36). Figure 37 provides a complete view of the pendulum, together with its mechanisms described later in details while Fig. 38 shows all units manufactured for the project.

The configuration of the mechanical pendulum is such that the center of mass of the mobile part is above its axis of rotation. This configuration generates instability or a negative stiffness, which reduces the pendulum’s balancing spring and pivot stiffness. The natural frequency of the pendulum can then be expressed as:

4 $$ f_{0} = \frac{1}{2 \pi } \sqrt{ \frac{c - mg D_{g} \cos \alpha + p}{J}} = \frac{1}{2 \pi } \sqrt{ \frac{K}{J}}, $$

where $c$ is the leaf spring restoring torque, $p$ the pivot torque, $J$ the moment of inertia of the VBB with respect to the pivot rotation axis, $m$ the pendulum mobile part mass, $g$ the local gravity vector norm and $D_{g}$ the distance of the center of mass of the pendulum away from the pivot axis, $K$ the overall angular stiffness of the pendulum. $\alpha $ is the angle between the plane defined by the pivot’s rotation axis and local gravity vector and the vector between the pivot center and the center of mass of the mobile pendulum (see Fig. 35). The vector perpendicular to this plane defines the sensitivity direction of the VBB pendulum ($\alpha $ is also the angle between that axis and the horizontal plane).

The equilibrium of the pendulum is achieved when the leaf spring restoring torque in zero mechanical position ($M_{0}$) equals the gravity moment:

5 $$ M_{0} = m gD_{g} \sin \alpha . $$

As the leaf spring is balancing the pendulum weight, it can be sized in order to have the desired pendulum natural frequency. A stiff spring will increase the frequency, while a soft one can lead to an unstable pendulum, as soon as the gravity torque is larger than ($c+p$) in Eq. (4). The springs therefore were cherry picked individually for each VBB unit from a family of different springs in order to compensate for the dispersion of the actual pivot stiffness, weight momentum and geometry of each unit. Leaf springs are cut by electrical discharge machining from a Thermelast 0.12 mm thick sheet. They have a trapezoidal shape. Different families with various length and width have been produced to guarantee a good dispersion of properties. After thermal treatment—30 min at $750^{\circ}\mbox{C}$ to minimize their Young’s modulus temperature dependency—each spring is characterized by a momentum and stiffness. Springs are demagnetized before final mounting to minimize the VBB magnetic sensitivity.

The mechanical gain defines the capability to measure low frequency accelerations of the pendulum. It is the ratio of the displacement generated at the level of the displacement transducer and the acceleration that is generated along the sensitivity axis at very low frequencies. It is given by the following formula:

6 $$ G = \frac{m D_{g} D_{c}}{K}, $$

where $D_{c}$ is the distance between the pivot and the center of the capacitive plates and other terms have been defined in Eq. (4).

In this inverted pendulum configuration, the gravity term in (4) reduces the overall pendulum stiffness and allows to reach a low natural frequency in the range of 0.3–0.5 Hz. The sensor has this a high capability to measure low frequency accelerations, a high mechanical gain and a low Brownian noise while keeping the mobile mass small enough (190 g per axis). Equations (4) and (5) are also useful to see the properties of VBBs during Earth tests, for which momentum equilibrium must be met (see Sect. 6.2.2). Table 10 summarizes the properties of the pendulums. It shall be noted that the directions of sensitivity are close to 30° and not the 35.26° of a Galperin configuration. This orientation is optimizing the mechanical gain versus an increase of the self-noise of the recomposed vertical axis. Self-noise of the vertical axis will nevertheless be $1/(2\sqrt{3}) = 1.15$ larger on the vertical axis than on the oblique ones.

**Table 10:** Mechanical properties of the Flight VBB pendulum and of the VBB EM in Earth configuration. $J$ is the moment of inertia, $mD_{g}$ the product between mass and distance of the center of gravity to the pendulum and $\alpha $ the sensitivity direction. $Q$ values are measured in high vacuum below $10^{-5}~\mbox{mbar}$. $f_{0}$ variations with temperature are due to pivot stiffness variation in temperature. Note that for VBB1 as an example, the mechanical gain of the VBBE is reduced by 2.74, close from the gravity ratio between Earth and Mars gravity and that the gain on Mars will increase by another factor of 1.75

Unit	*J*	$mD_{g}$	$D_{c}$	*α*	$f_{0}$	*Q*	$f_{0}$	$f_{0}$
	(kg m^2^)	(kg m)	(m)	(^∘^)	20^∘^C (Hz)	20^∘^C	−10^∘^C (Hz)	−65^∘^C (Hz)
VBB1	2.71 × 10^−4^	4.88 × 10^−3^	0.057	29.33	0.506	>200	0.463	0.382
VBB2	2.59 × 10^−4^	4.83 × 10^−3^	0.057	29.15	0.546	>200	0.488	0.394
VBB3	2.67 × 10^−4^	5.05 × 10^−3^	0.057	29.66	0.474	>200	0.434	0.365
VBBE	4.38 × 10^−4^	1.78 × 10^−3^	0.057	30	0.325	>200	NA	NA

#### Pendulum Brownian Noise

**Fig. 39:**
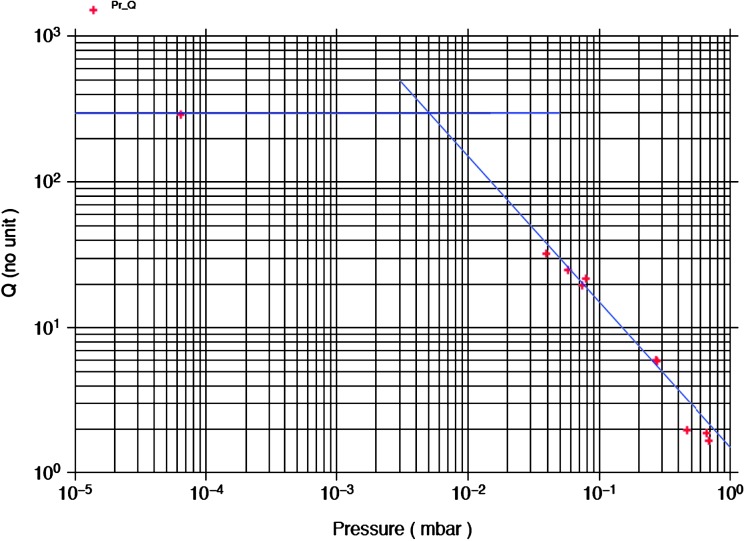
$Q$ of VBB 13 as a function of pressure. The gas in the chamber was air

The VBBs have been designed in order to have a very low Brownian noise for their moving part. Despite their moderate proof mass this is achieved by their low frequency and high $Q$. For a pendulum, the Brownian noise generates angular noise which translates in acceleration noise along the VBB axis as

7 $$ a_{\mathrm{brownian}} = \sqrt{8 \pi k_{B} T D_{c}^{2} \frac{f_{0}}{JQ}}, $$

where $k_{B}$ is the Boltzmann constant. The $Q$ as a function of pressure has been measured at ambient temperature for a few VBBs and is shown below for VBB13 (a unit used as spare), while individual $Q$ of the Flight VBBs are provided in Table 10. Below 0.1 mbar, $Q$ larger than 10 was found, while $Q$ drops to 5 for 0.3 mbar and $Q \sim 2$ for 0.5 mbar (Fig. 39).

This pressure dependency can be well understood with the Free Molecular model (Christian 1966), which predicts the following $Q$ proportionality:

8 $$ Q = \frac{k}{P} \sqrt{\frac{RT}{M}}, $$

where $K$ is the proportionality factor, $P$ the pressure, $R$ the gas constant, $T$ the thermodynamical temperature and $M$ the molecular mass of the residual gas. At $-25^{\circ}\mbox{C}$ and for internal pressure of 0.035 mbar, Brownian noise of the VBB is smaller than $10^{-10}~\mbox{m}\,\mbox{s}^{-2}/\mbox{Hz}^{1/2}$ and $Q$ is larger than 100. Such low pressure was one of the motivations for the EC, in addition to its very high temperature insulation, which for Mars conditions, remains as the most sensitive motivation. Pressure measurements were made during the ATLO phase including a last measurement, two months prior to the May, 5th, launch. They have shown that the pressure at launch will be smaller than 0.005 mbar, which will lead to $Q$ larger than 200 and therefore negligible Brownian noise.

#### Displacement Capacitive Sensors and Proximity Electronics

**Fig. 40:**
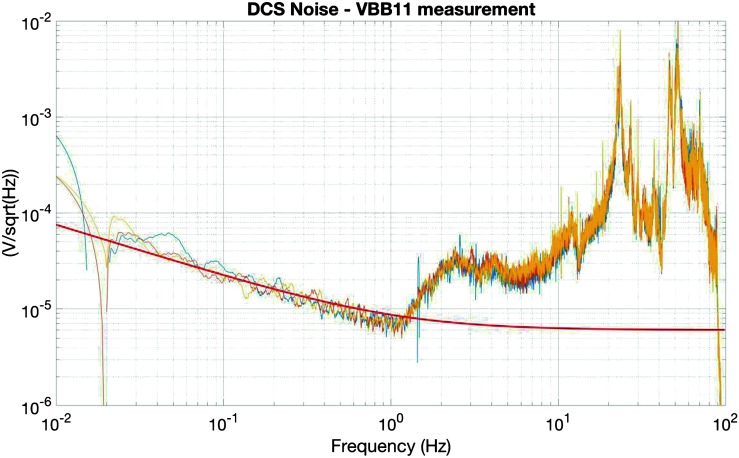
DCS noise. Nominal noise in red and VBB11 measurement in other colors. Noise above 1 Hz is residual micro-seismic background

The pendulums are equipped with a capacitive displacement sensor. It is composed of electrodes made of ceramic plates with a gold deposit mounted at the extremity of the pendulum (see Fig. 37e). A very small and extremely low dissipative front-end electronic is integrated in the electrodes. The proximity electronic, located close but outside the sphere, generates the excitation signal from a reference voltage and a clock integrated in the feedback board and transforms the charge back from the electrodes into a voltage proportional to the electrode’s displacement. Each axis has its own clock and all three clocks have been designed to prevent cross-talks. The proximity electronics also conditions the DCS measurement signal towards the feedback board. The measurement is fully differential. The nominal gain of the DCS is $2.6~\mbox{V}/\upmu\mbox{m}$. The DCS self-noise is shown in Fig. 40. It can be assumed as:

$$\begin{aligned} \textstyle\begin{array}{l@{\quad}l} 5.4 \times 1/(f/f_{0})^{0.54}~\upmu\mbox{V}\,\mbox{Hz}^{-1/2} & \mbox{below 1~Hz where}~f_{0}=1~\mbox{Hz}. \\ 5.4~\upmu\mbox{V}\,\mbox{Hz}^{-1/2} & \mbox{above 1 Hz}. \end{array}\displaystyle \end{aligned}$$

This translates into a proof mass displacement resolution of $2.1~\mbox{pm}\,\mbox{Hz}^{-1/2}$ at 1 Hz which will be practically the VBB ground displacement sensitivity at high frequency along the measurement direction. At low frequency, the DCS resolution increases as $1/\sqrt{f}$ and therefore to about 0.2 Angstrom at 100 s period.

#### Feedback and VBB Transfer Functions

A force feedback allows:

- erasing the natural frequency peak in the transfer function (and partially the thermal variations of the mechanical gain),
- locking the proof mass mechanical zero near the electrical zero of the displacement transducer and thus a linear response,
- increasing the bandwidth,
- and tuning the sensor gains to the desired dynamic.

See Wielandt (2012) for a review on feedback sensors.

**Fig. 41:**
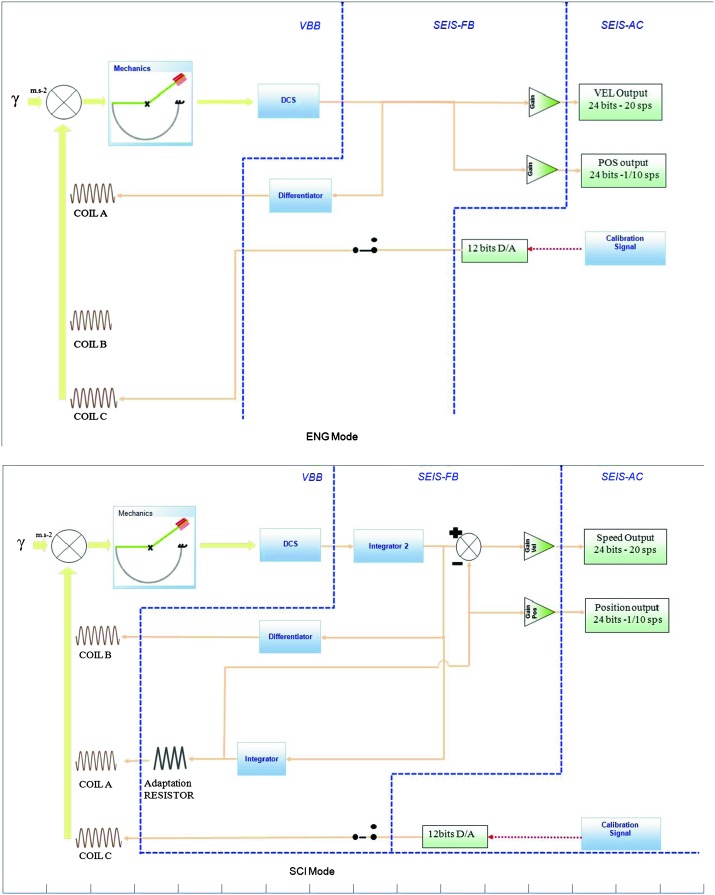
FB schematics. Top: ENG mode. Bottom: SCI mode

**Fig. 42:**
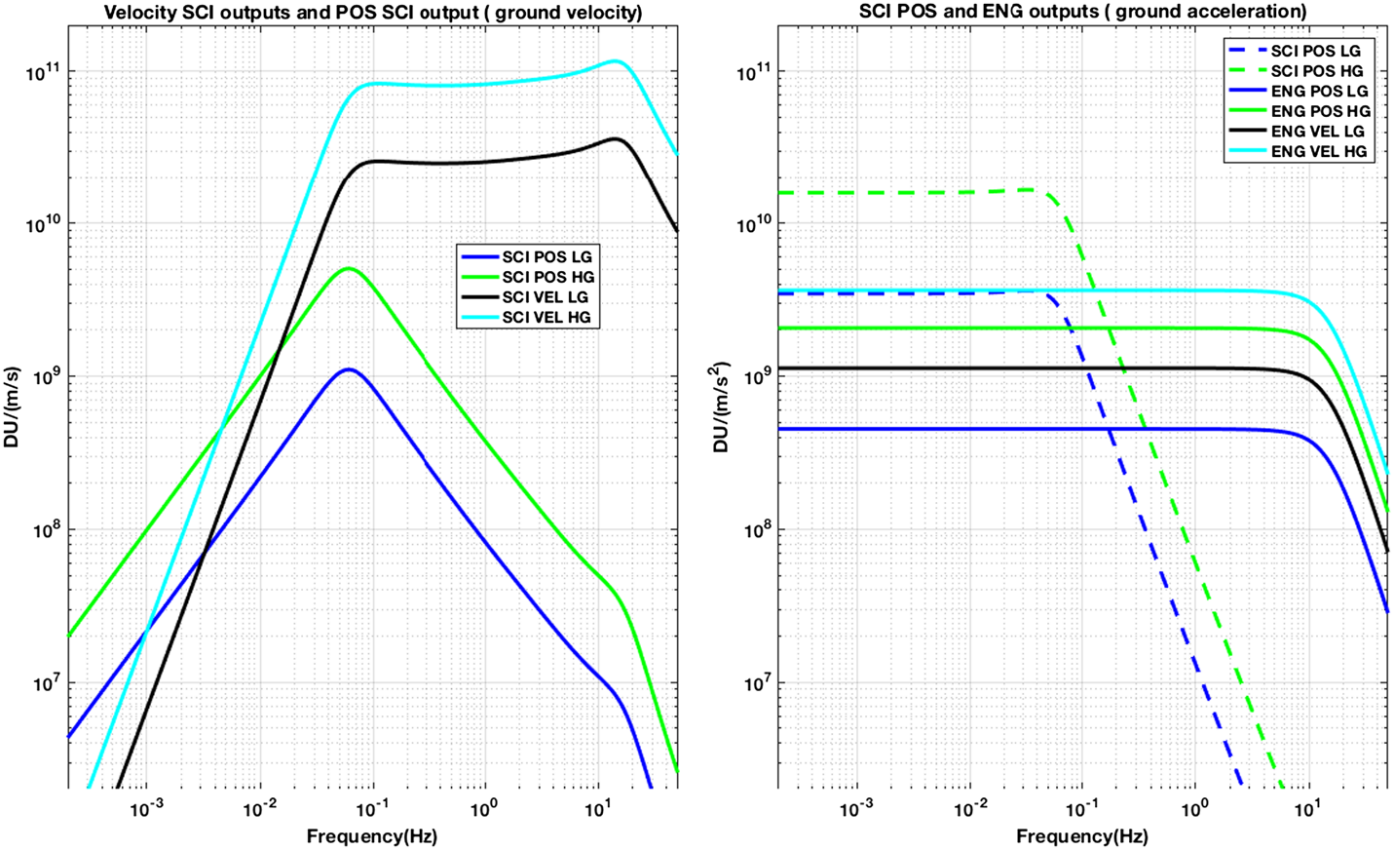
FB transfer function at $-55^{\circ}\mbox{C}$ for unit VBB10. On left are the Transfer functions for the ground velocity and at right are those for the ground acceleration, in Digital Unit (DU) per ground velocity ($\mbox{m}/\mbox{s}$) or ground acceleration ($\mbox{m}/\mbox{s}^{2}$)

The VBBs pendulums are equipped with 3 concentric voice coils: one is dedicated to calibration and interfaces with SEIS-AC DAC output. The two others are connected to the analog feedback circuit to close the control loop. The analog feedback input is the DCS signal. The feedback can work in 3 different modes:

- Scientific (SCI, Fig. 41 bottom) for optimal performances and nominal operations. The feedback loop gain is very high at very long periods (larger than 900) but is reduced in bandwidth in order to get amplification of the natural mechanical gain, with a minimum loop gain of about 100 at 10 s. Two different loops control the mechanical pendulum: the first one (Integrator + Coil A) is active from DC to 0.05 Hz and the second one (Derivator + Coil B) from 0.05 Hz to 10 Hz. The transfer function is relatively flat in ground velocity for frequencies between 0.067 Hz and 5 Hz (Fig. 42) but the small bump on the two sides of the pass band have different amplitudes on the 3 units due to dispersion of the actuator’s efficiency and mounting.
- Engineering (ENG, Fig. 41 top) is more robust (flat feedback loop strength larger than 700 in all the bandwidth) and has a higher clipping level. This mode is intended for starting the VBB, recentering and fallback in case of anomaly or degradation of the SCI mode. As it has less amplification than the scientific mode, its robustness is bigger: it can withstand a daily temperature variation greater than $\pm 50~\mbox{K}$ (VEL Low gain) and $\pm 150~\mbox{K}$ (POS Low Gain). It has been used for extensive testing and can operate on Mars in case of WTS failure or any other major failure leading to very large temperature variations. It will also be used during the commissioning for performing the Thermal Compensator optimal positioning and is used during recentering. The analog output is flat in acceleration over most of the bandwidth (Fig. 42).
- Open loop (OL) to investigate the health of the mechanical pendulum in case of abnormal behavior.

For all modes, two outputs are provided to SEIS-AC: the VEL and the POS output. Each output is equipped with a selectable gain (low or high) to allow dynamic adaptation. In ENG and Open loop mode, the only difference between the two outputs is the gain, while in SCI mode, the VEL output is a ground velocity output with a 2nd order high-pass filter with 6 dB corner frequency at about 0.0625 Hz (16 s period), while the POS is a ground acceleration flat output with a 2nd order low-pass filter with the same corner frequency. A lower cut-off frequency could not be implemented due to the limited size of the low temperature sensitive space qualified capacitors implemented in the VBB feedback and the required noise at 100 s period, which prevented the use of larger resistors in the implementation of the integrator cutoff frequency. Note that the SP was able to accommodate larger automotive capacitors after a dedicated qualification process, but the latter have temperature sensitivity about 5 times larger and were finally not selected for implementation on the VBBs.

The feedback board provides also a logic interface with SEIS-AC to allow mode and gain changes and mechanisms activations. In addition, several housekeeping signals are transmitted to SEIS-AC for acquisition, especially temperature of the VBB sensor head, Proximity Electronics and Feedback card temperatures.

The gains are shown on Fig. 42. SCI LG VEL has at 0.02 Hz a gain of $2.8\times 10^{9}~\mbox{DU}/(\mbox{m}/\mbox{s})$ comparable or twice smaller to most of the IRIS global stations equipped with STS-1 Streckeisen seismometer and Quanterra digitizer (ranging between $3\times 10^{9}$ and $6\times 10^{9}~\mbox{DU}/(\mbox{m}/\mbox{s})$), while the gain, larger by 3.2 for the SCI HG, will be slightly larger. The gain is therefore much larger in the 0.05–10 Hz frequency band, mostly as a consequence of design choices related to the larger self-noise of space qualified amplifiers as compared to those used for Earth instrumentation. For periods larger than 250 s, the higher gain and lowest noise will be found for the SCI POS HG, which has a gain 3–10 times smaller than VHZ IRIS global stations, a limitation mostly related to the much larger temperature variations expected on Mars as compared to an STS-1 in a seismic vault on Earth. This POS HG channel has been designed to record tides and all long period signal with the best performances.

**Fig. 43:**
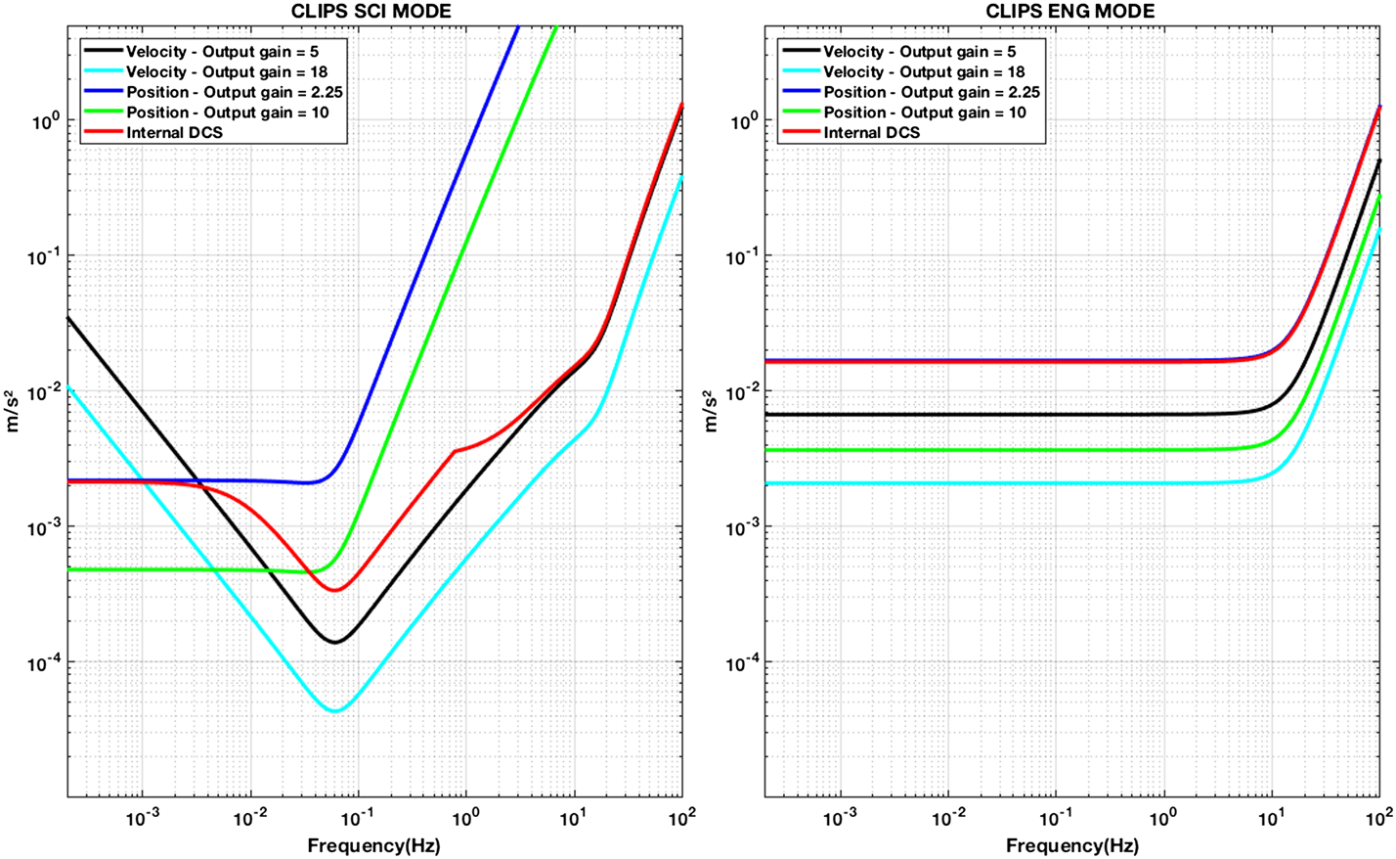
Saturation Levels at $-55^{\circ}\mbox{C}$ for unit VBB10. Left are those of the SCI output and right those of the ENG outputs. In addition to the saturation of the outputs gains, the internal saturation is shown in red. In SCI mode, this internal saturation occurs at long period at the output of the INT2 filter and above about 1 Hz at the output of the displacement transducer. This internal saturation matches therefore the Low Gain modes of the instruments at long periods for POS LG and at short periods for VEL LG. The same is valid for the ENG, where the internal saturation is then only due to the displacement transducer saturation and matches the one of the POS LG output

Saturation levels are shown in Fig. 43. At long periods, the SCI and ENG in LG are saturating for $0.002~\mbox{m}/\mbox{s}^{2}$ and $0.016~\mbox{m}/\mbox{s}^{2}$ respectively, which correspond on Mars to tilts of 0.03° and 0.25°. The first value is smaller than the requirement of the LVL system (0.1°) and will be achieved by the re-centering motors of the VBBs (see next subsection). In ENG LG mode however, the saturation level is larger than the LVL requirement and provide a backup in case of re-centering motor failure. Even in the VEL SCI high gain mode, saturation levels between 1 Hz and 3 Hz are 10 times larger than those of Viking in the most sensitive, high data rate mode (Anderson et al. 1977a, 1977b). They correspond to a ground velocity saturation level of $0.3~\mbox{mm}/\mbox{s}$ in the 0.05–10 Hz bandwidth in SCI LG, comparable to the SP HG saturation level and to about $0.1~\mbox{mm}/\mbox{s}$ in SCI HG. More precise gain values, as a function of temperature, are given in Table 11 and will of course be updated in the SEED metadata.

**Table 11:** Gain at various temperatures (Celsius) of the Flight Units and Earth Engineering unit. Gains of the SCI POS low gain are given at $5\times 10^{-5}~\mbox{Hz}$ in $10^{9}~\mbox{DU}/(\mbox{m}/\mbox{s}^{2})$ (or $\mbox{DU}/\mbox{nm}/\mbox{s}^{2}$) while those of the SCI VEL low gain are given at 100 s in $10^{9}~\mbox{DU}/(\mbox{m}/\mbox{s})$ or $\mbox{DU}/(\mbox{nm}/\mbox{s})$. LSBs are the inverse of these values. POS high gain is 4.565 times larger than low gain. VEL high gain is 3.2211 times larger than low gain

Unit	SCIPOS	SCIPOS	SCIPOS	SCIPOS	SCIVEL	SCIVEL	SCIVEL	SCIVEL
	−60^∘^	−40^∘^	−20^∘^	0^∘^	−60^∘^	−40^∘^	−20^∘^	0^∘^
VBB1	9.610	9.543	9.479	9.419	0.748	0.742	0.737	0.733
VBB2	9.704	9.718	9.734	9.751	0.755	0.756	0.757	0.758
VBB3	9.067	8.999	8.939	8.882	0.705	0.700	0.695	0.690

#### Re-centering

**Fig. 44:**
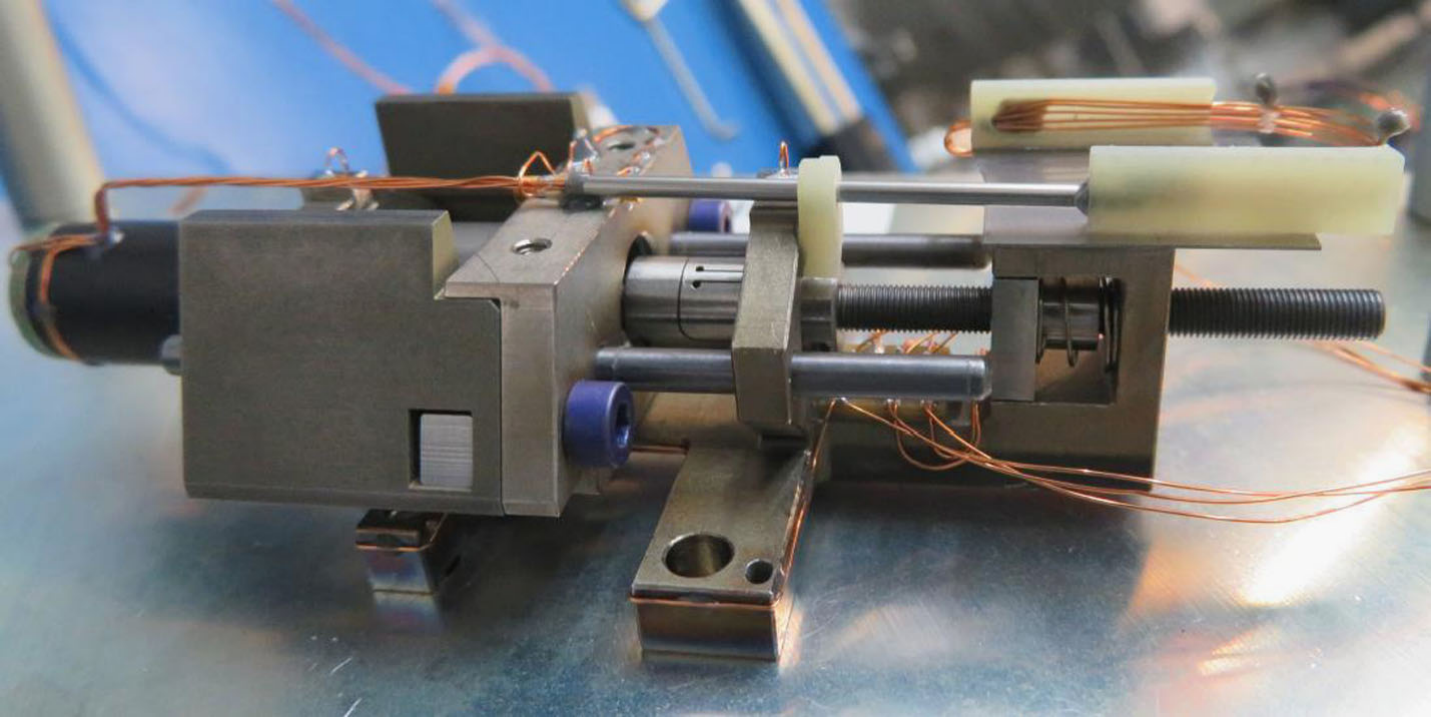
Re-centering mechanism from the top and from the side. A lead screw is driven by a stepper motor through a 1:256 gear box and a flex coupling to displace the mechanism. Two parallel guides prevent the motor gear box to rotate. To minimize overall mass, the motor is on the moving part. The re-centering mechanism fits in a $86\times 36\times 22~\mbox{mm}^{3}$ volume. The motor and gear box have a 10 mm diameter

Due to the large gains, the VBB pendulum includes a balancing mechanism (Fig. 44), which will be used for precise re-centering of the VBB sensors after the leveling made by the LVL. This mechanism has two main functions: at first it is used to adjust precisely the balance of the mechanical pendulum on Mars with respect to local gravity, leveling system inaccuracy, residual manufacturing offsets and secondly it serves to compensate for long term drifts that would otherwise drive the instrument into saturation.

This mechanism is located on the mobile part of the pendulum. Its principle is to move a 60 g mass along a 17 mm course until fine balancing is achieved. Compared to Earth instruments, the re-centering mechanisms have been oversized and have the capability to re-center one VBB for tilts from about $-2.8^{\circ} \mbox{ to } 3.5^{\circ}$ with respect to the leveled conditions on Mars, in order to accommodate local gravity variation and possible manufacturing offsets or aging of the pendulum. Because of the inverted pendulum design however, the natural frequency of each VBB will vary significantly, from unstable configuration (in open loop) up to 0.7 Hz within this tilt range and only a leveled platform will allow all three VBBs to achieve their nominal frequency and thus performances in Mars conditions.

To achieve a re-centering within 1 V in SCI POS high gain, fine positioning accuracy is required. Design tradeoffs lead to a stepper motor ($20~\mbox{steps}/\mbox{turn}$, 10 mm diameter) from Faulhaber and a 1:256 four stage planetary gear box with low backlash from Faulhaber. A counter nut preloaded with a spring avoids any backlash on the lead screw. Overall absolute positioning accuracy error is dominated by a harmonic one of the worm gear rotation with an amplitude of the order of magnitude of $20~\upmu\mbox{m}$. It is driven by the combination of screw/nut geometry and parallel guide play and results from a simple design optimized for reliability rather than absolute positioning. The step-by-step algorithm chosen to drive the mechanism relies only a relative positioning accuracy which is about a few $\upmu\mbox{m}$ over 12 steps and meets the requirement.

Gear box, lead screw and parallel guide are lubricated with Braycote grease to ensure a high reliability over the whole lifetime. The drawback is an operational constraint: the re-centering mechanism can be powered only above $-50^{\circ}\mbox{C}$ but was nevertheless qualified at $-65^{\circ}\mbox{C}$.

#### Magnetic Sensitivity

Most of the mobile part has been designed with non-magnetic material with the exception of the motors, invar column of the thermal compensation system and Thermelast spring. Magnetic momentum on the mobile part is dominated by far by the balancing motor. Based on component tests, the residual magnetic momentum has been bounded at $10^{-2}~\mbox{A}\,\mbox{m}^{-2}$, which would lead to $2\times 10^{-9}~\mbox{m}\,\mbox{s}^{-2}/\mbox{nT}$. Requirements has been set to $0.5\times 10^{-9}~\mbox{m}\,\mbox{s}^{-2}/\mbox{nT}$. All VBBs magnetic sensitivity have been measured in their final flight configuration. Measurements are spread between $0.1 \mbox{ to } 0.5\times 10^{-9}~\mbox{m}\,\mbox{s}^{-2}/\mbox{nT}$.

#### Thermal Compensator and Thermal Sensitivity

Thermal variations are expected to be the source of the largest non-seismic excursion of the VBBs output. As an example, Streckeisen STS-2 seismometers have a no-centering range of $\pm 25^{\circ}\mbox{C}$ for temperature and $\pm 0.03^{\circ}$ for Earth tilt (Kinemetrics 2017), corresponding to sensitivities of about $2\times 10^{-5}~\mbox{m}/\mbox{s}^{2}/{}^{\circ}\mbox{C}$ and comparable or better thermal sensitivities were required in order to not only have a continuous daily recording without recentering but also to meet thermal noise requirements at 100 s. Due to the lack of testing capabilities in Earth conditions and to the possibility to encounter aging, an active thermal compensator device has been integrated in the VBB design. The function of this second mechanism included on the VBB pendulum is to minimize the dependence of the sensor output signal on temperature variations. This allows reducing the part of the noise due to temperature in the VBB recordings and in turn allows to maximize the gain of the sensor.

**Fig. 45:**
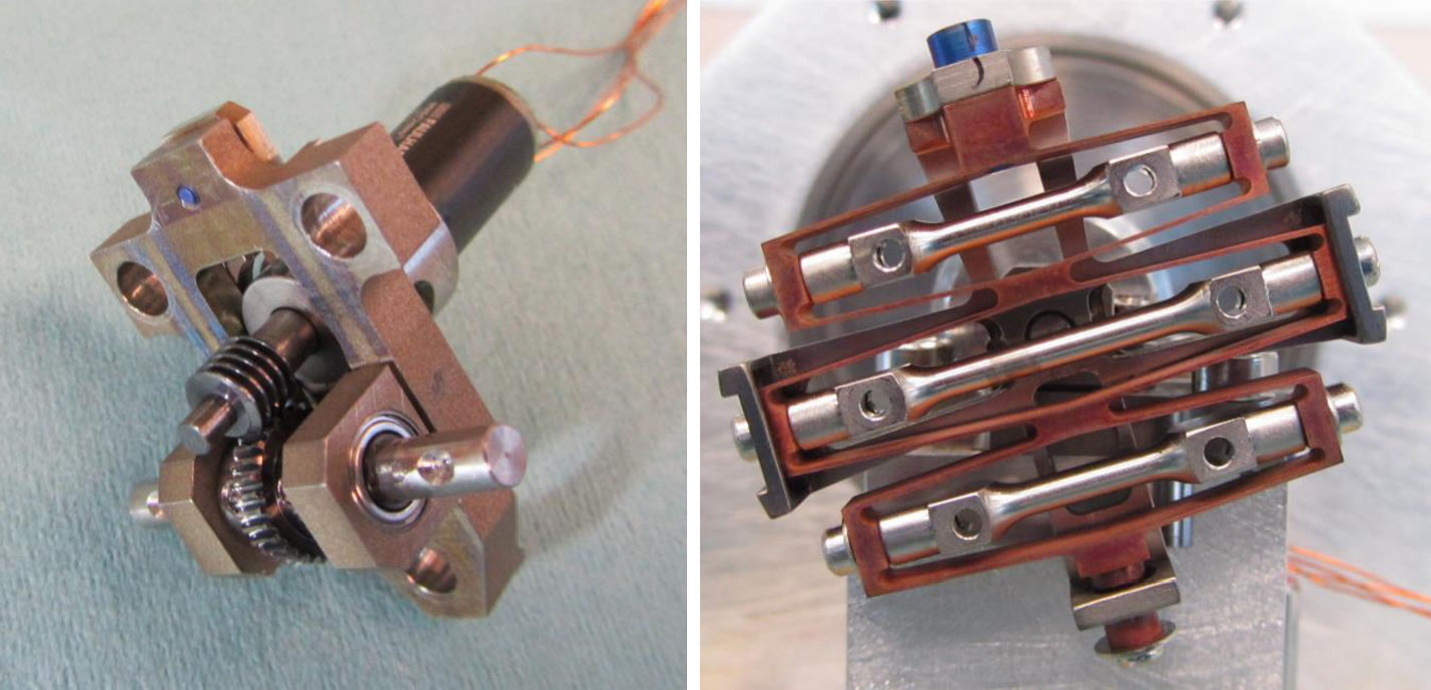
Thermal Compensator Device Mechanism. Two thermal compensation devices are mounted on a shaft. A tuning mechanism allows tuning of their orientation in a vertical plane. It is composed of a stepper motor, a 1:256 gear box and a worm screw. The compensation device is $37~\mbox{mm} \times 37.1~\mbox{mm}$ and can extend $12~\upmu\mbox{m}$ per °C. The orientation mechanism is 43.2 mm long. Its motor and gear box are 8 mm in diameter

The principle of this mechanism, shown in Fig. 45, is to translate passively along an axis a small mass on the mobile part of the pendulum proportionally to temperature variation, in order to adjust the balance of the mobile pendulum so that it stays centered while the temperature is changing. The compensation can be tuned in amplitude and sign by rotating the translation axis. When it is vertical, there is no balancing momentum change as the mass is moving. When horizontal, the compensation is at its maximum capability.

The passive compensation device is made of a CuBe_2_ cage and an invar column. The geometry has been optimized to maximize the center of gravity displacement with temperature. The latter is associated to length variation differences due to the different thermal expansion coefficients of the two metals. A structure around the cage acts as a stop-end to protect the mechanism under random vibrations. The orientation mechanism has an absolute rotation accuracy of 1°.

**Fig. 46:**
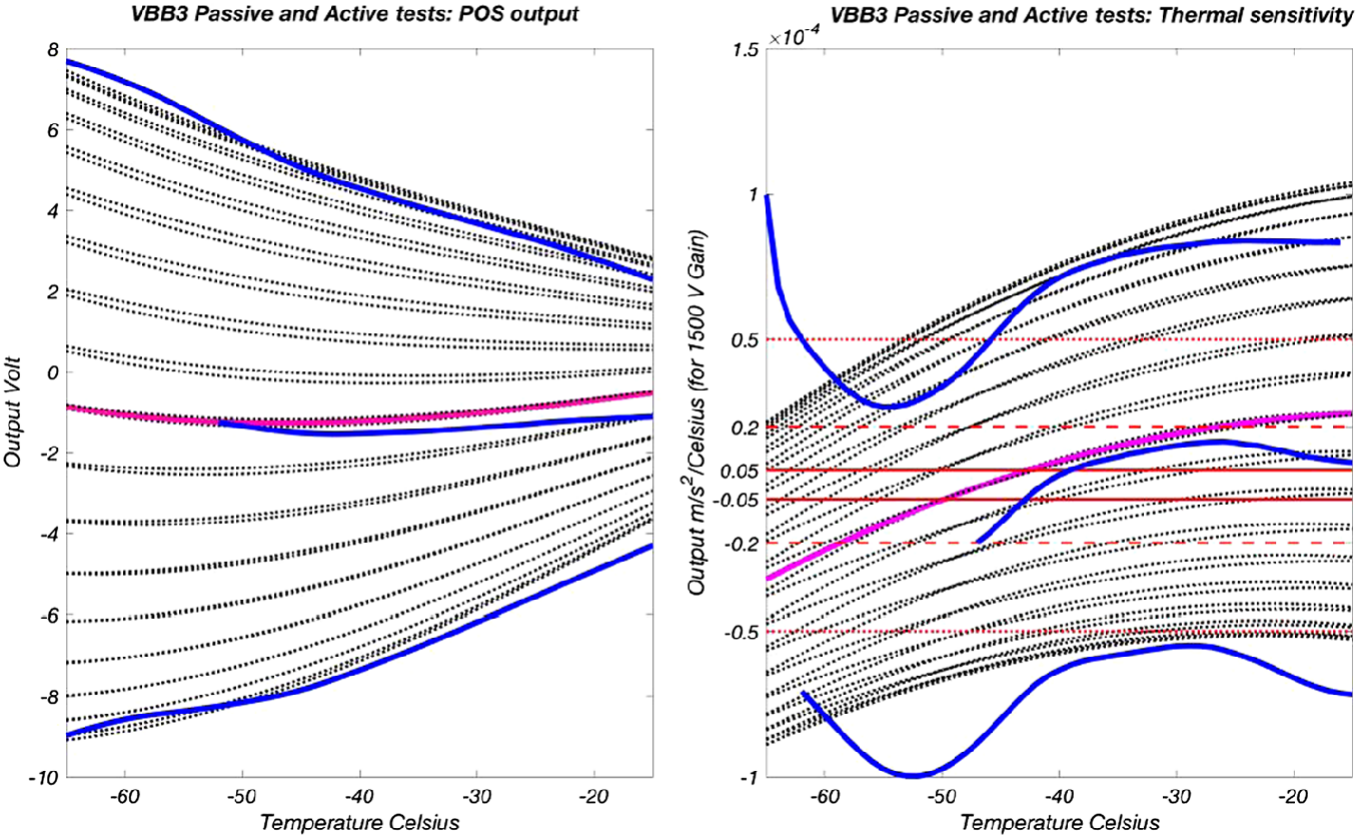
Thermal tests of one of the Flight VBBs (VBB3) during which the temperature of the VBB went from $-70^{\circ}\mbox{C}$ to $-10^{\circ}\mbox{C}$. The test was made on the POS ENG low gain (about $1500~\mbox{V}\,\mbox{s}^{2}/\mbox{m}$), which has about 8 times less gain than the POS SCI low gain. In the neutral position (magenta solid line), signal varies by about 0.5 Volt from $-52^{\circ}\mbox{C}$ to $-9^{\circ}\mbox{C}$. On the right, dotted, long dashed and continuous red lines are for $\pm 5\times 10^{-5}~\mbox{m}/\mbox{s}^{2}/\mbox{K}$, $\pm 2\times 10^{-5}~\mbox{m}/\mbox{s}^{2}/\mbox{K}$ and $\pm 5\times 10^{-6}~\mbox{m}/\mbox{s}^{2}/\mbox{K}$ thermal sensitivities. Blue line corresponds to data measured during passive heating of the VBB. Left is for signal output in Volt while right is the temperature derivative, for a fixed gain and for the temperature sensitive gain. Black dotted lines are for the different positions of the TCDM. The very large temperature sensitivity at $-60^{\circ}\mbox{C}$ is suspected to be exaggerated by the testing device on Earth

Figure 46 shows an example of measurements, made during passive heating of the VBB14 unit, which is the VBB3 flight unit. Passive heating was made in a thermal chamber, which was then cooled down to $-70^{\circ}\mbox{C}$ and adjusted back to ambient temperature passively, in order to minimize the thermal noise from either the chamber or the cooling/heating systems. Nevertheless, the VBB sensitivity in such a test is only an apparent one, as the test system is also likely injecting tilt (note that $10^{-5}~\mbox{m}/\mbox{s}^{2}/\mbox{K}$ is about $10^{-6}~\mbox{radian}/\mbox{K}$ in tilt in Earth tests). The three blue lines are the VBB output variation, from $-70^{\circ}$ to $-10^{\circ}$ in the two extreme positions of the TCDM (i.e. where the TCDM either adds or subtracts its maximum strength in terms of temperature sensitivity) and in the neutral position (i.e. were it is minimizing its strength), while the black thin lines are the theoretical VBB variations for given TCDM position. This illustrates the capability of the TCDM to change the sign of the sensor thermal sensitivity, as the slope of the output can be either tuned as growing or decreasing with temperature.

**Fig. 47:**
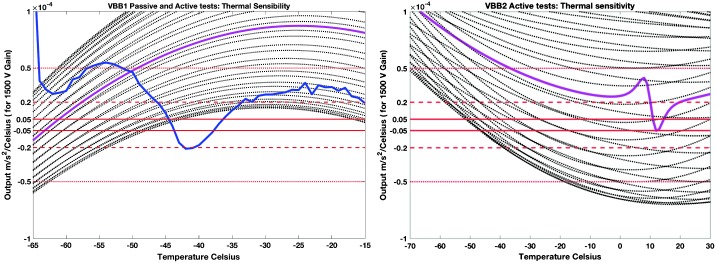
VBB 1 & 2 Thermal Sensitivity performances. VBB2 has higher thermal sensitivity at cold that exceed TCDM capabilities at low temperature. VBB2 meets its requirements over $-55^{\circ}\mbox{C}$. VBB1 is compliant over the full range. Color and lines definitions are the same as the right Fig. 46. Only the active tests results are shown for VBB2

The TCDM will have to be tuned regularly, e.g. every few months, in order to accommodate the seasonal changes on Mars, as the sensor’s sensitivity is expected to vary with temperature. This is illustrated by the neutral line in Fig. 46, where the apparent temperature VBB sensitivity varies from $-2\times 10^{-5}~\mbox{m}/\mbox{s}^{2}/\mbox{K}$ at $-50^{\circ}\mbox{C}$ to $1.5\times 10^{-5}~\mbox{m}/\mbox{s}^{2}/\mbox{K}$ at $-25^{\circ}\mbox{C}$. The sensitivity of the other VBBs is given in Fig. 47.

### Short Period Sensors

#### SP Introduction

InSight’s SP seismometer consists of a set of three sensors in enclosures that are deployed with the rest of SEIS on the surface and feedback (FB) electronics integrated into the Ebox on the lander. The SP sensors, with their front-end electronics, are connected to their lander electronics via the tether between SEIS and the lander. The SP sensors are labeled SP1, for the vertical and SP2 and 3 for the two horizontals separated by a 60° azimuth (a 90° separation was not possible on the tripod structure due to volume limitations on the LVL ring). The three sensors are attached around the outer ring of LVL directly on which the VBB sphere is also mounted. In contrast to the VBBs, the sensors have been designed to operate at up to a 15° tilt from the vertical, the leveling range for LVL. They will therefore be able to operate prior to the leveling of SEIS, including on the lander deck before the SEIS deployment. In this configuration, they will be in contact with the lander through the cradle. After deployment, they will, like the VBBs, be in contact with the ground through the 3 LVL feet mounted on the outer-ring of the LVL.

#### SP Sensors Description

**Fig. 48:**
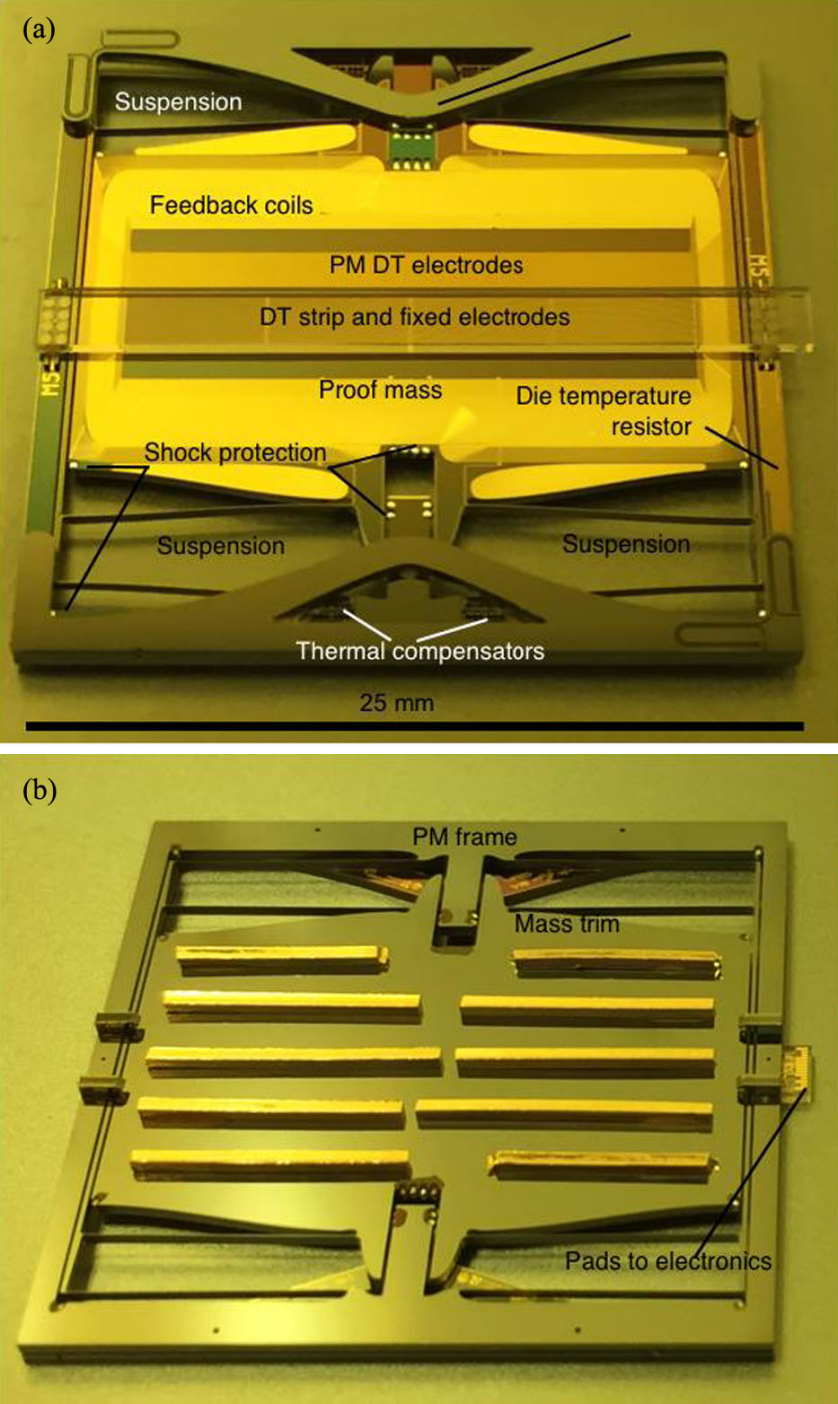
The unmounted SP sensors showing in (**a**) the top view of SP1, the vertical axis sensor and (**b**) the back view of SP2, one of the two horizontal axes

The SP sensors are micromachined from single-crystal silicon by through-wafer deep reactive-ion etching to produce a suspension and proof mass (PM) die with a fundamental vibrational mode of 6 Hz (Fig. 48). The sensors are of a novel design (Pike et al. 2018) to give a much lower noise floor than has been previously (e.g. Bernstein et al. 1999) or subsequently (Middlemiss et al. 2016) achieved by through-wafer etching of silicon, while being sufficiently robust to survive launch and landing and capable of autonomous levelling and operation on the surface of Mars.

The suspensions of the horizontal sensor dies are symmetric, while for the vertical the suspension is machined in an offset geometry so that under Mars gravity it takes up a symmetric configuration. Bumpers formed by the reflow of solder balls in cavities formed during the through-wafer etching protect the low-frequency suspension from damage (Delahunty and Pike 2014). Additional strengthening is provided by co-fabricated buttress structures that are bonded to the frame, with micromachined backstops inserted into the frame to provide protection for vibrations and shocks in the out-of-plane direction of the dies.

The displacement of the proof mass is sensed with a capacitive displacement transducer (DT): two interposed arrays of electrodes on the PM are differentially driven and facing sets of fixed electrodes plated on a fixed glass strip above the PM. The capacitance varies with the areal overlap of the driven and pickup electrodes providing a displacement signal with the $96\mbox{-}\upmu\mbox{m}$ periodicity of the array. The DT strip is connected mechanically and electrically to the PM frame using solder-ball bonding with pads at one end of the strip for electrical connection to the proximity electronics. Feedback is closed at the nearest null point of the periodic output of the DT. This allows operation over a large tilt range while keeping the actuation force low. The electrical connections to the coils and DT drives on the PM are routed along the suspension flexures using plated and sputtered gold traces.

The SP sensors are designed for low-noise operation at ambient pressure. The thermal Brownian noise is therefore minimized by the geometry of the DT which operates with Couette flow in the smallest gap between the PM and DT strip. Viscous flow in this gap, at around $12~\upmu\mbox{m}$, offers far less resistance with Couette flow than the alternative squeeze-film damping of a gap-based capacitance sensor. Additional reduction in the thermal Brownian noise is provided by the attachment of gold bar to the backside of the proof mass (Fig. 48b), with this mass trimming also used to set the fundamental resonance of the suspension. The SP sensors operate in feedback mode with electromagnetic actuation from coils plated onto the proof mass.

An approximately $1~\mbox{k}\Omega$ resistor is sputtered onto the PM frame to allow direct monitoring of the sensor temperature. Thermal compensation is incorporated into the base of the SP1, vertical suspension to attenuate the effect of temperature changes on the SP output (Liu and Pike 2015).

**Fig. 49:**
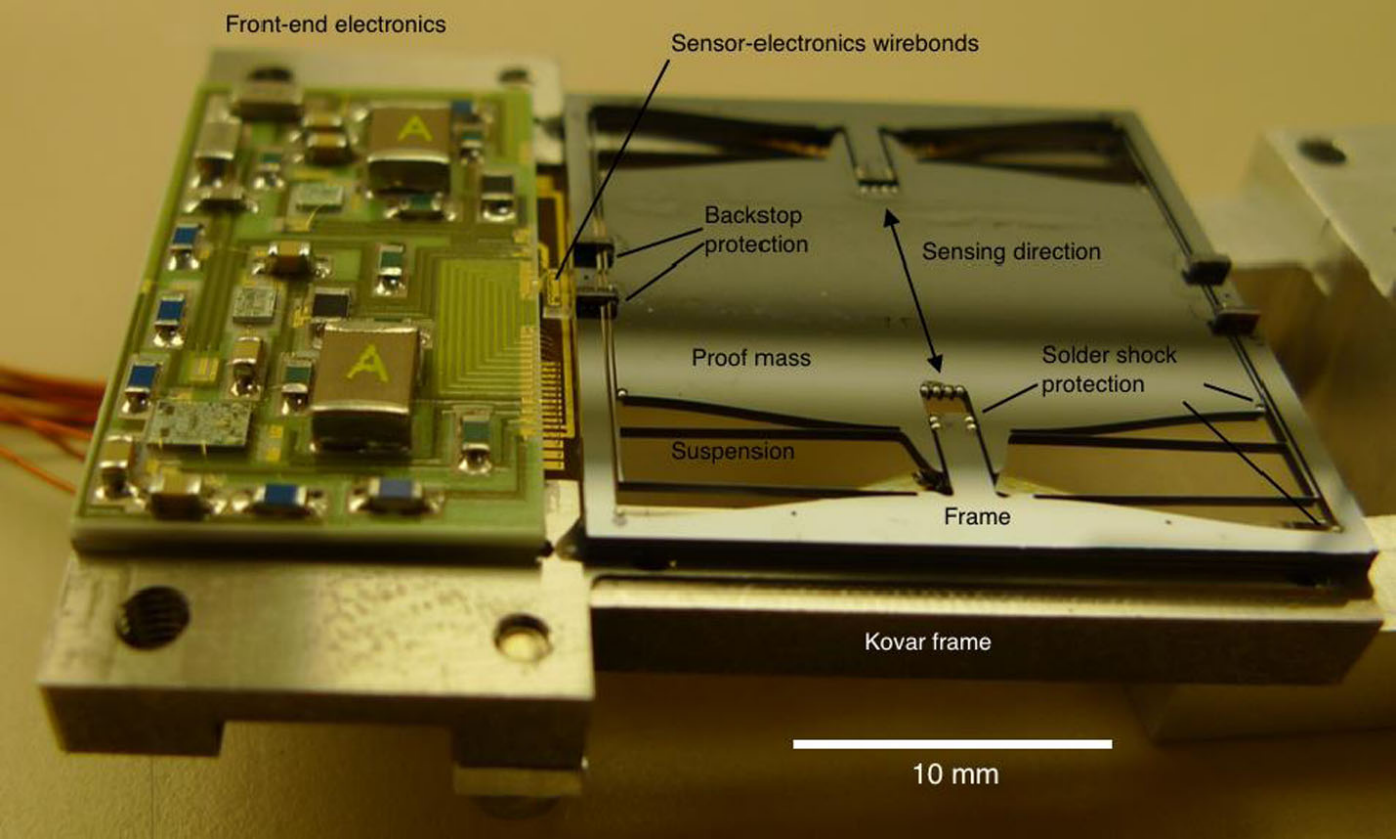
The SP sensor mounted on its frame and connected to the proximity electronics

**Fig. 50:**
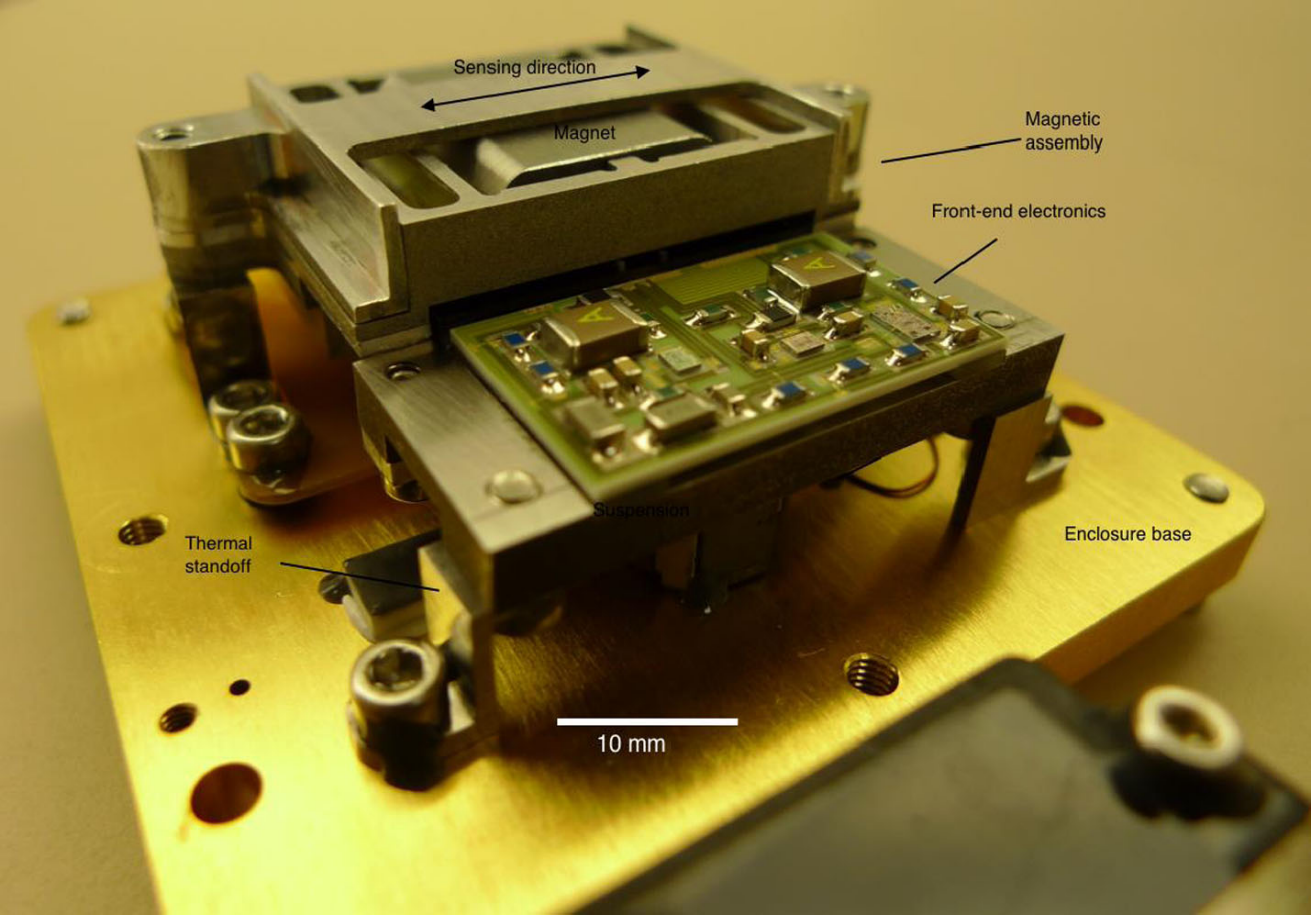
The SP sensor assembly with the magnetic assembly and proximity electronics mounted on the SP sensor enclosure base prior to sealing

**Fig. 51:**
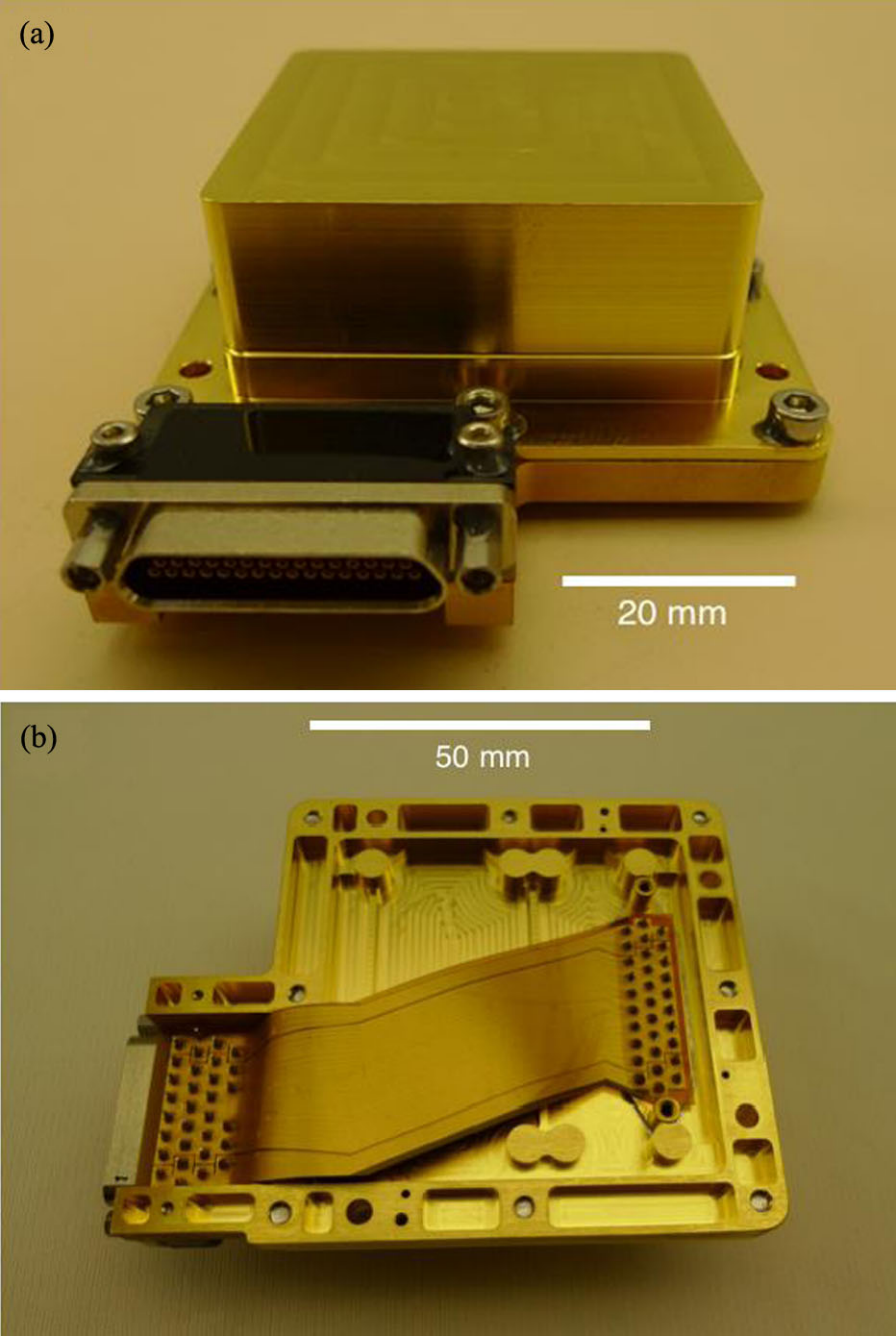
A sealed horizontal SP unit viewed from (**a**) the connector side and (**b**) the LVL mounting direction

The sensors are mounted on a Kovar frame which is inserted into a magnetic assembly to provide the actuation (Fig. 49). The sensors, magnets and front-end electronics are mounted on to the base of their enclosure via standoffs to provide thermal insulation between the sensors and the enclosure (Fig. 50). A second temperature sensor, a standard platinum resistance thermometer, is mounted on the enclosure base. The enclosure lid is hermetically sealed (Fig. 51) to the base and the enclosure evacuated and then backfilled with nitrogen to 10 mbar to provide a stable environment. Electrical feedthroughs to a flexprint external to the base of the enclosure provide routing to the connector to the tether.

The SP sensors are an innovative design which evolved significantly during InSight development so each was treated as a “protoflight” unit and subjected to qualification levels of vibration and thermal cycling for limited periods. Separate qualification units were also subjected to long term thermal cycling to simulate the mission on Mars and survival of the proof mass suspension was demonstrated for in-plane vibration levels up to 32g rms.

#### SP Electronics Description

**Fig. 52:**
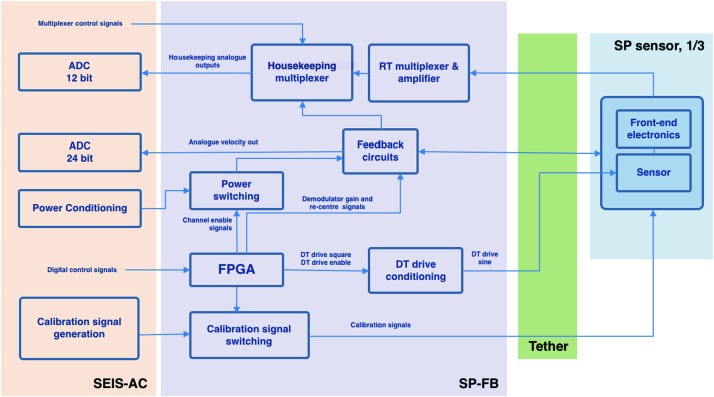
A schematic of the SP electronics. SEIS-SC, the SEIS acquisition electronics are installed in the Ebox of the lander while the SP sensors are deployed on the Martian surface on the SEIS instrument assembly

A schematic of the SP electronics is shown in Fig. 52. The front-end electronics include the DT preamplifiers and routing for the coil drives and temperature resistors on the sensor. The feedback (FB) electronics within the lander’s SEIS EBOX contain three sets of feedback electronics for the SP sensors and a DT drive conditioning circuit. The feedback provides an analogue velocity output (SP1, 2 and 3) at two selectable gains (see later for details) and an acceleration mass-position output (MPOS1, 2 and 3). The SP output signals are digitized by the SEIS-AC, the separate acquisition electronics, at 24 bit at either 100 sps or 20 sps, while MPOS are digitized at 12 bit as housekeeping signals, together with the temperature resistor signals interleaved on an analogue multiplexer at either 1 sps or $1/100~\mbox{sps}$. SP commanding consists of a power on followed by an enable for the SP sensors required for the observation. The SP also has a calibration capability which is enabled during operation by sending a calibration signal generated by SEIS-AC to the selected sensor. SP’s standard calibration signal consists of a shaped swept sine signal to validate the transfer function of the selected SP output. Power for SP is provided via SEIS-AC.

#### SP Transfer Function

**Fig. 53:**
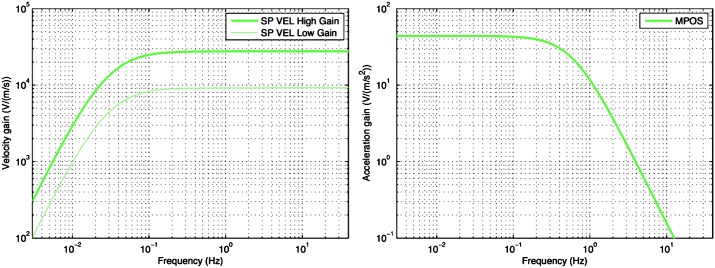
Solid lines: Generic shape of the Transfer functions of the SPs for the VEL output (left) and for the MPOS output (right). On the left, the dashed line is the low gain VEL output. The Full Scale Range of SEIS-AC is 25 Volt for SP VEL (with 24 bits) and 10 Volt for SP POS (with 12 bits for A/D and 16 bits after averaging)

The velocity output of SP is flat below 2 kHz with two gain settings, a high gain of $27{,}000~\mbox{V}/(\mbox{m}/\mbox{s})$ and a low gain of $9000~\mbox{V}/(\mbox{m}/\mbox{s})$, with a 2-pole roll-off at a corner frequency at 0.0286 Hz (35 s) with close to critical damping (Fig. 53). The high gain has been selected to ensure that the SEIS-AC digitizer noise is below the 10 Hz SP requirement of $10^{-8}~\mbox{m}/\mbox{s}^{2}/\sqrt{\mbox{Hz}}$. For the $\pm 12~\mbox{V}$ input range of SEIS-AC ADC the two gain settings correspond to a clip level of $0.9~\mbox{mm}/\mbox{s}$ and $0.3~\mbox{mm}/\mbox{s}$. A second output, mass position or MPOS, is the acceleration signal required to keep the feedback closed below the corner frequency of the velocity output. The gain is flat in acceleration, with a gain of $44~\mbox{V}/\mbox{m}/\mbox{s}^{2}$ and a low pass roll off at a corner frequency of 0.6 Hz. These transfer functions are illustrated in Fig. 45.

#### SP Thermal Response

From previous temperature measurements on the surface of Mars thermal effects are expected to be the major noise injection directly into the SP (Mimoun et al. 2017). For the vertical unit, SP1, the transduction is through the thermal coefficient of Young’s modulus of the silicon suspension ($57~\mbox{ppm}/\mbox{K}$, Liu and Pike 2015) which will cause movement of the proof mass under gravity due to spring softening. On Mars this would give an uncompensated thermal coefficient of $2.2\times 10^{-4}~\mbox{m}/\mbox{s}^{2}/\mbox{K}$, considerably above the requirement of $5\times 10^{-5}\mbox{ m}/\mbox{s}^{2}/\mbox{K}$. Therefore, the suspension of SP1 is passively thermally compensated with solder reflowed into cavities at the base of the suspension (Liu and Pike 2015). The resulting mismatch in the thermal coefficient of expansion (TCE) gives a thermoelastic tilt that can compensate for the suspension softening. The design target of the solder compensator was therefore an attenuation of ten in the thermal response.

In addition, for all the SPs there is a thermoelastic response due to the sensor materials TCE mismatch. This mismatch will cause tilts which will inject a component of gravity into the SP’s outputs. Any external thermoelastic stress is minimized by the compliant mounting points of the PM die. Therefore, the dominant TCE contribution is between the silicon and the borosilicate glass of the DT strip, which are matched to within $5\times 10^{-7}/\mbox{K}$. The overall thermoelastic constant of the sensor is however difficult to predict, as it depends on integration asymmetries during assembly.

Outside of the sensor there will be a thermoelastic response due to temperature gradients within the SP enclosures. The largest temperature gradients are across the low-conductivity thermal pathways used to attenuate the temperature variation at the sensor die, with a targeted thermal time constant of 200 s. Again, the resulting thermoelastic response is difficult to predict as it depends on non-nominal asymmetries in the thermal pathways, though it is expected to be proportional to the difference between the sensor and enclosure temperatures.

A simple lumped-element thermal model of the SPs can be constructed to quantify the thermal response (Stott et al. 2018). One node is the sensor, with a temperature $T$_sensor_ measured from the resistance of a gold element on the frame of the proof-mass die. This node has a heat capacitance $C$_sensor_. The second node is the SP enclosure, which is mechanically and thermally connected to LVL. This node’s temperature, $T_{\mathrm{enc}}$, is determined from a calibrated platinum resistance thermometer attached to the inside of the enclosure. Between the two nodes we model the thermal isolation pathways as a thermal resistance $R$_sensor_ to give a thermal time constant for conduction to the die of $\tau_{\mathrm{sensor}} = R_{\mathrm{sensor}}C_{\mathrm{sensor}}$.

The thermal acceleration signal for each SP can then be calculated and removed from the data as

9 $$ \alpha _{\mathrm{thermal}} = \alpha _{\mathrm{sensor}} T_{\mathrm{sensor}} + \alpha_{\mathrm{enclosure}} ( T_{\mathrm{sensor}} - T_{\mathrm{enc}}), $$

where $\alpha _{\mathrm{sensor}}$ and $\alpha _{\mathrm{enclosure}}$ are the thermal response of the sensor and enclosure respectively. To determine the model parameters and calibrate the die temperature outputs, the flight SP units were logged over a controlled thermal cycle and multiple regression performed on the results. In addition, this test allowed a calibration of the mass position signal. The results are shown in Table 12 together with the time constant of the enclosures. The correlation coefficient to the model was very high for SP1 and SP2, but the results for SP3 were poor due to a subsequently identified failure in the tether between the sensors and electronics used in this test. The completeness of the thermal model was assessed by repeating the multiple regression with a further node at an external temperature reference. Inclusion of this node did not increase the correlation significantly.

**Table 12:** Temperature sensibility parameters of the SPs. Those of the SP3 are to be determined (TBD)

	SP1	SP2	SP3	
$\alpha_{\mathrm{sensor}}$	(−0.9 ± 2.8)×10^−6^	(−3.7 ± 0.2)×10^−5^	TBD	m/s^2^/K
$\alpha_{\mathrm{enclosure}}$	(−3.8 ± 0.2)×10^−4^	(1.2 ± 0.1)×10^−4^	TBD	m/s^2^/K
$\tau_{\mathrm{enclosure}}$	467	444	436	s
MPOS gain	51.4	51.5	44.7	V/(m/s^2^)

Table 12 shows that the sensor thermal sensitivity requirement is met, both for the uncorrected SP output and after regression of the SP output against temperature, where the residue is set by the uncertainty in the sensitivity. The thermoelastic sensitivity is not an SP requirement, but the effect at 0.1 Hz can be estimated using the SEIS model value of the external temperature noise under the WTS of $3\times 10^{-6}~\mbox{K}/\sqrt{\mbox{Hz}}$. This gives a thermoelastic noise injection of $3\times 10^{-9}~\mbox{m}/\mbox{s}^{2}/\sqrt{\mbox{Hz}}$ for SP1 and $5\times 10^{-9}~\mbox{m}/\mbox{s}^{2}/\sqrt{\mbox{Hz}}$ for SP2, both a factor of 2 or more below the SP noise requirement at this frequency.

This thermal analysis performed at CNES can be repeated during the calibration phase of SEIS on Mars to determine a revised, or in the case of SP3, new thermal sensitivities for each unit. These new values will incorporate any additional injection from the thermoelastic response of LVL.

#### SP Magnetic Response

The SP magnetic response should be very low. Silicon is a diamagnetic material and so the suspension should show no effect from any changing magnetic field. Although SP does use an electromagnetic actuator, the geometry of the magnetic circuit ensures that the coils are not sensitive to any change in an external field—the forces on the two sides of the coil will be common-mode rejected if there is no gradient in the field along the sensitive direction of the SP unit.

To confirm this, an SP sensor unit was placed into a magnetic test coil and the response to a 1.5 mT change in field recorded. The resulting highest sensitivity in any orientation was determined as $0.15~\mbox{m}/\mbox{s}^{2}/\mbox{T}$ compared to a requirement of $1~\mbox{m}/\mbox{s}^{2}/\mbox{T}$. This sensitivity is likely to be an overestimate as the magnetic-field inhomogeneity rather than its absolute value are likely to have produced a response of SP.

### LVL and Tiltmeters

#### Leveling System Overview

The LVL (Fig. 18) has a dual purpose: It will ensure level placement of the SEIS sensors on the Martian ground under as yet unknown local conditions of ground slopes up to 15° in any direction, a requirement that needs to be fulfilled for proper operation of the highly sensitive VBB seismometer and provide the mechanical coupling of the SEIS sensor assembly to the Martian ground. The LVL subsystem consists of a mechanical part, the leveling structure and an electrical part, the Motor Driver Electronics (MDE) board.

The structural ring of the LVL subsystem is the central interface to the VBB and SP seismic sensors and their proximity electronics, to the dampers with their interface to the lander deck during cruise and to the RWEB thermal enclosure. With the three extendable legs, namely the Linear Actuators, the LVL structure also provides the signal path from the Martian ground to the seismic sensors.

#### Linear Actuator Legs and Feet

The linear actuator is designed and developed as a separate unit which is assembled and tested alone and later integrated as a part onto the LVL structure. The linear actuator housing and the foot are made of titanium grade 5 (Ti6Al4V); the housing is gold plated to decrease the thermal emissivity. The telescopic leg is made of Invar with TiN coating. To protect the mechanism against dust and to maintain the thermal environment, the telescopic leg is covered with bellows underneath the SEIS sensor assembly. The mass of one linear actuator is $\sim 350~\mbox{g}$ without bellows and foot.

**Fig. 54:**
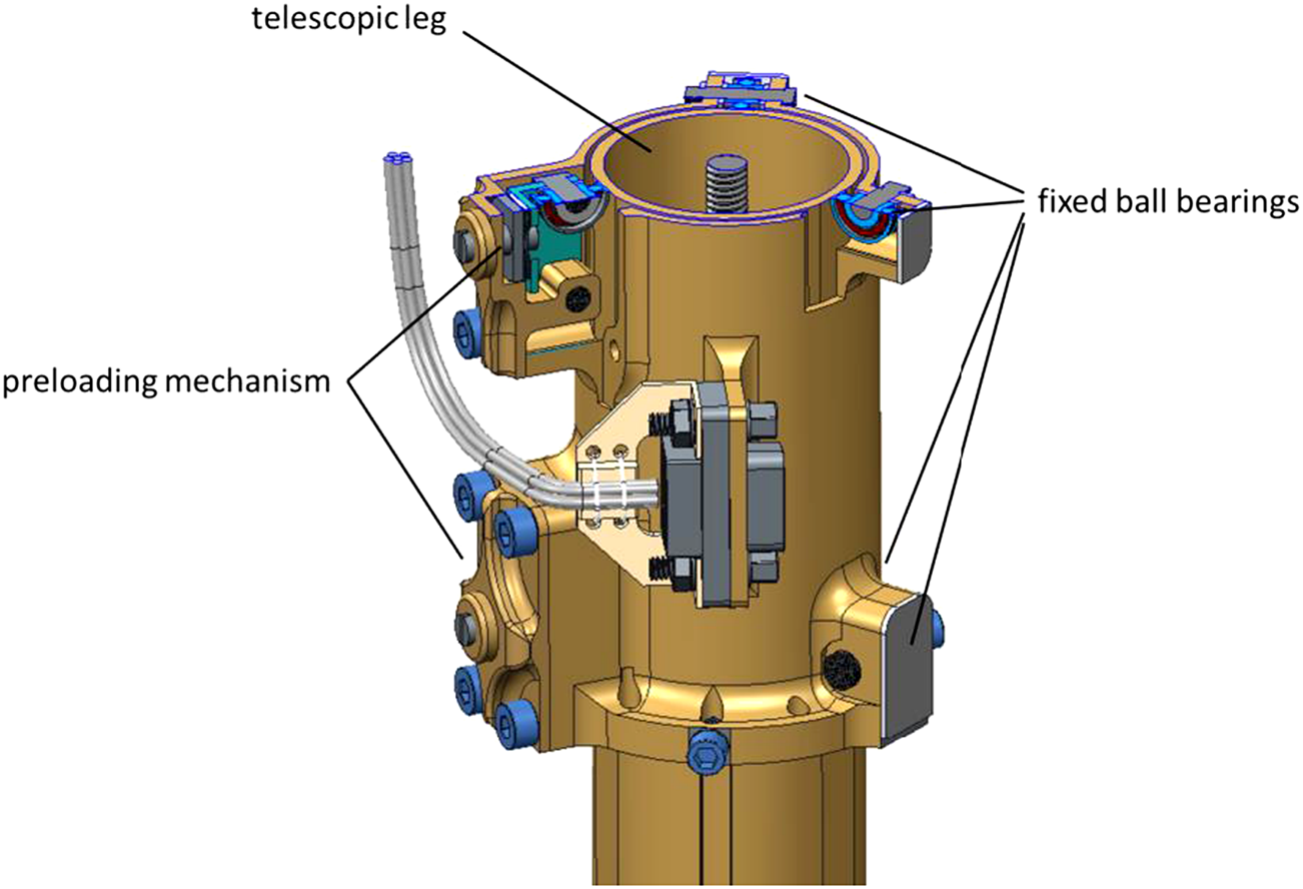
Linear guidance of the telescopic leg. The diameter of the telescopic length is 25 mm

The two main purposes of the three identical linear actuators in the LVL subsystem are the ability to level the SEIS sensor assembly from inclinations up to 15° by independently extending or retracting their telescopic legs and to transmit the seismic movements from the ground to the seismic sensors. An unbiased transmission of seismic motion is only possible with the first eigenfrequency of the sensor assembly much higher than the bandwidth for the measurements. This leads to a stiffness requirement for the extendable legs of the linear actuator which defines the geometrical shape and the guidance of the movable leg in the housing (Fig. 54). The stiffness has by system design a maximum value when the telescopic leg is mostly retracted; the stiffness decreases with extending the leg.

**Fig. 55:**
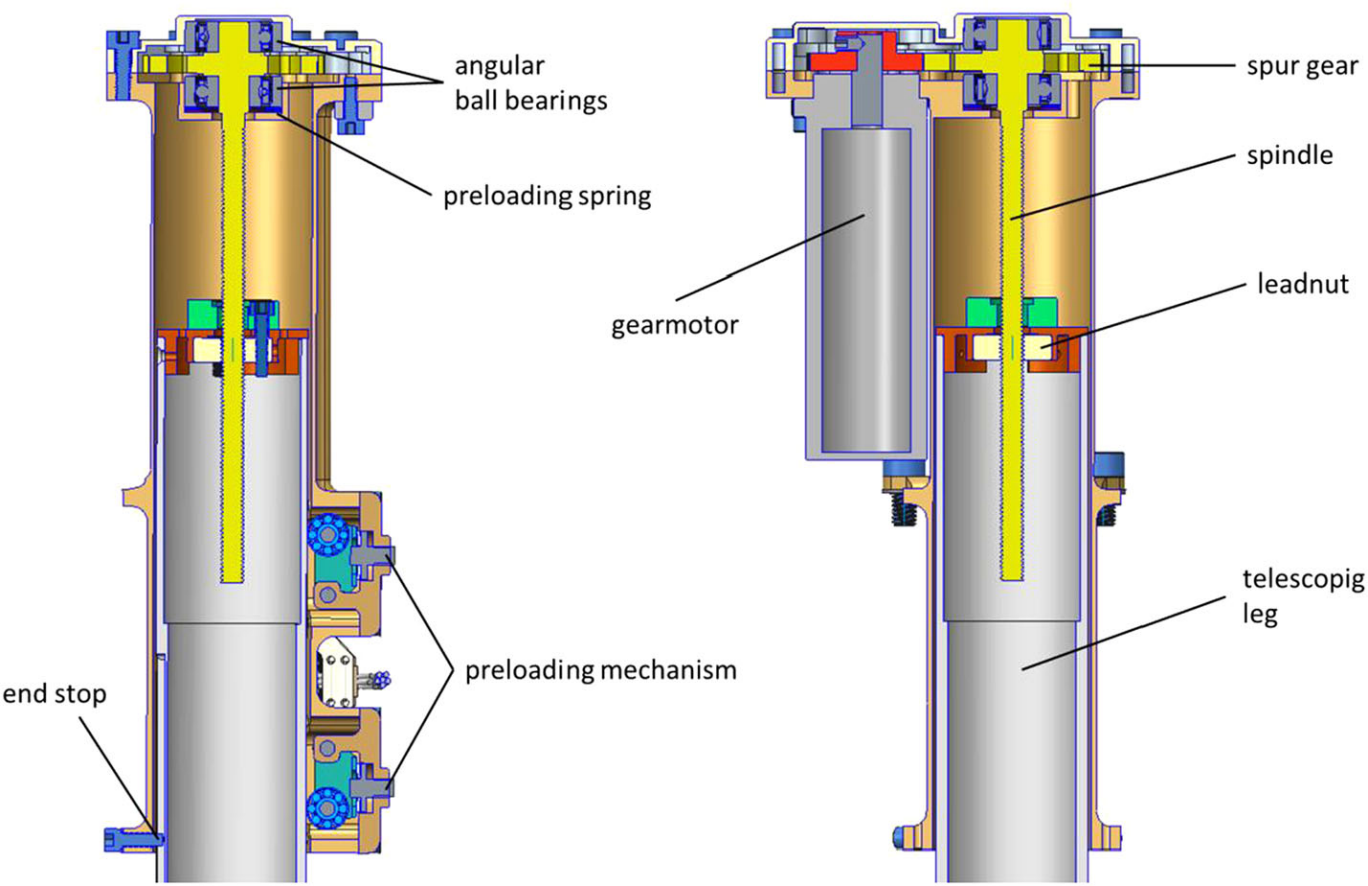
Geartrain of the linear actuator. The diameter of the telescopic legs is 25 mm

The diameter of the telescopic leg was selected to 25 mm; the round shape provides by its geometry the most efficient flexural stiffness for the part itself. A linear guidance off the shelf could not be used for various reasons like mass, materials and CTE mismatch, surface quality, etc. The solution is a preloaded guidance based on two systems of three ball bearings positioned in a 120° angle around the telescopic leg. Two ball bearings are on fixed positions in the stiffened housing; the third ball bearing presses the telescopic leg with an adjustable preload against the two other bearings (Fig. 55). One system is located close to the lower end of the linear actuator housing; the other system at a distance of 45 mm, which is close to the upper rim of the LVL structural ring. The linear actuator is mounted at the bottom and the top of the LVL ring; both fixations are close to the two guidance systems to maintain the stiffness path towards the structural ring and the seismic sensors.

The design of the mechanism for the linear movement is driven by geometrical requirements. With the effective diameter of the LVL structural ring of 250 mm, the required travel for a compensation of 15° inclination is 59 mm for each linear actuator. Due to the volume envelope of the sensor assembly, the gearmotor has to be mounted beside the housing with a spur gearhead on top. Mechanical end stops on the telescopic leg keep the moving part in place.

The motor is a Phytron two-phase stepper motor with a 48:1 planetary gearhead. The spur gear head has a ratio of $58/38$; the spindle has a pitch of 0.7 mm. With the 200 steps per motor turn, this results in a theoretical linear resolution of $\sim 50~\mbox{nm}$ displacement per motor step. The spindle gearhead and spindle is made in one piece. Two angular ball bearings with a preload against the top of the housing are fixing the spindle in $z$-direction while keeping the stiffness path when the SEIS sensor assembly is standing on the ground. The material of the spindle is also titanium grade 5 to match the CTE. The leadnut is made of gearbronze. The system is dry lubricated with a MoS_2_ coating on the spindle.

The LVL feet need to provide a stable contact and good coupling between the SEIS sensor assembly and the Martian surface at the landing site, where a regolith cover composed of fine basaltic sand with low rock abundance is expected (Golombek et al. 2017). As cone-shaped feet, which are commonly used for terrestrial seismometers, can result in uncontrolled sinking if deployed on a non-rigid surface, a round metal disk of 60 mm diameter was added to the upper end of each foot.

The optimum dimensions of the foot cone were determined by dedicated measurements at the Ecole des Ponts ParisTech, using a specially developed penetration device. Measurements were performed on Mojave simulant provided by JPL and chosen as a Mars analogue of the surface materials. See Delage et al. (2017) and Fayon et al. (2018) for more details. This is a mix of MMS simulant, containing alluvial sedimentary and igneous grains from the Mojave Desert, with basaltic pumice, sieved at 2 mm. A series of preliminary tests with foot cones of 20 mm length and maximum diameters of 20 mm and 30 mm showed that full penetration could not be achieved under the maximum force of 10 N. Therefore, two alternative cones with a smaller maximum diameter of 10 mm and lengths of 10 mm and 20 mm were designed that achieved full penetration. In addition, some plate loading tests, using just the disk without any cone attached, were conducted.

After complete penetration, repeated elastic loading cycles between 10 N and 8 N were performed for samples of the Mojave simulant with densities of $1640~\mbox{kg}/\mbox{m}^{3}$ and $1670~\mbox{kg}/\mbox{m}^{3}$. For the lower density, the plate loading test and the test with the 20 mm cone result in similar values of stiffness. In the denser sand, the response with the cone is softer than that of the plate alone, as stiffness increases markedly with higher sand density for plate loading but does not show a similar effect for the cone test. The stiffness values for the 20 mm cone are about 50% larger than for the 10 mm cone and stiffness values obtained with both cones increase progressively during consecutive load cycles (Fayon et al. 2018). Consequently, a foot cone of 20 mm length at 10 mm maximum diameter was selected, together with an 60 mm diameter disk which will ensure ground contact also for tilted configurations (Fayon et al. 2018).

#### Structural Ring and Sensors

The LVL structural ring is a complex interface part made in one piece of titanium grade 5 (Ti6Al4V). It is gold plated to decrease the thermal emissivity. On the inner side of the ring, three foil heaters are mounted to maintain a sufficiently warm environment for the seismic sensors during winter times on Mars. The two SCIT (SCIentific Temperature) sensors are also mounted on the ring.

Two types of tiltmeter are installed: A two-axes MEMS sensor for the coarse-leveling and two single-axis high precision electrolytic tiltmeters (HP tiltmeter) for fine leveling. The MEMS is oriented towards the reference coordinate system of the SEIS sensor assembly. Inside the MEMS, a small proof mass is hanging in springs; the position of the proof mass is a measure of tilt. The output signal depends on the gravity; together with the MDE, an inclination of ∼±25° can be measured on Earth and ±90° on Mars with a resolution better than 0.1°.

The two electrolytic tiltmeters are oriented in the movement direction of two Linear Actuators. They have a measurement range of $\pm 720~\mbox{arcsec}$, dependent on the sensor temperature. Inside the electrolytic tiltmeter, a small amount of liquid is filled in a hermetic sealed geometry like a water level. The liquid is conductive. Three inserted electrodes form a voltage divider with the liquid as inclination dependent resistances. The output amplitude is a measure of the tilt. To avoid electrolysis and corrosion, the tiltmeter is powered with AC voltage. Two dedicated sensor front end electronics pre-process the sensor signals. These electronics PCBs are mounted on the LVL structural ring close to the HP tiltmeter, underneath two SP sensors. The theoretical resolution is better than 1 arcsec.

#### MDE Board

**Fig. 56:**
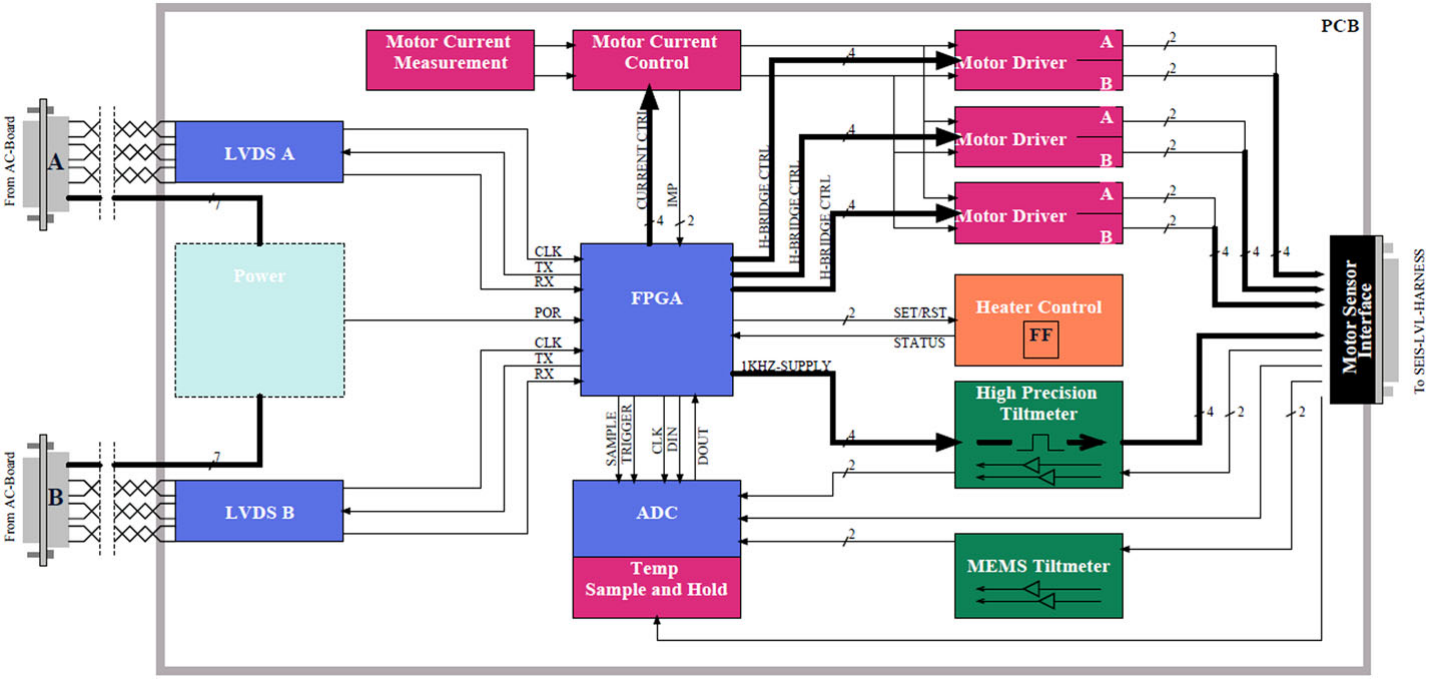
MDE block diagram

The MDE controls the levelling system and its block diagram is shown on Fig. 56. It operates the motors of the linear actuator, acquires the signals from the MEMS and high precision tiltmeters and switches the winter mode heater on the LVL ring. The PCB is mounted into the SEIS E-Box. The connection between MDE and LVL is realized with a dedicated LVL tether.

In deviation from the general E-Box architecture, the MDE is a single system without redundancy. Nevertheless, it receives power and data from two sides. The power lines are cross-strapped in hardware; the data interfaces are merged in the FPGA. The MDE acts as a slave, i.e. it only communicates and operates the LVL when being asked to. With a serial three-byte protocol, all functions can be commanded; the MDE answers on each command with an acknowledge pattern and the requested data. There is no autonomous functionality implemented except hardware safety features like short circuit or over-temperature protection of the motor lines.

The motor controller is a current controlled bipolar stepper motor driver with free programmable start speed, ramping and total number of steps for the three motors. A minimum of 12 motor steps can be commanded, resulting in a minimum displacement of $\sim 0.6~\upmu\mbox{m}$ of the Linear Actuator. A maximum displacement of $\sim 12.6~\mbox{mm}$ can be commanded in one motor run.

A common 2-phase current controller is switched to one of three full bridge motor drivers. Consequently, only one motor can be operated at a time. The current controller has four fixed current steps: 100 mA, 125 mA, 150 mA (nominal motor current), 180 mA (boost motor current). It is a free running switching controller using the motor inductance in buck converter architecture. A supervisor circuit processes the on/off signals of the current control and determines nominal and fault conditions. For a short circuit, i.e. the current rises too fast, the motor control immediately stops and an error flag is raised. From the duty cycle of the current controller at constant current, the motor temperature can be derived as a function of the resistance of the motor coil. This information is used in the MDE to protect the motor against over-temperature.

Each motor can be operated in half step, full step or in pre-heating mode. The pre-heating of the motor is realized with a full step mode without phase shift of the two motor currents. Heat is generated in the motor coil, but the motor does not move.

On the LVL ring, the MEMS and high precision tiltmeter provide analogue signals of the tilt in X- and Y-direction. The data are sampled and digitized using a 12 bits AD converter. The sampling rate is limited by the communication interface where only one channel can be transmitted within one command. As the high precision tiltmeter is a device requiring an AC excitation, a square wave signal of 2 Vpp 500 Hz is generated on the MDE and provided to the sensors. A synchronous rectifier is located on the sensor front end electronics mounted on the LVL ring. The high precision tilt information is transmitted back via the LVL tether to the MDE as a DC value.

Heaters are installed on the LVL structure with a total power of $\sim 1.5~\mbox{W}$ to keep the seismometer warm during winter time. The heaters are powered from the MDE board from a 6 V supply. They are not actively controlled, only commanded on and off via the MDE FPGA. To save power during winter when the other levelling functions are not required, the MDE can be partly switched off. The heater control remains active in the winter power mode and keeps the heater on.

### Ebox

#### Overview

**Fig. 57:**
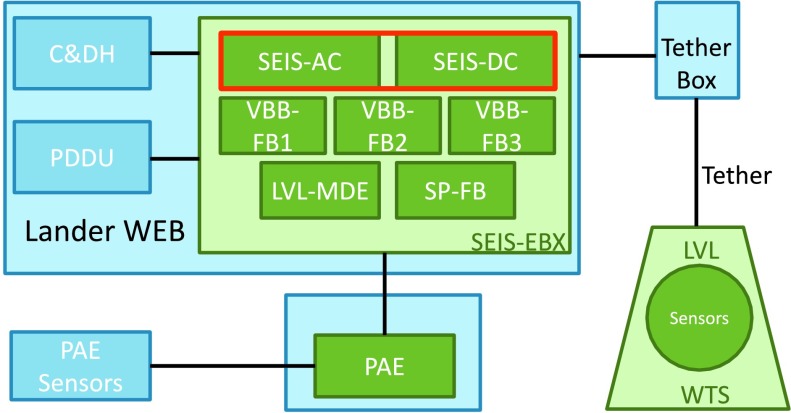
The place of the E-Box in the system

The E-Box (see Fig. 19 and Fig. 20) contains the main part of the electronics for SEIS and resides in the lander warm electronics box (WEB). Figure 57 shows the place of the E-Box in the system. The E-Box is controlled by the Command and Data Handling (C&DH) and supplied by the Power Distribution and Drive Unit (PDDU), which are both part of the lander.

The design and production of the electronics in the red boxed part and the top level integration of SEIS-EBX has been conducted under the auspices of ETH Zurich. The blocks VBB-FB[123] are part of the VBB sensors, the block SP-FB is part of the SP sensors and the block LVL-MDE is part of the leveling system. The description in this section mainly focuses on the functions included in the SEIS-AC and the SEIS-DC, i.e. the red boxed part.

This electronic must withstand the harsh environment during cruise to and operations on Mars. To overcome adverse effects due to radiation, vacuum and temperature variations, only space-qualified components can be used and dedicated design techniques are needed. These techniques include latch-up protection for analog circuits and an FPGA design with implementation of Triple Mode redundancy (TMR) for flip-flops, safe state machines and Error Detection and Correction (EDAC) for memories.

On top of that the electronics is made fully cold spare redundant. The only exception is made for the signal conditioning and analog to digital conversion for the seismic signals from the VBB and SP sensors neither of which is redundant.

#### Functionality

##### Data Acquisition

**Fig. 58:**
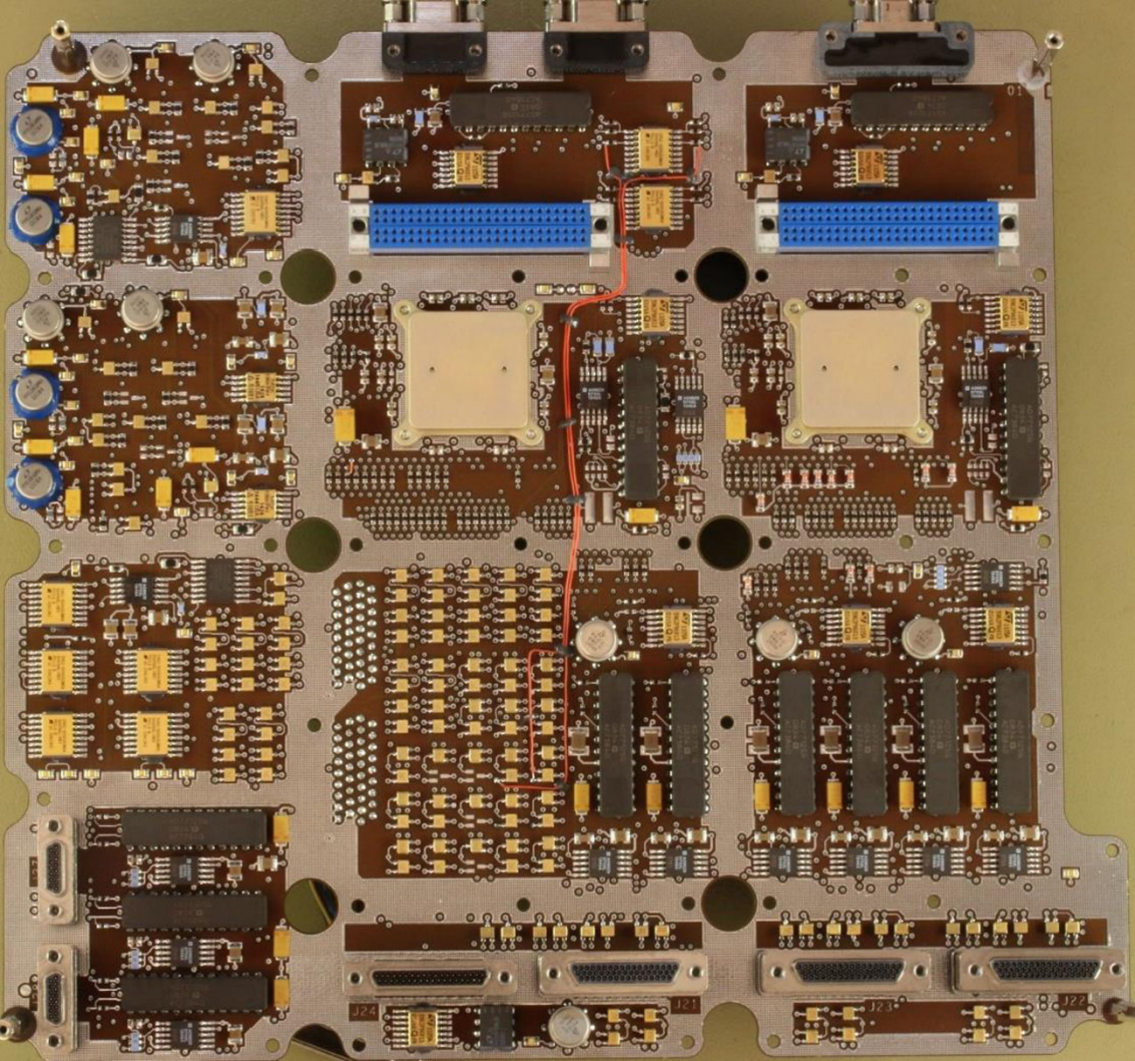
The SEIS-AC acquisition electronics including redundancy

The E-Box acquires data by digitizing analog signals, which is stored in the on-board non-volatile memory. This function is made by the SEIS-AC card (Fig. 58). Up to 65 hours of data can be stored. The lander computer is able to gather that data when it is active.

There are 9 seismic channels, from the VBB and SP sensors and the scientific temperature (SCIT), each acquired as continuous signal by a dedicated sigma-delta ADC (AD7712). These channels are digitized at a rate of 32 kHz using the ADC filters to output 24-bits samples at 500 Hz. Further digital filtering is used on SEIS-AC to reduce the sample rate to the chosen output sampling rate of these channels.

Unlike the seismic channels, the remaining channels are acquired as samples by two ADCs that are used for multiple channels and have a multiplexer in front of them. These remaining channels comprise the 3 VBB temperatures and 48 housekeeping (HK) channels.

The 3 VBB temperatures are acquired using one sigma-delta ADC (AD7712) that delivers 16-bits samples using the filters inside the ADC itself. A sample is composed of the average of 16 consecutive samples taken at 100 Hz by this ADC, which is stored as a 16-bit value. SEIS electronics measure resistivity for the Temperature sensors with a Full Scale Range of 896.25 $\Omega $ (231.11 $\Omega $ to 1127.36 $\Omega $). The Transfer function of the sensors in °C/DU is provided in Sect. 6.4.1.

The 48 housekeeping channels are acquired using one rather fast successive-approximation ADC (ADC128S102QML) that delivers 12-bit samples. For each of the 48 channels a sample is composed of the sum of 16 samples taken at $194.3~\upmu\mbox{s}$ intervals by the ADC, which is stored as a 16-bit value. Between the channels there is a delay of 1.166 ms to allow for the input multiplexer to switch and the input of the ADC to settle. These housekeeping channels include 1 dummy channel and measured voltages, currents and temperatures of the instrument.

##### Digital Filtering

**Fig. 59:**
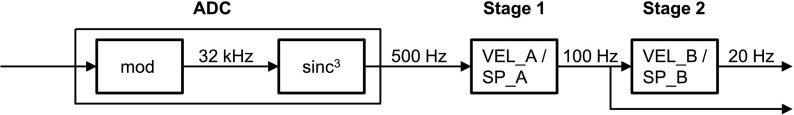
Digital filtering in acquisition chain for velocity signals

The 9 seismic channels and the scientific temperature are acquired as continuous signals and therefore digital filters are used to reduce the sample rate. The sample rate is reduced in order to lower the data volume to be stored in the non-volatile memory without losing information that is in the bandwidth of interest. The filters remove high frequency components of the signal that would have aliases in the pass band if just decimation would be applied. The filters following the ADC have a suppression of at least 120 dB for frequencies out of the pass band, before decimation takes place. Figure 59 shows the part of the acquisition chain incorporating digital filtering for the velocity channels.

**Fig. 60:**
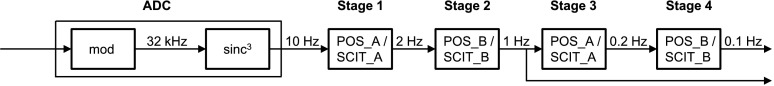
Digital filtering in acquisition chain for position and SCIT signals

Different filters are implemented for the position channels and scientific temperature, as they have a lower frequency of interest. Figure 60 shows the digital filtering for the low frequency channels.

The filter in the ADC is a 3rd order sinc-in-time filter. The remaining stages are FIR filters in the FPGA that is part of the SEIS-AC. This FPGA can be configured to store the data at two sample rates. For velocity channels the data are stored at a sample rate of either 100 Hz, out of stage 1, or 20 Hz, out of stage 2. For position and SCIT channels the data are stored at a sample rate of either 1 Hz, out of stage 2, or 0.1 Hz, out of stage 4. In cases where both sample rates are needed, the E-Box is configured to store the higher sample rate and the lower sample rate is reproduced in the SEIS-FSW by an exact copy of the filter present in the E-Box.

The coefficients for the FIR filters in the FPGA are stored in the non-volatile memory of the E-Box. Commands are available to upload 8 different sets of coefficients. Each one can hold up to 256 factors. The sets of coefficients identified as VEL_A and VEL_B are used in stage 1 and 2 respectively for the VBB velocity channels and the sets SP_A and SP_B are used in stage 1 and 2 respectively for the SP velocity channels. For the position and SCIT channels two stages are used to decimate by a factor of 10, because the two stages use less resources than a single stage would. This also takes two sets of coefficients, which are POS_A and POS_B for the VBB position channels and SCIT_A and SCIT_B for the SCIT channel. Thus, stage 1 and 3 of the position and SCIT channels share the same set of coefficients and so do stage 2 and 4 of these channels.

The FIR filters are loaded with symmetric coefficient sequences to make them linear phase filters, i.e. there is a constant delay for all frequencies in the pass band. The amount of this delay depends on what coefficients are uploaded, but naturally the delay of the position and SCIT channels is much more than that of the velocity channels.

##### Temperature Offset Compensation

For all temperatures but SCIT an offset compensation is implemented in the FPGA in order to reduce measurement error related to the electronics. For the SCIT a precise current source is implemented, together with a 4-wire measurement. Hence, for the SCIT this compensation is not needed. For the VBB temperatures and the housekeeping temperature channels a precise $1~\mbox{k}\Omega$ resistor is measured in addition to the temperature sensors, using the same acquisition electronics. There are separate acquisition electronics for the VBB temperatures and the housekeeping channels and thus separate precise $1~\mbox{k}\Omega$ resistors for both.

The value measured on the precise $1~\mbox{k}\Omega$ resistor is then used to perform the compensation by the following formula:

10 $$ R_{\mathrm{T\_COMP}} = R_{\mathrm{T\_MEAS}} + (R_{\mathrm{1k\_REF}} - R_{\mathrm{1k\_MEAS}}) $$

This compensation is done on the digital values in the FPGA, i.e. before they are converted to physical values. The value $R_{\mathrm{T\_MEAS}}$ is what is acquired on the temperature sensor, the value $R_{\mathrm{1k\_MEAS}}$ is measured on the precise $1~\mbox{k}\Omega$ resistor and $R_{\mathrm{1k\_REF}}$ is a constant that holds the digital value that converts to $1~\mbox{k}\Omega$. The calculated $R_{\mathrm{T\_COMP}}$ is the offset compensated value that is normally stored in the data packets.

In case the value measured on the precise $1~\mbox{k}\Omega$ resistor is not in the range of $R_{\mathrm{1k\_REF}} \pm 6.25\%$, the compensation is not applied, $R_{\mathrm{T\_MEAS}}$ is stored in the data packets and the corresponding data invalid flag residing in the science packet is raised. It is implemented this way to avoid that a malicious acquisition of the precise $1~\mbox{k}\Omega$ resistor leads to corrupted data on the corresponding temperature channels.

**Fig. 61:**
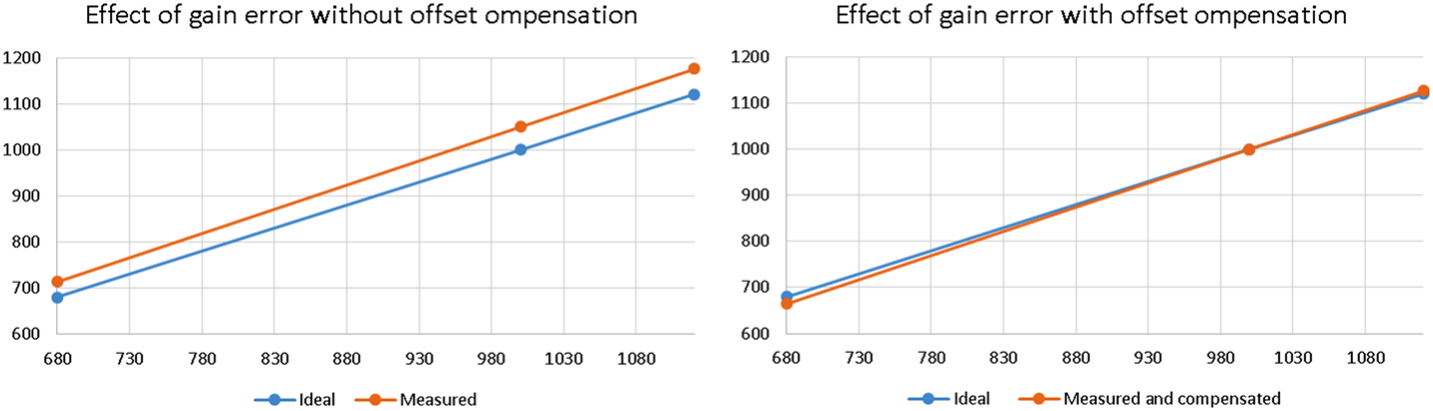
The reduction of gain error by offset compensation for temperatures

This offset compensation cancels the measurement error due to offset in the electronics and resistances in the multiplexers and leads that are common between the sensor and the precise $1~\mbox{k}\Omega$ resistor. Variations in the sense current, that constitute as a gain error, are not fully canceled by the offset compensation, but still reduced. Figure 61 shows the reduction of the gain error by the offset compensation for the VBB temperatures. The gain error is exaggerated (5%) in this figure for illustrative reasons.

##### Data Management

The data of the seismic channels and the VBB temperature channels is packed in chunks that contain the samples acquired during 1 s. This data, together with a time stamp and a header that includes also the SEIS status flags, will always fit in one page of the non-volatile memory, regardless of the selected sample rates. If no channels are acquired, only the status flags are stored. Thus, there is always one page of the non-volatile memory written each second the SEIS is active.

A different area of the non-volatile memory is used for storing the housekeeping (HK) data. In contrary to earth seismometer for which the only acquired channels are the velocity and mass position outputs, the health of SEIS will indeed be monitored through the recording of the different voltage supplies and not only those delivered by the lander but also those provided by the SEIS DC/DC (see Sect. 4.1.4) to the SEIS sub-systems, as well as through recording of these subsystem temperatures.

A sample for each of these channels is acquired by performing a scan over all the channels. A scan over all the channels takes less than 1 s to finish, regardless of the selected data rate. The start of this scan is triggered on an internal 1 Hz signal and it can be configured to store the result of the scan every 1 s, every 100 s or not at all. Other rates of housekeeping data may be achieved by decimating the data from the E-Box, e.g. by the SEIS-FSW, which is a process happening outside the E-Box.

The way the housekeeping data are stored depends on the rate at which the data are to be stored. If there is data every 100 s, the data of a single scan is stored together with a time stamp and a header in one page of the non-volatile memory. If there is data every 1 s, the data of 10 scans is collected and stored with a time stamp per scan and a header in one page of the non-volatile memory. The reason to store 10 scans together in one page is that the memory capacity reserved for housekeeping data would not be sufficient if one page is used every 1 s. On the other hand, if 10 scans are to be collected that are 100 s apart, housekeeping data of up to 900 s is not stored in the non-volatile memory at a time. If the instrument is shut down by the fault protection, housekeeping data of up to 900 s may be lost then, which may hamper the investigation of what has happened. Hence, there are different strategies how to store the data for the different data rates.

**Fig. 62:**
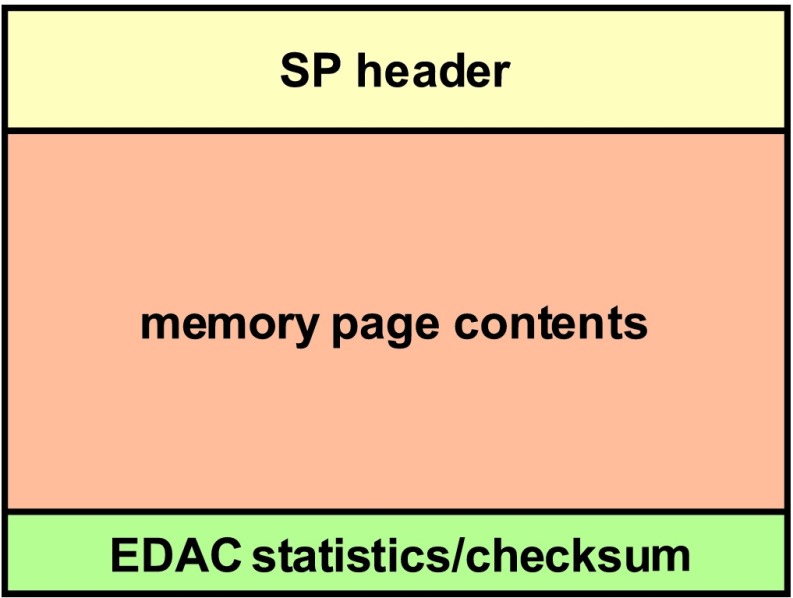
The structure of packets containing seismic or housekeeping data

The data stored in the pages of the non-volatile memory is transferred to the lander computer, when a request is received to do so. If the lander computer requests packets with seismic or housekeeping data, the E-Box creates exactly one packet from each memory page. Figure 62 shows the structure for these packets.

The SP header provides a “marker” between the instrument data records and defines what type of packet is transferred, the memory page contents form a self-contained body of the packet and at the end there are the EDAC statistics and a checksum. The EDAC statistics comprise of a count of single errors, which are corrected and double errors, which cannot be corrected. The checksum at the end is used on the lander computer to detect transmission errors.

**Fig. 63:**
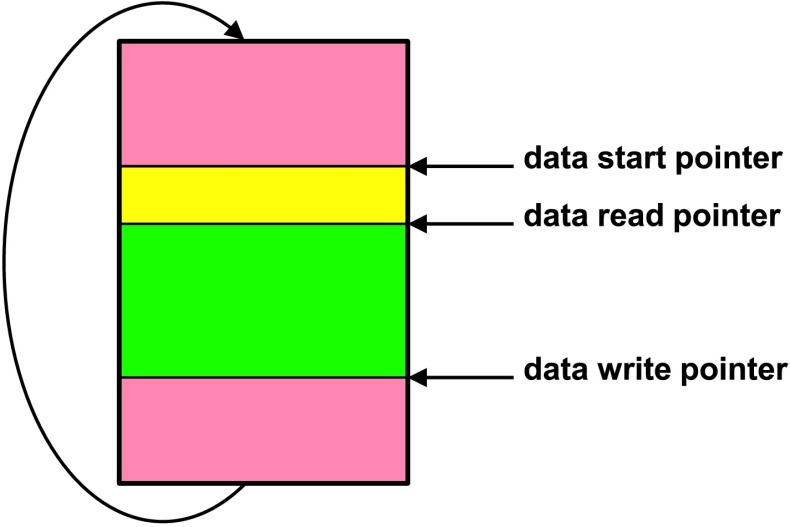
The circular buffer as used for seismic and housekeeping data

The memory areas for the seismic and housekeeping data are managed as circular buffers. Packets are transferred to the lander computer in the same order as they are stored in the E-Box. Figure 63 shows such a buffer and the pointers used to manage the data.

The area colored red is the part of the memory that is not in use and may contain old data. The areas colored yellow and green contain data stored in the E-Box that can be retrieved by the lander computer. The data start pointer is set to the page that contains the oldest data and the data write pointer is set to the first page where new data can be written. When a new packet is stored, it is stored where the data write pointer is set to and then this pointer is advanced. Data are deleted by advancing the data start pointer. If any of the pointers reaches the end of the area, then the pointer is moved to the start of the area when advanced.

There is also a read pointer that is set to the first page that is not yet read. At the start of a packet transfer, the data read pointer is made equal to the data start pointer and the read pointer is advanced each time a page is read. The read finishes when all requested packets are transferred or if the data read pointer has become equal to the data write pointer. The data read pointer is also used to delete data, as this is safer. When an amount of packets is requested to be read, less packets may be available. If after this the same amount would be requested to be deleted, a packet that has been stored in the meantime would be deleted without ever have been read. Therefore, there is a command to delete the packets that having been read, which causes the data start pointer to be set equal to the data read pointer. Thus, with this command the yellow colored area would be deleted, i.e. become memory that is not in use.

##### Time Stamps and Synchronization

The SEIS instrument keeps its own time, which is independent of the lander computer. The SEIS time is called LOBT (Local On-Board Time). The time is kept by a 40-bit counter that counts $1/1024~\mbox{s}$ ($2^{-10}~\mbox{s}$) ticks and can count more than 34 years. This LOBT is used to provide the time-stamps for the data that is stored in the E-Box.

The data gets its time-stamp at the moment it is stored. Only one time-stamp is provided for the data of all channels of a 1 s time frame. The data that becomes available in a second gets a time-stamp that is set to the start of that second. So, for the VBB temperatures and the housekeeping channels the data are acquired at a time that is later than what the time-stamp is set to, i.e. in the 1 second period that started at the time put in the time-stamp.

For the seismic channels and the scientific temperature channel the output data of the digital filters is stored. The group delays of the filters are not compensated, i.e. the signal is first delayed and then a time-stamp applied. That means that the actual signal is acquired before the time that is put in the time-stamp. For velocity channels there are multiple samples in a 1 s time frame. The time-stamp applies to the first sample and each subsequent sample is taken one sample period later.

It is chosen to supply only one time-stamp to reduce the amount of data to be transferred. The difference between the time the signals are actually acquired and the time-stamp applied is constant and known, thus a single time-stamp supplies all information needed. To rearrange the data such that the packet has data that is actually acquired in the same 1 s frame, would need temporary storage of the signals with no or little delay. The group delay of the position signals at 0.1 Hz is several minutes, in which a lot of velocity data are acquired. The memory capacity of the FPGA used is not sufficient and external memory would cause an increase in power consumption and volume. Hence, it is chosen to have this resolved in the SEIS-FSW, in which the data are rearranged and further processed anyway.

The SEIS LOBT may drift a little with respect to the time kept in the lander, which is called SCT (SpaceCraft Time). These times are not aligned, as it is chosen to have continuous data out of the E-Box without jumps in the time. Instead, time pairs of SCT and LOBT are generated, which contain both values measured at the same instant of time.

In order to correlate data of SEIS with data from APSS a 1PPS signal is supplied by SEIS E-Box to the PAE (Payload Auxiliary Electronics of APSS). Figure 57 shows this connection. A rising edge of the 1PPS signal supplied to the PAE coincides with the instance of time a time-stamp is generated on SEIS. The APSS supplies data that allows to determine when this rising edge occurred measured in APSS LOBT.

##### Several Control Functions

The E-Box SEIS-AC switches the power of the subsystems and provides several, mostly custom, interfaces with the other boards in the E-Box. This includes configuring the feedback electronics for the VBB sensors, on which different feedback modes and gains can be set. The status of this electronics can be read, which includes the electrical end stop detection of the mass re-centering mechanism. For the SP sensors the feedback electronics can be set to different gains, re-centering can be started and control signals are provided to switch the power of the 3 SP sensors separately. All these functions are made available through SEIS commands from the lander computer.

An asynchronous serial interface is supplied to the leveling motor driver electronics, allowing to operate the leveling motors, the heater and the tilt sensors by writing and reading registers in this electronic. The stepper motors in the VBB sensors for the thermal compensator and mass re-centering are controlled by SEIS-AC, which includes keeping record of their position. Power is supplied for the motors by SEIS-AC, which can be used for one motor at a time. The operation of the leveling motor driver electronics and the VBB stepper motors is also done by commands from the lander computer to SEIS.

A calibration waveform can be sent to the VBB and/or SP sensors. Calibration waveforms, one for the VBB sensors and one for the SP sensors, are stored in the non-volatile memory. A sensor calibration can be started during which the VBB mode and acquisition configuration is changed. After the waveform is output to the sensor for the requested amount of repetitions, normal operation mode is resumed automatically.

#### Performance

##### Signal Acquisition

For the acquisition of the science channels a 24 bit sigma-delta ADC (AD7712) has been chosen primarily for its excellent low frequency noise performance, its low power consumption and radiation robustness. Sigma-delta ADCs, like other integrating ADCs, do not contain any source of non-monotonicity and inherently offer no missing codes performance. The AD7712 achieves excellent linearity by the use of high quality, on-chip silicon dioxide capacitors. The device also achieves low input drift through the use of chopper stabilized techniques in its input stage, which thus greatly reduces the $1/f$ low frequency noise.

A space qualified external voltage reference (RH1021-2.5), with the temperature stability of $< 5~\mbox{ppm}/\mbox{K}$, is used to achieve good stability in the harsh temperature environment on Mars and has a low noise performance matching the ADC performance.

**Fig. 64:**
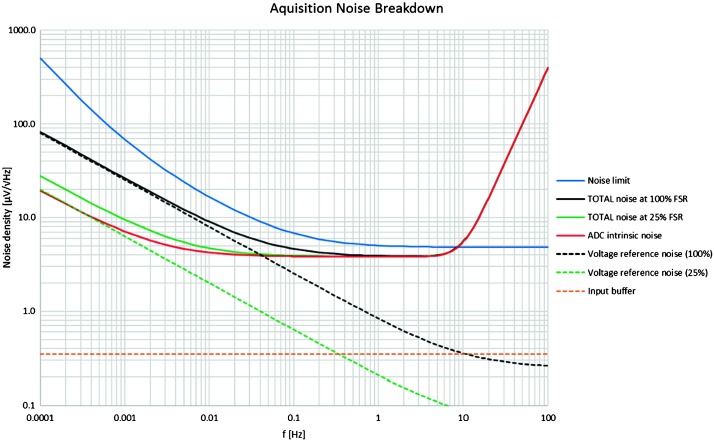
The acquisition noise breakdown of VBB VEL channels ($\mbox{FSR} = \pm 25~\mbox{V}$, $1~\mbox{LSB} = 3~\upmu\mbox{V}$)

Acquisition noise level at low frequency ($<100~\mbox{mHz}$) depends on input signal amplitude since the voltage reference noise is scaled with the acquired signal. For signals less than about 25% of the full scale range (FSR), the voltage reference noise is attenuated below the intrinsic noise of ADC (Fig. 64). Full scale range are $\pm 25~\mbox{Volt}$ for the VBBs (for both VEL and POS outputs). It is $\pm 12.5~\mbox{Volt}$ for the Velocity output of the SP and $\pm 5~\mbox{Volt}$ for the SP POS output. Full Scale Range of the TSCI is $1432.66~\Omega$ ($0~\Omega$ to $1432.66~\Omega $) with transfer function of the TSCI given in Sect. 6.4.1.

The ADC intrinsic noise of $3.8~\upmu\mbox{V}/\sqrt{\mbox{Hz}}$ is flat (white) down to 10 mHz, below which the ADC $1/f$ noise becomes visible. Beyond about 8 Hz the ADC quantization noise starts to dominate over the ADC white noise. The FPGA FIR filter will sharply attenuate this noise beyond 40 Hz (80% of the Nyquist frequency) for high 100 Hz output data rate selection. For low 20 Hz data rate, the filter will completely attenuate the quantization noise since the corner frequency is 8 Hz in this case.

### Tether, Tether Storage Box and Load Shunt Assembly

#### Overview

The Tether System has the task of bringing power and excitation waveforms from the Ebox in the Thermal Enclosure on the Lander to the Sensor Assembly deployed on the Martian ground and taking output voltages from the Sensor Assembly back to the Ebox for digitization and storage. It must provide this connectivity while also permitting the deployment of the Sensor Assembly, surviving the forces involved in deployment of the Sensor Assembly, surviving the Martian environment for at least 1 Mars year and after deployment, not exerting forces on the Sensor Assembly that would contaminate the seismic data. It is worth noting for comparison that a standard terrestrial seismometer (STS-2), which has all its analog feedback in the sensor assembly, has 18 conductors going from the sensor assembly to the data logger, while SEIS requires over 200 conductors between the sensor assembly and the Ebox in the Thermal Enclosure in the Lander. All VBBs and SP feedback cards are indeed located in the S/C warm box, in addition to all oscillators used by their displacement transducer.

**Fig. 65:**
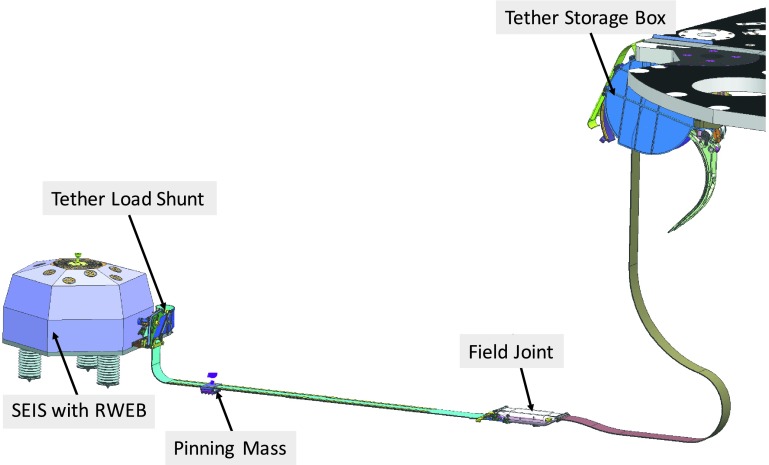
Tether System overview, Deployed Configuration. The TSA-3 and TSA-4 between the Tether Storage Box and the Ebox are unchanged from. The distance from the center of the Sensor Assembly of SEIS and the point below the Tether Storage Box is about 1.40 m in this configuration

The solution consists of the Tether itself, a Tether Storage Box (TSB) that holds the excess Tether up until deployment and allows the Tether to pay out during deployment and a Load Shunt Assembly (LSA) that is strong during deployment and provides significant mechanical decoupling of the Tether from the Sensor Assembly after deployment. The Tether System is shown in its stowed (Fig. 21) and deployed configurations in Figs. 65 (see Fig. 12 for picture during deployment tests).

#### Tether

**Fig. 66:**
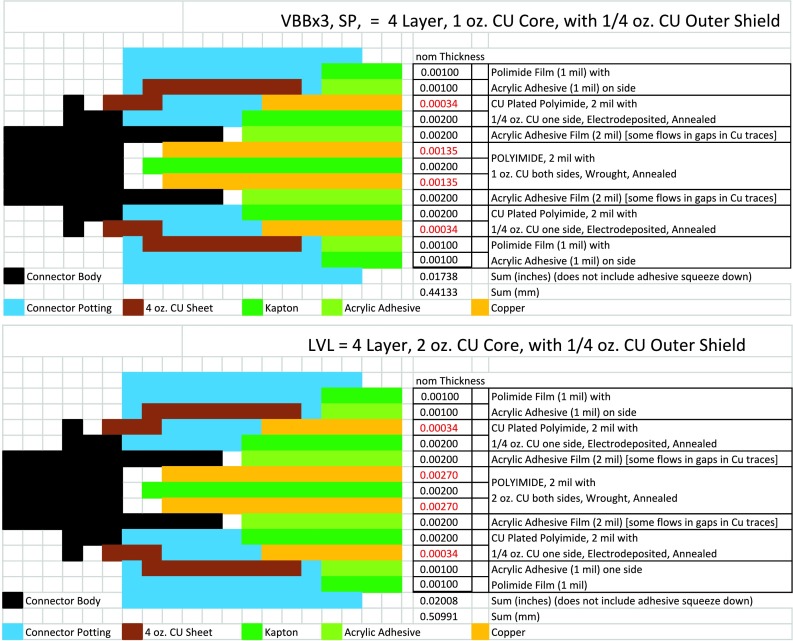
Construction and thicknesses of Tether belts

The tether comes in 4 segments, 3 of which consist of flat copper and Kapton belts (TSA-1, 2 and 3 in) and the remaining segment (TSA-4), which is constructed of a normal wiring bundle. Each belt in the tether is made of 5 layers of Kapton interleaved with 4 layers of copper bonded together with acrylic adhesive as shown in Fig. 66. This construction was chosen to minimize mass and because the belts are flexible in the out-of-plane direction, permitting deployment of the sensor assembly to the Martian ground.

There is a pinning mass attached to the tether just outside where the WTS wall crosses the tether (Fig. 12). The pinning mass is intended to anchor the tether and greatly attenuate thermoelastic and mechanical noise on the lander side from getting to the sensor assembly and also provides a hook whereby it is possible to adjust the geometry of the Load Shunt Assembly (LSA described below) after the Frangibolt has opened the LSA. The camera on the arm will image the LSA after it has opened to check the geometry. If the geometry is not as desired, the arm and scoop will be used to move the pinning mass by up to a few centimeters.

The field joint (see Fig. 14) permits removal and re-integration or connection of the Sensor Assembly during testing with specific Earth ground systems with minimal impact to the rest of the spacecraft.

#### Tether Storage Box

The Tether Storage Box resides under the deck of the Lander. It holds the excess tether during launch and cruise, allows some of the tether to be released during the Sensor Assembly deployment to the surface of Mars and then the bottom opens to deposit the remaining tether on the ground. During deployment, the tether is held above the ground to avoid snag hazards.

#### Load Shunt Assembly

The Load Shunt Assembly (LSA) was invented to isolate the sensor assembly from thermoelastic expansion and contraction in the tether. Standard terrestrial broad band seismometer installations often arrange the cable to the sensor head to loop around the sensor head or otherwise take a serpentine path leading into the sensor head. This minimizes any forces that the cable might be able to exert on the sensor head as a result of thermoelastic expansion and contraction in the cable. Although such configurations were considered initially, they were discarded due to a combination of mass and complications associated with deployment. In its place, we have the LSA. Prior to deployment on Mars the LSA is held closed with a Frangibolt—a bolt that will be broken to release the LSA after the seismometer has been deployed to the surface of Mars. This permits the LSA to be strong during deployment, yet weak after it has been opened.

There are two primary performance requirements that the presence of the LSA affects:

- The overall SEIS thermoelastic tilt shall not exceed $2\times 10^{-5}~\mbox{deg}/\mbox{K}$ for a variation of temperature occurring under the TBK/RWEB.
- SEIS shall be able to transmit frequencies from DC up to 55 Hz to the VBB sensor sphere and from DC to 55 Hz to the SP sensor box without any significant amplification ($Q<25$).

The thermoelastic tilt requirement is the reason for the existence of the LSA. Analysis showed that if the tether came straight across the ground and into the Sensor Assembly, that this requirement was broken by orders of magnitude.

**Fig. 67:**
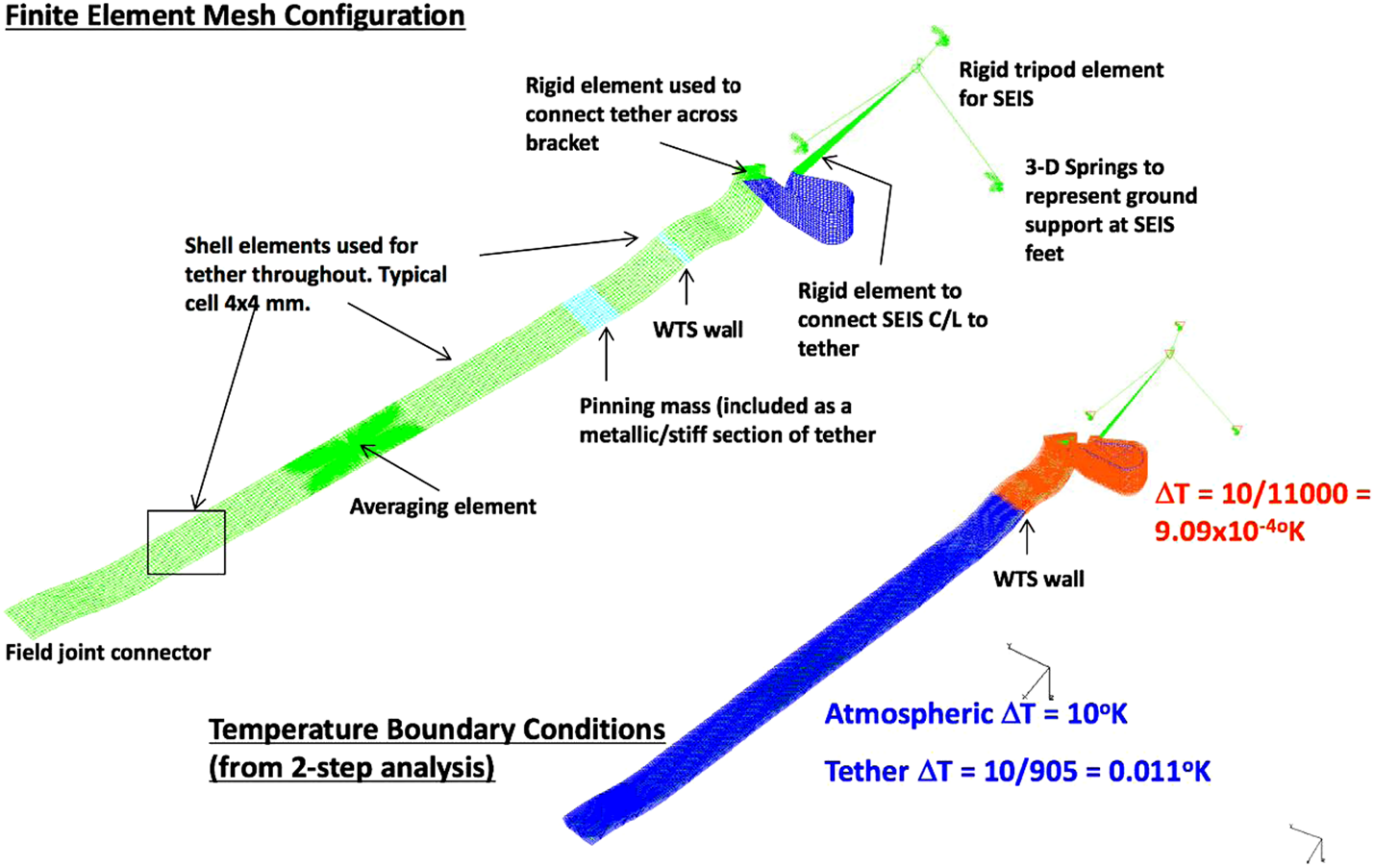
Finite element mesh configuration for thermal and elastic models of the tether

The fact that gravity on Mars is about $3/8$ that of Earth, implies that it is impossible to replicate while on Earth, the combination of interaction of the Sensor Assembly feet with the Martian regolith, the normal forces on the tether and Load Shunt Assembly and the linear and rotational inertia of the Sensor Assembly. Therefore, it is impossible to verify compliance of the system to the subsystem requirements solely via test on Earth. In the face of this situation, we have relied on unit level tests, Finite Element modeling with two independent models and formal verification methods (Uncertainty Quantification and Phenomena Identification and Ranking Table) to verify compliance with the thermoelastic requirement. Figure 67 provides some information on the primary thermal and elastic Finite Element Models with their meshing configuration. The most constraining situation for the thermoelastic case occurs at 0.1 Hz, so inertial effects are relatively small compared to the tilt signal.

Below 10 Hz, the open LSA has two natural frequencies of about 5 Hz and 8 Hz with low $Q$ of about 2.5 and 4 respectively under Earth’s gravity and under zero slope conditions. The exact frequency is influenced by details of the geometry after deployment. At this frequency, inertial effects are not negligible. The presence of this resonance is important for the second requirement above, to transmit motions from 0–55 Hz. Initial experiments have shown that the acceleration amplitude at 3 Hz between the outer portion of the LSA and seismometers on the sensor assembly is reduced by a factor of about $10^{4}$ when the sensor assembly is sitting on coarse sand under Earth’s gravity. More extensive characterization of the transfer function of the LVL and open LSA sitting on Martian regolith simulant in Earth gravity is being carried out and will be reported in a later publication.

### Evacuated Container (EC), RWEB and WTS

#### Overview

The SEIS instrument assembly includes a series of structures designed to mechanically couple the seismometer sensors to the Martian regolith while hermetically, thermally and mechanically isolating the core VBB and SP pendulums from the surrounding Mars atmospheric and thermal environment. The Evacuated Container (EC) contains the VBB sensors, the Remote Warm Enclosure Box (RWEB) contains the EC and the rest of the sensor assembly and the Wind and Thermal Shield (WTS) is placed over the whole sensor assembly as the final layer of protection.

**Fig. 68:**
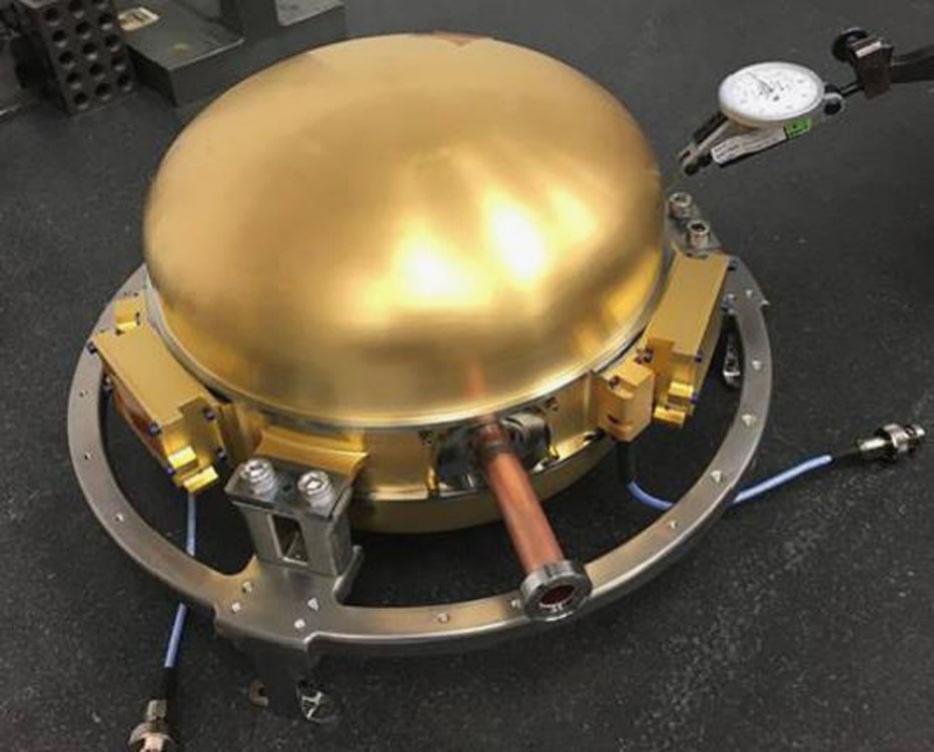
EC (Evacuated Container) with non-flight ground handling ring. The diameter of the sphere is 198 mm

The EC (Fig. 68) is a 20 cm diameter oblate spheroidal welded structure, which accommodates the vacuum environment required to minimize aerodynamic damping of the VBB oscillations (see Sect. 5.1.3) and to provide the necessary thermal protection for the VBB sensors. The vacuum environment is part of the first layer of thermal insulation to the outside environment. In addition to being leak tight to UHV (Ultra High Vacuum), the EC includes six passive gas absorption canisters to absorb any H_2_O, CO, or CO_2_ which might outgas or leak into the EC over its life and two SAES coated titanium plates (“getters”) to capture H_2_. Most of the internal EC structures are gold coated titanium to achieve the desired optical and conductive properties necessary to thermally isolate the VBBs. Finally, the EC includes six hermetic electrical feedthroughs and a $1/2''$ copper exhaust tube (queusot) which is cold welded shut at the end of assembly processing to achieve a hermetic seal.

**Fig. 69:**
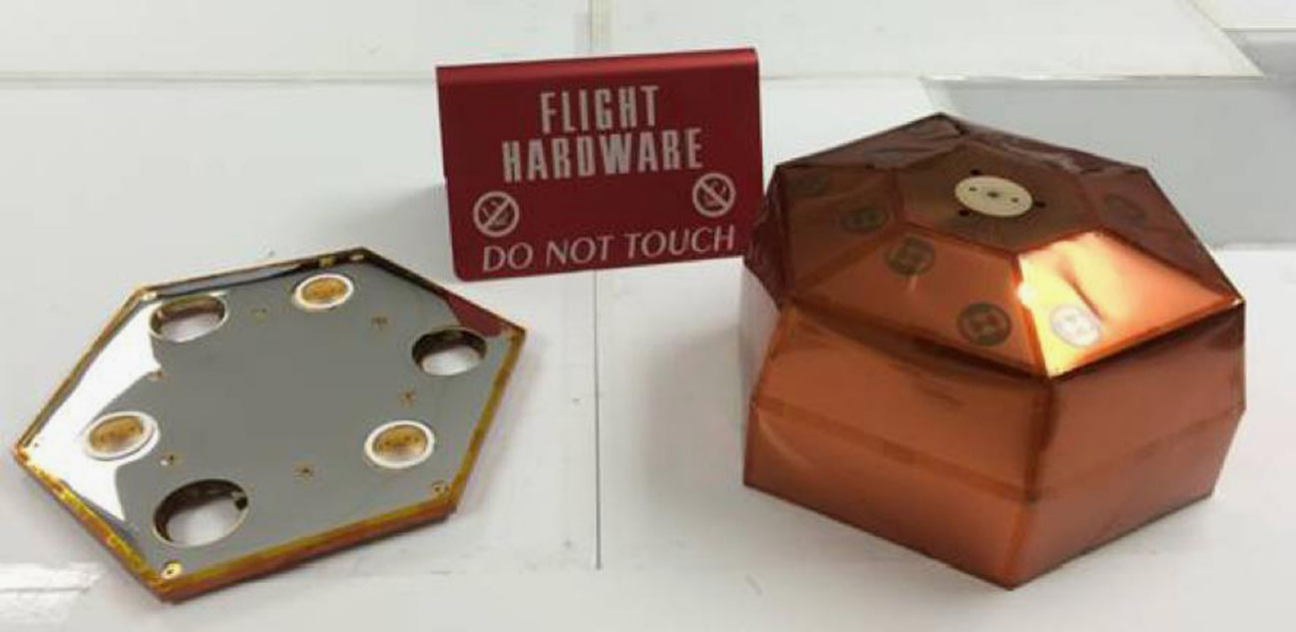
SEIS RWEB (Remote Warm Enclosure Box). The width of the RWEB is $\sim 355~\mbox{mm}$ and its height is 212.5 mm

**Fig. 70:**
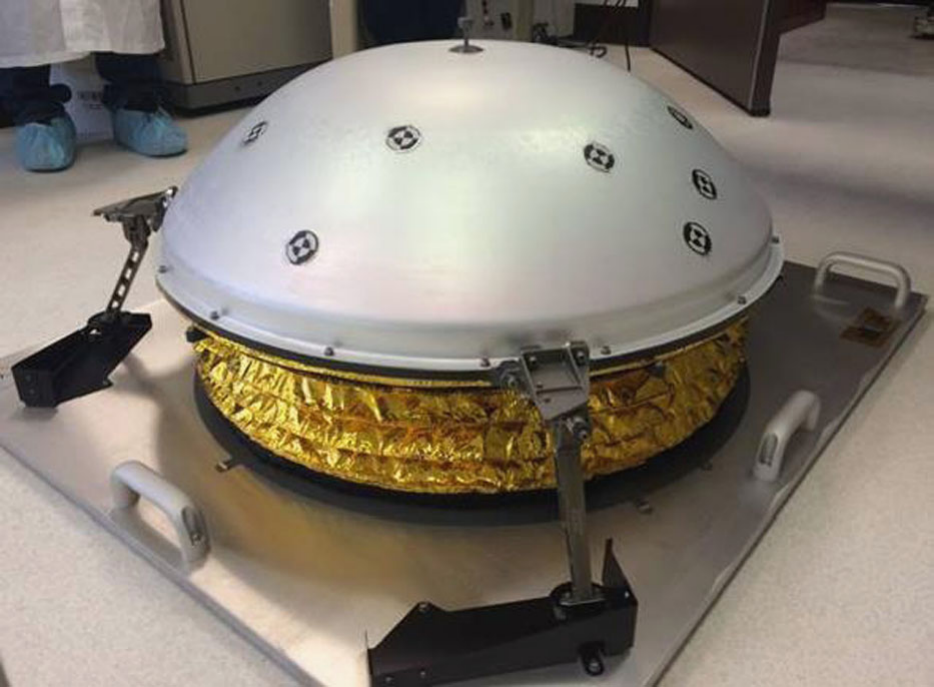
SEIS WTS (Wind and Thermal Shield). It is 72 cm in diameter and 35 cm tall

The second layer of thermal insulation around the core EC structure is the $34~\mbox{cm wide} \times 21~\mbox{cm tall}$ RWEB (Fig. 69). Constructed from titanium and mylar, the RWEB forms a 1–2 cm CO_2_ filled insulating gap around the instrument. The RWEB provides a stagnant layer of trapped CO_2_ gas and is designed to eliminate the possibility of natural convection cells from developing. All of the internal layers are coated with low IR emissivity materials. The external surface is bead blasted Kapton to achieve an absorptivity emission ratio which avoids excessive heating/cooling of the instrument while it is directly exposed to the Mars ambient environment after landing. The final layer of thermal/mechanical insulation comes from the $72~\mbox{cm diameter} \times 35~\mbox{cm tall}$ WTS (Fig. 70).

The configuration of the dome, legs and skirt have all been designed to protect the SEIS instrument from mechanical vibrations and tilts induced by Mars winds in excess of $75~\mbox{m}/\mbox{s}$. The reinforced Kapton and CRES/Aluminum chainmail skirt has also been designed to conform and seal around various terrain obstacles. As with the RWEB, the dome internal surfaces are coated with low IR emissivity aluminum and the external surface with SiO to avoid excessive heating/cooling.

Together the WTS, RWEB and Vacuum inside the Sphere provide a very significant decoupling of the instrument from the Martian environment. Specifically, a time constant of at least 11 hours is required between the VBBs and Martian atmosphere in terms of 2nd order attenuation. This is met by multiplying a 2 hours time constant between the VBBs and the outside of the Sphere, with the 5.5 hours time constant between the outside of the Sphere through the RWEB and WTS and the Martian environment. This requirement led to specific thermal design specifications of at least $4.0~\mbox{K}/\mbox{W}$ thermal resistance through the RWEB at cold temperatures (173 K) and convection heat transfer coefficients through the WTS skirt of no more than $0.28~\mbox{W}/\mbox{m}^{2}\,\mbox{K}$, as well as the need to minimize any pressure build up within the Sphere.

#### Getters Description

Zeolite-loaded aerogel (ZLA) getters were devised, developed and produced for maintaining vacuum $<0.01~\mbox{mbar}$ in the SEIS instrument, which facilitates nominal function of the VBBs. The total outgassing in the fully populated EC was estimated at roughly $10^{-7}~\mbox{mbar}\,\mbox{L}/\mbox{s}$; hence the ZLA composition was designed to cope with that gas load. The ZLA getters are light compound materials ($\sim 0.2~\mbox{g}/\mbox{cm}^{3}$) with very high surface area ($>500~\mbox{m}^{2}/\mbox{g}$), loaded with $2\mbox{--}5~\upmu\mbox{m}$ diameter zeolite particles. They were prepared using a modified two-step silica aerogel process and are loaded with fumed silica and zeolite particles in the liquid aerogel precursor. The precursor composition was chosen such that the zeolite particles stay homogeneously dispersed during the gelation process. The aerogel precursor with the dispersed zeolite particles forms a wet gel, locking the zeolite particles into the silica network formed. The material is then dried super-critically to produce a rigid silica network and to minimize shrinkage. The zeolites (13X faujasite) were ion-exchanged (Na^+^, Ca^+^, Mg^+^) to enhance the adsorption characteristics for water, CO_2_ and volatile organics, which were detected in the SEIS outgassing spectra.

The liquid precursor was cast in six Ti-6Al-4V cylinders, designed to utilize the available space in the instrument without interference. A total of $33~\mbox{cm}^{3}$ ZLA was super-critically dried in the cylinders, then outgassed and sealed with a lid. To avert the risk of particle transport, the 1 cm diameter opening on the lid provided molecular access to the getters through $1~\upmu\mbox{m}$ filters without noticeably affecting the adsorption rate. The silica aerogel provides a mesoporous network, in which the zeolite particles were dispersed, providing excellent molecular conductance to the zeolite particles. This dramatically increases the effectiveness of the zeolite adsorption in comparison with their standard pellet form applications.

Experimental verification of the ZLA performance was done by using water vapor as a proxy for the instrument outgassing. It was demonstrated that these materials are capable of maintaining $\sim 10^{-3}~\mbox{mbar}$ vacuum over extended periods of time (months to years at room temperature), meeting the engineering requirement with a large margin. The ZLA adsorbance will increase dramatically at Mars temperatures, facilitating a pressure level below $10^{-5}~\mbox{mbar}$ throughout the duration of the mission.

The residual outgassing risk not addressed by the ZLA comes from hydrogen outgassing from steel and Ti alloys. All EC Ti-6Al-4V components were outgassed in vacuum at $320^{\circ}\mbox{C}$ to reduce the outgassing by orders of magnitude. In addition, H_2_ getters were implemented by applying the standard SAES Rel-Hy deposition process on both sides of two Ti disks with large surface area (minimum $80~\mbox{cm}^{2}$ total). The disks were then welded to the inside of each shell. The manufacturer specification of $3~\mbox{cm}^{3}\,\mbox{Torr}/\mbox{cm}^{2}$ provides orders of magnitude larger absorption than the required capacity. Due to the low emissivity of the Rel-Hy film, 0.05–0.06, the getters also served as a redundant thermal shield.

#### Feedthroughs and Queusot Overview

**Fig. 71:**
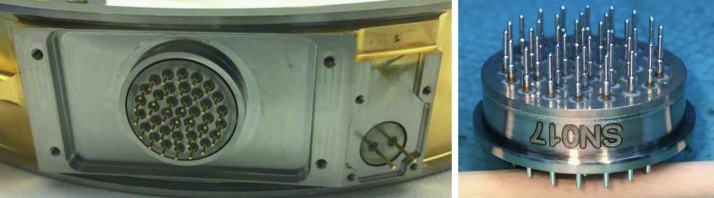
(Left) 37-pin and 2-pin electrical feedthrough installed into EC Crown; (Right) 37-pin feedthrough closeup

The EC was designed with 6 high vacuum electrical feedthroughs, three 2-pin feedthroughs and three 37-pin feedthroughs to provide power and signal paths to the VBBs (Fig. 71).

The strict leak rate requirement ($10^{-10}~\mbox{mbar}\,\mbox{L}/\mbox{s}$ helium standard leak rate) for a wide range of temperatures ($-120^{\circ}\mbox{C} \mbox{ to } {+}120^{\circ}\mbox{C}$) led to the development and qualification of these unique feedthroughs custom built for InSight by Solid Sealing Technology Inc. Both types of feedthroughs share the same key technology: each individual pin is electrically insulated from the others using BPS glass (borophosphosilicate glass) and held within a stainless-steel body (304L).

The pins and the feedthrough body are both made of stainless steel 304L which generates a diffusion bond with the glass when chromium from the stainless diffuses into the BPS glass. During the manufacturing process, at the vacuum furnace the glass becomes solid at around after $500^{\circ}\mbox{C}$ and remains in compression below this temperature, increasing robustness and decreasing the chances of crack propagation compared to standard alumina feedthroughs.

The considerable benefit of this technology compared to traditional alumina insulator feedthroughs is that the insulator is continuously maintained under compression over the entire temperature range as described above, as well as limiting the stresses and deformations due to differences in the thermal expansion coefficients to a very small radius (of the individual insulators). This compares to the single alumina design with 37 holes that would have an outer braze with a considerably larger diameter, leading to more stress due to thermal dilatation coefficient’s mismatches.

The stainless transition ring is brazed to a Ti-6Al-4V flange to allow the feedthroughs to be Electron Beam Welded (EBW) to the titanium EC. This transition ring is vacuum brazed using CuSil in a separate process. Later the transition ring is EBW to the stainless-steel body which contains the pins.

The EC is sealed using a $1/2''$ custom pinch-off tube (“queusot”). The pinch-off tube assembly was designed with a standard CF16 stainless-steel corn flat flange which later connected to the vacuum ground support equipment, an $1/2''$ oxygen free high conductivity copper tube and a Ti-6Al-4V adaptor where the assembly is EBW to the EC. The assembly process included two vacuum brazes in order to achieve a leak tightness of $10^{-10}~\mbox{mbar}\,\mbox{L}/\mbox{s}$ of He Std. The welds of the pinch-off tube to the EC, as well as for the feedthroughs, were performed from the inside of the EC to minimize possible trapped volumes.

**Fig. 72:**
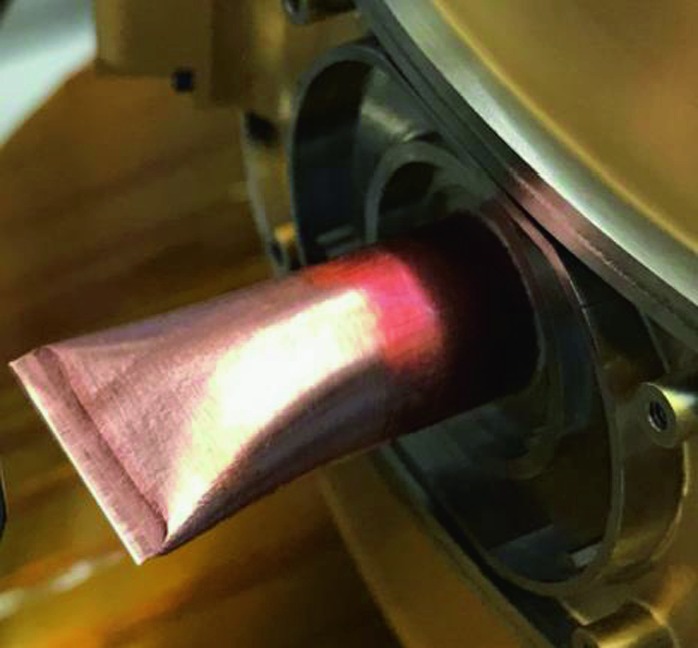
Pinched-off queusot

The sealing mechanism for the pinch-off tube was a customized hydraulic pinch-off tool provided by Custom Products & Services Inc., Model HY-500 set at 5000 psi. Once the pinch-off tool closed its jaws on the copper, the tube plastically deformed until the cold weld took place (Fig. 72), sealing the EC.

The feedthroughs and pinch-off tube were qualified at the part level undergoing multiple thermal cycles, vibration, shock and Packaging Qualification and Verification (PQV) successfully. Extensive helium leak tests were performed during the process. All qualification programs were completed.

#### EC Thermal Design

Thermal isolation of the SEIS instrument from the Martian environment is enhanced by the vacuum inside the Evacuated Container (EC). Initial studies of the heat transfer paths within the EC showed that even conduction through low pressure gas within the EC could be large compared to the thermal conduction through the titanium structure. The dominant conduction path is through the coax and flexible ribbon cables that lead from the VBB to the connectors in the Sphere wall; conduction through the Inner Plate and up through the flexures to the shell was the secondary path.

**Fig. 73:**
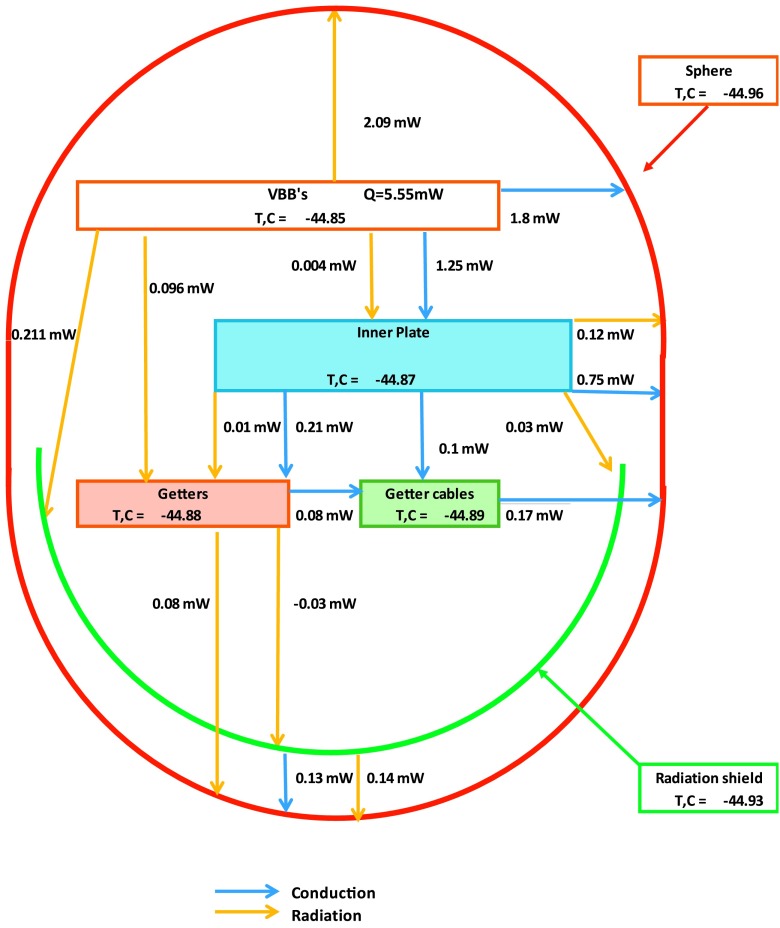
Sphere heat flow diagram under beginning of life pressure conditions. The average temperature in Celsius are given for the different units

Figure 73 shows the sphere heat flow diagram under beginning of life pressure conditions. Without gas conduction present, radiation accounts for 46% of the heat lost from a VBB and conduction through solid structure accounts for 54%. If gas pressure builds up inside the EC to the end-of-life allowable value of $10^{-1}~\mbox{mbar}$, gas conduction has passed through the free molecular regime and is in the transition regime between free molecular and full continuum flow. Under these conditions, gas conduction is the dominant path for heat loss from the VBB and may account for 57% of the value, reducing the proportion of conduction through solid structure to 35%. Radiation then accounts for only 7.6%. When this gas is present, 36% of the heat lost from a VBB is directly off the many surfaces of the VBB assembly straight to the EC walls. Table 13 below highlights these parameters, while the diagram of the EC shows the typical radiation and conduction paths in vacuum, assuming a 5.5 mW dissipation of the VBB. These studies highlighted the critical need to keep the EC leak tight and to minimize the buildup of outgassing products.

**Table 13:** Sphere heat flow summary with beginning of life pressure inside the sphere

Heat flow		Conduction % of total flow of Sphere	Radiation % of total flow of Sphere	% of total flow of Sphere
From	To			
VBB	Sphere	34.0	39.6	73.7
Getter cables	Sphere	3.3	0.0	3.3
Getters	Sphere	0.0	1.5	1.5
Thermal shield	Sphere	2.5	2.6	5.1
Inner plate	Sphere	14.2	2.2	16.4
% of total flow to Sphere		54.0	46.0	100.0

### Cradle

**Fig. 74:**
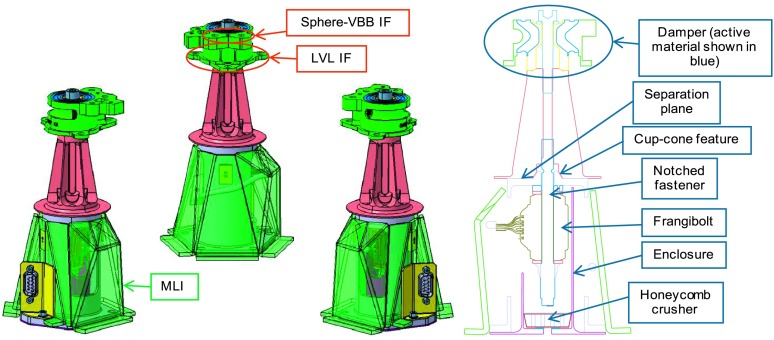
Cradle Dampers (on left) and internal design of the cradles with the release mechanism (on right). The cradles are 185.5 mm height

The Cradle subsystem connects the spacecraft at its base with the Sphere-VBB and LVL subsystems at its top (Fig. 74). It has two functions: to reduce the vibrations levels and to fix the Sensor assembly on the lander deck during launch, cruise and landing and unlock it during deployment prior the robotic arm deployment.

**Fig. 75:**
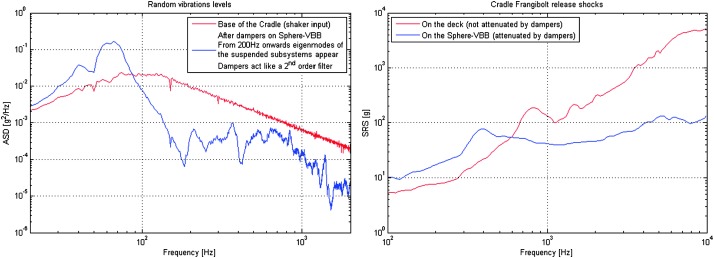
Cradle damping efficiency. The left figure provides the damping of launch vibration, while the right figure provides the damping efficiency during cradle release. Red lines are those on the deck and blue lines are the acceleration levels at the SEIS assembly

It consists of 3 nearly identical turrets at 120° around the SEIS Sensor Assembly. A set of 3 dampers is used to decrease the mechanical loads seen by the VBB and SP sensors (random vibrations during launch and shocks during release mechanisms activation (Fig. 75)).

Its active part is made of a silicone based elastomer, the geometry and material of which are tuned to provide the required characteristics. As the center of gravity does not lies precisely in the geometrical center of the 3 dampers (mainly due to the LSA—Load Shunt Assembly), the damper material closest to the LSA has been tuned to be slightly stiffer in order to achieve optimal performance. A grounding strap is also integrated between the inner and outer damper in order to have electrical continuity in the deployed configuration.

The Cradle also releases the deployed part of the Sensor Assembly once on Mars. The separation plane is at the level of the lower thermal blanket in order to avoid catch hazards during deployment. A cup-cone feature ensures that the Sensor Assembly remains in place after release (the deck inclination can reach up to 15°). The Launch Lock assembly is built around an off-the-shelf FC4 Frangibolt. The Frangibolt is an SMA (Shape Memory Alloy) device in the shape of a tube which extends in length when heated over its transition temperature (around 90°C). It is mounted around the Ti-6Al-4V fastener which fixes the deployed part of the Cradle to the non-deployed part. Upon actuation of the Frangibolt, the fastener breaks at a notched portion which is set at the separation interface. Washers are mounted at both sides of the Frangibolts to spread the loads. An additional vented washer is mounted to the separation interface to act as a thermal barrier to keep heating energy in the SMA material. An enclosure acts as a bolt catcher and as a radiative screen for the Frangibolt. A honeycomb crusher is integrated on the bottom of the enclosure to absorb the kinetic energy of the broken fastener.

The Frangibolt has nominal and redundant heater circuits that are connected to the unregulated load switches of the spacecraft. Depending on the spacecraft bus voltage, between 60 W and 110 W heating power is applied during actuation. A Pt1000 RTD integrated in the Frangibolt provides temperature feedback. Actuation times are between 15 s and 110 s, depending on firing circuits, starting temperature and bus voltage.

## Noise and Transfer Functions Measurement Strategy

### Measurement Strategy, Setup and Testing Sites

#### Measurement Strategy

The self-noise of the SEIS seismometers (see Table 4 and Sect. 3.4) is a key parameter because it defines the smallest signal detectable on Mars. It is very likely that during the day time, this noise floor will be much less than the signal recorded, which will contain contributions from the environment noise and station noise, the sum of the two later being described in the SEIS noise model (Mimoun et al. 2017) and described in Sect. 3.4. Possibly, an additional noise might also be recorded, associated with Rayleigh waves trapped in the low velocity zone of the subsurface, similar to Earth observations (e.g. Withers et al. 1996). It is however likely, as indicated on Fig. 6, that the quietest period during the noise might be closer to the self-noise limit, especially in the frequency band 0.02–5 Hz which remains far from the temperature noise which is likely the most limiting source of noise at long period for surface temperature non-controlled installations.

The SEIS noise floors are very low and their measurement on Earth is very challenging due to the natural and anthropogenic background noise, the limited amount of time for tests made available by the development schedule, the limited numbers of models available and, for the VBBs and the vertical SPs, the fact that the sensors cannot be operated under Earth gravity in nominal configuration. For comparison, incoherent noise between two Streckeisen STS-2 seismometers in a vault installation, is close to $2\times 10^{-10}~\mbox{m}\,\mbox{s}^{-2}/\sqrt{\mbox{Hz}}$ between 0.05 Hz and 0.5 Hz on the vertical axis while almost reaching $10^{-9}~\mbox{m}/\mbox{s}^{2}/\sqrt{\mbox{Hz}}$ at 100 s for the horizontal components (Kinemetrics 2017).

Since the seismic noise on Earth is present everywhere and remains largely above the VBB requirement between 0.05 Hz and 1 Hz, it is not possible to directly assess compliance with this requirement. At larger periods, differences of installations can also generate noise levels larger than the VBB requirements on horizontal components, which are rarely much below $10^{-8}~\mbox{m}\,\mbox{s}^{-2}/\sqrt{\mbox{Hz}}$ at 100 secs (e.g. Beauduin et al. 1996). See another example in Fig. 91 during the tests performed at BFO between 3 STS-2s on the same seismic pillar.

As a result, we tested the seismometers with the two instrument coincidence testing techniques (e.g. Holcomb 1989; Ringler et al. 2011), while three channel correlations will be considered for further analysis (e.g. Sleeman et al. 2006). To have good results with this method, three constraints were integrated:

- The self-noise of the reference seismometer used to measure the ambient noise had to be lower than those of the sensors to characterize and we used STS-2s. We additionally used Trillium compacts to better map the noise at different locations with respect to the SEIS instrument.
- We performed the tests in low noise environments by removing as much as possible all the potential sources of noise. For flight hardware, this was only possible in selected clean rooms at the SEIS level and in urban seismic vaults at the sensor level. But for Engineering Model tests, tests were made in low noise seismic vaults.
- The coherence between reference seismometer and seismometers under test was optimized and the quietest periods were used for an efficient non-coherent noise estimation.

The processing of test data for estimating sensor noise and transfer function has been performed independently by Imperial College (ICL), ISAE, IPGP and CNES teams, the CNES algorithm being only used for double checking during the last noise tests. Teams exchanged their codes and performed cross-validation of their software. However, some differences in the processing are kept in between these approaches: the alignment of the test sensor axis with the reference sensor axis in the same direction is done in the time domain by ICL and in the frequency domain by others and the estimates of noise level are sometimes different. On this last point, ICL results appear to be able to remove the micro-seismic noise peaks remaining in the ISAE estimates, probably because of a different way to manage the cross-axis sensitivity of sensors. Noise between the POS and VEL outputs of the VBBs was also measured and compared to the noise model.

The transfer functions were estimated either relative to reference sensors or by coil calibration. They are converted to absolute transfer functions by using the calibration of the reference sensors.

#### Experimental Setup

**Fig. 76:**
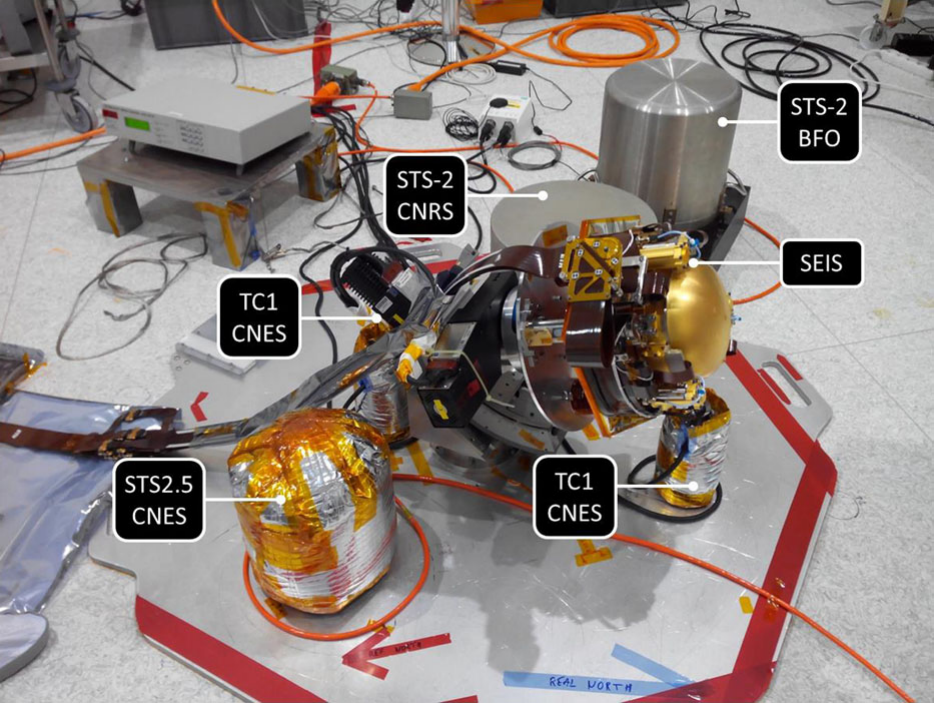
Typical setup with 5 reference seismometers. STS-2 BFO was covered by an air lock, STS-2 CNRS was covered by mu-metal/thermal shield and STS-2.5 only by thermal shield. SEIS was tilted at 68° to balance one VBB and the vertical SP

**Fig. 77:**
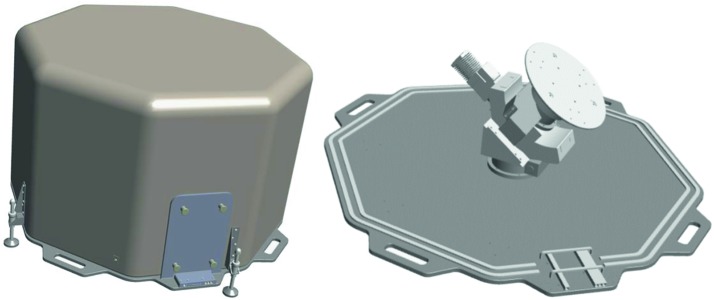
Left: Big thermal shield used for tests. Right: Aluminum plate and goniometer mounting. SEIS was mounted on the goniometer while the reference seismometers were installed on the plate or nearby

The typical setup for noise measurements consists of recording seismic signals with many reference seismometers as close as possible to the instrument. SEIS was put on a goniometer to simulate the Mars gravity (Fig. 76 for SEIS without TBK and Fig. 81b for SEIS with RWEB) and allow the VBB and vertical SP to be balanced on Earth. This goniometer was likely the main source of non-coherent noise which depreciated noise estimations. A large heavy metallic aluminum plate put on the cleanroom floor was used to maximize coherency between all these sensors and a big thermal shield covered all the setup to minimize effects at long periods (Fig. 77). The big thermal shield was decoupled from the plate to avoid additional noise due to the drum effect and each reference seismometer under this dome received individual skinny thermal protection to prevent convection.

**Fig. 78:**
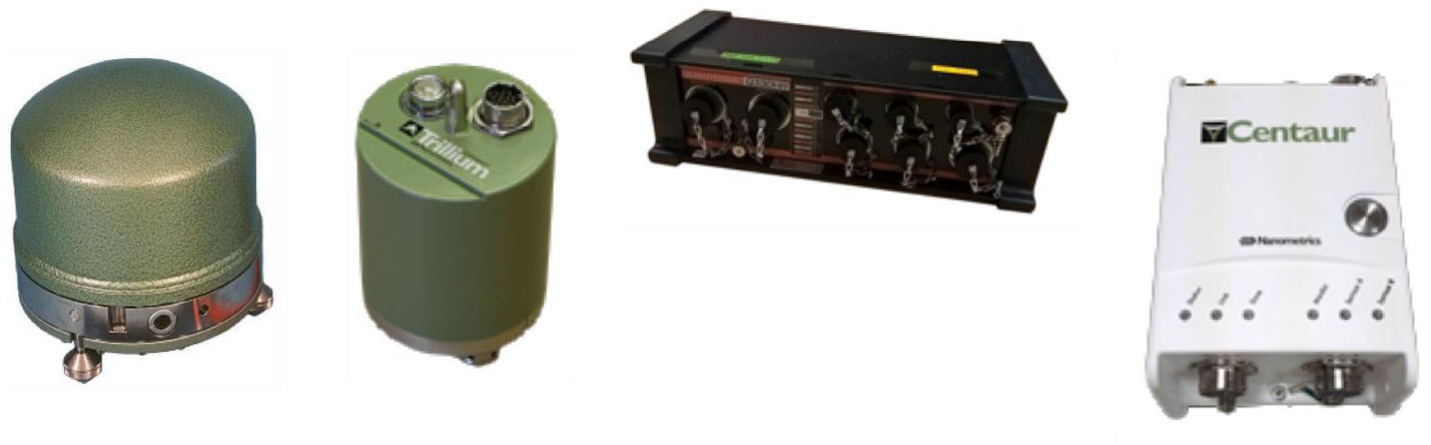
References sensors and reference acquisition *systems* used for the performance tests. From left to right, STS-2 and Trillium compact seismometers from Streckeisen and Nanometrics respectively, Q330HR and Centaur digitizers from Kinemetrics and Nanometrics respectively

The reference seismometers used for noise tests were 2 (or 3) STS-2s from Streckeisen and 2 Trillium compact from Nanometrics (Fig. 78). Even if the Trillium compact self-noise is too high for the VBB assessment, they are still useful for SPs self-noise assessment. The STS-2s were connected to a 6 channels/26 bits Q330HR Data acquisition unit, while 6 channels/24 bits Centaur acquisition unit recorded the Trilliums.

In addition, environment parameters like pressure, magnetic field and temperature were sampled and recorded at 100 Hz, the same sampling rate as the velocity output data of both SEIS and the reference seismometers. Note that the pressure sensor was a MB2005 microbarometer (Larsonnier and Miller 2011) able to measure small pressure fluctuations around the ambient pressure in a 20 Hz bandwidth. The synchronization of all the data acquisition units was possible using a GPS repeater in the cleanroom. An external active antenna receives GPS signal, amplifies and provides it in the cleanroom through a passive antenna. Nevertheless, even if all the reference acquisition units were synchronized by GPS, it was not the case for the EBOX which time-tags the VBBs and SPs acquisition.

Several different ways were tested to achieve the good synchronization that was required for post processing

**Fig. 79:**
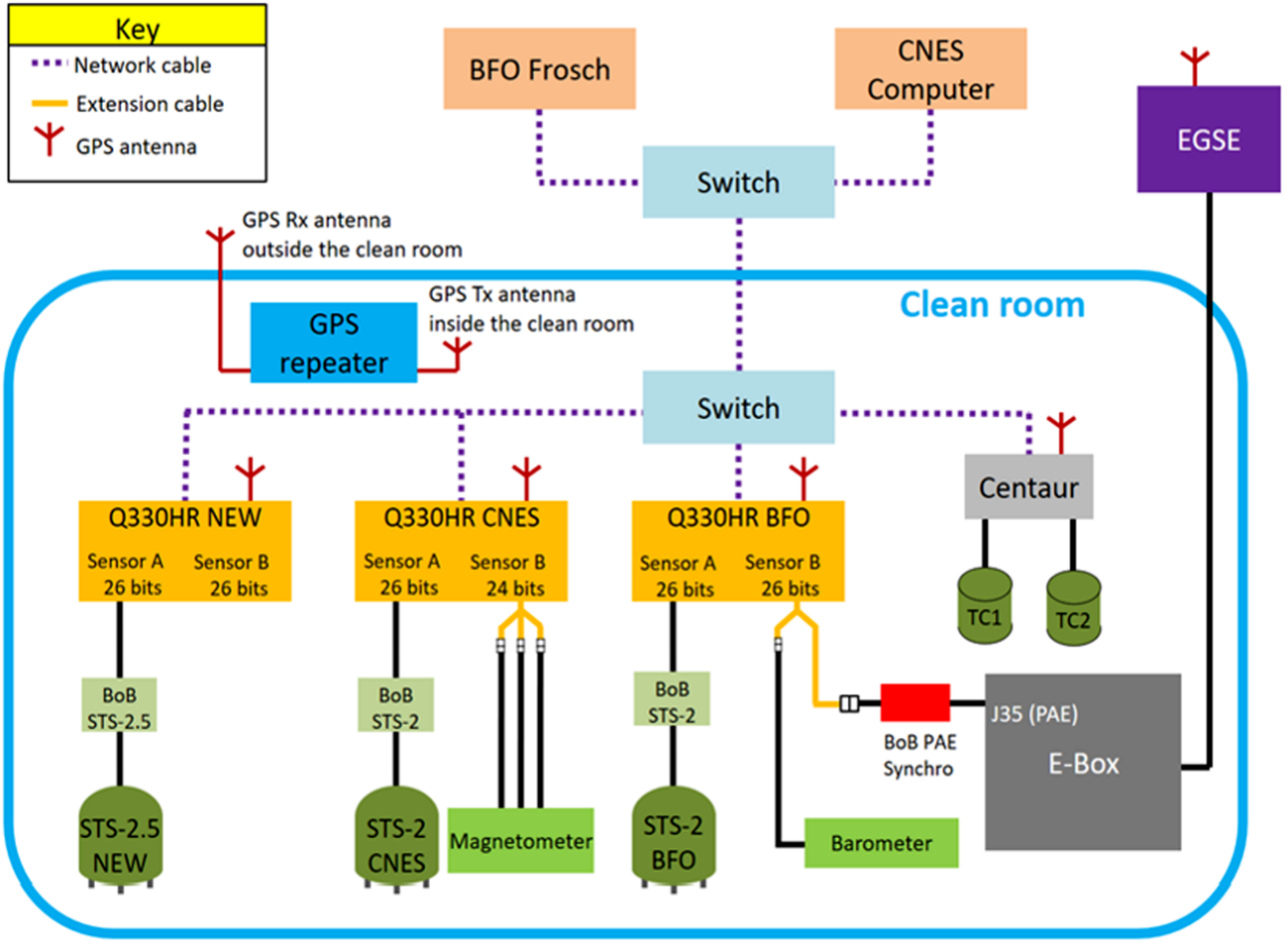
Typical setup with 5 reference seismometers. SEIS (not seen) is connected to the Ebox

- The Ebox clock was updated from a GPS synchronized PC at the beginning of each test. Nevertheless, this update was asynchronous and did not ensure an accuracy better than 0.5 s.
- Calibrated shocks were done on the plate at the beginning and at the end of each acquisition period. These shocks were seen by both reference seismometers and SEIS sensors and facilitated the alignment of time series.
- An additional box (BOB PAE Synchro—see Fig. 79) was connected to the EBOX to record safely the 1PPS signal provided from the EBOX clock to the external APSS sensors

All these methods contributed to improve the synchronization of the records but, finally, post processing based on the coherency method gave the last correction before the noise estimation process.

Finally, all data acquisition units were connected to a local network for remote control and data collection.

#### Test Sites

Most of the tests with the full SEIS instrument were performed with the flight model in the cleanroom at CNES. Nevertheless, despite all the efforts to remove anthropogenic noise sources (air conditioning, fans, lights…) and to prevent excess noise (remote control, tests performed during night and weekend…) a large portion of background noise remained. This situation prevented us from meeting self-noise requirement level noise outside the 1s–10s band for the VBBs. Nevertheless, the self-noise compliance was pretty fully demonstrated for SPs in the full band because the requirement level is ${\times}10$ above the VBB one.

The last tests performed in ATLO in Lockheed Martin facilities gave the worst results because major sources of noise, such as air condition, had not been switched off to prevent risks to the Insight lander flight model.

**Fig. 80:**
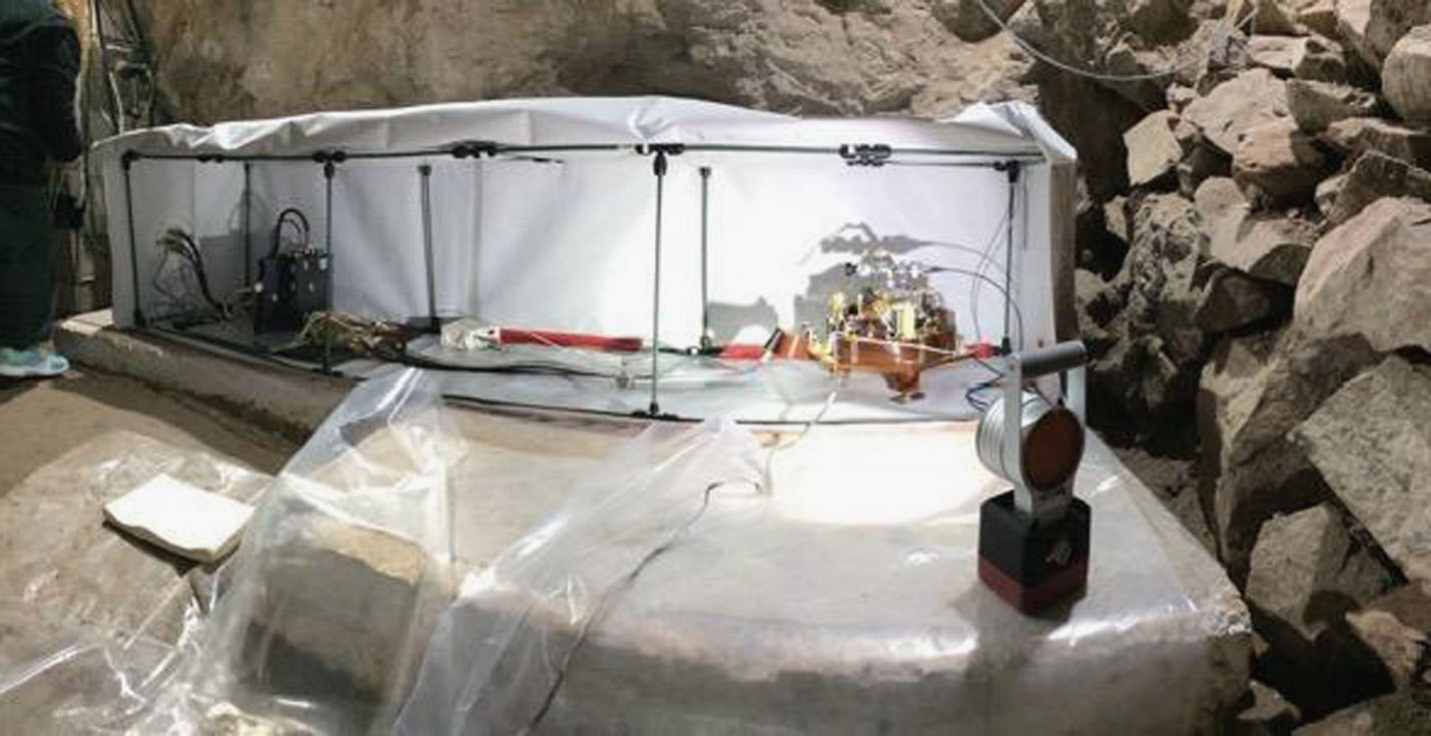
Setup for SEIS QM test in the Black Forest Observatory mine seismic vault. EBOX on the left, sensor assembly with “cuvinette” on the right. Once the tent was closed the reference seismometers were installed on the pillar in the foreground

In order to demonstrate that the SEIS seismometers meet the requirement over the full frequency band by design, a dedicated test campaign was carried out at the Black Forest Observatory, Germany. This place is the quietest facility in Europe and likely in the world, dedicated to seismic long period measurements. However, the seismic vault is at the bottom of an old silver mine with high humidity and dusty environment not compatible for space instruments. For that reason, the tests were done with the qualification model with great care. A first test was performed in March 2017 to complete the SPs noise assessment and proved that these sensors meet the requirement in the full band with margins. Because no more VBB in a sphere was available for this test, we had to develop a dedicated small vacuum chamber able to receive the VBB#11 in Earth configuration on the SEIS leveling system (cuvinette). In addition, a specific tent to control humidity in the same range as the cleanroom ($55 \pm 10\%$) had been built with a passive humidity control based on desiccant (to avoid noise induced by a standard dehumidifier, see Fig. 80). This new test campaign at BFO occurred in March 2018.

### VBBs Results

#### Earth Operation of Flight Models VBBs

To be operational the torque exerted by gravity must be equal and opposite in sign to the torque by the spring and therefore when

11 $$ M_{0} = m_{\mathrm{Earth}} [ \vec{D}_{g, \mathrm{Earth}} \times \vec{g}_{\mathrm{Earth}} ]\cdot \vec{n}_{\mathrm{VBB}} = m gD _{g} \sin ( \alpha ), $$

where $m_{\mathrm{Earth}}$ and $\vec{D}_{g, \mathrm{Earth}}$ are the Earth mass and Center of gravity in the Earth configuration, $\vec{g}_{\mathrm{Earth}} $ the Earth gravity vector, $M_{0}$ the moment of the spring at equilibrium and $\vec{n}_{\mathrm{VBB}}$ the VBB sensing direction. This allows therefore two testing strategies.

The first one acts on the product $g_{\mathrm{Earth}} m_{\mathrm{Earth}} \vec{D}_{g, \mathrm{Earth}}$ and consists in reducing its norm to the Mars one by adding a mass on the opposite side of the pivot, in order to reduce the larger gravity in such a way that

$$ g_{\mathrm{Earth}} m_{\mathrm{Earth}} D_{g, \mathrm{Earth}} = g_{\mathrm{Mars}} m_{\mathrm{Mars}} D_{g, \mathrm{Mars}}. $$

The drawback of this additional mass is that the mechanical gain is reduced by the ratio of the Mars to Earth gravity and therefore by a factor of 2.65. Tests in this configuration have been made either with the early prototype or in the Black Forest Observatory with the EM model.

**Fig. 81:**
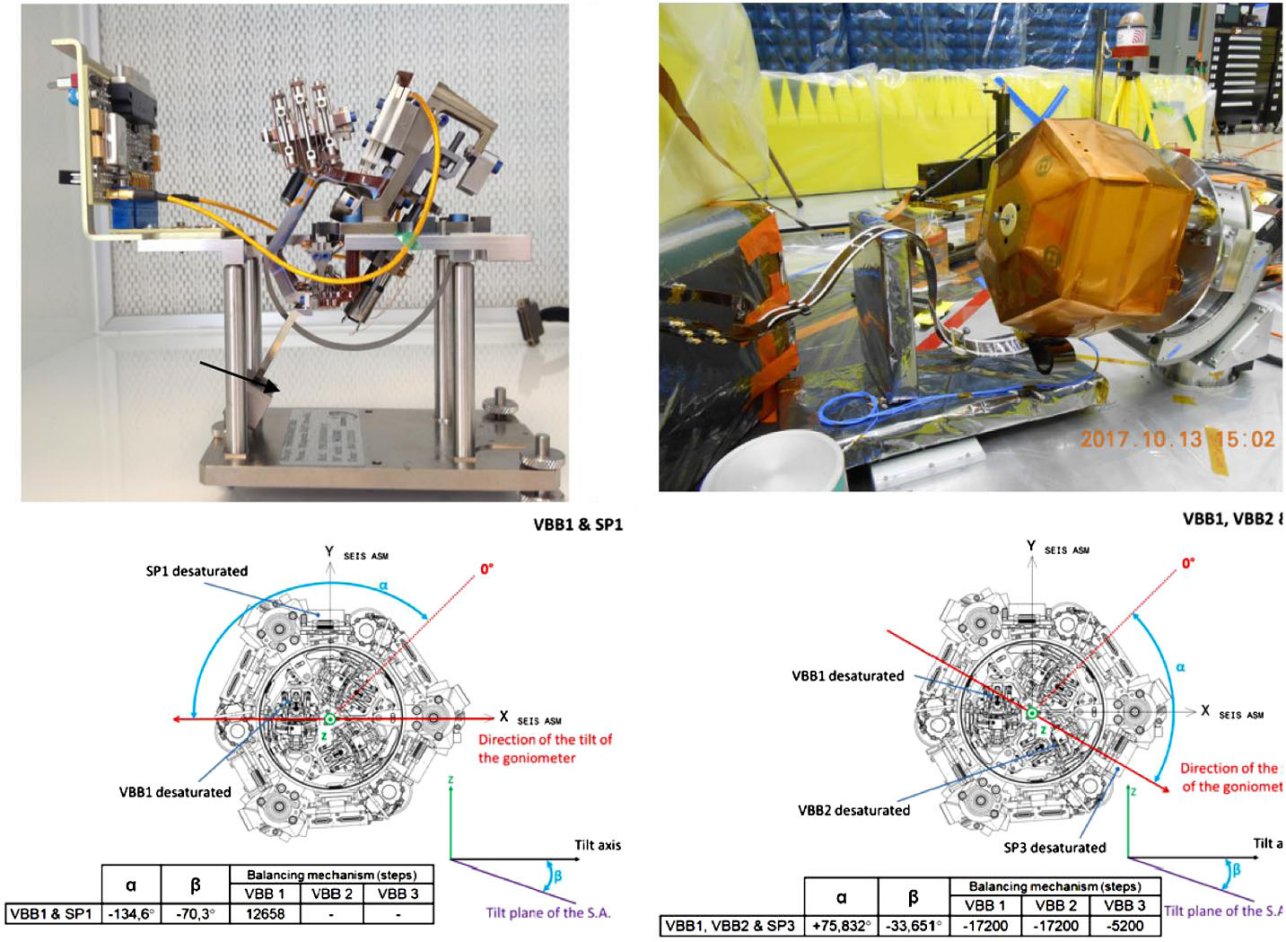
Top left: VBB in Earth configuration. The Earth mass is indicated by a black arrow. VBBs can operate in Earth mass in the nominal configuration but with a gain smaller than the Flight model by about 2.65. Top right. Flight model during tests as fixed on the test goniometer. The test goniometer was used to tilt the Sensor Assembly to the required position. Bottom left: one of the 68° positions, used for testing the VBB1. The rotation is made with a rotation axis in the direction of Y. During this rotation, both VBB1 and the vertical SP1 can be desaturated. Generally, fine recentering was made with the goniometer in order to reduce the use of the Flight unit’s recentering motors. Bottom right: Configuration for the 32° test, in which two VBBs as well as a horizontal SP can be desaturated and tested

The second strategy is to tilt the sensor in order to get an Earth gravity projection equal to the Mars one, with the aid of a goniometer (Fig. 81). This can be achieved either by a 19° tilt of the plane defined by the pivot direction and $\vec{D}_{g, \mathrm{Earth}}$ or by tilting the VBB on its side. Two sides of tilting were used. A tilting at $68\mbox{--}70^{\circ}$, where the tilt direction is in the plane defined by the pivot and gravity and a $32\mbox{--}34^{\circ}$ tilting, where the tilt direction is in a plane with a 60° angle with the pivot, which enable to test two VBBs on Earth. In all these tilting configurations, the precise value of the tilt is depending on the recentering mass, which explains the angular range.

In all these configurations, care must however be taken regarding restoring moment of the VBBs because of the inverted pendulum design. This can be expressed as

12 $$\begin{aligned} M =& - \biggl[ c - \frac{\partial [ m_{\mathrm{Earth}} [ \vec{D}_{g, \mathrm{Earth}} \times \vec{g}_{\mathrm{Earth}} ]\cdot \vec{n}_{\mathrm{VBB}} ]}{\partial \alpha _{\mathrm{vbb}}} + p \biggr] \delta \alpha _{\mathrm{vbb}} \\ &{}+ \frac{\partial [ m_{\mathrm{Earth}} [ \vec{D}_{g, \mathrm{Earth}} \times \vec{g}_{\mathrm{Earth}} ]\cdot \vec{n}_{\mathrm{VBB}} ]}{\partial \beta _{\mathrm{gonio}}} \delta \beta _{\mathrm{gonio}} \end{aligned}$$

where $\delta \alpha _{\mathrm{vbb}}$ is the pivot angular rotation and the angular deviation with respect to the equilibrium position, corresponding to rotation in the pivot direction and where $\delta \beta _{\mathrm{gonio}} $ is the rotation of the goniometer corresponding to rotation in the direction of the center of mass position $\vec{D}_{g, \mathrm{Earth}}$. The first part of Eq. (12) shows that the natural frequency of the VBB will depend on the tilt and can even be imaginary, when

13 $$ c + p < \frac{\partial [ m_{\mathrm{Earth}} [ \vec{D} _{g, \mathrm{Earth}} \times \vec{g}_{\mathrm{Earth}} ]\cdot \vec{n}_{\mathrm{VBB}} ]}{\partial \alpha _{\mathrm{vbb}}}, $$

and the second part shows that the small rotations of the goniometer transverse to the VBB sensing axis, either due to creep in the goniometer or due to ground micro-seismic noise, are generating a moment change and therefore a decrease of the signal to noise ratio. Table 14 summarizes the different configurations. Note that for the 19° and 32° configurations, self-noise increases due to a larger norm of the frequency and a smaller mechanical gain while in the 68° configuration, a source of noise appears due to the transverse sensitivity. All these consequences of the tests under Earth gravity made the performances tests somehow challenging, especially with the respect to the sensitivity at long periods.

**Table 14:** Frequencies and sensitivity directions of the VBB, including the transverse mode in tilted configuration. Only the $0^{\circ}$ (on Mars) and $68^{\circ}$ (on Earth) configuration have the same frequency and are stable. Both the $19^{\circ}$ (Earth) and $32^{\circ}$ (Earth) configuration are unstable with large imaginary frequencies. Only the $19^{\circ}$ (Earth) and Mars nominal configurations have zero transverse sensitivity, while the $68^{\circ}$ and $32^{\circ}$ have a growing transverse sensitivity. The TT angle provides the azimuth of the sensitivity of the VBB. In all cases, feedback recovers the un-stability

Configuration	Unit	0^∘^ (Mars)	19^∘^ (Earth)	32^∘^ (Earth)
Long stiffness	N m/radian	0.0032	−0.0273	−0.0218
Frequency	Hz	0.546	1.58*i*	1.417*i*
Trans stiffness	N m/radian	0	0.000	−0.022
Trans frequency	Hz	0.000	0.000	1.45*i*
TT cosine	degree	90	90	62.27
Noise (100 s)	nm/s/s/V Hz	0.9	3.3	2.7

#### VBB Transfer Function Calibrations on Earth

##### Overview

**Fig. 82:**
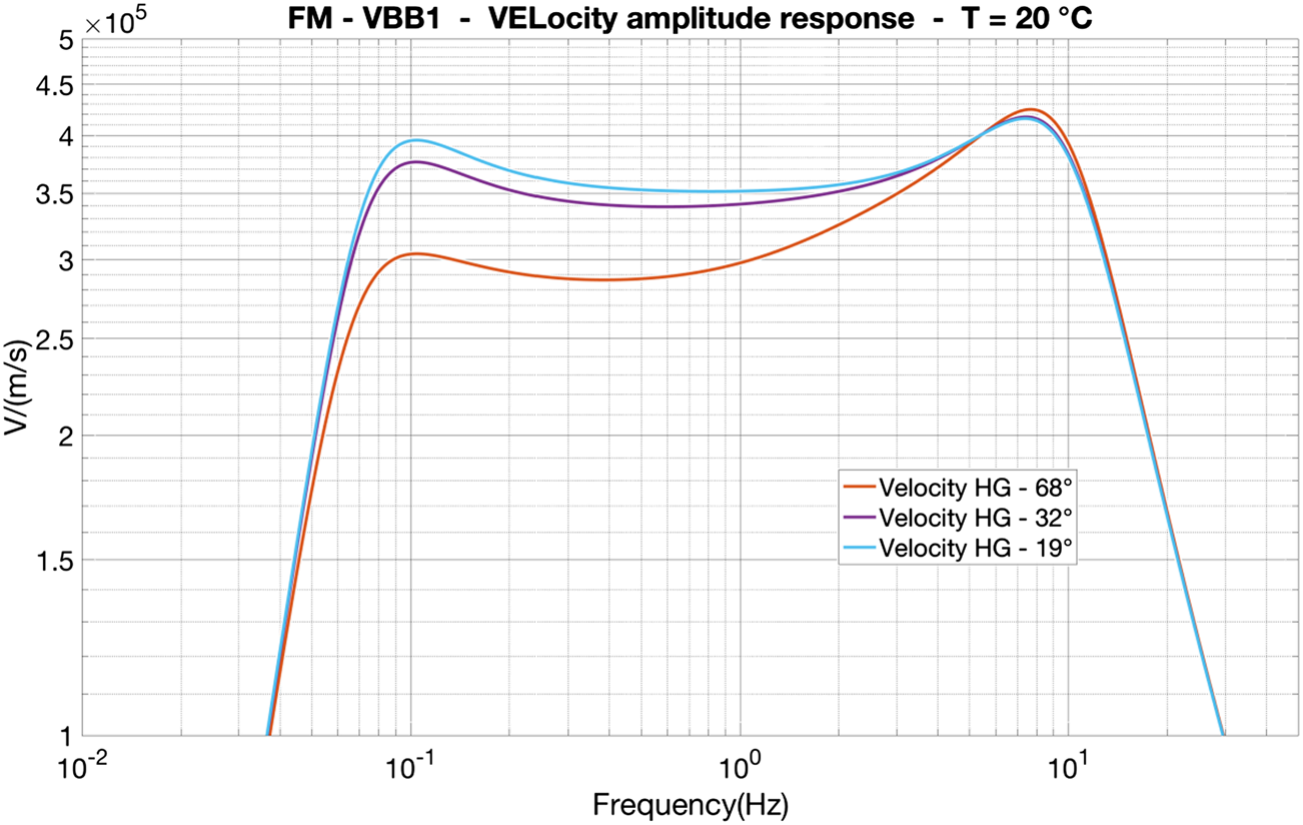
Details of the Transfer function between 30 s (0.03 Hz) and 30 Hz for the VEL output for the different testing configurations. Up to 30% of variations of the Transfer function are observed mostly due to the VBB frequency change. Gain is always larger than the 68° configuration which is close to the Flight configuration. Note however that even in this case, the pivot is operating in off-nominal condition, with a large force along its rotation axis due to the projected weight of the pendulum

Transfer function of the VBB were therefore different for all tests, as the feedback is not strong enough to fully erase the large variation of the instrument frequency between these configurations (Fig. 82). All calibrations have therefore been made with respect to the Instrument Model, which is able to correct for these operational configuration differences.

The full Flight unit, with flight tether and flight Ebox, has been calibrated and tested during 4 weeks during the project development. Two of these weeks were made prior to the delivery of the instrument in one of the cleanrooms of CNES in Toulouse, France, while the other two weeks of testing have been made in Denver, in the LMA facility. In both cases, tests were made before and after environmental tests. To compensate for the increased gravity on Earth relative to Mars, the entire SEIS instrument package was tilted such that every night at least one VBB (for the 68° tilt) or two (for the 32° tilt) sensor(s) were operational. At the end of each night a frequency calibration of the operational VBB sensor was performed. So, two types of calibrations could be compared: the built-in one and the one made by comparing the VBB signals to the reference instrument through the two instrument coincident techniques.

Dispersion of the gain measurements of the VBBs during coil calibration were ranging 4–6% for the VEL gain and 0.7–0.9% for the POS gain. Full calibration information is given in the SEED dataless associated with the VBB sensors and we provide in this section only part of the calibration information.

##### VBB Coil Calibration

The VBB seismometers and the EBOX were built to provide the possibility to perform relative calibrations once deployed on the surface of Mars. To that goal the EBOX can generate a well-defined calibration current which can be fed into the calibration coils integrated in each of the VBB sensors. By doing so, a force proportional to the calibration current and the coil efficiency is exerted on the proof mass of the seismometer that simulates a ground acceleration. From the known electric current and the measured response of the seismometer it is then possible to estimate the frequency dependent response of the seismometer to accelerations of the ground. What this experiment does not provide is the absolute gain of the seismometer, as only the gain relative to the injected force is determined.

**Fig. 83:**
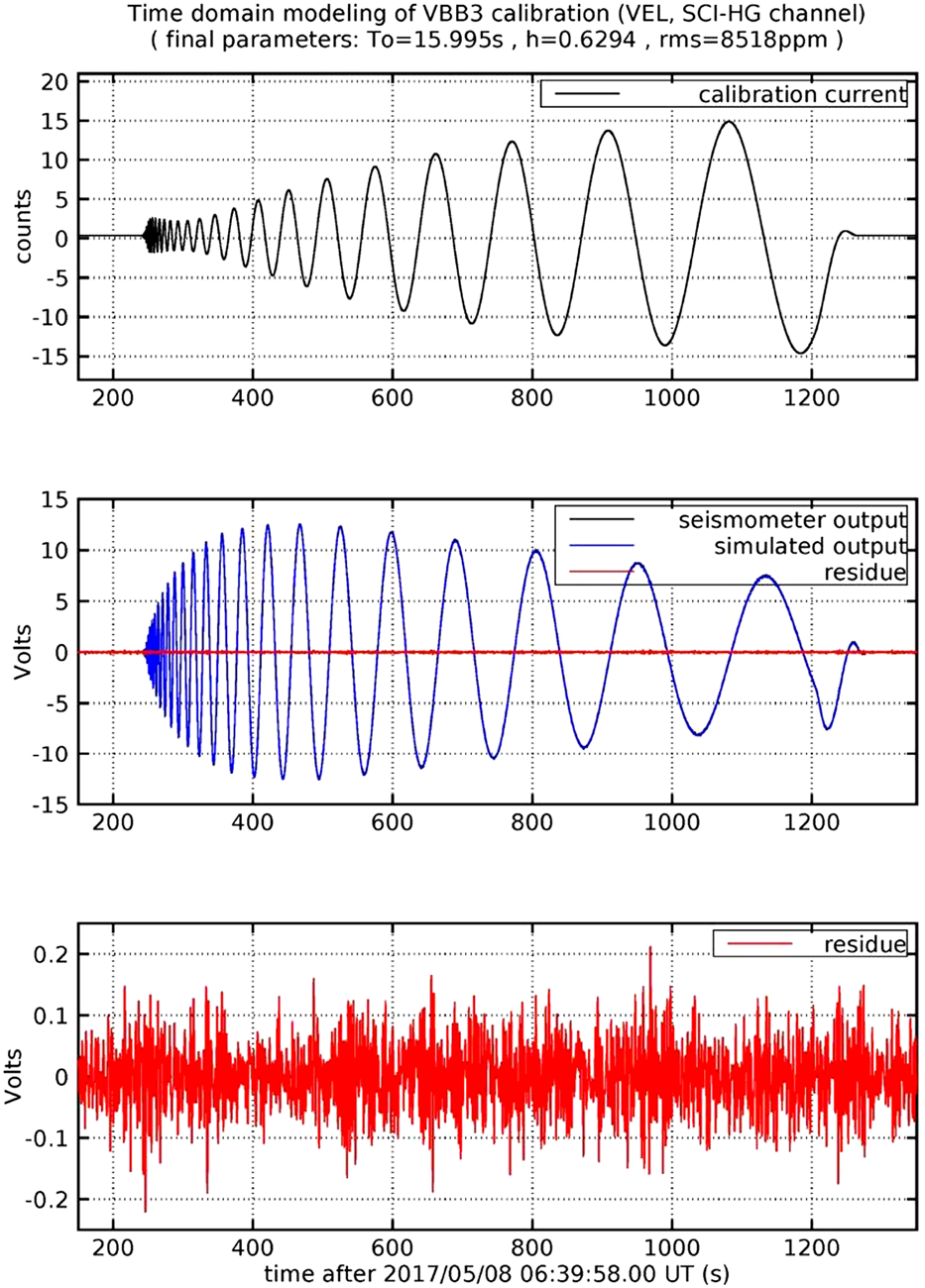
Modeling of the VBB seismometer output from a sweep calibration experiment. The top panel shows the current flowing through the calibration coil. It covers frequencies from 1 Hz to 0.01 Hz. The middle panel shows the output of the seismometer overlain with the modeled output and the residue, that is the difference between the two signals. The bottom panel shows only the residue. The modeled output fits the measured output so well that nothing from the sweep is visible in the residue. Only the background noise level from CNES is visible. Instrument parameters can be constrained more tightly if such test can be conducted at seismically quieter locations

Calibration is made by a 1000 s long sweep (top panel of Fig. 83) applied to the calibration coil of the VBB, which is defined as the digital input to the Digital-to-Analog converter, sampled at 20 sps (samples per second). This wave form is stored in the EPROM of the EBOX and can be modified by upload and command. Both the POS and VEL outputs of the VBB were digitized and recorded in the Ebox: the POS signal at 1 sps and the VEL signal at 100 sps. The FORTRAN program CALEX (Wielandt and Forbriger 2016), which uses impulse invariant recursive filters (Schuessler 1981) to model the digital output of a system with a rational transfer function was used on the processed data, decimated to the same acquisition rate as the calibration waveform (20 sps) and detrended for POS output when necessary. CALEX uses a conjugate gradient method to find a best fitting model. As such it depends on a starting model and we used the nominal VBB transfer function for that purpose.

In the frequency band covered by the sweep the transfer function of the VBB to ground acceleration can be modeled by a second-order system: a second order band pass for the VEL channel and a second order low-pass for the POS channel. There are thus only 4 unknowns in this model: a time shift $\delta $, a gain factor $A$ and one complex conjugate pair of poles in the $s = i\omega $ plane. The same model can also be represented by the physically more intuitive parameters $\delta $, $A$, $T_{0} $ and $h$, where $T_{0}$ is the corner period and $h$ the fraction of critical damping of a second-order system.

The expressions by which $T_{0} $ and $h$ are related to the complex conjugate pair of poles $p$, $q$ are:

14 $$ T_{0} = \frac{2 \pi }{\sqrt{pq}}\quad \mbox{and}\quad h= \frac{p + q}{2\sqrt{pq}}. $$

The pole-zero transfer function model for the POS channel to ground accelerations is then

15 $$ H_{\mathrm{POS}} ( s )= A_{p} \frac{1}{( s - p )( s - q )}, $$

where $s$ is the complex frequency of the Laplace transformation. This gives for the VEL channel:

16 $$ H_{\mathrm{VEL}} ( s )= A_{v} \frac{s}{( s - p )( s - q )}. $$

Table 15 and Fig. 83 summarize the results of the CALEX runs. The normalized *rms* residue (= *rms* of the residue divided by *rms* of the signal) given in ppm units was typically a factor of 5–10 larger for the 20 sps VEL data than the VEL or POS data sampled at 1 sps. This was only due to the large high-frequency ambient seismic noise present at CNES. Much of this noise is above 1 Hz and hence outside the band covered by the sweep. The VEL data was therefore also low-passed and decimated to 1 sps. The modeling of the VEL sweeps worked almost perfectly: no sign of the sweep is left in the residue. The simple model with only 4 parameters can completely explain the seismometer output.

**Table 15:** Summary of the obtained system parameters from the frequency calibrations at CNES. During the first three nights the tilting of the SEIS instrument package was such that only one sensor could be calibrated. For the last night the orientation of SEIS was such that both VBB1 and VBB3 were balanced and could be calibrated

	Start time of sweep (UT)	Output	To (s)	*h*	rms (ppm)	Sampling freq.
VBB1	2017/05/06 05:45	VEL	16.114	0.6229	21200	$ 20~\mbox{Hz} $
		VEL	16.110	0.6229	6442	$ 1~\mbox{Hz} $
		POS	16.162	0.6252	1776	$ 1~\mbox{Hz} $
VBB2	2017/05/07 06:46	VEL	16.020	0.6279	14846	$ 20~\mbox{Hz} $
		VEL	16.004	0.6283	3101	$ 1~\mbox{Hz} $
		POS	16.024	0.6321	1112	$ 1~\mbox{Hz} $
VBB3	2017/05/08 06:39	VEL	15.995	0.6294	8518	$ 20~\mbox{Hz} $
		VEL	16.000	0.6292	2533	$ 1~\mbox{Hz} $
		POS	16.014	0.6331	661	$ 1~\mbox{Hz} $
VBB1	2017/05/09 06:39	VEL	15.274	0.5879	14730	$ 20~\mbox{Hz} $
		VEL	15.269	0.5877	3599	$ 1~\mbox{Hz} $
		POS	15.310	0.5903	1079	$ 1~\mbox{Hz} $
VBB3	2017/05/09 06:39	VEL	15.117	0.5930	12908	$ 20~\mbox{Hz} $
		VEL	15.074	0.5942	3520	$ 1~\mbox{Hz} $
		POS	15.151	0.5965	1204	$ 1~\mbox{Hz} $

**Fig. 84:**
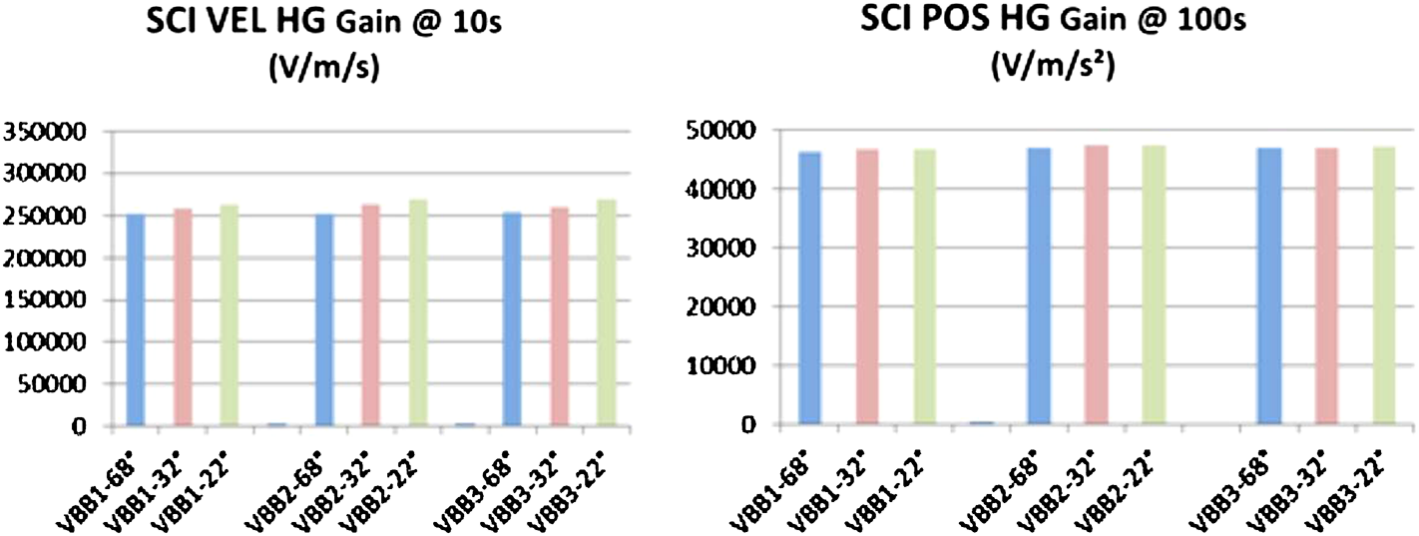
Dispersion of the gain measurements of the VBBs during coil calibration. Dispersions were ranging 4–6% for the VEL gain and 0.7–0.9% for the POS gain

To summarize the sweep experiment, we can say that in the frequency band covered by the sweep (1–0.01 Hz) a very simple analytical model of the transfer function is sufficient to describe the response of the VBBs to ground acceleration: a second order band-pass with only three free real parameters: generator constant (= gain factor), corner period, $T_{0}$ and damping, $h$. The differences in $T_{0}$ and $h$ between the POS and VEL channels can be taken as an indication of the error in these parameters. Relative errors for $T_{0}$ and $h$ range between 0.1–0.5%. This is mostly due to the elevated ambient seismic noise in the clean room at CNES/Toulouse where the Flight models were tested. We have more dispersions on the output gain measurement, assuming the generator constant of the VBB is known. When taking in account the expected variation of the gain as a function of the tilt configuration, dispersions were ranging between 4–6% for the VEL gain and between 0.7–0.9% for the POS gain. Results are shown in Fig. 84 and Table 15.

In similar experiments conducted in 2012 at BFO with commercial broad-band seismometers we found residues that correlated with the input sweep. For some of the sensors a residue with twice the instantaneous frequency of the sweep was prominent. Such frequency doubling is a clear sign of a quadratic or cubic non-linearity. No indication of such a non-linear response was found in the experiments analyzed here on the VBBs. However, we note that if the frequency calibrations had been conducted at a seismically quieter site small non-linear behavior might have appeared that remains hidden in the elevated seismic noise present on the CNES campus at Toulouse.

##### Temperature Sensitivity of the VBB Transfer Functions

**Fig. 85:**
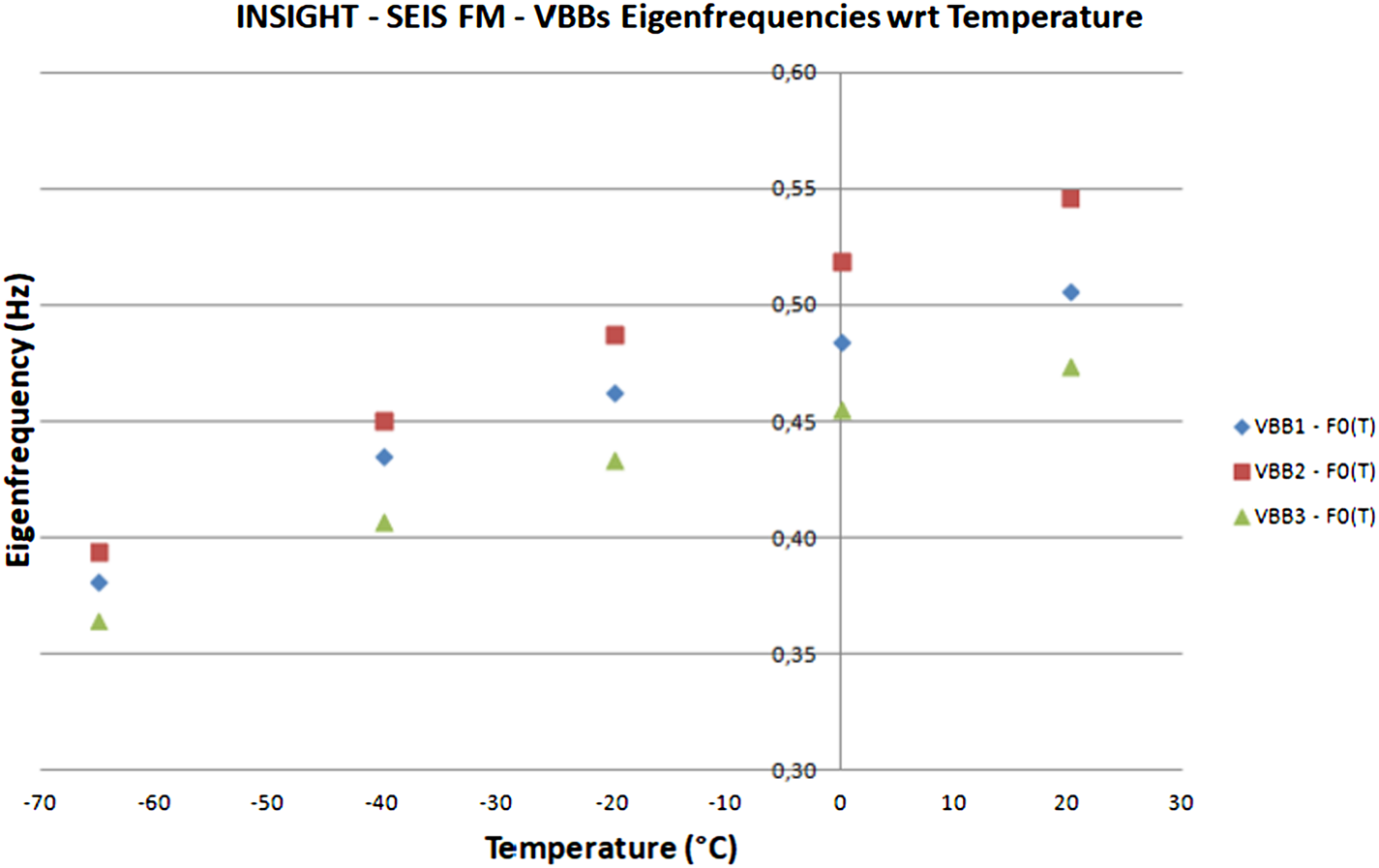
SEIS VBBs eigenfrequencies with respect to Temperature

**Fig. 86:**
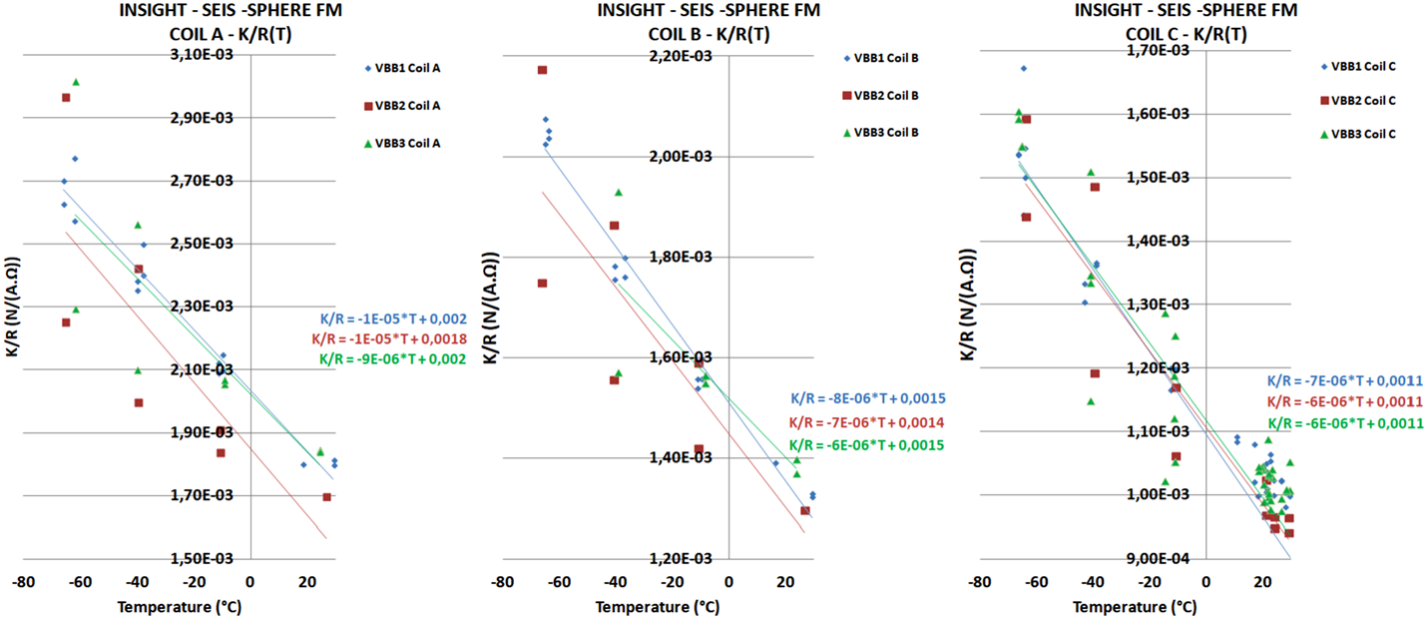
SEIS VBBs Magnetic actuators ratios of the force coefficient by the internal resistance with respect to Temperature. Coil A, B, C are the integrator, derivator and calibration coils respectively (see Sect. 5.1.5). Measurement errors remain large

**Fig. 87:**
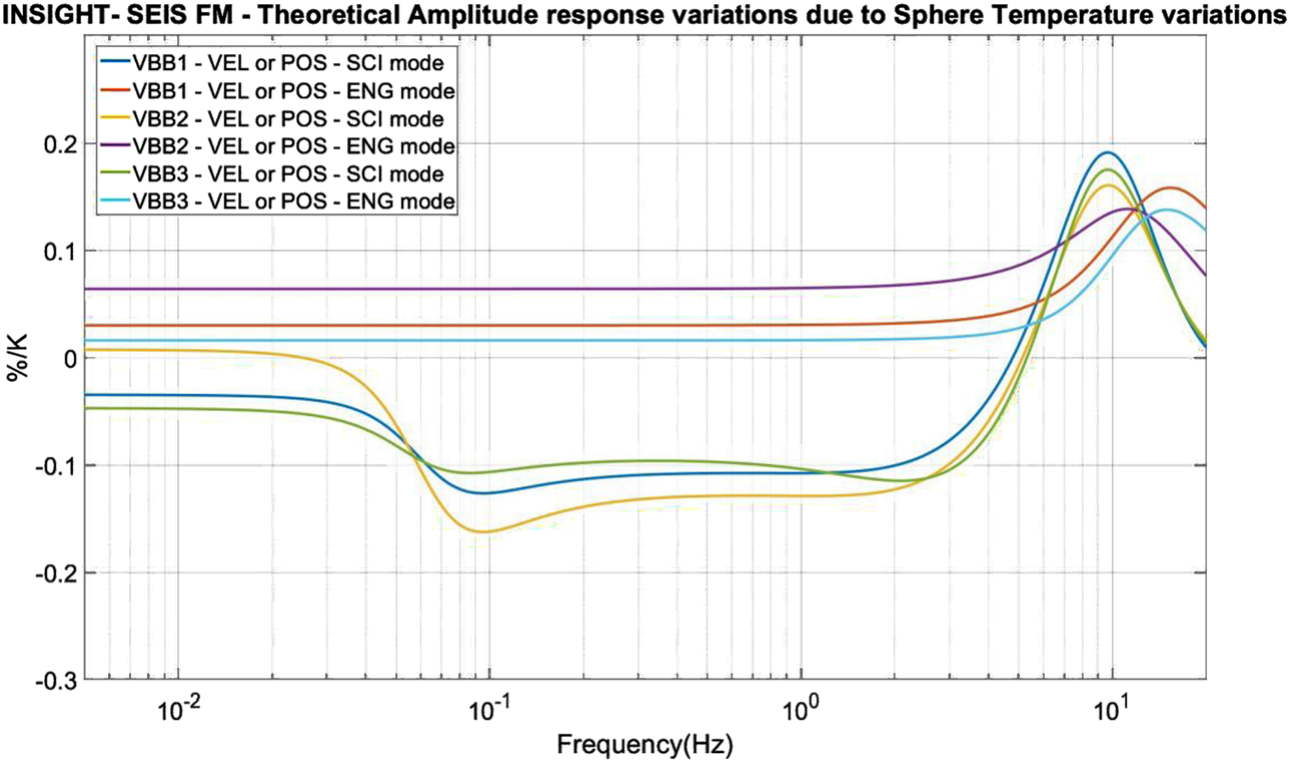
SEIS VBBs Theoretical VBBs Amplitude Response variation wrt Sphere Temperature from feedback actuator thermal sensitivity measurements. At periods larger than 100 s, the transfer function sensitivity is $<0.05\%/{}^{\circ}\mbox{C}$, leading to 2.5% over the 50°C climatic variation between the coldest and hottest temperature of the VBBs over one Martian year

**Fig. 88:**
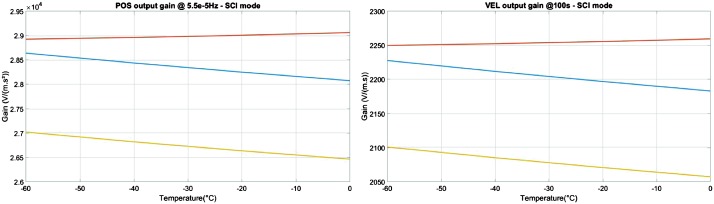
SEIS POS output at the Phobos Tide frequency and SEIS VEL output at 100 s, as a function of temperature. Temperature dependency will be recalibrated on Mars but can be considered as linear as a function of frequency and with a frequency dependency as indicated by Fig. 85. The information of temperature dependency will be encrypted in a SEED comment

We expect significant climatic and daily temperature changes on Mars and due to the temperature variations of the feedback actuators and of the natural frequency of the VBBs, the transfer function will vary with temperature. During VBB integration and the protoflight test program, special care has been taken to obtain pendulum thermal sensitivities over the complete temperature range. Pendulum thermal sensitivities were characterized before and after environmental tests ($\mbox{vibrations} + \mbox{thermal cycles}$) and also after integration in the sphere. The main parameters screened during the test program were:

- Pendulum eigenfrequencies: decreasing eigenfrequencies will increase the mechanical gain and the signal-to-noise ratio with a $1/f _{0} ^{2}$ law. Expected variations are $\pm 10\%$ ($0.5\%/{}^{\circ}\mbox{C}$) over the Martian climatic range, leading to a mechanical gain almost 50% larger in winter than summer (Fig. 85). Although minimized by the feedback, these eigenfrequency variations will generate variations of the transfer function, which are hopefully reduced as the variation is divided by the feedback strength to first order (about 80 at 0.07 Hz and 730 at very long period).
- Magnetic actuators parameters ($K$ (N/A)—force coefficient and $R$ ($\Omega$) internal resistor): The Feedback outputs drive these magnetic actuators. As the coil is made of copper, it is very sensitive to temperature changes. Due to thermal dilatations the geometry of these actuators changes also which may result in force coefficient changes. Modeling these effects is difficult since they are strongly related to the final mounting of the coil on the hardware and measurements of these efficiencies were performed Fig. 86. When injected in the instrument models, this enables prediction of the expected sensitivity of the transfer function for both the ENG and SCI modes described in Fig. 87 and Fig. 88. These sensitivities, based on all Earth tests, will of course be updated on Mars. Precise analysis based on long time series, like normal modes spectrum (expected on 6–12 hours times series) and tidal analysis will need to incorporate these effects for precise determinations of amplitude or frequencies. A simplified description of the temperature model will be documented in a comment blockette of SEED. See 0 for more details.
- The calibration coil actuator will be more critical as it will affect the in-situ calibration. We show on Fig. 86 and for the 3 coils, the temperature variation of $K/R$, where $K$ is the strength of the coil (in $\mbox{N}/\mbox{Amp}$) and $R$ the coil resistance (in ohm). For the calibration coil C, this is the parameter driving the coil calibration described in the previous section. For a given mode and to first order, the output of a calibration at a temperature $T$ will be the output of a calibration at temperature $T_{0}$ multiplied by the ratio of the $K/R$ at the two temperatures. This will enable interpretation of the transfer function found through calibration during the climatic variation, as well as comparison of these calibrations on Mars with those made at ambient temperature on Earth.

#### VBB Noise

**Fig. 89:**
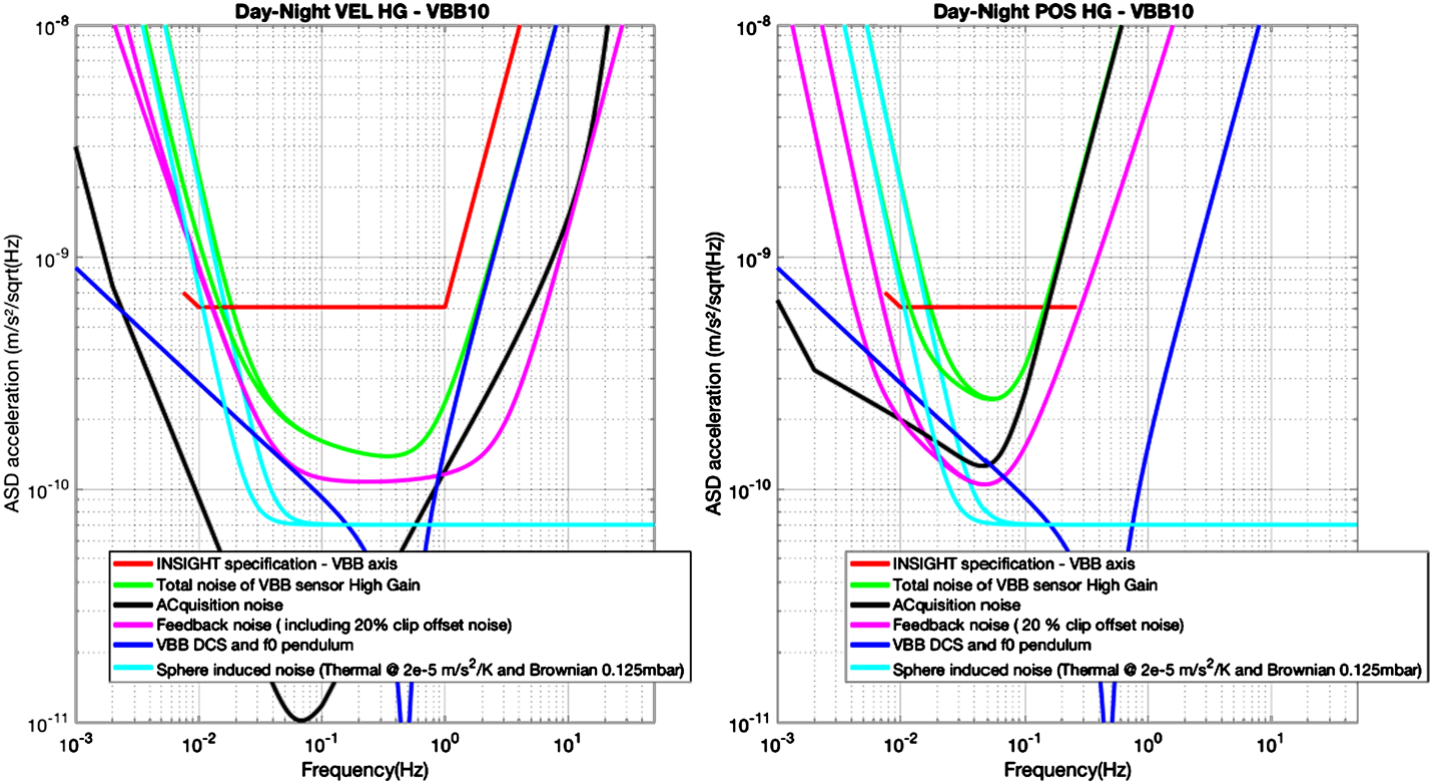
Noise model of one VBB axis in Mars conditions, for Night and Day conditions, for both the VEL (left) HG and POS (right) HG. For LG, the acquisition noise will be respectively 3.2 and 4.5 larger for VEL and POS output respectively

As indicated in Sect. 6.2.1 the operation of the Flight VBBs on Earth is challenging because of the difference of gravity and therefore and especially with the very low expected self-noise, the characterization of noise was also extremely challenging. A special care has therefore been used to elaborate on the self-noise model of the instrument, integrating all known sources of sensor noise and modeling the noise in a given test configuration (Fig. 89). This noise model has first been calibrated by noise measurements of all stages of the feedback in open loop (e.g. integrator, derivator, output gains, internal gains, DCS noise, Acquisition system, etc.), of the expected temperature sensitivities, both in terms of sphere and proximity electronic temperature variations and lander temperature noise for the FB cards. Note that the first two are related to thermal noise shielded by the WTS/TBK and Sphere and WTS/TBK and PE box respectively, while the second one is shielded only by the SEIS electronics box and depends on the thermal enclosure temperature noise. This noise model excludes the pressure and magnetic noise, which are in facts signals detected by the seismometer and potentially monitored by the APSS pressure sensor and IFG fluxgate magnetometer.

The noise model suggests that in Mars conditions, the long period noise on the VEL output will be driven by the temperature during the day, while there is the possibility that during night, the VEL output will have a noise driven by the feedback integrator noise, offering then better data on the POS HG output at long periods. The POS LG noise will be sensitive to the acquisition noise which is expected to be above the transducer noise, in contrary to the POS HG noise. VEL HG and VEL LG are however expected to have a very similar self-noise, unless very low noise levels at 1 Hz are found.

**Fig. 90:**
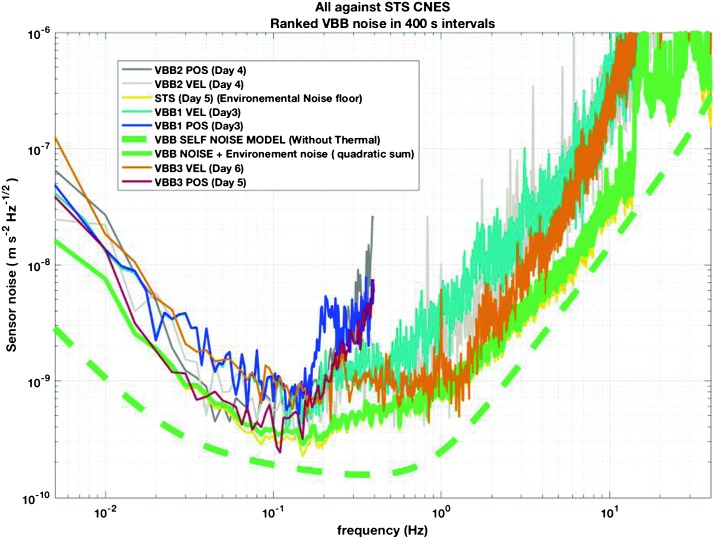
Results of tests made on the flight units in the CNES clean room, for both the POS and VEL outputs. The yellow curve shows the noise between two STS-2, which is significantly above the VBB noise model. The dashed green continuous curve is the VBB noise model while the continuous green curve is the quadratic sum between the VBB self-noise and the observed STS-2 noise, which might be more representative to the noise of the VBB with respect to a STS-2. Some of the VBBs measurements are very close to the environmental limits, despite the lower quality installation of the VBBs on the goniometer compared to the better installation quality of the STS-2

Tests made on the VBB Flight units in the CNES clean rooms are shown on Fig. 90. Testing conditions were impacted by an important environmental noise, which was characterized by cross-correlation between two STS-2s and is shown as the solid yellow line along the VBB measurement direction. This practically limited the self-noise of the VBBs to this environmental noise. Nevertheless, noise levels ranging from $5\times 10^{-10}~\mbox{m}/\mbox{s}^{2}/\mbox{Hz}^{1/2}$ to $10^{-9}~\mbox{m}/\mbox{s}^{2}/\mbox{Hz}^{1/2}$ can be detected between 0.05 Hz and 1 Hz. At high frequencies, noise levels are larger than those recorded by the STS-2s. Such larger noise levels are also recorded on the SPs, as seen in Fig. 95.

**Fig. 91:**
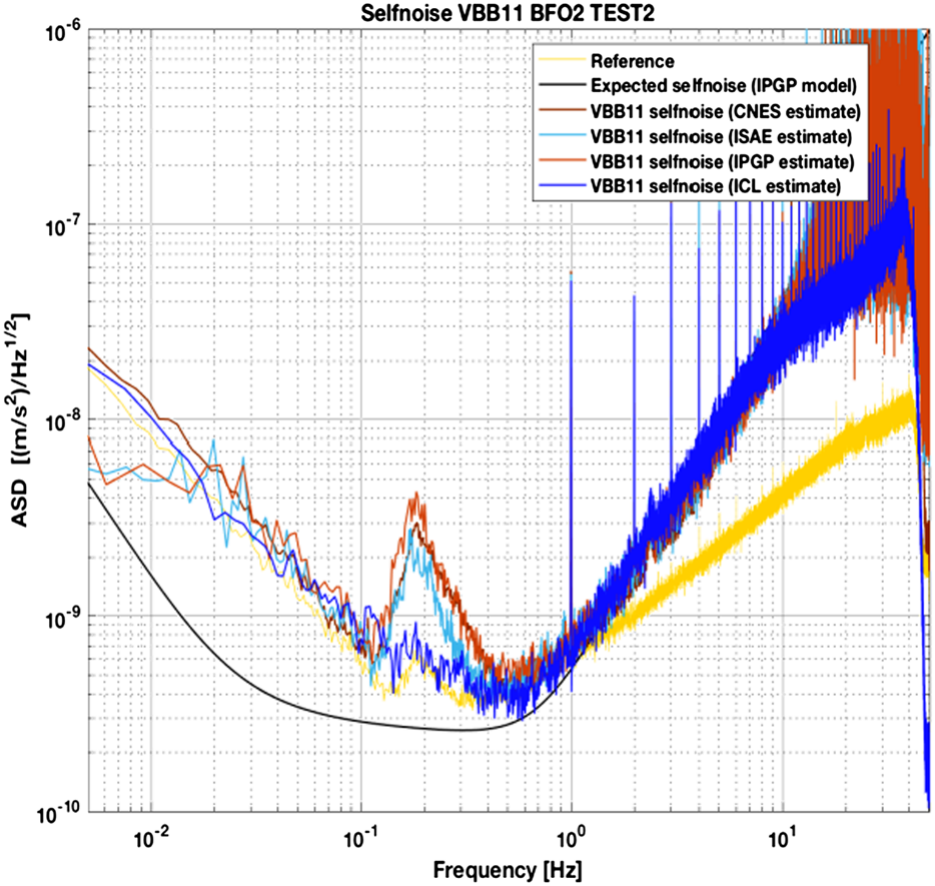
Noise measurement of the VBB in Earth configuration at BFO

**Fig. 92:**
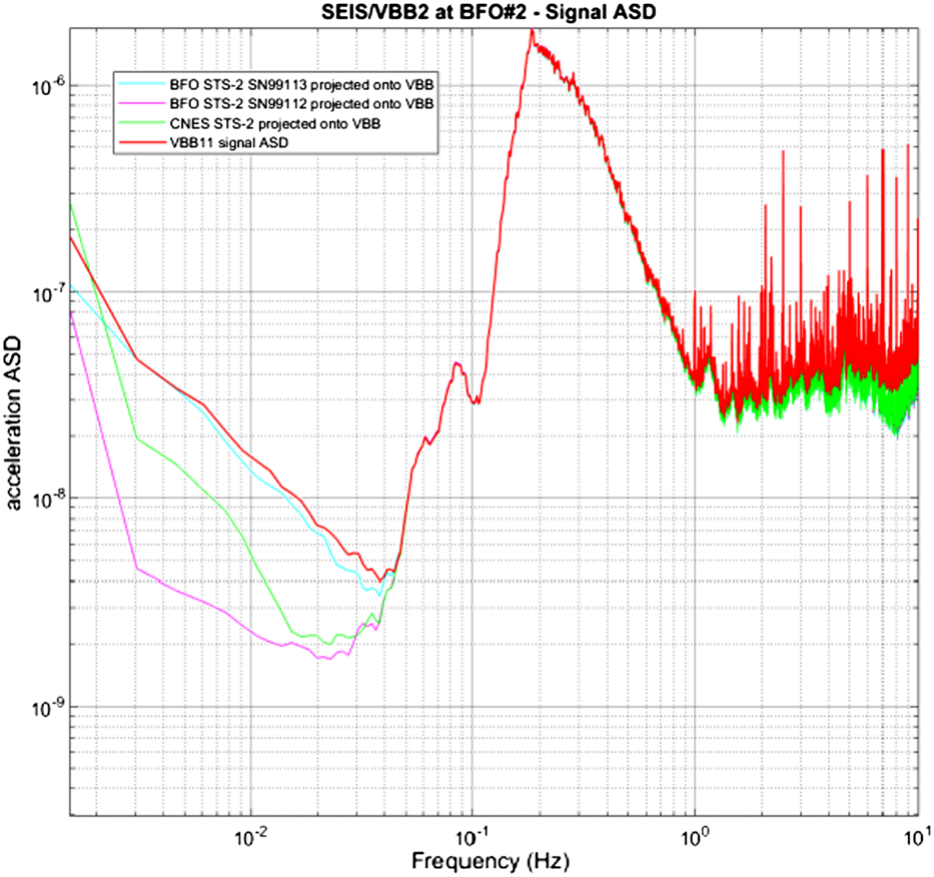
Comparison of three closely colocated STS-2s at BFO

**Fig. 93:**
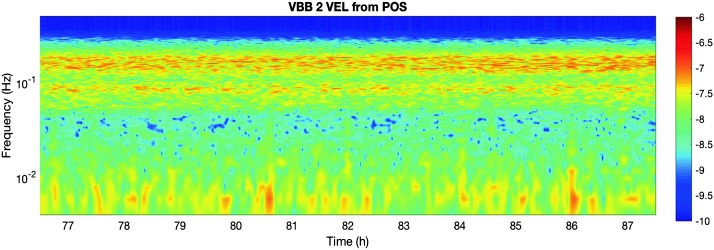
Spectrogram of the VBB output in ground acceleration, during 10 hours of BFO tests with Earth configuration. The series of events seen at frequencies smaller than 0.01 Hz are likely related to pressure transient variations associated with the leak rate of the vacuum chamber used for the test and whose spectrum might be proportional to $f^{-1}$. The amplitudes shall be compared to those recorded by the STS-2s shown on Fig. 91

Results of tests performed in the Black Forest Observatory (BFO) with the complete EM system and with the VBB in Earth configuration are shown in Fig. 91. In this configuration, the Earth VBB was operating in a small vacuum chamber. At high frequencies above 0.5 Hz, noise is matching fairly well the Earth VBB noise level and remains below the BFO noise recorded by a reference STS-2 almost up to 10 Hz. It reaches a noise level of $2\times 10^{-9}~\mbox{m}/\mbox{s}^{2}/\mbox{Hz}^{1/2}$ at 2 Hz and $5\times 10^{-9}~\mbox{m}/\mbox{s}^{2}/\mbox{Hz}^{1/2}$ at 3–4 Hz. These noises are directly related to the Displacement Transducer noise and as noted in Sect. 6.2.1, will be reduced by a factor of 2.65 on Mars, which suggests that the VBB has a comparable noise as the SP at 3–4 Hz. At long periods, significant variability of the noise, as measured by STS-2s, was found on the tilted direction along the Earth VBB axis (Fig. 92). The only differences of these three STS-2s, which recorded noise levels at 100 seconds ranging from $2\times 10^{-9}~\mbox{m}/\mbox{s}^{2}/\mbox{Hz}^{1/2}$ to $10^{-8}~\mbox{m}/\mbox{s}^{2}/\mbox{Hz}^{1/2}$, were their thermal protections and locations on the seismic pillar on which the SEIS was located. This illustrates the challenge of such performance tests, especially in a controlled schedule context, but might also not be so surprising as a $10^{-8}~\mbox{m}/\mbox{s}^{2}/\mbox{Hz}^{1/2}$ noise level is equivalent to an instrument tilt of about $1.2~\mbox{nano-radian}/\mbox{Hz}^{1/2}$ which might be easily induced by convection forcing on the instrument. Further analysis identified that the recorded VBB noise was likely related to the temperature noise induced by pressure transient variation in the small vacuum chamber (Fig. 93).

In summary, the analysis of all tests demonstrated performances below $10^{-9}~\mbox{m}/\mbox{s}^{2}/\mbox{Hz}^{1/2}$ between 0.04 Hz and 1 Hz with a noise floor smaller than $5\times 10^{-10}~\mbox{m}/\mbox{s}^{2}/\mbox{Hz}^{1/2}$ between 0.1 Hz and 1 Hz. Earth tests at long period were only able to reach a noise of $10^{-8}~\mbox{m}/\mbox{s}^{2}/\mbox{Hz}^{1/2}$ at 100 s. At 0.01 Hz, all open loop measurements of the VBB feedback and transducers were within the requirements with a modeled noise of $2\times 10^{-9}~\mbox{m}/\mbox{s}^{2}/\mbox{Hz}^{1/2}$ at 100 s for Earth configuration. If this was a non-modelled electronic noise, it will correspond on Mars to a noise of about $4\times 10^{-9}~\mbox{m}/\mbox{s}^{2}/\mbox{Hz}^{1/2}$ but is most likely noise related to the environment and Earth testing conditions

### SPs Results

#### SP Transfer Functions Calibration

**Fig. 94:**
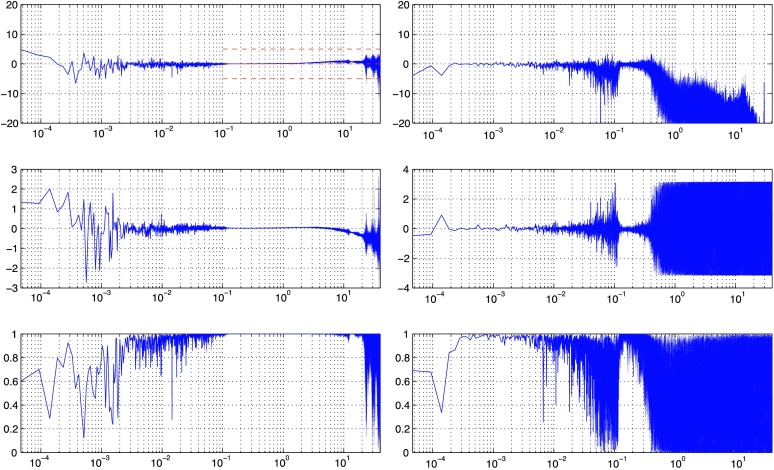
The transfer function (TF) of SP determined from coherence testing shown in amplitude and phase. The requirement of $\pm 5~\mbox{dB}$ flatness within the bandwidth of SP is shown. The TF determination is valid within the bandwidth where the coherence is high, allowing extension of SP meeting the TF requirements to 0.006 Hz

The TFs of the SP flight units have been quantified using coherence testing against a known reference seismometer. This has confirmed the expected form (see Fig. 53) with the gain, poles and zeroes adjusted to give a corrected flat response in the SP velocity output (Fig. 94). The fitted TF parameters are shown in Table 16. The TF determination is valid when the coherence is high, allowing extension of the 5 dB requirement beyond the 0.1 Hz requirement to 0.002 Hz.

**Table 16:** Transfer function parameters of the SP outputs

	SP1	SP2	SP3	Unit
Gain	27,600	28,100	24,500	V/(m/s)
Zeroes	0	0	0	Hz
	0	0	0	Hz
Poles	−0.018231	−0.018388	−0.018592	Hz
	−0.044269	−0.044649	−0.043164	Hz
Corner	35.2	34.9	35.3	S
Damping	1.1	1.1	1.09	No unit

SP Mass position is in turn validated against the SP velocity output, differentiated to acceleration. No correction has been applied to the TF determined in Fig. 94, apart from an MPOS gain, in $\mbox{V}/(\mbox{m}/\mbox{s}^{2})$ selected to give a 0 dB output. The uncorrected high-frequency roll-off in MPOS is evident with a corner frequency of 0.4 Hz.

#### SP Self Noise

**Fig. 95:**
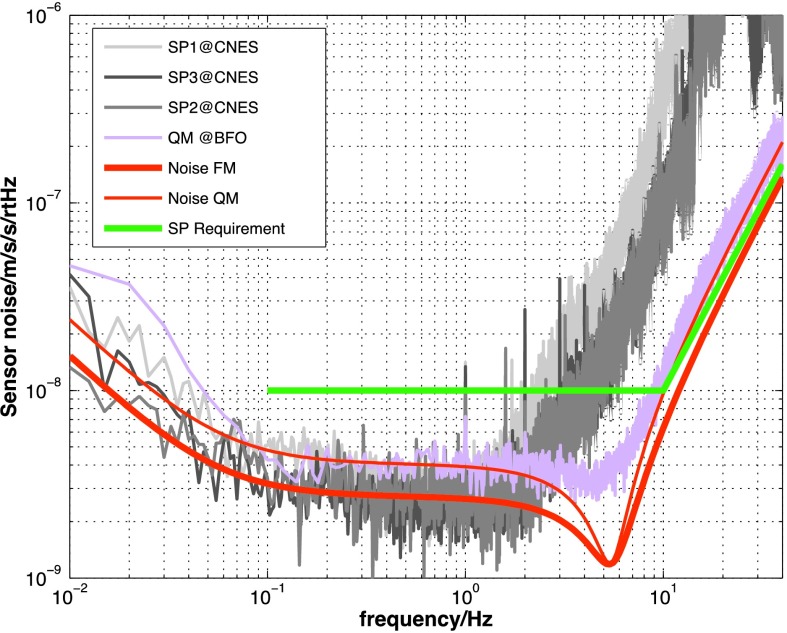
Self noise of the flight model (FM) SP’s as determined by coherence testing on the SEIS instrument assembly at CNES Toulouse, together with the self-noise of a qualification model (QM) unit determined at the lower noise Black Forest Observatory (BFO). The SP performance requirement is marked as well as the noise models for the FM and QM units. All SPs are at least a factor of 2 better than their requirements at 0.1 Hz

The performance floor of the SP is set by the internal self-noise of its sensors and the aseismic noise from the environment transduced into the SP’s output. The self-noise was determined by coincidence testing (Holcomb 1989) against a conventional broad-band seismometer with a noise floor at least an order of magnitude lower. This allows the self-noise of the sensor to be attributed to any loss of coherency, subject to minimization of any common environmentally induced terms in the signal. Figure 95 shows the self-noise of the flight-model (FM) SPs determined by coincident testing at two sites, one with a low-ambient noise above 1 Hz and the second with better coupling to the reference seismometer at lower frequencies. The sensor is very close to the fundamental thermodynamic limits, for this sensor of $0.2~\mbox{ng}/\sqrt{\mbox{Hz}}$. The expected higher sensitivity at resonance, down to $0.1~\mbox{ng}/\sqrt{\mbox{Hz}}$ is challenging to validate, but the shaping of the noise through the transfer function of the suspension is clearly seen. The SP performance requirement is marked as well as the noise models for the FM and QM units. All SPs are at least a factor of 2 better than their requirements at 0.1 Hz, in either amplitude or frequency.

### Temperature and Tiltmeters

#### Temperature Sensors

SEIS instrument includes more than twenty temperature sensors distributed on each subsystem mainly for housekeeping purposes. Most of them are based on standard Class B PT1000 probes calibrated by the manufacturer for which generic calibration can be used. Several were however calibrated and the calibration method and results are provided in this section.

The number of sensors allows mapping the temperature of the instrument which is a key point for seismometer performance. The temperature sensors used to monitor the health of SEIS are digitized with a resolution of 12 bits while the sensors dedicated to science on VBBs and Levelling system feed respectively a 16 and 24 bit ADC for better resolution.

**Fig. 96:**
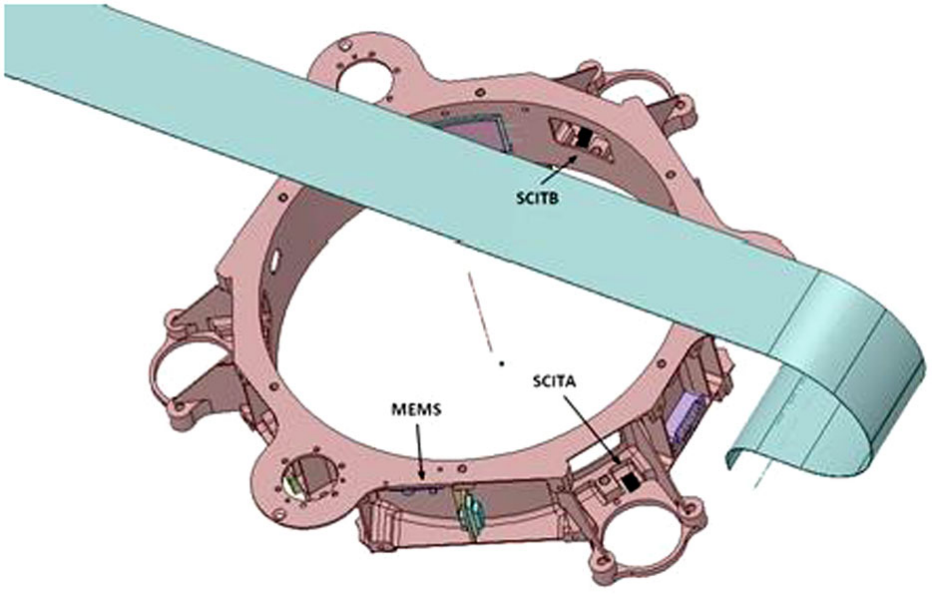
Position of SCIT A&B on the LVL subsystem

The SCIT A&B (Scientific temperature) sensors are placed on the LVL system as shown in Fig. 96. To meet the resolution and accuracy requirements the sensors were selected from 16 thermistors based on their linearity characteristics. This step was done by performing a comparison test between a reference thermometer HART SCIENTIFIC 159 (which is calibrated at French National Calibration Laboratory) and each PT1000 thermistor. The polynomial coefficients describing the behavior of the thermistors were taken into account by the acquisition system HART SCIENTIFIC 2590 used to interface the thermistors. Then, we measured the difference between temperature reference and each PT1000 in order to make the best choice.

The CNES calibration lab test benches used for covering the temperature range were:

- A specific climatic chamber for temperatures from 0°C to $-120^{\circ}\mbox{C}$ SANYO MDF-1156
- A dry heat chamber for temperatures from 0°C to 50°C ISOTECH Europa-6 Plus

**Fig. 97:**
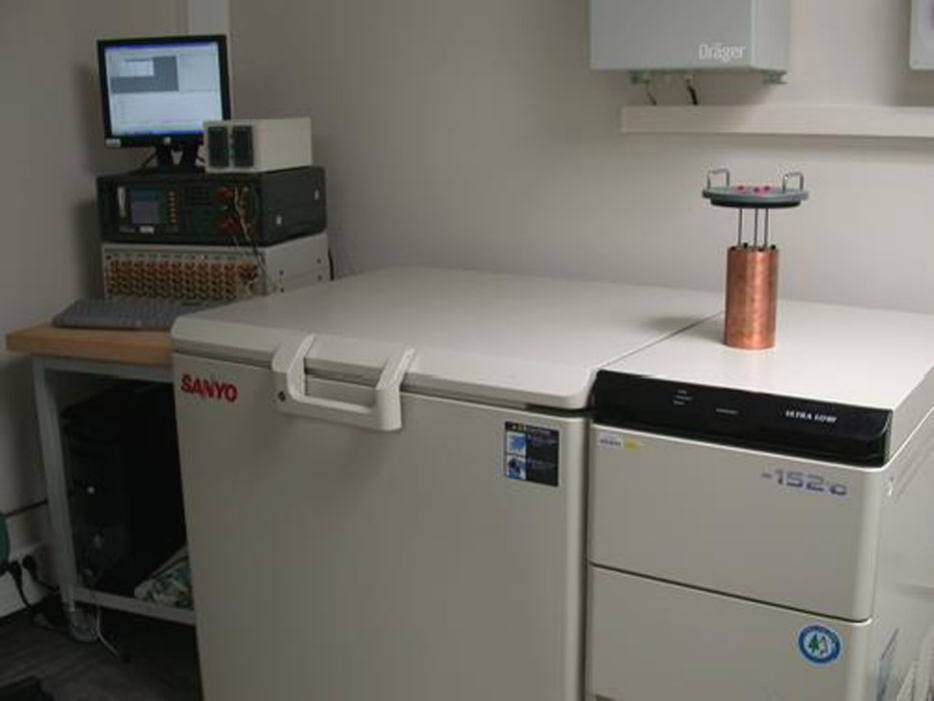
HRTS-5760-B-U-0-12 sensor head

**Fig. 98:**
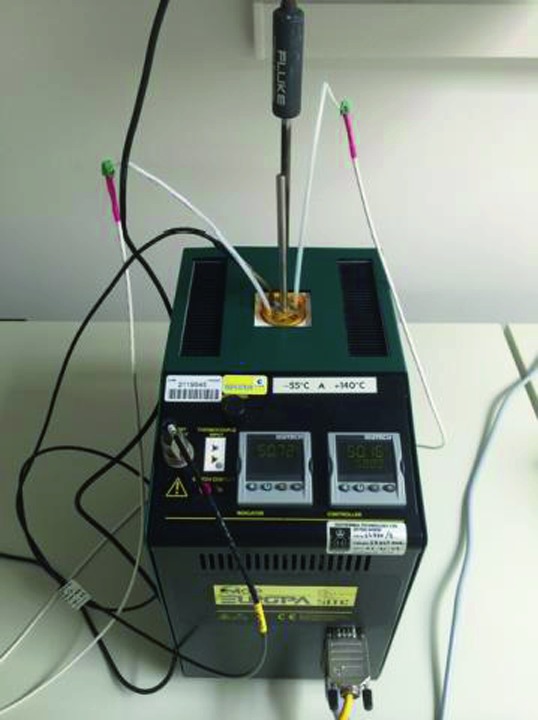
Dry Heat chamber

Since the flight model sensors could not be calibrated in the standard calibration baths, the calibration was performed in dry air with a very high constant time copper cylinder (see Fig. 97 and Fig. 98).

The science thermal sensors measurement chain includes not only the sensor itself but also a large portion of the tether which can add a parasitic resistance despite the 4 wires method used. In addition, the EBOX contains electronics devices which can affect the accuracy of the measurement. In consequence an end to end calibration was performed using very high stability resistors to simulate the PT1000 behaviour (a full chain calibration including sensor and measurement chain was not possible due to cleanness constraints).

The calibrated sensors have a response which can be fitted with a 3rd order polynomial:

17 $$ T=a x^{3} +b x^{2} +cx+d, $$

where $T$ is the temperature in degree Celsius and $x$ the raw data in bits and with values in Table 17.

**Table 17:** Transfer Function polynomial coefficients of the VBB Temperature sensors

Part #	*a*	*b*	*c*	*d*
SCITA	−6.91742 × 10^−22^	1.16867 × 10^−13^	1.98544 × 10^−5^	−247.538
SCITB	−1.60749 × 10^−21^	1.43078 × 10^−13^	1.97121 × 10^−5^	−248.043
VBB1_TEMP	−5.92947 × 10^−15^	3.03717 × 10^−9^	3.20300 × 10^−3^	−190.556
VBB2_TEMP	−5.92426 × 10^−15^	3.03505 × 10^−9^	3.20217 × 10^−3^	−190.495
VBB3_TEMP	−5.92295 × 10^−15^	3.03562 × 10^−9^	3.20159 × 10^−3^	−190.678
PRT	−2.66477 × 10^−9^	1.97266 × 10^−5^	0.230808	−247.992

#### Tiltmeters

**Fig. 99:**
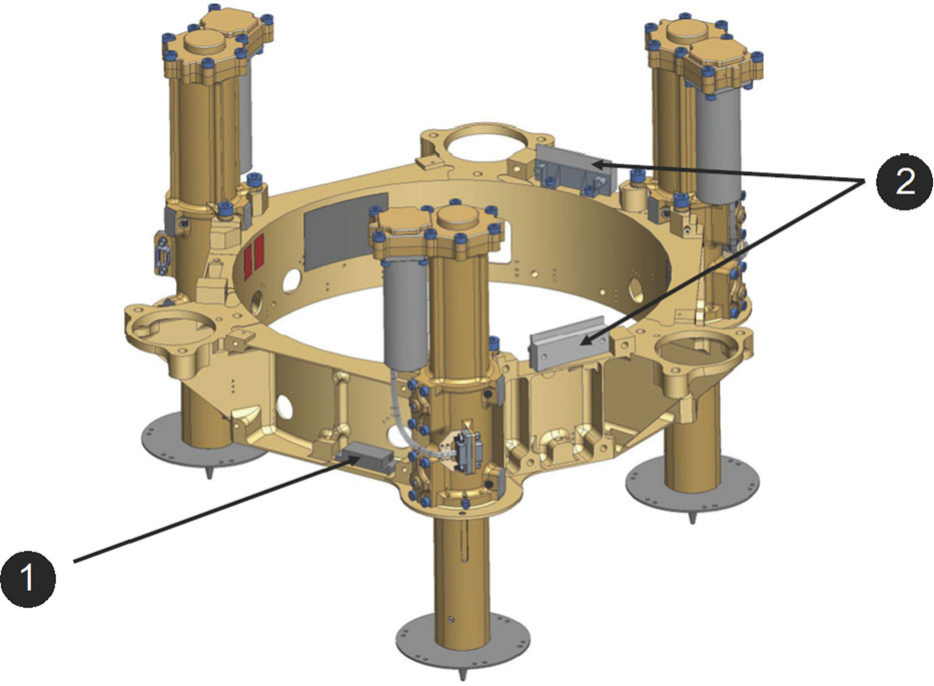
Location of MEMS (1) and HP tiltmeters (2) on the LVL

The levelling system carries all the SEIS sensors and maintains the instrument horizontally on the Mars surface. For that, the LVL includes coarse (MEMS) and precise tiltmeters (HP Tiltmeters) to measure the tilt of the instrument on the regolith (relative to the local gravity direction). Their location is indicated in Fig. 99.

##### MEMS Sensors

The MEMS Tilt sensor is based on an ADXL203 built by Analog Devices. This is an high precision, low power, complete dual-axis accelerometer with signal conditioned voltage outputs, all on a single, monolithic integrated circuit. The ADXL203 measures acceleration with a full-scale range of $\pm 1.7~(\mbox{Earth})~\mbox{g}$. The typical noise floor is $110~\upmu\mbox{g}/\sqrt{\mbox{Hz}}$, providing a rms noise of 0.44 mg over a 16 Hz bandwidth. This corresponds to 0.025° of inclination on Earth and 0.067° on Mars, for tilt sensing applications. This sensor is connected through the LVL tether to the Motor Driver electronics card (MDE) which amplifies the 2 analogue signals provided by the 2 axes sensor. These two signals are digitized on the MDE board after low pass anti-aliasing filtering to reduce noise before amplification. The only internal FPGA data processing consists in an averaging and a measurement on a [$\pm 15^{\circ}$] range is ready to be read out by the E-Box. Note that an absolute error of $0.1^{\circ}$ around zero degree of tilt on this measurement is required to be able to balance the VBBs properly.

##### HP Tiltmeters Sensors

**Fig. 100:**
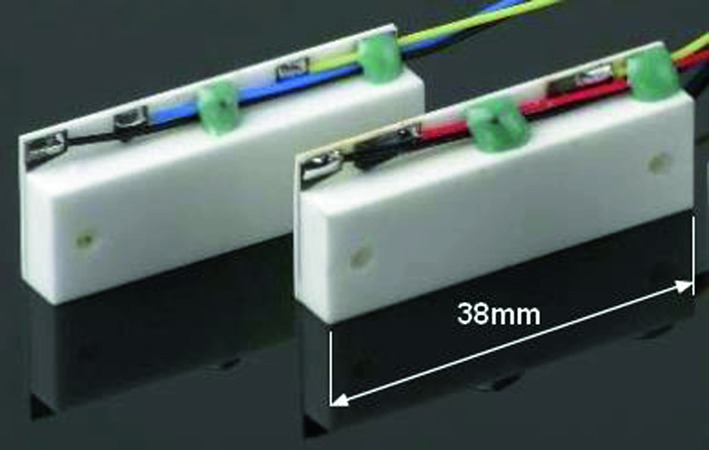
SH 50055 Family Sensor

The component used to perform an accurate measurement of tilt is a SH 50055-A-031 Electrolytic Tilt Sensor from Spectron (Fig. 100). This is a modified version of a SH 50055-A-009 commercial version component which includes changes to the electrolytic fluid and wire. This allows to enhance the temperature range to be compliant with requirements ([$-65^{\circ}$ to $+125^{\circ}$] for operating, [$-120^{\circ}$ to $+150^{\circ}$] for storage). After temperature compensation, the accuracy of the sensors is better than 3% over the $\pm 0.2^{\circ}$ range.

**Fig. 101:**
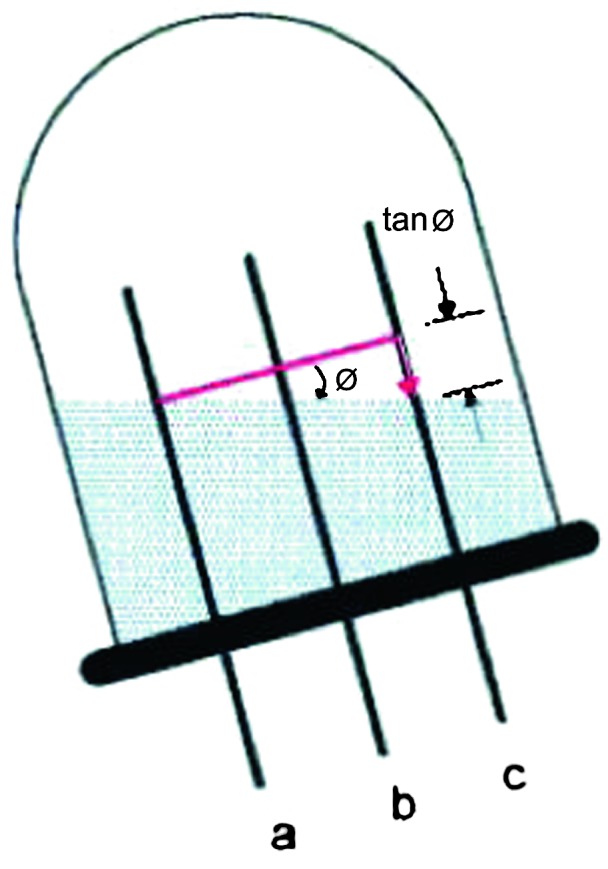
Working principle of the HP sensors

Figure 101 shows the principle of the electrolytic tilt sensor. As the sensor tilts, the surface of the fluid remains levelled due to gravity. The fluid is electrically conductive and the conductivity between the two electrodes is proportional to the length of electrode immersed in the fluid. At the angle shown, for example, the conductivity between pins a and b would be greater than that between b and c. Electrically, the sensor is similar to a passive potentiometer, with resistance changing in proportion to tilt angle.

**Calibration Process**

##### Calibration on Earth

The calibration functions of both sets of tiltmeters on the LVL depend on the sensor temperature. This dependence takes different shapes for the MEMS and the HP tiltmeter due to their different sensor techniques. For the MEMS sensors, the gain of the transfer function is independent of temperature, while the measured value at zero inclination decreases or increases with varying temperature. For the HP tiltmeters in contrast, the measured value at zero inclination is independent of temperature, whereas the gain changes. This means that the measurement range and the smallest measurable change in tilt corresponding to one unit of HP tiltmeter output also depend on temperature, resulting in a higher sensitivity at lower temperatures.

The calibration of the tiltmeter was performed with the complete LVL flight model in a thermal oven. The basic layout of the test is to acquire the transfer function of a high precision tiltmeter by step-wise moving the LVL with the related Linear Actuator and measuring and recording the output of all four tiltmeter and the inclination of the LVL with a reference inclinometer. The test runs are repeated at various temperatures and the data processed against temperature. Although the MEMS tiltmeter only gets a small excitation in tilt, the offset shift with temperature is clearly seen. The difficulty with the test setup is that the reference inclinometer has to be kept at room temperature while the LVL temperature must be varied. This problem was solved using a metal profile beam going through a feedthrough of the thermal oven. This bar was fixed to the LVL and transmitted the inclination to the outside of the chamber where the reference inclinometer was placed. As reference, the WYLER BlueLEVEL inclinometer with $1~\upmu\mbox{m}/\mbox{m}$ (0.2 arcsec) resolution was used.

**Fig. 102:**
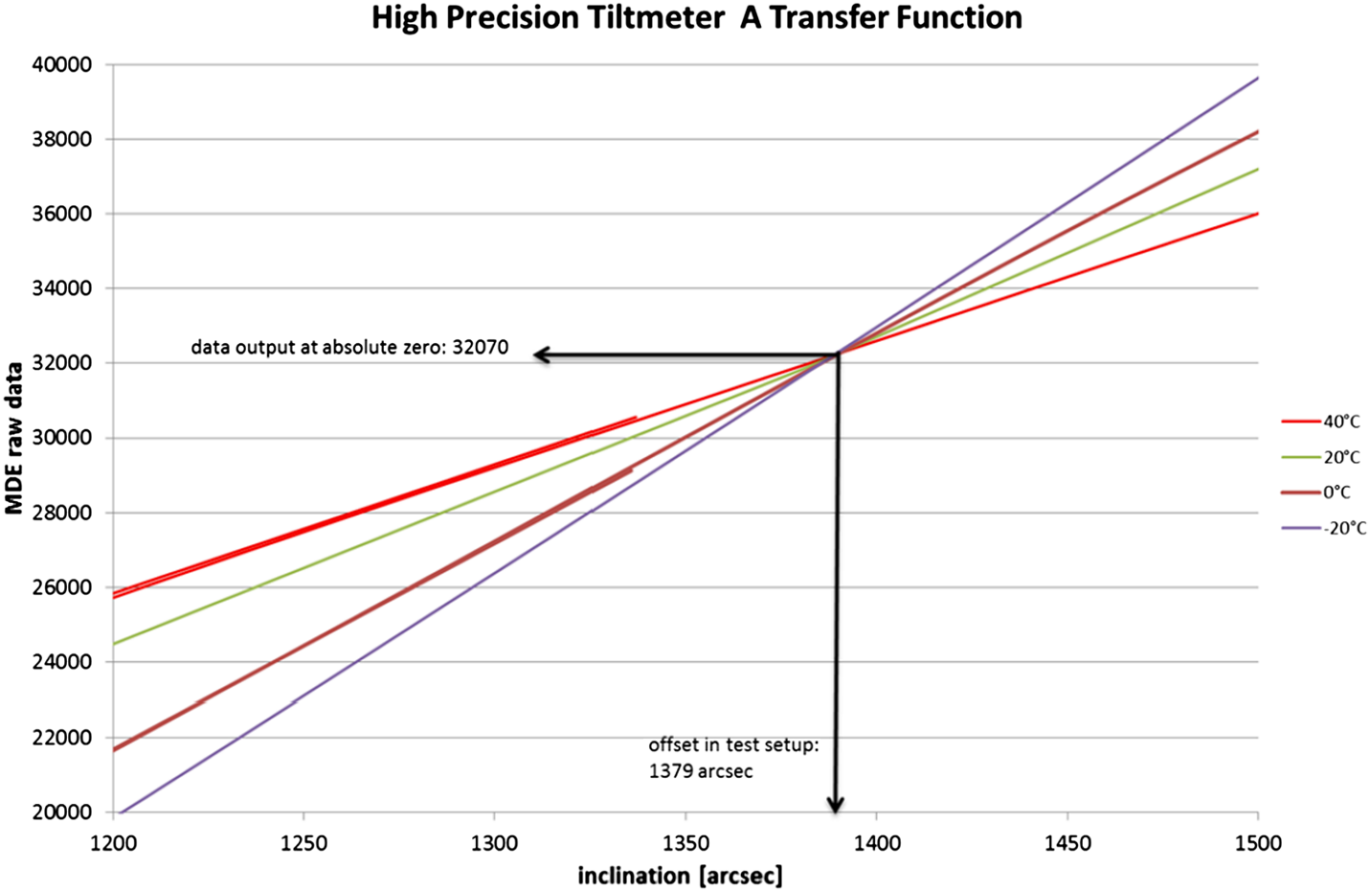
Temperature calibration curves for HP tiltmeter, measured at −20°C to 40°C in 20°C steps as described in the text

The results of the high precision tiltmeter show the absolute zero point for this axis (Fig. 102). At this point, the output signal is independent of temperature; the liquid is always in balance. With the coefficient of thermal expansion of the housing material and the manufacturing precision, the measurement range varies with temperature.

**Fig. 103:**
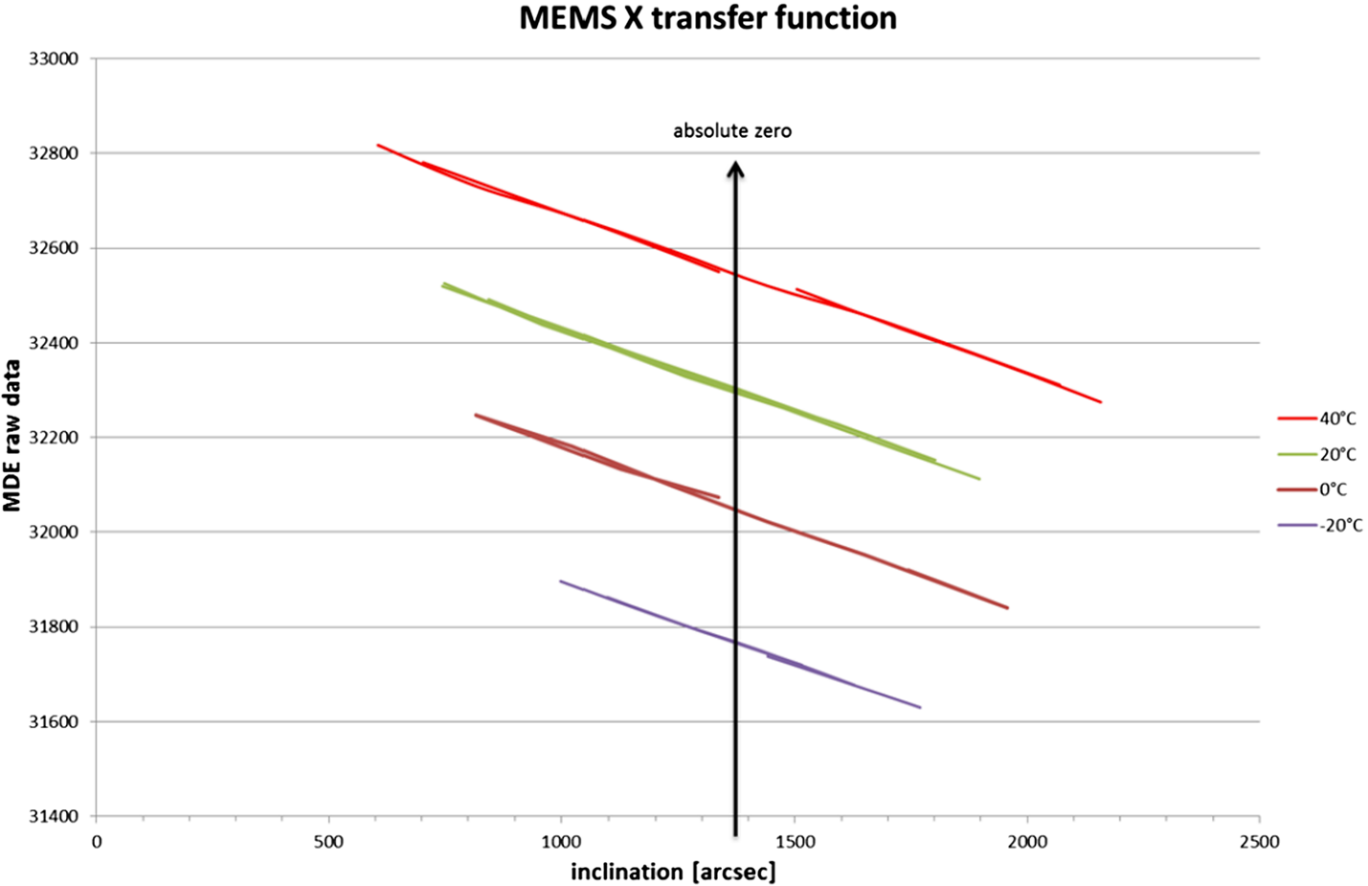
Temperature calibration curves for MEMS tiltmeter, measured at −20°C to 40°C in 20°C steps as described in the text

The MEMS output shows the variation of the offset against temperature while the slope of the transfer function curves remains constant (Fig. 103)

##### Calibration During Cruise

Assuming that zero gravity is equivalent to 0° of tilt of the MEMS proof mass, the transfer between Earth and Mars was an opportunity to assess the potential drift of the sensor. As a result, we plan to measure the offset for a given temperature two times during the cruise wake-up. If stable results are observed, we will be able to better extrapolate the value of the MEMS sensor offset on Mars.

Unfortunately, experiment under microgravity aboard the CNES Airbus zero-G demonstrated that this calibration will not be possible for the HP tiltmeters due to the measurement method based on a fluid displacement.

### LVL

As all ground motion is transferred to the SEIS sensors via the LVL, it is important to understand and characterize its possible influence on the recorded waveforms. The LVL transfer function was determined during different stages of integration, for increasingly flight-like configurations and a simplified analytical model of the LVL was developed. A more detailed description of the results and methodology can be found in Fayon et al. (2018). The actual LVL transfer function on Mars can only be determined once SEIS is deployed, though, as it depends on the deployment configuration (leg extraction) of the LVL as well as on yet unknown local soil properties at the deployment site.

The mayor way in which the LVL affects recorded signals is by horizontal resonances of the system due to the details of the leg structure. These resonances were first observed during forced excitation in a test of the LVL structure on a shaker with an input acceleration of 0.1 g, using a sweep signal between 5 and 200 Hz with a sweep rate of two octaves per minute. The resulting acceleration at various points of the LVL was recorded with miniature accelerometers glued to the LVL structure. The tips of the LVL feet were likewise glued to the shaker’s table, unlike the SEIS deployment configuration and the LVL legs were extended to an intermediate length. Due to the missing SA, the weight of the structure was significantly less than the flight weight at 5300 g. Measurements were conducted for acceleration both in X- and Y-direction. During acceleration in each of these directions, only accelerometers pointing in the same direction recorded any significant amplification within the whole frequency band covered. The resonance frequencies observed for sensors at different locations on the LVL are identical in each of the two configurations, with varying peak amplitudes on the order of 5 and comparatively broad peaks, with a plateau covering about 10 Hz. Peak frequencies are slightly shifted between X- and Y-direction and centered at 50 and 48 Hz.

A more thorough investigation of the seismic transfer functions was done in the MPS cleanroom, using a configuration typical in seismometer calibration (Holcomb 1989; Pavlis and Vernon 1994): We recorded ambient vibrations with a broad-band “test” sensor placed on the LVL and compared the data to that recorded by a “reference” sensor located on the ground close enough to assume that both sensors record the same ground motion. The used sensors are Trillium compact seismometers, connected to a six-channel 24-bit Centaur data logger. The final mass of the setup, including the seismometer and additional dummy masses, is 9082 g.

The transfer function was determined under a variety of surface inclinations in both X- and Y-direction, using a magmatic rock with a slope of 15° over a square area of $30 \times 30~\mbox{cm}$. In total, we performed measurements in 21 different configurations. As the LVL design is symmetrical with respect to tilts in ±Y-direction, only a limited number of measurements at the same angles in both +Y- and −Y-direction was conducted to confirm the symmetry. For each measurement, we calculated the power spectral densities for the three components of the reference as well as the test sensor. The orientation between the two sensors was adjusted by minimizing the incoherent noise in the frequency domain and the relative transfer functions calculated by division of the power spectral densities in the aligned system.

In all cases where the three legs are not of equal length, two different resonance frequencies occur, which, depending on configuration, either do or do not align with the X- and Y-axes of the system. Resonance frequencies lie between 34.7 and 46.4 Hz, depending on configuration. Lower resonance frequencies than observed during these measurements are possible if the LVL mass is higher than used here, if a high slope in both X and Y that we could not reach with our test equipment needs to be accommodated, or if all legs are extended equally to a large extent. The latter case is not foreseen for SEIS deployment.

Additionally, LVL resonances were determined for a more complete SA including LSA/tether during performance testing at CNES Toulouse. The measurement principle was the same as above and results are broadly consistent with those previously obtained, both for measurements on a solid surface and for measurements at three different tilted configurations on sand, using the LVL QM. A further measurement was conducted using actual horizontal SP sensors, at a comparable mass and partly extracted legs, which showed resonances around 40 Hz.

No measurement showed any clear LVL influence of the phase of the transfer function. Observed amplifications at the resonance peaks range between 10 and larger than 100, but the determined values depend on the coherence between the channels of the reference and test sensors more strongly than the measured resonance frequencies and are thus less certain.

The LVL also has an impact on high frequency measurements, i.e. the HP^3^ hammering, as it averages the ground acceleration sensed across the three feet and in this way acts as a low-pass filter (Kedar et al. 2017).

Modeling of the LVL is based on a method to detect and compensate for inconsistent coupling conditions during seismic acquisition (Bagaini and Barajas-Olalde 2007). Four main elements characterize the LVL model: one platform and three legs. Each 3D platform-leg coupling phenomenon is modelled by one vertical spring with a rigidity constant $k ^{p} _{v}$ and two horizontal ones with a representative constant $k ^{p} _{h}$. Likewise, each 3D foot-ground coupling phenomenon is described by constants $k ^{g} _{v}$ and $k ^{g} _{h}$. Equivalent masses for the platform subsystem $M _{p}$ and the three legs are used to complete the system. This configuration permits six degrees of freedom for each subsystem. However, as the complete instrument configuration does not allow for a vertical rotation of the legs, the final system in total has 12 degrees of freedom in translation and 9 in rotation.

Newton’s second law is applied for each part of the global structure in both translation and rotation. For example, for the LVL platform

18 $$\begin{aligned} M_{p} \frac{d^{2}}{dt^{2}} {{\overrightarrow{\Delta G_{p}}}} =& \sum_{i=1}^{3} \overrightarrow{\Delta F_{i}^{+}}, \end{aligned}$$

19 $$\begin{aligned} J_{p} \frac{d^{2}}{dt^{2}} \overrightarrow{\Omega _{p}} =& \sum_{i =1}^{3}\overrightarrow{G_{p}P_{i}^{+}} \times \overrightarrow{\Delta F_{i}^{+}}, \end{aligned}$$

The second derivative terms represent the platform’s center of mass acceleration in translation, in (18) and rotation, in (19) and $J _{p}$ is the platforms’ moment of inertia. $\overrightarrow{\Delta F_{i}^{+}}$ is the relative movement between the two ends of the spring on top of leg $i$ and $\overrightarrow{G_{p} P_{i}^{+}}$ corresponds to the vector between the platform’s center of mass and the top of the considered spring.

These equations are also written for each leg of the LVL structure. Combining all equations, the [$M$] and [$K$] matrices (size $21\times 21$) are defined and implemented numerically. This allows finding the eigenmodes of the global structure.

The adjustable parameters in the model are the various masses, the length of each leg, the stiffnesses of the springs, the torque induce by the ground on the legs $C ^{g} _{h}$ and the attenuation coefficient $Q$ of the ground. Once the extracted lengths of the LVL legs are known, this also sets their masses and the horizontal stiffnesses $k ^{p} _{h}$ between them and the platform. Values for $k ^{p} _{v}$ and $k ^{g} _{v}$ can be selected arbitrarily as tests show that they do not significantly influence the results. The main parameters to adjust because of their considerable influence on the calculated resonances are $k^{\mathrm{g}} _{{h}}$ and $C^{\mathrm{g}} _{{h}}$.

**Fig. 104:**
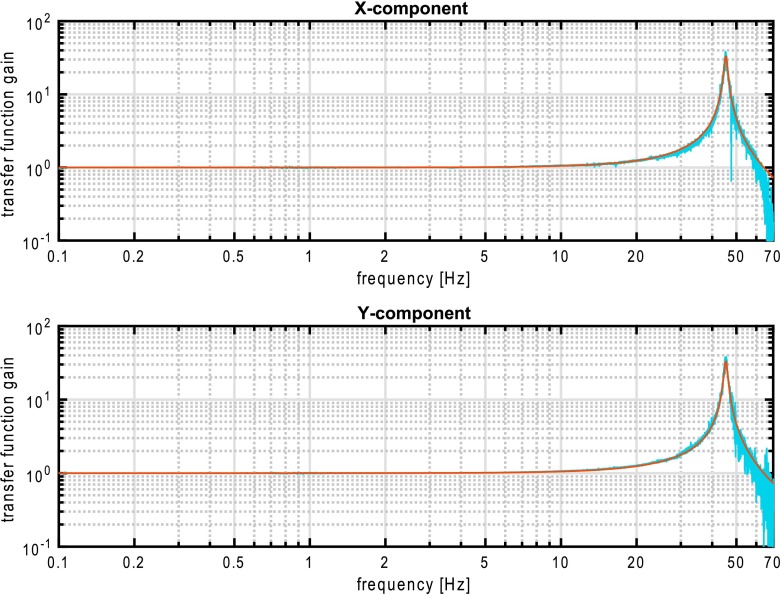
Measured (blue) and modeled (red) gain of the horizontal transfer functions in the LVL baseline configuration (all legs extended by 0.5 mm). Masses, leg lengths and values of $k^{p}_{h}$ of the model were set to those of the measurement, whereas parameters $k^{g}_{h}$, $C^{g}_{h}$ and $Q$ were adjusted to fit the data

When calculating all of the LVL’s 21 vibration modes (resonances and structure’s movements) with the analytical model, only two of the obtained frequencies are within the range covered by the measurements. They correspond to horizontal translations of the platform in X- and Y-direction, respectively, in good agreement with the laboratory results. A further validation of the model was done by changing either the mass of the platform or the leg lengths (same length for all three legs). When one of these parameters increases, the horizontal resonance frequencies decrease. The same effect is observed in the measured data and the model covers the same range of frequency values. The model can also describe the complete LVL transfer functions as determined during test measurements in the laboratory. Figure 104 shows an example for the baseline configuration (level low, with all legs at the same length). Our modeling indicates that the horizontal resonances of the LVL are highly dependent on ground properties. The model presented here could thus not only be used to predict at which frequencies SEIS measurements might be affected by LVL resonances, but also to invert for ground properties at the InSight deployment site once SEIS data from Mars are available.

### Thermal Protections

#### Thermal Objectives

The SEIS thermal protections aim to maintain all the elements of the instrument within Allowable Flight Temperature range all along the mission (Table 18), in operating, non-operating and start-up conditions, in deployed or stowed configurations.

**Table 18:** Allowable Flight Temperature range

Item	Operating		Non-operating		Start up
	$T_{\mathrm{min}}$	$T_{\mathrm{max}}$	$T_{\mathrm{min}}$	$T_{\mathrm{max}}$	$T_{\mathrm{min}}$
Sphere VBB	−65^∘^C	+30^∘^C	−90^∘^C	+40^∘^C	−65^∘^C
Linear actuator	−50^∘^C	+40^∘^C	−105^∘^C	+40^∘^C	−65^∘^C
LVL	−50^∘^C	+40^∘^C	−105^∘^C	+40^∘^C	−65^∘^C
Proximity electronics	−65^∘^C	+40^∘^C	−100^∘^C	+40^∘^C	−65^∘^C
SP sensors	−65^∘^C	+40^∘^C	−100^∘^C	+40^∘^C	−65^∘^C

Moreover, in order to guarantee the performance of the instrument, the environment temperature variations shall be filtered for VBBs and SPs measurements. This filtering is achieved thanks to high time constants of the system obtained through efficient thermal isolation. The meaning of the time constant is explained below.

Let us consider a system without internal heat dissipation at temperature equilibrium with its direct surrounding environment. If this system is exposed to a sudden external environment temperature step, the thermal time constant “$\tau $” of a system is the characteristic time defined by relation (17)

20 $$ T_{\mathrm{final}} - T_{B} ( t ) =( T_{\mathrm{final}} - T_{\mathrm{initial}} ) e ^{{-t}/{\tau }}, $$

where $T_{B}(t)$ is the system temperature at instant $t$ (°C), $T_{\mathrm{initial}}$ is the initial temperature of the system (before the temperature step) (°C), $T_{\mathrm{final}}$ is the final system temperature equal to the environment temperature step (°C). Two thermal time constants are specified for SEIS:

- between VBB and the sphere crown: this time constant shall be higher than 2 hours;
- between the sphere crown and WTS: this time constant shall be higher than 5.5 hours.

Other objectives of the thermal control are to guarantee the daily temperature stability ($<35^{\circ}\mbox{C}$ peak to peak for the sphere and $<45^{\circ}\mbox{C}$ for PE) and internal gradients ($<60^{\circ}\mbox{C}$ between VBB and crown).

#### Thermal Constraints

**Fig. 105:**
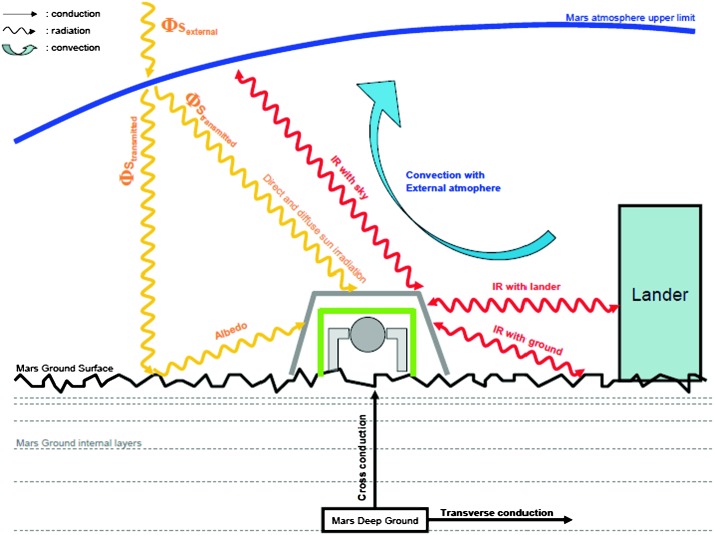
Schematic of the SEIS sensor assembly environment

The thermal control needs to deal with Martian environment: air and ground temperature variation, external heat flux (Sun, albedo and IR flux, convection). Wind and dust are key parameters for the heat flux exchanges. A model of the ground has been realized to account for the surface thermal equilibrium under the WTS dome (Fig. 105). Indeed, ground surface temperature is determined by thermal equilibrium under WTS since Martian regolith is a low conductive material.

#### Thermal Design

The instrument Thermal Control System (TCS) described hereafter has to ensure the following elements of thermal control:

- sphere and the 3 VBB;
- proximity electronic boxes;
- SP sensors;
- LVL (structural ring) and its associated linear actuators.

The electronic box (inside the lander) and the tether external part have dedicated thermal control. The Sensor Assembly thermal control is ensured thanks to several levels of protection (WTS, RWEB, Evacuated Container) as described in Sect. 5.6.

#### Validation (Model, Analysis and Tests)

##### SEIS Thermal Model

**Fig. 106:**
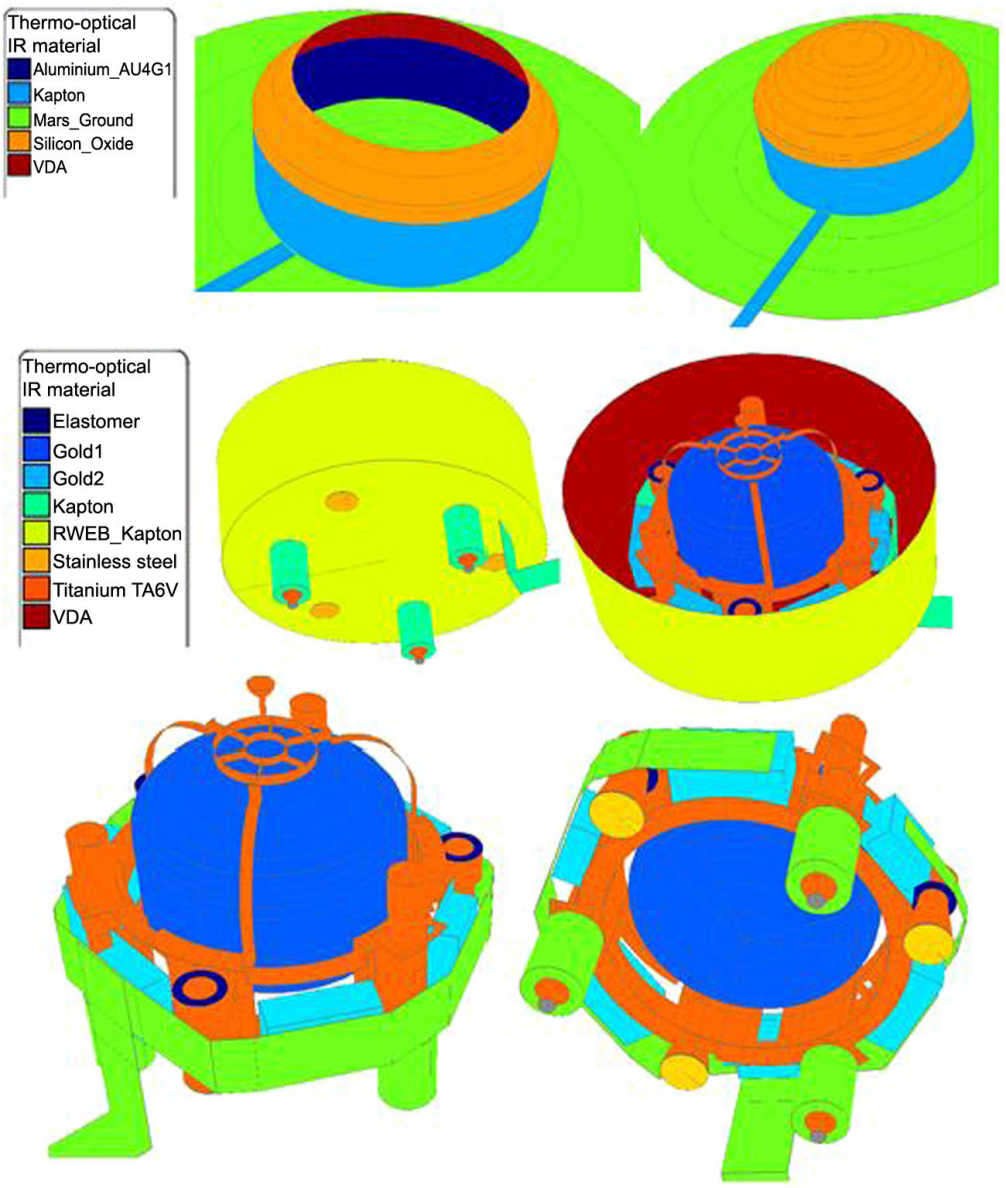
Views of the SA coatings

A detailed model of the sensor assembly (stowed and deployed configuration) has been built to perform thermal analysis in flight condition and demonstrate the performance of the design. SEIS sensor assembly model is composed of 5457 thermal nodes (5115 for the sphere). The model is built in Systema/Thermica format. Figure 106 summarizes the coating used in the SEIS instrument model.

**Fig. 107:**
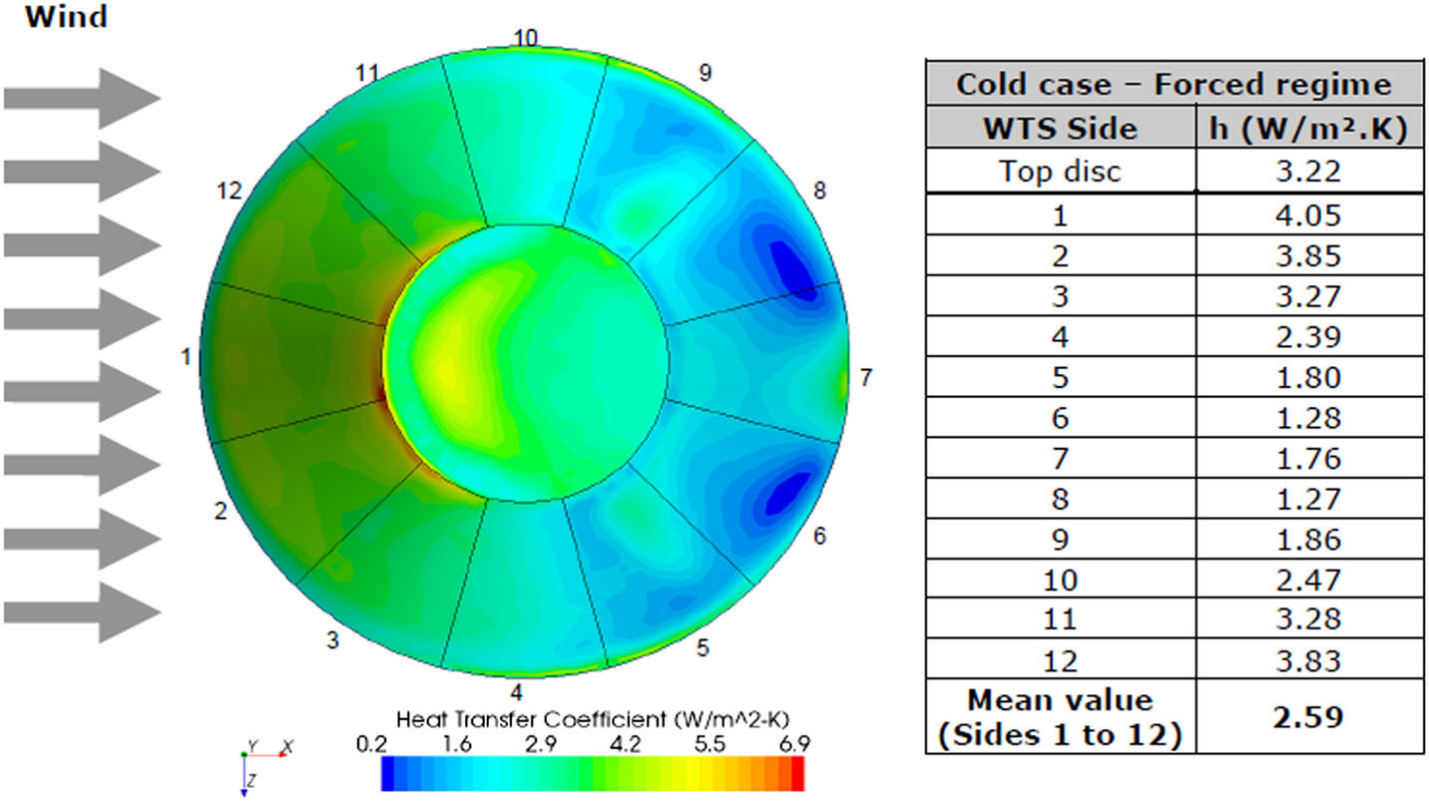
Results of CFD computation on WTS in cold case with $20~\mbox{m}/\mbox{s}$ wind

Particular attention has been paid to the convective couplings. For external convection, computational fluid dynamics studies have been conducted to estimate the equivalent convective coefficients by area (Fig. 107). Internal convective couplings are managed by linear conductors that have been updated following thermal balance tests.

Sensitivity studies on key parameters (including convective coefficients) allowed defining the model uncertainties.

Note that CFD computation remains a source of uncertainty because exchange coefficients have been estimated using a constant wind speed from one direction. However, the variation of WTS temperature shall have a minor impact on instrument units thanks to the multiple thermal isolation levels.

The thermal analyses confirm the compliance to all requirements and identified the critical cases: cold non-operating case on ground and on the lander. An interface thermal model has also been delivered to Lockheed Martin to complete thermal analysis at lander level.

##### SEIS Thermal Tests

Several of the thermal tests completed on the SEIS instrument were used for thermal validation:

- Thermal Balance Test on the Structural and Thermal Model to correlate the thermal model in 2013,
- Thermal Vacuum Test TVAC#4 to achieve the instrument qualification in thermal environment and verify the sphere thermal time constant in 2017,
- Thermal Balance/Thermal Vacuum Test on the lander (Landed TVAC) used by SEIS team to correlate the thermal model and validate the thermal time constants such as sphere and WTS time constant in 2017,
- TVAC#4.

**Fig. 108:**
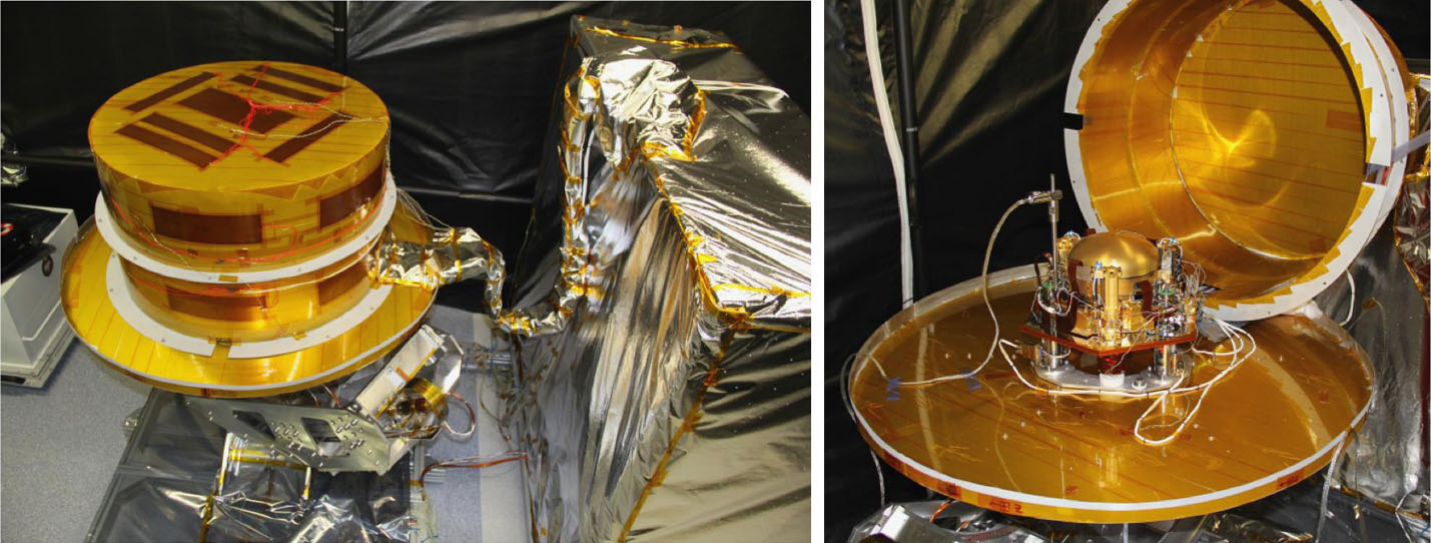
Photos of TVAC#4 configuration

Figure 108 show the TVAC#4 configuration: the SA was mounted within a thermally controlled cover installed on a TGSE (Thermal Ground System Equipment). This TGSE allowed tilting any of the VBBs up to 68° for functional tests.

**Fig. 109:**
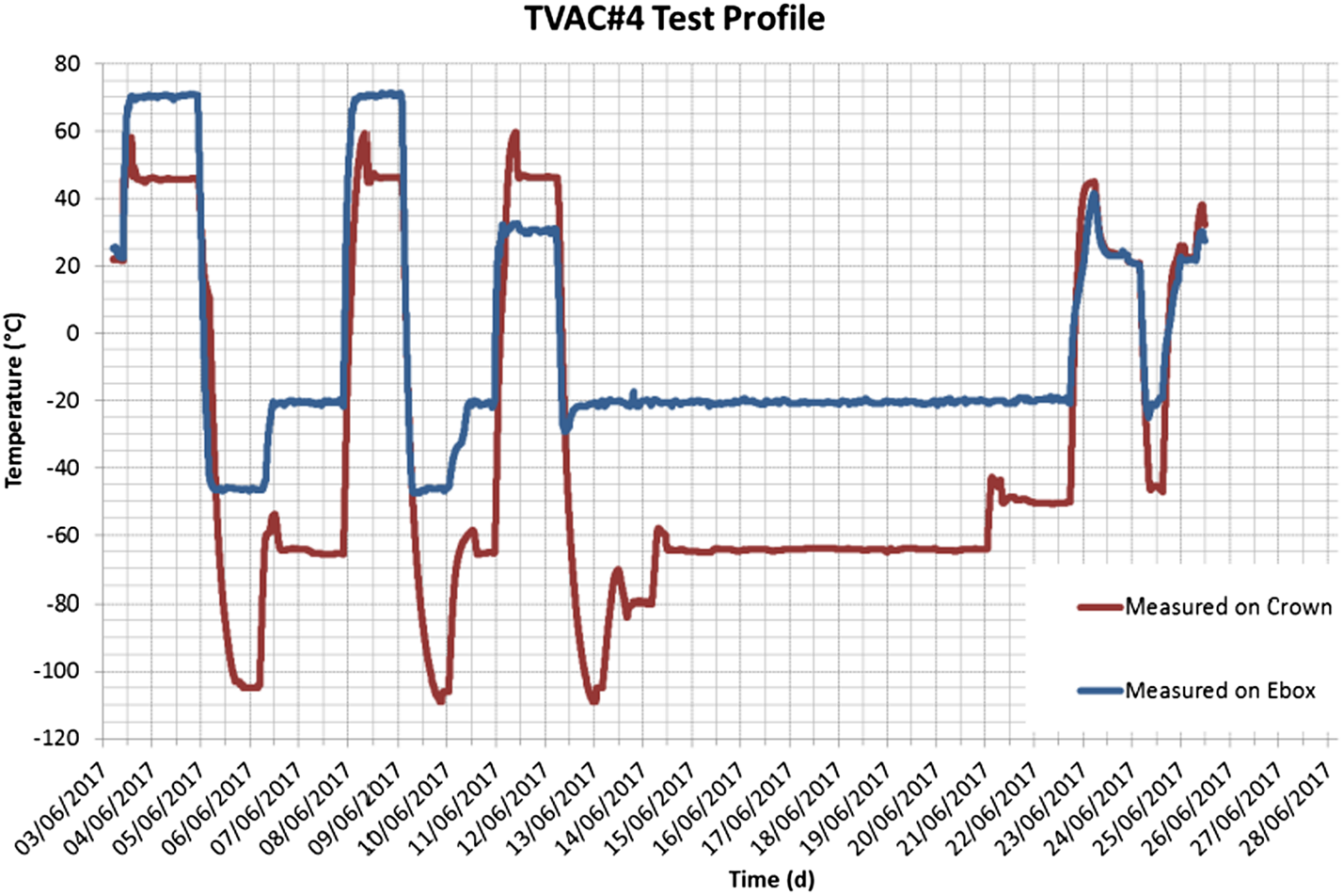
TVAC#4 profile as realized

Time constants were verified during the test using a first filter order model (see Fig. 109). The time constant VBB-sphere was estimated at 4 hr.

The Flight Model was successfully qualified within qualification temperature range. The benefit of the sphere time constant was used to reach qualification temperature on the sphere queusot for the first time after the sphere pinch out. It was achieved thanks to touch-and-dwells where the queusot temperature reached qualification levels while the VBBs inside the sphere had to remain within reduced temperature range. The test had to be driven carefully to not exceed a temperature difference higher than 40°C between crown and VBB during transients.

##### Landed TVAC

The landed TVAC (thermal balance test for SEIS) was used to correlate the thermal model and to measure the time constants of SEIS.

The estimation of the First order filter between sphere and VBB and between WTS and VBB was made using data from test with the formalism described in Sect. 6.6.1. But we also used the detailed thermal model correlated after landed TVAC test, for which the time constant is the time to have the sphere temperature at 63% of an external step, taken as $+10^{\circ}\mbox{C}$ during these tests. Results are shown in Table 19.

**Table 19:** Measurements of the Time constants during Landed TVAC tests

Time constant result for test	Requirement	1st order filter	Detailed thermal model
Sphere-VBB	2 hr	4 to 4.5 hr	5 hr
WTS-sphere	5.5 hr	5 to 6 hr	2.5 hr (in N_2_ atm.)
			4.6 hr (in CO_2_ atm.)

**Fig. 110:**
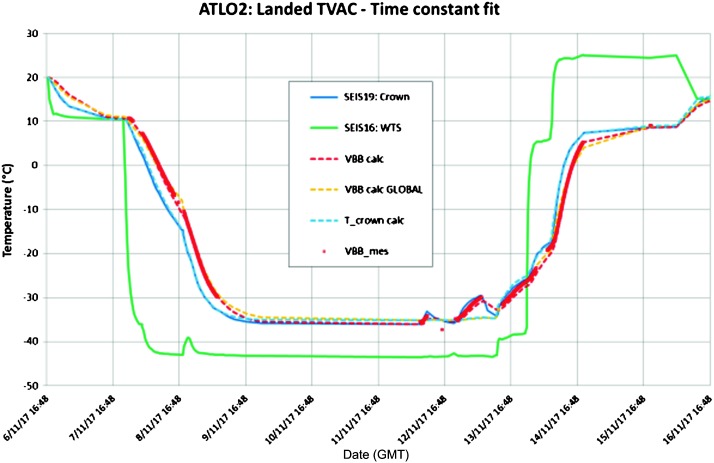
Comparison data vs. temperature calculated with 1st order filter

The Fig. 110 shows the good correlation between temperature calculated with first order filters and measurements, where:

- *VBB calc* is computed using a first order filter between VBB and crown temperature,
- *VBB calc GLOBAL* is computed using a first order filter between VBB and WTS temperature,
- *T crown calc* is computed using a first order filter between crown and a pondered temperature of RWEB and SEIS plate (70% SEIS plate—30% RWEB).

However, the WTS-sphere time constant is unexpectedly lower when computed with detailed correlated model on a 10°C step: 2.5 hours instead of 5 hours. This is because the 1st order filter is not representative of the heat exchanges between WTS and the sphere. In this method, the sphere temperature is a function of only one temperature (taken as the mean of the WTS and plate temperature) and tau. In reality, sphere temperature is driven by exchanges with the plate, the RWEB and harness and the RWEB itself depends of air exchanges. The detailed thermal model is representative of thermal heat exchanges.

The radiative exchanges (not linear) are not considered in the 1st order filter whereas they are significant and the detailed thermal model is representative. However, a 10°C step is not a realistic case (and is conservative) but this is the case defined to verify the compliance with the specified time constant. The detailed thermal model correlation appeared to be slightly optimistic on sphere-VBB time constant and pessimistic on WTS-sphere thermal constant.

Finally, the WTS-sphere time constant obtained with the detailed thermal model is more representative and is the one to consider for comparison with requirement. The values above are obtained in test condition: with N_2_ atmosphere instead of CO_2_ and in Earth gravity. Thermal exchanges are lower in Mars conditions and time constants on Mars are expected to be higher. This is consistent with the thermal flight prediction at EOL: 4.6 hr between WTS and sphere.

**Fig. 111:**
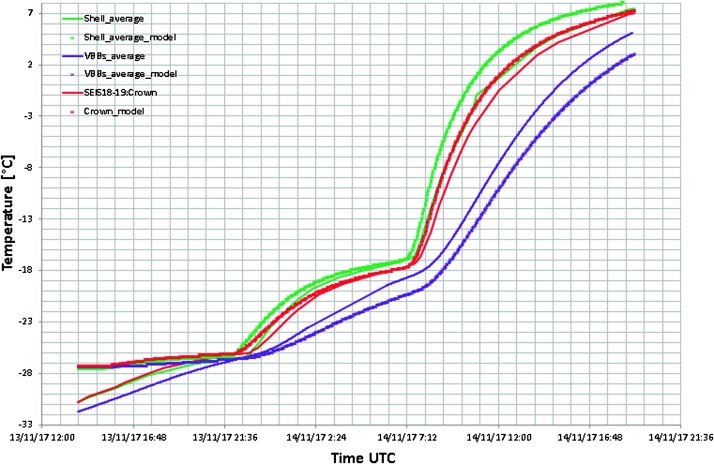
Comparison model-test data on the warm-up phase of Landed TVAC

The thermal model was successfully correlated within $\pm 5^{\circ}\mbox{C}$ based on Landed TVAC data even in transient phases. Figure 111 illustrates the good correlation between model (dashed line) and measurement (plain line) on sphere and VBB during warm-up phase of the test.

This model completed with the Flight sphere correlated model provides trustable flight prediction to confirm the compliance to thermal requirements. It will be used in operation to define the time of the day to switch on the instrument before WTS deployment.

#### SEIS Flight Thermal Prediction

Flight prediction has been achieved on 18 thermal cases that cover the whole mission on Mars:

- worst hot and cold conditions were analyzed in operating and non-operating mode,
- dedicated cases were analyzed for operation at deployment,
- additional cases were studied to refine the VBB coil temperature profile: it helped to perform thermal tests in more representative temperature range,
- sensitivity cases achieved to understand the sensitivity of SEIS to some parameters (wind speed, heaters, deck temperature).

**Fig. 112:**
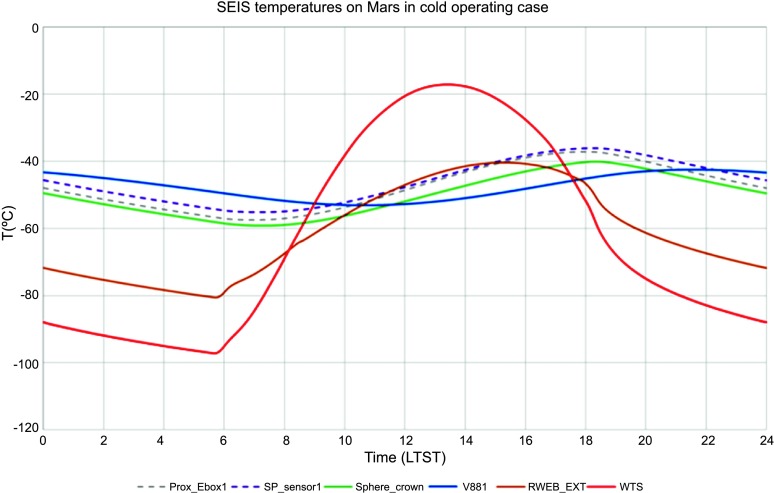
SEIS temperatures on Mars in cold operating case. Time is in Mars hours

**Fig. 113:**
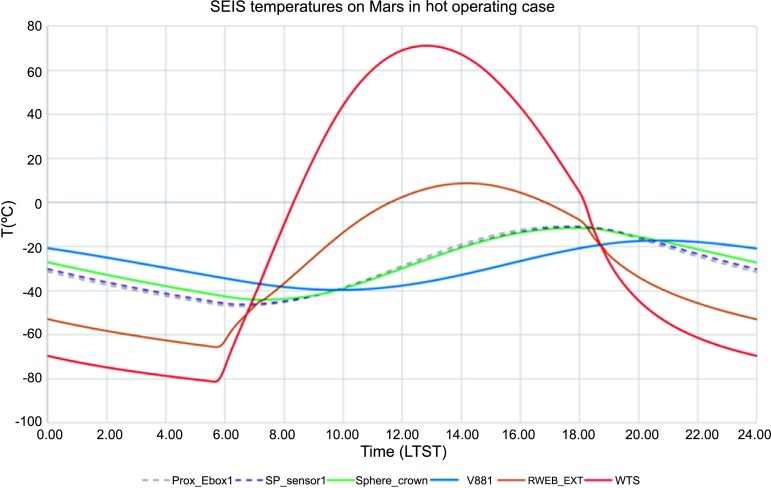
SEIS temperatures on Mars in hot operating case. Time is in Mars hours

Figure 112 and Fig. 113 show the temperature profile of main components for the cold and hot operating case respectively. 1.7 W of heating power is used in cold case. The good efficiency of the thermal protections is clearly visible: WTS temperature evolved from $-80^{\circ}\mbox{C}$ to $+70^{\circ}\mbox{C}$ while VBB remains between $-40^{\circ}\mbox{C}$ and $-20^{\circ}\mbox{C}$ in hot case. Note that uncertainties on dust conditions implied to study worst condition leading to higher solar absorptivity on the WTS and as a consequence to “high” temperature on WTS silicon oxide in hot case (Table 20).

**Table 20:** Impact of dust on solar absorptivity

Material/coating name	Infrared emissivity—$\varepsilon_{\mathrm{IR}}$	Solar absorptivity—$\alpha_{\mathrm{S}}$		
		Clean	Dusty horizontal surface	Dusty vertical surface
Stainless steel	0.40	0.50	0.58	0.52
Titanium TA6V	0.17	0.50	0.58	0.52
Titanium UT40	0.17	0.5	0.58	0.52
Aluminium AU4G1	0.2	0.5	0.58	0.52
Kapton	0.6	0.4	0.52	0.43
RWEB Kapton	0.84	0.56	0.62	0.57
Gold l (sphere)	0.05	NA	NA	NA
Gold 2	0.1	NA	NA	NA
Silicon oxide	0.2	0.25	0.46	0.34
Vapour Deposit Aluminium (VDA)	0.025	NA	NA	NA
Vapour Deposit Aluminium (VDA inside skirt)	0.06	NA	NA	NA

The main sources of daily variation of WTS temperature are the solar flux evolution and the air and ground temperature variations during the day. The thermal design implies very low leaks at each stage. The main leaks are through exchanges with air. The delay between sphere and VBB minimum temperature is clearly visible and is due to the sphere time constant. This delay impacts the operations in some particular cases, especially during deployment because PE and VBB must remain above their minimum allowable flight temperature (AFT) to operate and it does not happen in the same part of the day.

**Fig. 114:**
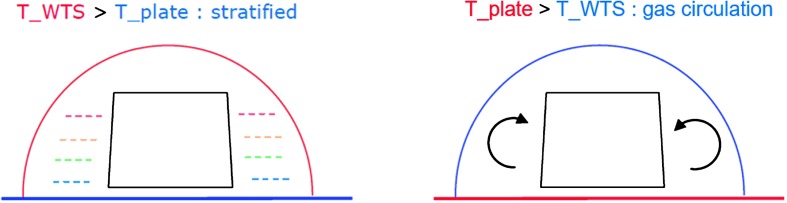
Air circulation impacting heat exchanges

The thermal time constant in the worst hot case end of life are 3.2 hours between sphere and VBB and 4.6 hours between WTS and sphere. The WTS-sphere time constant is lower than required but it is compensated by the larger sphere-VBB time constant. The unexpected lower efficiency of the WTS is essentially due to a phenomenon of air circulation increasing the heat exchanges between WTS and RWEB when the ground temperature is higher than WTS temperature and demonstrated that even for small atmospheric thickness, convection on Mars might appear (Fig. 114).

## Instrument Operations and Lander Onboard Management

### General Description of Operations

#### Overview

The SEIS operations will be performed by the SEIS/APSS Instrument Operations Team (IOT) at CNES, in Toulouse, France, with the support from both SEIS and APSS institutions. We therefore describe here not only the operations of SEIS, but also those related to APSS, as the latter is expected to provide critical data for assessing the impact of the Martian environment on the SEIS noise.

**Fig. 115:**
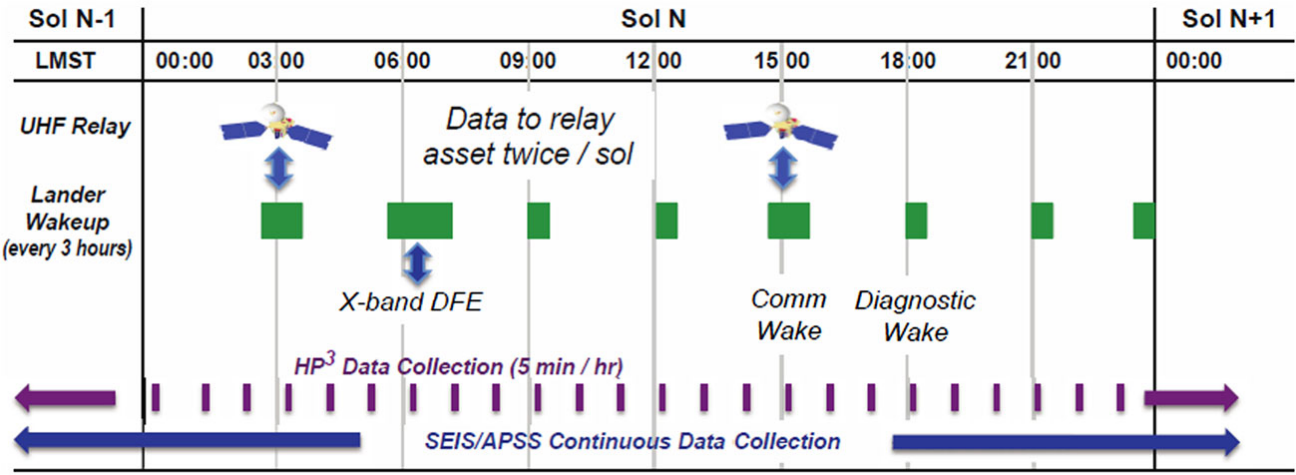
Overview of the SEIS Operations

SEIS operations are based on a weekly uplink cycle (Fig. 115) and two downlink opportunities per day. During the Science Monitoring phase, instrument operations teams will operate their instruments from their home institutions and the SEIS operation will be based on a regular week cycle with 4 working days, Monday to Thursday.

The lander communicates with the Lockheed Martin/JPL Ground Segment via UHF transmission to Mars Orbiters and then Earth transmission through NASA’s Deep Space Network (DSN).

During the science monitoring phase, there is insufficient energy to keep the lander powered continuously whereas both SEIS and APSS are working continuously and acquire high-frequency data. The lander wakes up every 3 h for about thirty to sixty minutes on average, to monitor and respond to faults, collect raw data from SEIS and APSS, store them in the lander mass memory, generate telemetry for the orbiters (NASA MRO and Odyssey), and receive command uplinks (via Direct from Earth or relay).

The continuous (i.e. low frequency) data will be downloaded entirely as described in Sect. 4.1.9, paragraph “Continuous Data” while the high rate will be selected and downloaded as event data. The key activity will be to manage the onboard data buffer for seismic events and ensure no data are erased onboard by newer data, since a cyclic buffer is used to store raw data. The event buffer, also called the raw data store, can store about 5 weeks of lossless compressed SEIS data. On average the mission can downlink $30~\mbox{Mbits}/\mbox{sol}$ of continuous data and $8~\mbox{Mbits}/\mbox{sol}$ of event data each sol, which is significantly less than the amount of data the instrument produces (about 650 Mbits per sol).

At the beginning of the planning process, SEIS team receives bandwidth and power allocations from Mission Planning team at JPL. Within this allocation, SEIS team determines the activities that can be performed with SEIS throughout the week. The SEIS science team analyzes the low resolution continuous data flow to detect seismic events, and then prepare a prioritized list of seismic events to be requested from the ground during the next uplink opportunity. Once the weekly activity plan has been defined, sequences of instrument commands, configuration files and possibly calibration waveforms are edited, validated and transmitted to JPL for bundling and radiation to the spacecraft.

#### Operational Roles

SEIS engineers are in charge of both the analysis of received data and the preparation of uplink products. They are operating from the SEIS Operation Center in Toulouse, France, called SISMOC (SeIS on Mars Operations Center). Their role consists in analyzing received telemetry to assess SEIS health and safety and prepare sequences of commands to be uplinked to SEIS.

#### Downlink Process

JPL is the only entity having direct interfaces to the spacecraft, via Lockheed Martin. SISMOC receives raw data from JPL.

Engineers track downlink data and check that received products correspond to the expected time spans. They report any possible missing products. They perform the SEIS health and safety assessment by monitoring key housekeeping parameters and metadata (activity durations, warning messages from the spacecraft, etc.) and check that no alarm has been triggered in the monitoring tools.

This process is fully part of the weekly activity plan preparation and requires a coordination within the SEIS operations team partners (CNES, IPGP, Imperial College, Oxford, ETHz, SEIS scientists from their home institution, etc.).

#### Uplink Process

**Fig. 116:**
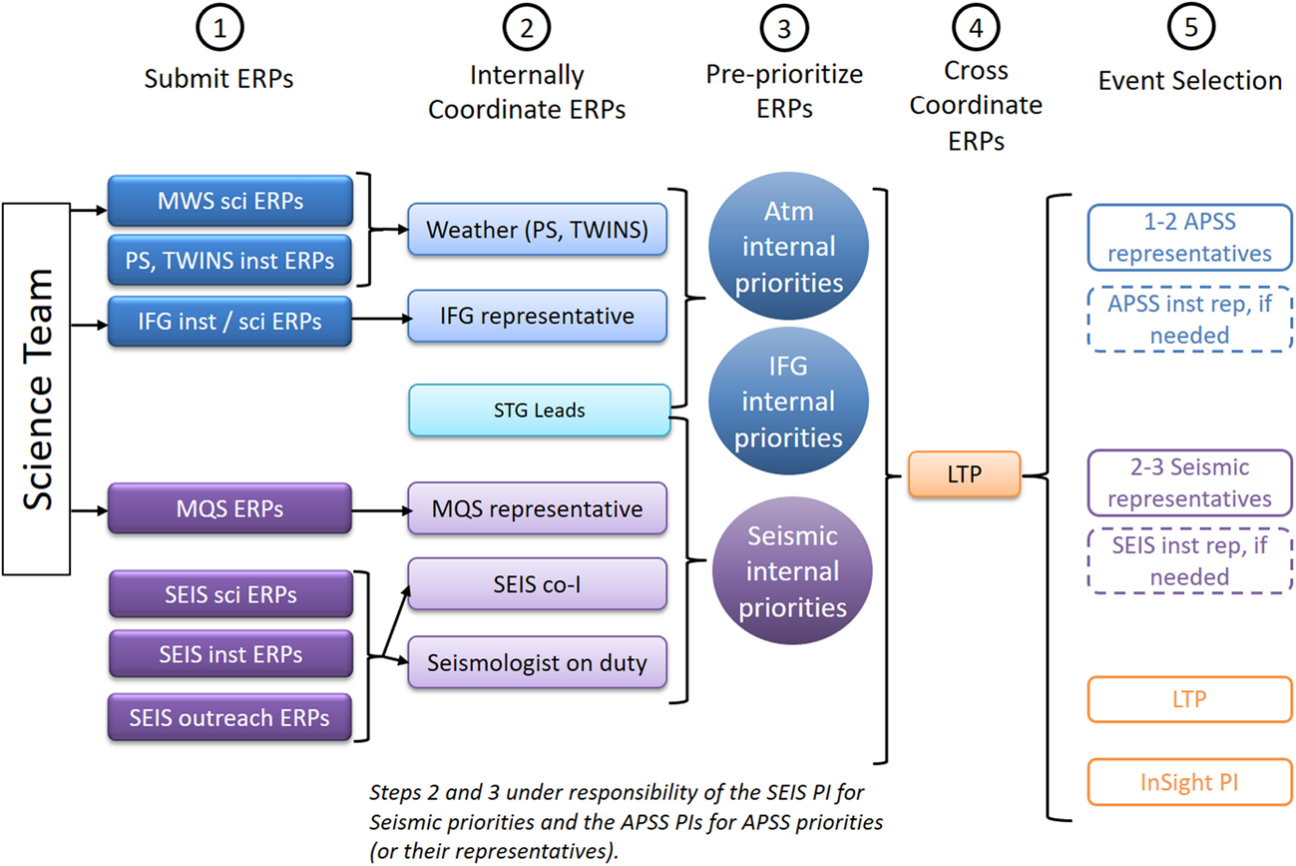
Event Request Selection Process

The main purpose of the uplink process is mostly to update on board data acquisition parameters: input acquisition frequency, gain change, decimation filters and output acquisition frequencies through configuration files. This process will also transmit the commands for the processing and downlink of the seismic events stored on board, based on the requests made by the science team (also called ERPs: Event Request Proposals). This is summarized in Fig. 116. The amount of event data transmitted back to earth depends mostly on the bandwidth, but also on the time allowed for data processing on the lander during the wake-up.

The SEIS operations team gathers all the sequences of commands and deliver them to JPL for bundling.

### SEIS Flight Software

#### Flight Software Functions and Design

SEIS on board Flight SoftWare (FSW) uses as inputs the SEIS channels provided by SEIS-EBOX and APSS channels provided by APSS-PAE. A total of 137 raw data channels are produced by the SEIS and APSS sensors. The outputs of SEIS-FSW are TeleMetry (TM) packets for transmission to Earth of SEIS and APSS data. The FSW is running on lander flight computer during lander wake-up periods. The FSW has the following functions:

- produce housekeeping TM packets,
- produce scientific data TM packets for continuous and event data flows,
- commanding and calibration functions of SEIS instrument.

Only scientific data processing will be described in this section.

#### Scientific Data Processing

The scientific channels produced by the SEIS and APSS sensors are recovered by SEIS-FSW during lander wake-up periods and processed in order to produce TM packets.

The FSW has a great flexibility because it allows the definition of processed channels that are the result of processing chains in which each stage is one of the algorithms described by Table 21. These algorithms are chained by taking the output channel of one stage as input of another stage to the processing chain.

**Table 21:** FSW algorithms available at each data processing stage described by inputs and processing parameters. For all processing, output is only one channel

Algorithm	Input channel	Parameters
FIR filtering	Any one SEIS or APSS channels	FIR coefficients, downsampling rate
Linear combination	Up to three SEIS or APSS channels	Inputs channel numbers, linear combination coefficients
Average over a time window	Any one SEIS or APSS channels	Window length
Standard deviation over a time window	Any one SEIS or APSS channels	Window length
Root mean square over a time window	Any one SEIS or APSS channels	Window length
Maximum over a time window	Any one SEIS or APSS channels	Window length
Time delay	Any one SEIS or APSS channels	Time delay to apply
Vector norm	Three SEIS or APSS channels	Inputs channel numbers, coefficients of vector components
No processing (but can downsample a channel)	One of any SEIS or APSS channels	downsampling rate

The processed channels are defined in a configuration file describing the processing chains starting with raw channels. All possible channels of a given sensor should be considered in order for the processing chain to be active when this channel is present. For example, all the processing chains of VBB1-VEL sensor should be defined for all the possible combinations of modes, gain and sampling rate of this sensor. For each processing stage, these configuration files include the numbers of input channel(s), output channels, the values of processing stage parameters and reference to FIR filter coefficient files as needed. Two types of configuration files are defined respectively for science continuous and event data flows.

#### Continuous Data Flow

The continuous data flow is defined through the data budget document and consists mainly of two types of channels:

- Downsampled data, corresponding to a single raw input channel downsampled to a lower rate by application of low pass anti-aliasing filters before downsampling operation.
- Processed data, corresponding to output channels involving more complex operations or even more input channels.

The downsampled data are produced by applying the “FIR filtering operation” which uses low pass anti-aliasing FIR filters defined for each downsampling ratio (2, 4 or 5) and decimates the channel with the corresponding ratio. These anti-aliasing FIR filters have been chosen identical to the ones of the terrestrial broad band seismological station CI.PFO. The same filters are also used inside SEIS-EBOX to perform the same operations. The highest sampling rate of the continuous downsampled channels is 2 samples per second (sps) during nominal operations.

**Fig. 117:**
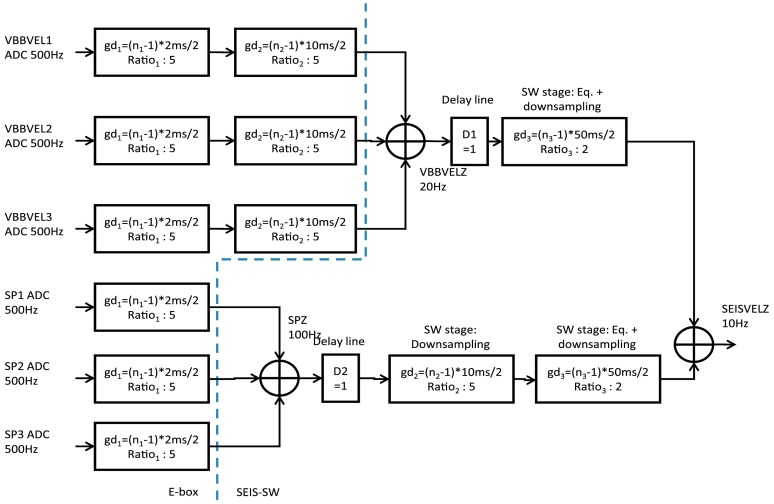
Example of SEISVELZ production chain by Ebox (on the left side of blue dashed line) and FSW (on the right side of blue dashed line) from SP and VBB channels. The final product (SEISVELZ at 10 sps) is provided in the continuous data flow

The processed data can be split into three main types of output channels:

- Energy Short Term Average (ESTA) channels, corresponding to estimate of signal energy of various sensors at frequencies above the Nyquist frequencies of continuous data.
- SEISVELZ channel, corresponding to a hybrid VBB/SP vertical velocity channel sampled at 10 sps (Fig. 117).
- Standard deviation and averaging operations applied on TWINS channels for wind retrieval.

ESTA channels that allow us to infer the signal content at high frequencies are computed for VBB-VEL, SP, Pressure and Magnetometer science channels. These energy estimates are designed to allow the detection of events involving only signals at high frequencies ($f>1~\mbox{Hz}$).

#### Event Data Flow

The INSIGHT mission stores all acquired data on board at full temporal resolution for approximately 30 Sols. This allows for the recovery of raw or high rate data stored in the lander’s mass memory through an event request. Such a request will select raw channels, or channels defined in an event configuration file, for specific time windows which are defined on Earth from the continuous data flow information.

The FSW event configuration file allows any of the operations in Table 21, except the vector norm or linear combination, before sending these to Earth. This capability allows us to tune the event data sampling rate to the available bandwidth. All the possible sampling rates for all the possible raw data channels should be defined in the event configuration file.

### Calibration on Mars

#### Coil Calibrations and VBB TCDM Tuning

The first SEIS Calibration on Mars will be made during the commissioning phase, which will start on sol 35 and last 60 sols. The calibration signals have all been designed in order to generate a signal reaching in the feedback loop about 50% of the saturation limit. The calibration results will be used for determination of the on-Mars transfer functions of the instrument, which will be distributed in the SEED dataless. The time line is described here:

- First 8 sols will be devoted to the TCDM optimization of the VBB.
- Next 10 sols will be devoted to calibration of the VBBs and SPs in their different modes and gains.
- The 28 following sols will be a passive cross-calibration, where both VBBs and SPs will operate continuously and will transmit back to Earth 2 sps continuous data, with selected time windows transmitted at 20 sps and 100 sps, with duration depending on the data budget.

VBB characterization & VBB+SP TF (transfer function) are the two first calibration phases and may be merged in order to perform VBBs calibration in every mode approximately at the same time (i.e. same temperature). The whole set of VBB Characterization and transfer function calibrations using SEIS-AC includes

- Open-Loop (LG/LG)
- ENG mode (LG/LG)
- ENG mode (HG/HG)
- SCI mode (HG/HG)

Each calibration lasts 17 minutes and recentering will occur in-between. During these 10 sols, this full set shall be performed three (to five) times, when VBBs temperatures reach their daily maximum, minimum and average.

After the commissioning, the calibration activities will be run every week for a short calibration sequence lasting a few seconds and designed to check the high frequency gain and every month for the “full” calibration sequence described above.

This set of characterizations and calibrations shall be performed seasonally in order to get calibration data for the VBBs with 20°C of interval.

#### Active Cross Calibration

##### Overview

In order to constrain better the internal structure of Mars, an active cross-calibration is designed between the VBBs and SPs in order to retrieve the gain of the VBBs relative to the SPs. This is detailed in Pou et al. (2018). As the frequency response of a VBB in POS SCI (position scientific) mode is flat for low frequency (Fig. 41), this cross-calibration is designed at low frequency and aims at determining the relative gain of all VBBs with respect to SPs, in order to determine the vertical gain of the VBB instrument with an accuracy of 0.5%. For operations purposes, the cross-calibration procedure can be done in one hour, by calibrating 2 VBBs in 30 min, then the 3rd one also in 30 min. Such procedure shall ideally be done for two or three different temperatures (max, min and mean temperature) in order to have a realistic model of the VBBs transfer function as close as possible to reality.

##### Principle

The principle of the cross-calibration procedure is to move the legs with the linear actuators in order to actively create a tilt and thus a signal seen by all VBBs and SPs. The most efficient signal was chosen to be a periodic signal with a period of 112 s, low enough to be close to the flat area of the VBB transfer function but high enough to be seen by the SPs (Fig. 53) with an amplitude of 0.002° in order to avoid saturation of any sensor.

The designed procedure is to cross-calibrate SPs with the VBB 1 and VBB 2 at the same time by moving first the levelling activator (LA) 1, then the LA 2 and LA 3 in a periodic way in order to create a split profile. Just after, SPs and the remaining VBB 3 shall be cross calibrated with a similar procedure but based on the movements of LA 1 and LA 3 first, then LA 1 and LA 2. The calibration is made by recording the outputs of all VBBs and all SPs for the whole duration of each procedure, together with the SEIS temperature. Using a periodic signal also allows us to do a frequency analysis to determine the gain of the VBBs while being more robust to noise sources and incertitude errors such as centering errors and ground dissipation of the leg movements. More details on the design and constrains on the tilt profile generated can be found in Pou et al. (2018) and only performances are summarized below.

##### Performances

Using these Split profiles for close to 30 min each time, the performance of the determination of the gain of the VBBs relative to the SPs is summarized in Table 22. The results are given on average and at the at the 90th percile over 1000 simulations, meaning for example that in all our simulations, in 90% of the cases, our knowledge of the VBB 1 gain was better than 0.22%. The results are worse for the VBB3 due to geometric reasons, since this VBB is the farthest from the SPs configurations.

**Table 22:** Accuracy of the active cross-calibration of the VBBs using the SPs (relative error over 1000 simulations)

Calibrated VBB	VBB 1	VBB 2	VBB 3
Accuracy on gain (average)	0.25%	0.30%	0.35%
Accuracy on gain (90th percile)	0.45%	0.45%	0.55%

After determining the gain of each VBB separately the vertical gain (Z-axis) of the VBB can then be cross-calibrated with the vertical SP1. With one hour of cross-calibration, it is possible to determine the relative differences between vertical VBB and SP1 with an accuracy better than 0.40% in 90% of the cases. This accuracy is better than the mean of the errors in Table 20 because positive errors and negative errors end up cancelling each other partially.

### SISMO and Events Management

The SISMOC (SeIS on Mars Operation Center), needed to support the SEIS operations, is specified, developed and performed by CNES.

#### SEIS Ground Segment Responsibilities

The functional capabilities allocated to the SEIS ground segment are:

- SEIS and APSS health and safety assessment.
- Programming of SEIS/APSS (including management of downlink bandwidth via configuration of the continuous processing).
- Various on board time correlations.
- FSW Configuration Management.
- On board seismic event buffers management.
- Detection/characterization of seismic events.
- Production, distribution and archiving of data and products.
- Detection of meteorite impacts (in collaboration with the Science Team of the Impact Working Group, see Daubar et al. 2018).
- Support Instrument deployment and commissioning phase.

In order to achieve these tasks, the SEIS ground segment is organized around 2 major components:

- The ***SISMOC***, which is installed in CNES-CST that mainly deals with the engineering operations and the science tactical processing,
- The ***Mars SEIS Data Service*** is in charge of producing high level end scientific products, to archive them and to distribute scientific products to the scientific community through the SEIS data portal. See Sects. 8.1 and 9.1.

#### SISMOC Main Functions

**Fig. 118:**
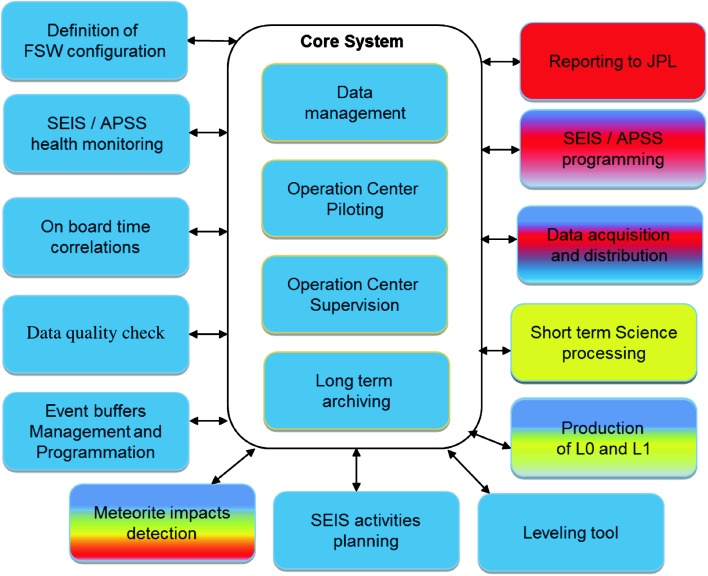
SISMOC functions with the roles of JPL (in red), CNES (in blue) and the Science services and team (in yellow)

On one hand the SISMOC offers a set of basic services such as data management, task scheduling and system supervision constituting the core system of the operation center and on the other hand, the SISMOC includes a set of mission specific services such as management of the event buffers or correlation of the various clocks. Figure 118 provides a schematic view of SISMOC functions. Some of these functionalities are partially or fully met either by tools that will be delivered by the JPL or the science team or by CNES multi-mission tools and CNES facilities.

#### SISMOC External Interfaces

The SISMOC interfaces with the InSight Ground Data Segment at *JPL*. SISMOC will receive from JPL:

- The ***SEIS FSW TM packets*** containing the raw CSSDS packets of SEIS and APSS,
- The ***Warning and Info Science*** ***EVR*** files containing the event records related to SEIS and APSS logged by InSight lander,
- The ***Ancillary data*** files containing the lander engineering data that are useful to state on SEIS and APSS health and safety,
- The ***Command history*** files containing a log of the command executed by the SEIS flight software,
- The ***Science activity planning*** files containing the predicted pass of satellite communication relays and associated estimated downlink volume for a certain period of time, foreseen lander and flight software wake-ups,
- The ***SCET files*** containing spacecraft event times that provide time correlation information between the spacecraft clock and UTC time,
- The ***TLM dictionary*** containing SEIS and APSS telemetry definition,
- The ***Deployment data*** are corresponding to images, digital terrain model, etc. allowing SEIS IOT team to support SEIS deployment on Mars surface,
- The ***ATLO data*** is a placeholder to handle ATLO specific data if any.

On the other hand, SISMOC will deliver to JPL:

- The sequences files containing SEIS and APSS sequences that constitute the weekly programming of SEIS and APSS instruments,
- The VML blocks files containing the definition of command blocks that will be stored on board InSight flight software lander and can be called or spawned by a sequence,
- The FSW configuration files are binary files that can be uploaded to the lander in order to configure SEIS flight software processing of continuous data,
- The reports files corresponding to the various reports that will document SEIS operations activities.

For data distribution purposes, SISMOC interfaces with the Mars SEIS data service and with CAB. It gets from MSDS the part of the SEED dataless which does not depend on the flight software configuration. It also gets from CAB the TWINS/APSS processed data product, providing wind and pressure in physical units. It then delivers to MSDS the SEIS and APSS ***level 0 data*** in miniSEED format and the SEIS and APSS ***level 1 data*** in SEED format. These data will also be transferred to the MQS for the purpose of quake detection.

For instrument monitoring purposes, SISMOC interfaces with the SEIS and APSS team taking part in the tactical operations, including health monitoring, calibration, etc., of the instruments. In addition to getting the SEIS and APSS data in miniSEED and SEED, monitoring data will be made available through a second, CNES hosted system (IMIS).

Finally, SISMOC will be responsible for preparing the event requests of both science and instrument communities, when the latter have been endorsed by instrument or science operations. SISMOC will therefore deliver to the SEIS data portal the ***event buffers content information*** (through the event buffer management tool) and will receive from both the science and instruments communities the SEIS and APSS ***event request proposal*** (through the event buffer management tool).

#### Focus on a SISMOC Function: Event Buffer Management

As described in Sect. 4.1.9, paragraph “Data budget”, SEIS data will be transmitted either in the form of continuous data-flow or event data-flow. The latter are selected high rate seismic data produced only for specific windows of time (the “seismic events”) and the operational implementation of the event transmission required the development of tools described below.

Into SISMOC, the Event Buffer Management (EBM) tool shall manage SEIS/APSS buffers on-board the lander, i.e. keep track of the content of each buffer at any time and support the development of new requests of full-rate data.

The EBM tool is accessible from everywhere for authorized users and offers the following functions:

- Visualize the event buffer contents,
- Develop a programming of event windows,
- Generate event sequences,
- Model and adjust the buffers content,
- Select the Event Request Proposal (ERP) from science teams.

After analyzing the continuous dataflow, the science teams will send their ERPs to SISMOC. Information about ERPs are available online for authorized users. A pre-ranking of ERPs by each science group should be possible before the weekly event selection meeting.

According to the on-board available volume and CPU, ERPs are selected weekly: the corresponding data are programmed to be stored into the on-board event buffers and then to be downloaded. All information about event request status, buffers status and on-board event plans are available online through the ERP/EBM tools.

## SEIS Services

### Mars SEIS Data Service

The Mars SEIS Data Service (MSDS) is led by IPGP. This is the operational service responsible for collecting from SISMOC, archiving and distributing data for the SEIS experiment to the scientific community. After project completion, the IPGP Data Center will also maintain an archive for long-term preservation.

The MSDS is also responsible for synchronizing the SEIS data to the IRIS Data Management Center (IRIS-DMC) and to a US CO-I responsible for archiving the data in NASA’s Planetary Data System (PDS). Then, the data will be delivered and is freely available through the IPGP Data Center, but also through IRIS and PDS. Data format on both MSDS and IRIS will be those compatible with IFDSN (International Federation of Digital Seismograph Networks), while the format on PDS will be PDS4 or PDS compatible.

The MSDS is responsible for the management of the raw, calibrated data and reduced products generated by the SEIS instrument (VBB and SP), the SEIS Flight Software and the APSS instrument (magnetometer, pressure, wind and temperature) in the same format as SEIS. In addition, the MSDS collects and archives housekeeping data.

**Fig. 119:**
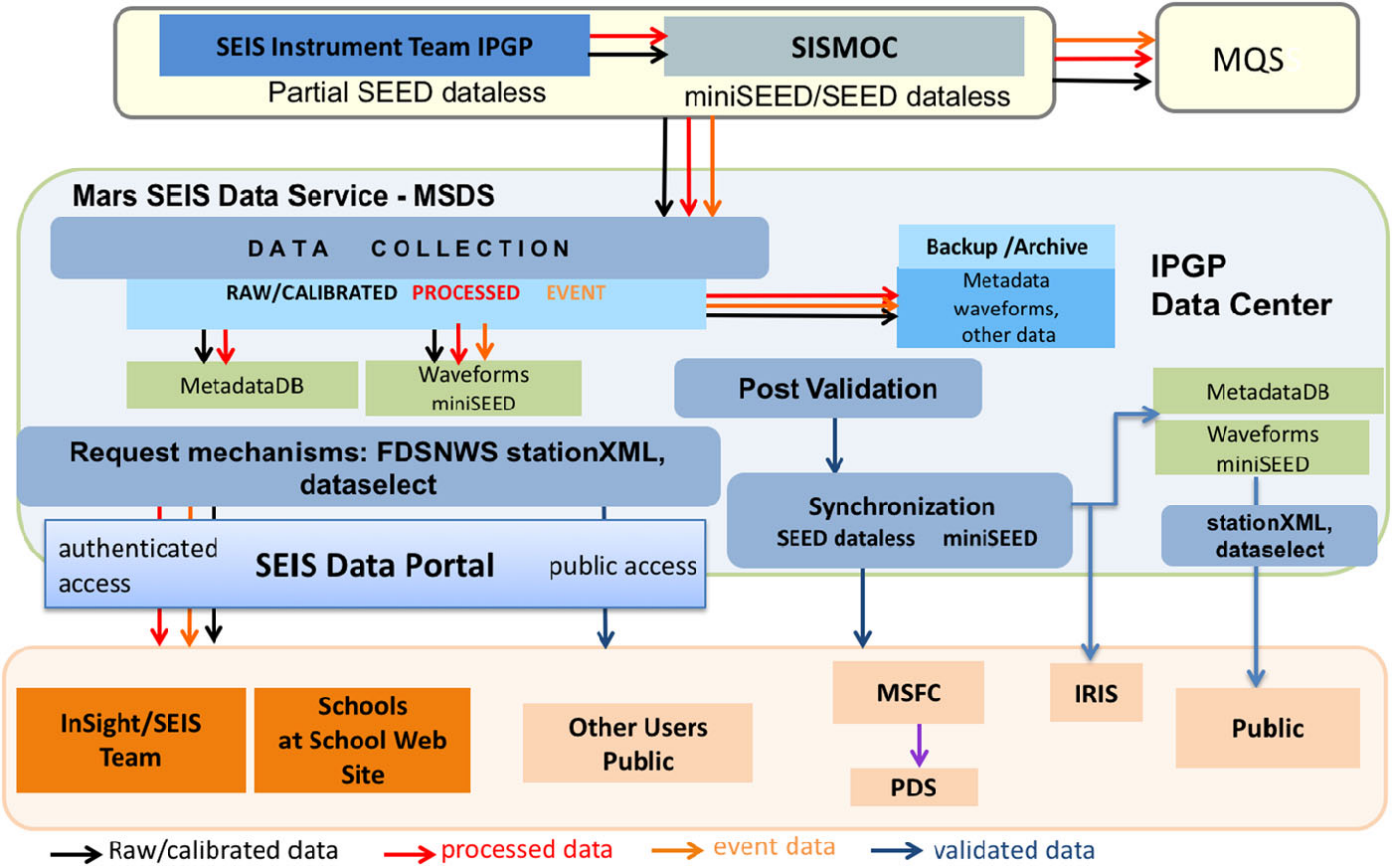
This summarizes the SEIS data flow from SISMOC to the scientific community

The data flow is described in Fig. 119 Data will be collected, archived and distributed in the standard exchange format defined by the International Federation of Digital Seismograph Networks (FDSN, http://fdsn.org): dataless SEED or stationXML for the metadata and miniSEED for the waveforms.

Data will be automatically collected by the MSDS from SISMOC after being converted to miniSEED format and following the notification of the new or updated available data. Then, MSDS checks integrity and format of the data; ingests new or updated data making them automatically available.

Users will access all available data provided by the MSDS through the standard FDSN Web Services ***station*** in stationXML format and ***dataselect*** in miniSEED format (https://www.fdsn.org/webservices/), as well as the dataless SEED through http protocol.

The SEIS Science Team will be able to access all the data by the authenticated Web Service during proprietary periods, while these data will be open after.

Web services available in MSDS will be used by the SEIS Data Portal to provide an interactive and guided access to the user, both through the public and the restricted access SEIS Portal sections.

Users will be notified of new or updated data by a RSS notification available through the SEIS Data Portal when registered on the RSS feed.

### Marsquake Service

The MarsQuake Service (MQS) is the operational service for the SEIS instrument responsible for delivering catalogues of seismicity, one of the primary science products of the InSight mission. In this role, throughout the course of the mission, the MQS is responsible for prompt and routine detection and characterization of seismic events according to currently preferred sets of Martian interior models; assembling events into catalogues; disseminating event and catalogue information to the Mars Structural Service (MSS), scientists and public via the InSight portal; and reviewing catalogues following model updates. At the end of the mission, the MQS will deliver a final catalogue version to the Planetary Data System (PDS).

A detailed discussion on the methods used to detect, locate and characterize seismic events is described in Khan et al. (2016) and Böse et al. (2017), following, for large earthquakes, the multiple Rayleigh detection method described by Panning et al. (2015). A probabilistic approach will combine independent estimates for distance, origin time and back-azimuth. These key parameters will be determined using any and all of the surface and body waves that can be identified. 3D crustal structure will be accounted for. Magnitudes will be determined following formulations typically used on Earth, with Mars and InSight-specific modifications, using amplitudes of various phases measured in specific frequency bands, such as Richter magnitude, body wave magnitude and surface wave magnitude (Böse et al. 2018). Efforts will be made to use depth phases and matching synthetics in order to infer depth. Discrimination between tectonic and impact events will be made where possible. The methods and software infrastructure have been exercised with success using Martian synthetics during the MQS Blind Test (Clinton et al. 2017).

Absolute locations, especially those for events with low signal-to-noise, will be refined within the context of an overall seismicity catalogue—once a significant number of events have been identified with good quality locations, in the absence of clear arrivals, the distance of weaker events can be inferred by matching signals from well-located events. Cross-correlation tools or Hide Markov Model methods will be used to further augment the catalogue with otherwise unlocatable or even undetected events.

For impacts, the preliminary locations of MQS will be updated by those provided by the Impact WG, JPL and CNES teams using Martian satellites, when new impact craters will be located on remote sensing data. These ground truth locations for impact events will provide strong constraints on the interior models. In these cases and when seismic events suspected to have an impact origin with a location known without large uncertainty, procurement of local high-resolution satellite images will be prioritized. Conversely, if impacts are identified by routine satellite observation, the seismicity catalogue can be reviewed to try to identify a corresponding seismic event. See details for impact location and science in Daubar et al. (2018).

A final key role of the MQS is to prepare ERPs in order to collect more complete high frequency seismograms for observed events. The MQS will also refine locations based on the higher sample rate data. The MQS is described in detail in Clinton et al. (2018).

### Mars Structure Service

The Mars Structure Service (MSS) is the operational service for the SEIS instrument responsible for delivering interior seismic structure models. This is one of the primary science products of the InSight mission and the MSS is responsible for producing and updating such models throughout the course of the mission and delivering a final version to the Planetary Data System (PDS) at the end of the mission.

**Fig. 120:**
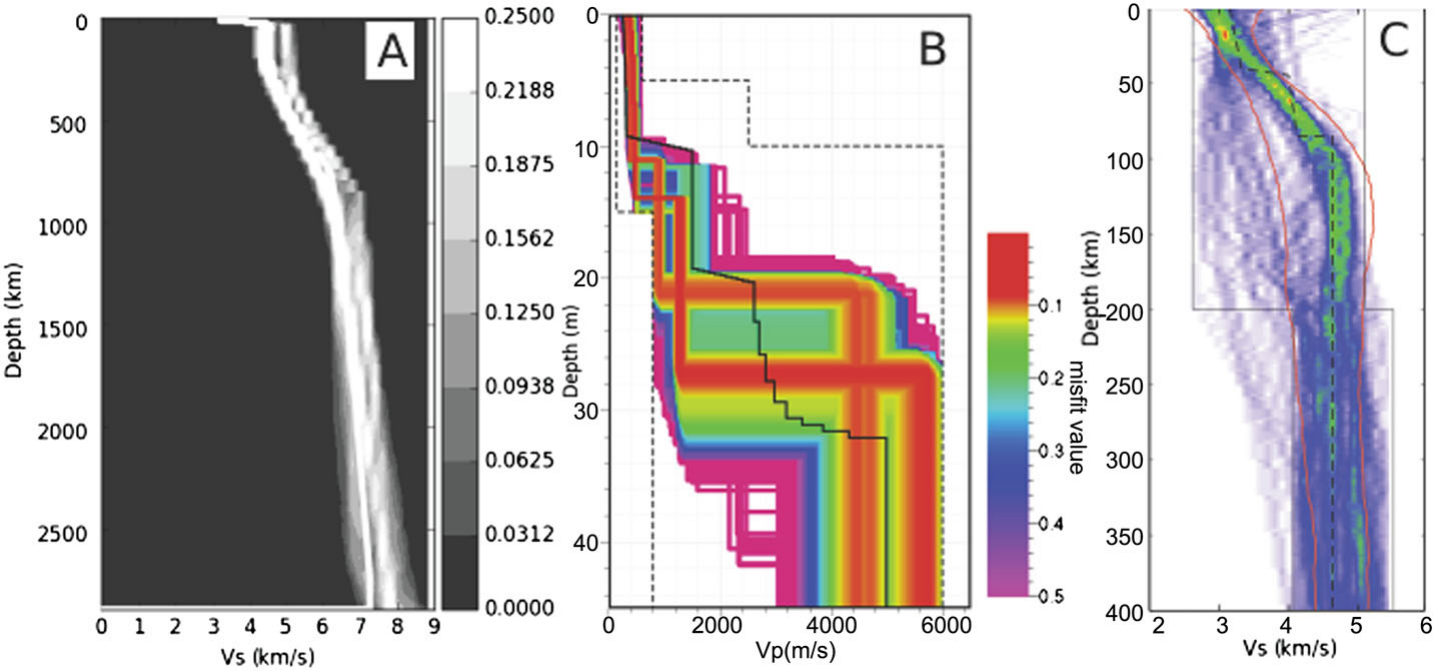
Examples of preliminary demonstrations of the anticipated products of the MSS from Panning et al. (2017). (**A**) Example demonstration of the probability density function output for the Bayesian inversion of a small number of P, S and Rayleigh wave group arrival times for resolution of Earth mantle velocity structure. (**B**) A range of models of shallow crustal structure with color of plotting representing data misfit inverted to match synthetic Mars observations of the frequency dependent ratio of vertical component to horizontal component amplitude. (**C**) Bayesian inversion of mantle structure from noisy synthetic long period normal mode spectra. Green colors represent higher probability models while blue color is lower probability

A detailed discussion of the range of modeling products planned to be produced by the MSS is described by Panning et al. (2017). This is anticipated to be a range of models on different scales ranging from the shallow subsurface to global-scale models using many different seismic observations (Fig. 120). The general approach to most of the modeling planned relies on Bayesian methods. Such approaches are increasingly common in geophysical applications and rely on the creation of large numbers of models with a distribution proportional to the probability density function of the models given the constraining data. Using such an approach allows for determination of the most likely range of models to fit the observed data without the need for the assumptions often required to explicitly determine the sensitivity of the data misfit to model perturbations. This creates a range of models that can satisfy the data within uncertainty, allowing for a clear understanding of model uncertainty (Fig. 120).

Operationally, the MSS is responsible for delivering a range of a priori models of structure (e.g. Sect. 2.2) which is used broadly by the science team for the mission, as well as allowing for probabilistic location of the initially recorded events by the MQS by taking in account differences in body wave and surface wave arrival time predictions for a reasonable range of models (e.g. Böse et al. 2017). As data becomes available, this set of models will be refined by Bayesian inversion of the recovered data and the revised models will be distributed to the community and used by the MQS to reduce location uncertainties.

## Data Distribution and Archiving

### SEIS Portal

The SEIS Portal (http://www.seis-insight.eu) is a hub leading to three distinct websites, each tailored toward a specific population of users/visitors: the general public, the scientist and finally students and teachers.

The public website offers a complete, understandable overview of the SEIS experiment and of the InSight mission, through four sections and 200 web pages.

The first section explains some basic principles in seismology and presents previous planetary seismology experiments. The second section is dedicated to SEIS and presents not only the instrument itself, but also the legacy of the development behind it, as well as lot of information related to tests. The third section focuses on the InSight mission itself, including the lander. Finally, the last section deals with Martian science: after a short presentation of the internal structure of rocky planets, several articles introduce the reader to the many scientific goals of the InSight mission. New contents will be regularly added through the mission. The primary target audience of the general public website is teenagers and adults. Suitable content for younger children will be available at the ETHZ website (http://marsatschool.ethz.ch/en/index.html) while those targeted for teenagers and students will be located at the GeoAzur website (https://insight.oca.eu/).

In order to provide the necessary graphic support for explaining the instrument characteristics and science rational, a set of didactic colorful original illustrations and animations has been created in the science section of the public website, including artist’s concepts. Several more sophisticated graphical products have also been developed, such as a fully textured cutaway of the SEIS instrument at the surface of Mars, a real-time interactive 3-D model of the VBB pendulum, 360° cylindric view of hardware or 3-D models of meteorites, etc.

The scientific website of the SEIS Portal is dedicated to more professional seismologists and will provide access to different sets of data and specific documentation. Data distribution is described in Sect. 9.2. The third website encompassed in the SEIS Portal is focused on education and outreach described in Sect. 9.4. It will give access to diverse education and public outreach initiatives and to multiple sets of educational resources. Two additional areas on the SEIS Portal are dedicated to news and media. With a few exceptions, content is presented both in English and French.

### SEIS Data Distribution

**Fig. 121:**
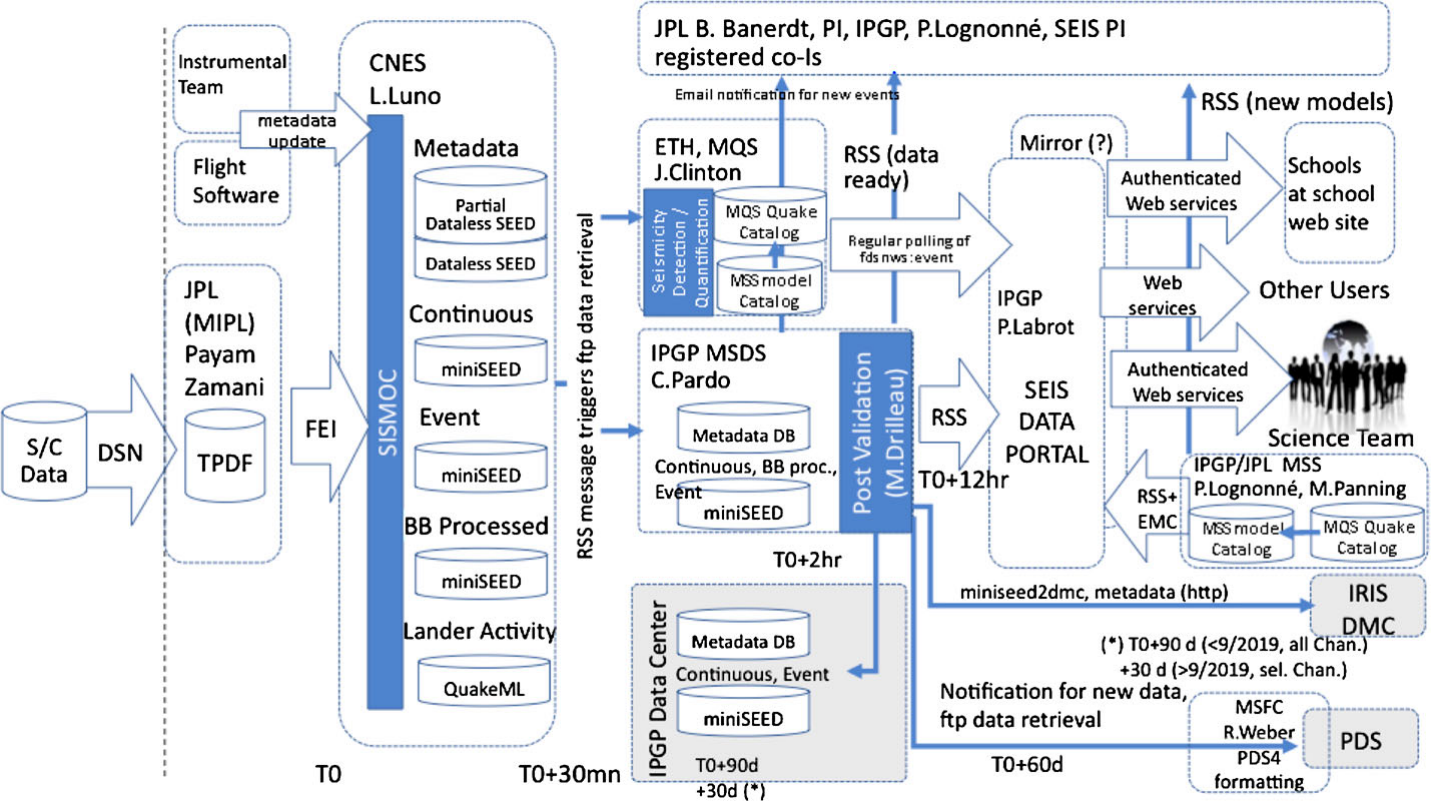
Data flow with key contact persons

The SEIS data flow is described in Fig. 121. Raw spacecraft data are downlinked through the Deep Space Network to the Multi-mission Instrument Processing Laboratory (MIPL) at JPL. These are transferred to SISMOC via the File Exchange Tool (FEI). Data are then archived in SEED format ($\mathbf{S}$tandard for the $\mathbf{E}$xchange of $\mathbf{E}$arthquake $\mathbf{D}$ata). Reader not used to SEED can found additional informations on this data format in the SEED manual (FDSN 2012) and in Appendix B.

IPGP will provide SISMOC with the static dataless SEED associated with the Instrument Transfer function and other non-flight software (F/SW) tunable parameters. SISMOC will then:

- Complete the SEED dataless with dynamic information from F/SW tunable parameters.
- Generate raw and calibrated data from the raw spacecraft data and static SEED dataless.
- Generate processed data from the “Black Box.”
- Distribute all above data in miniSEED/dataless SEED to MSDS (Mars SEIS Data Service) and MQS (Mars Quake Service).

Relevant APSS data (such as Magnetic field, Temperature, Pressure, Wind Direction and speed) will be packaged with the SEIS data. The IPGP SEIS Team (with support from the SP and APSS teams) will certify the integrity of the SEIS/APSS SEED data to MSDS.

MSDS will then archive and make the data available through FDSN Web Services (*fdsnws-station*, *fdsnws-dataselect*) in FDSN StationXML and miniSEED formats. Metadata in dataless SEED format will be also available. MSDS will archive the data as a mirror node of IRIS.

SEIS, InSight science, outreach and education teams will be able to access all scientific SEIS and APSS data both through authenticated FDSN Web services and through the SEIS Portal (SEIS Team members Intranet). MSDS is responsible for final delivery of SEIS data in SEED (dataless, miniSEED) format to the IRIS and the PDS archive generation team.

The data formatted according to the SEED format will be distributed under the reserved **FDSN Temporary Network Code XB** (period 2016–2022). Two station codes are planned: ELYSE for the scientific data and ELYHK for the housekeeping data. In addition, a Digital Object Identifier (DOI) is planned for these data.

IPGP will also deliver the Mars Reference Internal Structure catalogues through the MSS (Panning et al. 2017 and Sect. 8.3) while ETHZ will be in charge of delivering the Mars Quake catalogue through MQS (Clinton et al. 2018, this issue, and Sect. 8.2). See Sects. 8.2 and 8.3 for more details. The PDS will distribute and maintain all InSight archives for the NASA planetary science community and the general public. SEIS data will be archived with the Geosciences Node. Release of SEIS data in open access and in SEED format at both IPGP and IRIS DMC will be synchronized with the release of these data at PDS.

SISMOC will also make other relevant data available, in particular:

- The time correlation coefficients.
- The FSW configuration files.
- The EBOX/PAE configuration at a given date.
- The ERP approved/rejected list containing the results of the weekly coordination meeting on event selection.
- Notifications for a new file presence (RSS).
- Coherence files produced by the coherency calculation science processing (black box), and
- Deglitch log files produce by deglitch science processing.

### SEIS Services Higher Level Products

The key higher-level products that will be delivered from the SEIS experiment will be seismicity catalogues and models of the Martian structure.

Seismicity catalogues will be created and curated by the Mars Quake Service team. The methods used to detect and characterize seismicity are described in detail in Clinton et al. (2018) and summarized in Sect. 8.2. The catalogue is expected to include both tectonic and impact events but will not include lander or weather related activity. The format will be quakeML1.2 (https://quake.ethz.ch/quakeml/), with InSight-specific extensions to reflect the single-station methods, the probabilistic location formulation and the Mars-specific event types (marsquakes, meteor impacts). Typically, events will be identified within hours of data being received on Earth. Event information will be distributed immediately to the science team via the InSight portal via standard webservices fdsnws/event (https://service.iris.edu/fdsnws/event/1/), with extended Mars-specific options. Updated marsquake catalogues will be made available once structure models are updated.

The primary product from the Mars Structure Service will be models of the Martian interior. As described in Panning et al. (2017) and Sect. 8.3, models will be developed in a Bayesian probabilistic fashion and so the final product will be a suite of models of interior structure. These models will include seismic velocities and depths of major structural transitions, such as the core-mantle boundary and mean crustal thickness. The models will be delivered in a flexible “deck” format based on that originally utilized by the widely-used seismic free oscillations code MINOS (e.g. Woodhouse 1988), but modified to be more flexible and include more possible structural parameters to be compatible with the model format used by the AxiSEM numerical wave propagation code (Nissen-Meyer et al. 2014). 3D crustal models based on gravity and topography (e.g. Neumann et al. 2004) constrain only crustal thickness variation while not directly constraining mean crustal thickness. As seismic data constrains the crustal thickness at the landing site, updated 3D crustal models will also be produced, released in both latitude and longitude sampled files as well as spherical harmonic expansions useful for gravity comparisons. These models will be delivered and updated regularly during the course of the mission for use by the MQS probabilistic location algorithm and periodically made available via the InSight data portal.

Martian interior models and seismicity catalogues will be periodically distributed to the public through the InSight portal using the same services described above, following the ending of the embargo periods. At the conclusion of the experiment, the full range of models and seismicity catalogues will be provided to the final mission Planetary Data System (PDS) product.

### SEIS Education Plan

#### Overview

The SEIS INSIGHT education plan has been designed in order to develop a specific scientific programme for secondary schools, high schools and general public, allowing a generation of school kids, teens and students to follow the mission at the same time as the InSight project scientists.

The main objectives are:

- To provide to the school network the seismic activity of another terrestrial planet.
- To initiate comparative planetology activities in school based on space mission data.
- To test through fun hands-on experiments the key processing methodologies used by InSight.
- To organize workshops for teachers and to explore some innovative activities in geophysics teaching.

#### Resource Distribution

The resources will be organized by topics to facilitate the teachers’ school activities.

Topic ‘DATA’ will be one of the most highlighted topics. Hundreds of secondary schools and high schools mostly in US, UK, France and Switzerland, will receive SEIS (and weather) data from Mars daily following the public data release. This wide distribution of data is made possible by the worldwide partnership already existing between seismological educational networks (OCA-France, IRIS-US, ETHZ-Swiss, GS-England). See Courboulex et al. (2012) and for activities in France (Berenguer and Virieux 2008). Students will have access to data selected for educational use, then teachers will be able to propose case studies to investigate the planet Mars.

Topic ‘TELLURIC’ concerns a lot of hands-on about planetology, seismology experiments, meteorites impacts and/or physical states of water, pressure, temperature, etc. With the help of data from the camera HiRISE, students will also be able to print Digital Terrain Models files with 3D printers and learn more about Mars topography.

Topic ‘JOURNEY’ will provide the resources to explain the launch, Earth-Mars transfer and landing. This will be made with hands-on tools and dedicated software, providing Mars’ orbit characteristics, distances and planet positions and allowing the selection of the launch time window or landing site.

Topic ‘SENSOR’ means to better to understand data recorded on Mars. A lot of experiments using simple or more sophisticated sensors have been developed for the classroom. Technical high school students can build easily by themselves seismic, pressure, temperature and wind sensors and have the opportunity to draw and build replicas of the lander and sensors. Elysium, a replica made by students in Toulouse and presented in ‘Salon du Bourget’ in 2015, is one example. Robotics, electronics and computer courses will take advantage of this topic.

Topic ‘SIGNAL’ is necessary to understand communication techniques between Earth and Mars during the mission. It describes experiments for students about differences between analog and digital signals, on the sampling properties of signals and on the sun insulation on Mars as compared to Earth.

#### Share with the Educational Community

All of these resources and data must be provided and shared through the educational community. Data and activities, ranked by topic and by level of school age, will be displayed on the web site for Education. Teachers and the general public will be able to access these web pages through the SEIS portal. The teachers will find data (data selected and case studies), hands-on activities (described step by step), sensors and replica plans (helpful to build models) and dedicated software (to read data, to simulate phenomena) and it will be easy to download all these educational packages.

The success of this specific program for schools is dependent on good teacher training. It is necessary not to forget tutorials and documentation so that the teachers can access the resources easily and quickly. Some workshops with teachers and scientists involved in the mission will help to provide training for this specific program.

With this education program, we will be able to bring the InSight mission and the SEIS experiment into the classroom and give to the pupils and their teachers, the opportunity to do science, with a multidisciplinary approach and connected with the scientists.

## Conclusion

Forty-two years after the landing of the two Viking seismometers on Mars and 41 years after the end of the Lunar ALSEP seismic network on the Moon, the SEIS instrument on InSight will start a new chapter in terrestrial planetary seismology and will search for the first definitive quake detected on a terrestrial planet other than Earth.

More than 60% of the SEIS mass has been allocated to its surface wind and thermal protection and we can therefore expect a surface installation optimum with respect to the constraints of a Mars robotic mission. The deployment will however be made on a subsurface with low rigidity material (Morgan et al. 2018), due to landing safety contingencies and the need for such a subsurface for the successful deployment of HP3, another InSight payload instrument aiming to measure the heat flow of Mars. Pressure data will therefore be recorded continuously in order to minimize the pressure related ground deformation noise.

Thanks to InSight’s robotic arm, SEIS will therefore benefit from possibly the best installation scenario to be made by a static lander and will be able to detect quakes three orders of magnitude smaller than Viking. This results in a predicted detection threshold of moment magnitude $M_{w} \sim 4$ for a global detection and $M_{w} \sim 3$ for up to 40° epicentral distance. In addition, SEIS measurements will be continuously supported by the APSS suite of pressure, wind and magnetic sensors, which will not only allow systematic noise decorrelation and event validation but will also make joint event monitoring possible, from regional impacts with joint seismic and infrasound signals to local dust devils with seismic, pressure and magnetic signals.

SEIS is in essence a true discovery experiment on a Discovery mission, in the sense that it will possibly perform a sequence of discoveries comparable to those made on Earth 120–100 years ago: first marsquake detection, first solid tide observation on Mars’ surface, first seismic impact detection and first constraints on the crust, upper mantle and core size. In addition, SEIS will also explore a planet where micro-seismic noise is likely only generated by the atmosphere, in contrast to the Earth were micro-seismic noise is dominated by oceanic waves and anthropogenic activity.

SEIS data will be distributed in SEED format, following the schedule of NASA’s Planetary Data System. In addition, these data will be made available at the IRIS DMC and at IPGP Data Center. Data will also be distributed to a wide international network of schools and colleges, through the educational programs in the USA and in several European countries.

We can therefore hope that the installation of the, possibly long-duration, InSight geophysical station with SEIS will not only provide key constraints on the interior of Mars and on our understanding of Mars evolution since its early Noachian-Phyllosian era but will renew a systematic seismic exploration of the terrestrial planets, Earth’s Moon and icy moons of giant planets by future planetary science missions.

### Electronic Supplementary Material

Below are the links to the electronic supplementary material.

(PDF 2.4 MB)

(MP4 294.4 MB)

## Figures and Tables

**Fig. 122:**
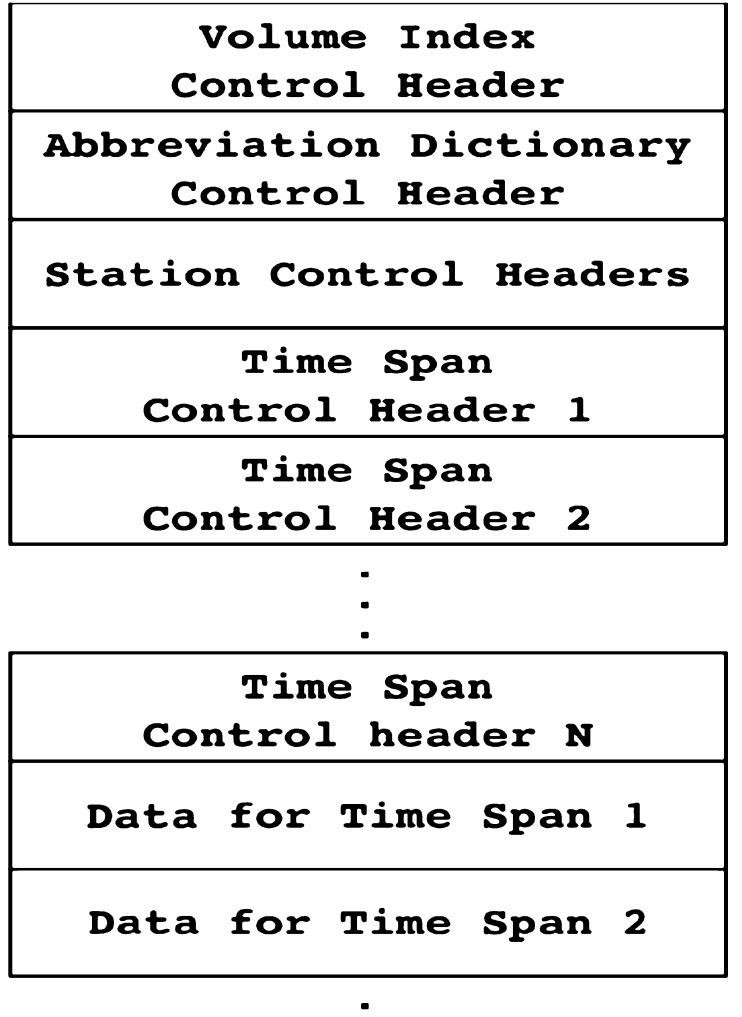
Seed volumes organization

**Fig. 123:**
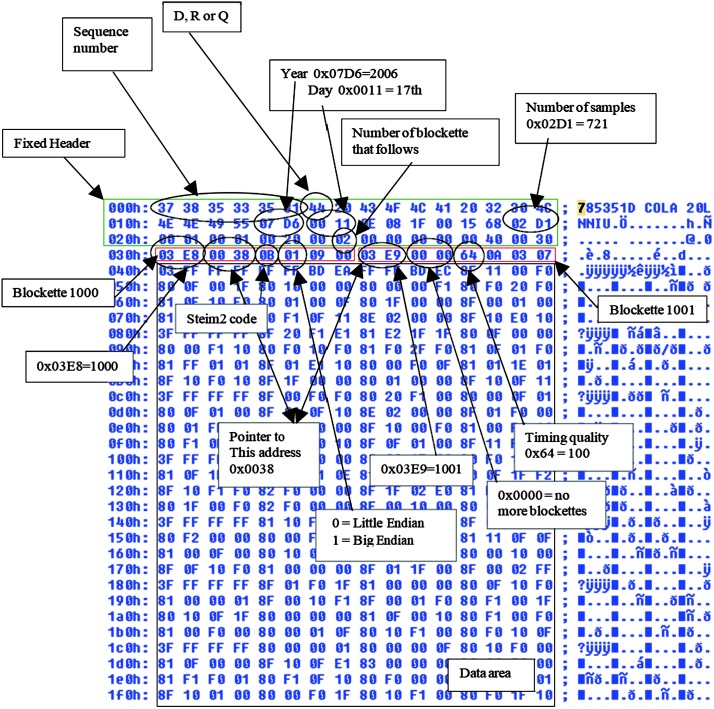
Hex dump of the station of cola.iu.liss.org. The most important fields are specified and linked to a description Table

## Appendix A: Single Station Analysis Strategies

The Concept Study Report listed and identified several single stations processing with respect the mission goals of the mission we recall in this Appendix. Normal modes, described in Sect. A.5 were nevertheless not integrated in the requirement flow. Based on the a-priori activity of Mars, only one quake per year might excite the fundamental spheroidal normal modes in the 0.01–0.02 Hz bandwidth with large enough amplitude compared to the expected instrument noise. An extended mission on several Mars years will therefore greatly improve the possibility for such detection, in addition to the possible long stack of SEIS signals for hum search.

### A.1 Event Location by P–S and Back-Azimuth (L1-6, L1-7)

The distribution of seismic activity is determined by monitoring the teleseismic body wave frequency band ($\sim 0.1\mbox{--}2.5~\mbox{Hz}$) for seismic events. The approximate epicentral distances are derived from the differential P–S arrival time on the vertical record. Initially, the distance error will be $\sim 10\%$ (reflecting the range of *a priori* estimates of Mars velocity models). The refinement of heat flow and crustal thickness (from HP^3^ and SEIS, respectively) will produce better constraints on upper mantle temperature. Together with SNC constraints on upper mantle mineralogy and detailed mineralogical modelling, this will result in further improvements to event locations and upper mantle seismic velocities.

The back-azimuth of the initial P arrival gives the direction to the source of the P wave. This is measured using the horizontal components, yielding an error of $\pm 10^{\circ}$ in azimuth for conservative projected levels of horizontal component noise, resulting in a roughly $\pm 15\%$ uncertainty in location. Events can thus be roughly localized within a $\sim 150~\mbox{km}$ radius at distances of 1000 km.

With an approximate location (augmented by guidance from observed geology), the spatial distribution and magnitude (from seismic amplitude and the approximate distance) of seismicity can be determined. This is a fundamental parameter of the seismic environment of a planet. See more in Böse et al. (2017), Clinton et al. (2018, this issue).

### A.2 Seismic Phase Analysis (L1-3, L1-5)

As noted above, the joint determination of P and S arrival times provides an estimate of epicentral distance to $\sim 15\%$ and RISE reduces the uncertainty in core radius to 200 km. With these constraints on the ray paths we can use the refined interior structure models with synthetic seismogram analysis to identify later-arriving phases. The additional differential measurements of arrival times, such as PcP–P PcS–S, ScS–S, as well as comparison of their relative amplitudes to P, provide additional constraints on the seismic velocities and attenuation in the deep mantle of Mars. These constraints help refine the core size estimate and place bounds on lower mantle discontinuities.

### A.3 Receiver Function Method (L1-2)

When a P or S wave strikes a discontinuity, it generates reflected and transmitted waves of both P and S. So waves from distant quakes passing through a layered medium like a crust/upper mantle can generate complicated seismograms containing many echoes. Such seismograms can be processed to generate simplified artificial waveforms (called receiver functions) (Phinney 1964; Langston 1979). These can be inverted to yield the variation of shear velocity with depth and are particularly sensitive to strong velocity discontinuities. Receiver functions are a powerful tool for studying the depth to the crust-mantle boundary or to other layers within the crust and are computed from single seismograms without using source location or time. This method has been widely used on the Earth and was successfully applied to the Moon (Vinnick et al. 2001). See more in Panning et al. (2017) and derivative techniques for constraining the crust (Knapmeyer-Endrun et al. 2018).

### A.4 Surface Wave Dispersion (L1-1, L1-3)

Surface waves are low-frequency seismic waves that propagate in the crust and upper mantle due to the presence of the free surface. By sampling the crust, lithosphere and upper-mantle, surface waves are an important source of information. The depths to which surface waves are sensitive depend on frequency, with low-frequency waves “feeling” greater depths and thus propagating at higher speeds. This results in dispersion, with low-frequency waves arriving earlier than higher frequencies. The details of the relation between frequency and group velocity are directly relatable to subsurface structure. They are extremely sensitive to the crustal thickness and variations $\geq 10\%$ are typical for crustal variations of 20 km. The sensitivity to the upper mantle is also important, as the group velocity of surface waves (or the differential group velocity between the fundamental and the overtones) varies by 5–10% for different models, as listed in Panning et al. (2017) and Smrekar et al. (2019, this issue).

In order to obtain velocity from arrival time, an estimate of the distance to the source or propagation distance between two arrivals shall be used. This can be obtained from multiple arrival of Rayleigh waves (R1–R2–R3) as proposed by Panning et al. (2015). This can also be obtained from P–S and from the R1–R2 See more details in Zheng et al. (2015), Khan et al. (2016) and Panning et al. (2017).

### A.5 Normal Modes and Hum Analysis (L1-1, L1-3)

For a single seismometer, the most effective techniques for studying deep structure use normal mode frequencies, which do not require knowledge of the source location. Normal mode spectral peaks from 5–20 mHz (sensitive to mantle structure) can be identified for a detection noise level of $10^{-9}~\mbox{m}/\mbox{s}^{2}/\mbox{Hz}^{1/2}$ (Lognonné et al. 1996, 2016; Gudkova and Zharkov 2004). This can be accomplished by single-seismogram analysis of a large quake of moment $\geq 2 \times 10^{17}~\mbox{N}\,\mbox{m}$ (equivalent magnitude $\sim 5.5$). About three such quakes are expected during one Mars year. The SNR of the mode peaks can be further improved by stacking multiple quakes with lower magnitudes. Due to the size of the planet, however, the frequencies of the modes, for a given angular order, are typically twice those on Earth.

Similar techniques can be applied to the background noise generated globally by atmospheric dynamics (Kobayashi and Nishida 1998) the so-called seismic “hum.” Calculations based on excitation by turbulence in the boundary layer that do not take in account resonance effects, non-turbulent wind and pressure variations associated with atmospheric circulation (Tanimoto 1999, 2001) yield amplitudes for Mars $\sim 0.1~\mbox{nanogal}$, a factor of 2–3 smaller than on Earth. See more in Lognonné and Johnson (2015), Lognonné et al. (2016), Panning et al. (2017), Schimmel et al. (2018), Nishikawa et al. (2019, this issue), Bissig et al. (2018).

## Appendix B: SEIS-AC Gains

This appendix provides the gains of SEIS AC for the different channels.

### B.1 Science and Engineering Conversion

Table 23 shows the gain and offsets required to convert the data into physical value, i.e. voltage or resistance. The general conversion is for example VBB vel $\mbox{Voltage}=(\mbox{ADC\_Data}-\mbox{offset})/\mbox{Gain}$. The Least Significant Bit (LSB), defined as $1/\mbox{Gain}$ is also given, as well as the Full Scale Range.

**Table 23:** Science and Engineering Data conversion factors

Parameter	FSR	Gain	Offset	LSB	Physical measurement
VBB VEL & POS*	50 V	335544.32	8388607	$2.98~\upmu\mbox{V}$	Voltage
SP VEL	25 V	671088.64	8388607	$1.49~\upmu\mbox{V}$	Voltage
VBB Eng Temp (QM)	896.25 Ω	68.725	−13291	$13.68~\mbox{m}\Omega$	Ohm
VBB Eng Temp (FM/FS)	896.25 Ω	73.1225	−16899.33	$13.68~\mbox{m}\Omega $	Ohm
SCIT (QM)	1432.66 Ω	11693.619	0	$85.39~\upmu \Omega $	Ohm
SCIT (FM/FS)	1432.66 Ω	11710.497	0	$85.39~\upmu \Omega $	Ohm

### B.2 House Keeping Data

Table 24 shows the gain and offsets required to convert the data into physical data, i.e. voltage, current and resistance. For example, Ch2 $\mbox{Voltage}=(\mbox{ADC\_Data}-\mbox{offset})/\mbox{Gain}$.

**Table 24:** FM HK Data conversion factors

Ch	Channel	Gain	Offset	Expected value (nominal mode)	Units
0	Ignore this data				
1	SP-HK1-MPOS1 [V]	6553.5	32767.5	±3	V
2	SP-HK2-MPOS1 [V]	6553.5	32767.5	±3	V
3	SEIS-DC+13VV [V]	2510.3846	0	13	V
4	CAL1-HKT [Ohm]	78.0941	−36780	1000	Ohm
5	SP-HK1-MPOS2 [V]	6553.5	32767.5	±3	V
6	SP-HK2-MPOS2 [V]	6553.5	32767.5	±3	V
7	SEIS-DC+15VA [mA]	658.6615	0	23.5	mA
8	VBB2-PXT [Ohm]	78.0941	−36780	1090	Ohm
9	SP-HK1-MPOS3 [V]	6553.5	32767.5	±3	V
10	SP-HK2-MPOS3 [V]	6553.5	32767.5	±3	V
11	SEIS-DC-13VV [V]	−2491.8462	0	−13	V
12	VBB3-PXT [Ohm]	78.0941	−36780	1090	Ohm
13	SP-HK1-TEMP-FB [V]	6553.5	32767.5	−3.65	V
14	SP-HK2-TEMP-FBE [V]	6553.5	32767.5	−3.65	V
15	SEIS-DC-15VA [mA]	−1324.667	744	13.5	mA
16	VBB3-HKT [Ohm]	78.0941	−36780	1090	Ohm
17	SEIS-DC+7VAV [V]	4657.7143	0	7	V
18	SP-HK1-SP1-TEMP [V]	6553.5	32767.5	−4.61	V
19	SP-HK2-SP1-TEMPE [V]	6553.5	32767.5	−5	V
20	SEIS-DC+7VAA [mA]	43.64138	0	275	mA
21	VBB1-HKT [Ohm]	78.0941	−36780	1090	Ohm
22	SP-HK1-SP2-TEMP [V]	6553.5	32767.5	−3.5	V
23	SP-HK2-SP2-TEMPE [V]	6553.5	32767.5	−2.9	V
24	SEIS-DC-10VV [V]	−3287.8	0	−10	V
25	VBB2-HKT [Ohm]	78.0941	−36780	1090	Ohm
26	SP-HK1-SP3-TEMP [V]	6553.5	32767.5	−3.15	V
27	SP-HK2-SP3-TEMPE [V]	6553.5	32767.5	−4.2	V
28	SEIS-DC-10VA [mA]	−343.3294	1138	−36	mA
29	VBB1-PXT [Ohm]	78.0941	−36780	1000	Ohm
30	SP-HK1+VREF [V]	6553.5	32767.5	3	V
31	SP-HK2-VREF [V]	6553.5	32767.5	−3	V
32	ACQ-HKT [Ohm]	78.0941	−36780	1090	Ohm
33	SEIS-DC+1V2V [V]	13108.3333	0	1.25	V
34	SEIS-AC+5VREF [V]	6553.6	0	5	V
35	SEIS-DC+1V2VA [mA]	62.0908	0	135	mA
36	DC-HKT [Ohm]	78.0941	−36780	1100	Ohm
37	SEIS-DC+3V3VA [mA]	105.0178	0	150	mA
38	SEIS-AC-6VSV [V]	3005.481	50506.63	−6	V
39	SEIS-DC+3V3V [V]	9912.7273	0	3.5	V
40	SEIS-AC+6VSV [V]	9154.875	0	6	V
41	SEIS-DC-5VV [V]	−6553.8	0	n/a	V
42	CTL-HKT [Ohm]	78.0941	−36780	1090	Ohm
43	SEIS-DC+5VV [V]	6553.5	0	n/a	V
44	SEIS-AC+6VSA [mA]	260.672	0	30	mA
45	SEIS-DC-5VA [mA]	−58.385	744	n/a	mA
46	CAL2-HKT [Ohm]	78.0941	−36780	1000	Ohm
47	SEIS-DC+5VA [mA]	58.385	0	n/a	mA

There is no expected value for channels 41, 43 (SEIS-DC $\pm 5~\mbox{VV}$) and 45, 47 (SEIS-DC ±5 VA) in nominal mode because in this mode no motor operation is performed.

The SP POS output is recorded with a FSR of 10 V ($\pm 5~\mbox{V}$) as an HK. The LSB after averaging is $152.59~\upmu\mbox{V}$ while the LSB of the 12 bit AD is $= 12.805~\mbox{m}\Omega$.

ALL HK TEMP have a LSB after averaging of $12.805~\mbox{m}\Omega$ while the 12 bits LSB is $204.88~\mbox{m}\Omega$. They are recorded with a FSR of $839.18~\Omega$ ($470.97~\Omega$ to $1310.15~\Omega$).

VBB PXT are VBB temperature sensors in VBB-PE boxes. HK voltages and currents have different LSBs that can be calculated in a similar way. FSR can be calculated using formula below in which minimum code of zero and maximum code of $2^{16}-1 = 65535$ shall be inserted: $\mbox{physical value}~[\mbox{V or mA}] = (\mbox{code}-\mbox{offset})/\mbox{Gain}$.

## Appendix C: MiniSEED Format Description

### C.1 SEED Data Description

#### C.1.1 Overview

InSight SEIS data as well as APSS data (for seismic use) will be released in SEED format (FDSN 2012). We briefly recall here this format as well as the general guidelines chosen by the project for SEIS data description. The Standard for the Exchange of Earthquake Data (SEED) format includes a data volume containing waveforms and a header, called dataless volume. SEED was designed for use by the earthquake research community, primarily for the exchange between institutions of unprocessed earth motion data.

A dataless SEED volume contains the normal Volume, Abbreviation and Station Control Headers. The purpose of these volumes is to provide an alternate method for making sure that various Data Centers have current and correct information for seismic stations.

It contains the metadata including instrument responses, instrument coordinates, compression type, etc. A dataless, by definition, contains no “data”, in the sense that no waveform data are included, only headers. It of course can be used in combination with a miniSEED volume which is a data-only volume.

It shall be noted that researchers interested mostly in APSS for atmospheric research shall use the APSS data from PDS, as the SEED is not able to fully represent the complex temperature dependency of the APSS data. APSS in SEED shall therefore be mostly used for decorrelation and diagnostic purposes on SEIS.

#### C.1.2 Seed Volume Description

Seed physical volumes may contain one or more logical volumes. Seed logical volumes may be dataless volumes or may contain waveforms and have an organization described by Fig. 122.

#### C.1.3 MiniSEED Information

All data information will be encrypted either in the miniSEED (mSEED) header or in the SEED dataless which will be provided in the released data. Details are given for the mSEED header and in for the SEED dataless. According to the SEED Reference Manual the mSEED data-packet is composed of the following main fields:

1. A fixed header of 48 bytes
2. One or two blockettes; always blockette coded as number 1000, optional the blockette coded as number 1001
3. Data field
  - mSEED header contains information about the waveform it contains such as
  - Station code
  - Location identifier
  - Channel identifier
  - Network code
  - Record start time
  - Details of mSEED format can be found in Appendix

#### C.1.4 InSight Dataless Description

InSight team choose SEED format to distribute data collected during the mission to ensure that data handling will be as clear and easy as possible. Indeed, the SEED format contains a maximum number of parameters and processing descriptions on the way the data has been produced, including by providing all the instrument information necessary to get a complete description of the way the onboard flight software processed the data.

#### C.1.5 SEED Network and Station

As the landing site is close to the Elysium mount, the InSight station name for the final configuration is Elysium Planitia and its acronym is ELYSE while data produced during cruise post-landing and during deployment will be identified as CRUI(1/2) and ELYS(0/1). The station name for synthetic is SYNT1 (e.g. Ceylan et al. 2017; Clinton et al. 2018). The position of the station is given in Mars geographic coordinates for ELYSE, while the position of the spacecraft with respect to Earth is chosen for CRUI coordinates at the time of the first data on the cruise check.

The mission network label is XB and is used for data produced during the test, cruise, commissioning and normal science operation mission phases. Therefore, data may have different network codes such as 7I for the test data produced during the pre and post-flight phases and 7J for the synthetic data.

#### C.1.6 Channel Naming Convention

Channel naming convention is as close as possible to SEED channel naming rules and is given in Table 25 Channel name components are Frequency ID, Instrument Label, Orientation code. Full channels names can be found in this document annex.

**Table 25:** Synthesized channel list

Channel naming	Label
High Gain Seismometer	H
Low Gain Seismometer	L
Mass Position Seismometer	M
Pressure	D
Magnetometer	F
Temperature	K
Wind	W
Synthetized beam data	Z
Non-specific instruments	Y
Instrument code	

#### C.1.7 SEED Informations

The SEED dataless volume contains the full description of data in the mSEED volume and may contain the blockettes describing the Volume (Table 26), Abbreviation and configurations (Table 27), Station informations (Table 28). Included in the station section, the channel identifier blockette is repeated for each channel instance (different starting/ending time) and is followed by the suitable set of description blockettes. The successions of processes that lead to the production of the data are described in blocks called stages.

**Table 26:** Volume section blockettes

Blockette No.	Blockette name	Blockette description
010	Volume Identifier	Record length, Beginning and ending time
011	Volume Station Header Index	List of station names that the volume may contain

**Table 27:** Abbreviation and configuration section blockettes

Blockette No.	Blockette Name	Blockette description
030	Data format dictionary	Dictionary referenced in the channel description field
031	Comment dictionary	Dictionary of comments used in blockette 59
033	Generic abbreviation	List of used abbreviations such as instrument type
034	Units abbreviation	List of used measurement unit abbreviations such as M/S, V
035	Beam configuration	List of the channels used for onboard generation of VBB/SP hybrid output

**Table 28:** Information encrypted in the SEED dataless for station informations

Blockette No.	Blockette name	Blockette description
050	Station identifier	Station short name, full name, localization, starting and ending time, network name
052	Channel identifier	Channel identifier, location code, instrument identifier, unit of signal, sample rate, starting and ending time
053	Poles and zeros	Contains real and imaginary poles and zeros and errors of the analog transfer function of the sensors or of the LVL
054	Response (filter)	Contains FIR filter coefficients of either the SEIS AC or the SEIS F/SW
057	Decimation	Contains the input sampling rate, decimation ratio and filters delay of either the SEIS AC or the SEIS F/SW
058	Sensitivity gain	Contains the sensitivity gain of the sensors and SEIS A/C
059	Comment blockette	Index to the comment dictionary (for instrument temperature sensitivity)
062	Response polynomial	Polynomial components used as $V=P_{0}+P_{1}^{*}S+P_{2}^{*}S^{2}+ P_{3}^{*}S^{3}\ldots $ (for temperature sensors)

### C.2 MiniSEED Fixed Data Header Fields

#### C.2.1 Overview

Data will be stored in the miniSEED (mSEED) files (see Fig. 123 for an example), each one with an header described in Table 29 and two blocquettes of additional informations, described in Sects. C.2.2 and C.2.3. The size of each packet is 512 bytes and it contains the starting time of the data in UTC. As the samples number is known, the Time difference between two mSEED packet will provide information of the drift of the SEIS-AC clock.

**Table 29:** Informations encrypted in the miniSEED header

	Field name	Type	Byte position	Length (bytes)
1	Sequence number	ASCII	1–6	6
2	Data Header quality indicator	ASCII	7	1
3	Reserved byte	ASCII	8	1
4	Station code	ASCII	9–13	5
5	Location identifier	ASCII	14–15	2
6	Channel identifier	ASCII	16–18	3
7	Network code	ASCII	19–20	2
8	Record start time	BTIME	21–30	10
9	Number of samples	UWORD	31–32	2
10	Sample rate factor	WORD	33–34	2
11	Sample rate multiplier	WORD	35–36	2
12	Activity flags	UBYTE	37	1
13	I/O flags	UBYTE	38	1
14	Data quality flags	UBYTE	39	1
15	Number of blockettes that follow	UBYTE	40	1
16	Time correction	LONG	41–44	4
17	Offset to beginning of data	UWORD	45–46	2
18	Offset to beginning of first blockette	UWORD	47–48	2

#### C.2.2 The Blockette Type 1000 Specifications

This is a Data Only SEED Blockette. It is 8 bytes long (Table 30) and has the following structure:

1. The blockette code that contains always the number 1000.
2. The offset to the beginning of the next blockette.
3. The encoding format according to the following basic table:
  Code Encoding Format
  0 ASCII text, byte order as specified in field 4
  1 16 bit integers
  2 24 bit integers
  3 32 bit integers
  4 IEEE floating point
  5 IEEE double precision floating point
  10 STEIM (1) Compression
  11 STEIM (2) Compression
4. The word order. According to the text in the SEED reference manual a zero (0) stands for little-endian and a one (1) for big endian.
5. The exponent of a base of 2 to specify the record length. In LISS miniSEED it is 9 that means $2^{9}=512$.
6. Reserved.

**Table 30:** Informations encrypted in the 1000 blockette

	Field name	Type	Length (bytes)
1	Blockette code	UWORD	2
2	Offset to the beginning of the next blockette	UWORD	2
3	Encoding format	UBYTE	1
4	Word order	UBYTE	1
5	Data record length	UBYTE	1
6	Reserved	UBYTE	1

#### C.2.3 The Blockette Type 1001 Specifications

This blockette is 8 bytes long (Table 31) and has the following structure:

1. Blockette code that contains always the number: 1001.
2. The offset to the beginning of the next blockette.
3. Timing quality. Can be used by the digitizer manufacturer to estimate the time quality of this data packet form (0 to 100% of accuracy).
4. Precision of the start time down to microseconds. This filed is present to improve the accuracy of the time stamping given by the fixed header time structure.
5. Reserved byte.
6. Frame count. Is the number of 64 byte compressed data frames in the 4k record. (maximum of 63).

**Table 31:** Informations encrypted in the 1000 blockette

	Field name	Type	Length (bytes)
1	Blockette code	UWORD	2
2	Offset to the beginning of the next blockette	UWORD	2
3	Timing quality	UBYTE	1
4	Microseconds	BYTE	1
5	Reserved	UBYTE	1
6	Frame count	UBYTE	1

### C.3 SEIS-INSIGHT Channel List

The naming of all SEIS channels is give in Table 32 for the VBB Velocity Channels, Table 33 for the VBB POS channels, Table 34 for the Temperature channels, Table 35 for the APSS channels, Table 36 for the SP channels and Table 37 for the onboard computed channels.

**Table 32:** Channel naming of the VBB velocity channels

Channel	Loc ID	100 Hz	10–80 Hz	2–5 Hz	1 Hz	Chan flag
VBB 1 Velocity High Gain Science mode	00	HHU	BHU	MHU	LHU	G
VBB 1 Velocity Low Gain Science mode	05	HLU	BLU	MLU	LLU	G
VBB 1 Velocity High Gain Engin. Mode	10	HHU	BHU	MHU	LHU	G
VBB 1 Velocity Low Gain Engin. Mode	15	HLU	BLU	MLU	LLU	G
VBB 2 Velocity High Gain Science mode	00	HHV	BHV	MHV	LHV	G
VBB 2 Velocity Low Gain Science mode	05	HLV	BLV	MLV	LLV	G
VBB 2 Velocity High Gain Engin. Mode	10	HHV	BHV	MHV	LHV	G
VBB 2 Velocity Low Gain Engin. Mode	15	HLV	BLV	MLV	LLV	G
VBB 3 Velocity High Gain Science mode	00	HHW	BHW	MHW	LHW	G
VBB 3 Velocity Low Gain Science mode	05	HLW	BLW	MLW	LLW	G
VBB 3 Velocity High Gain Engin. Mode	10	HHW	BHW	MHW	LHW	G
VBB 3 Velocity Low Gain Engin. Mode	15	HLW	BLW	MLW	LLW	G

**Table 33:** Channel naming of the VBB POS channels

Channel	Loc ID	1 Hz	0.1–0.5 Hz	Chan flag
VBB 1 Position High Gain Science mode	00	LMU	VMU	G
VBB 1 Position Low Gain Science mode	05	LMU	VMU	G
VBB 1 Position High Gain Engin. mode	10	LMU	VMU	G
VBB 1 Position Low Gain Engin. mode	15	LMU	VMU	G
VBB 2 Position High Gain Science mode	00	LMV	VMV	G
VBB 2 Position Low Gain Science mode	05	LMV	VMV	G
VBB 2 Position High Gain Engin. mode	10	LMV	VMV	G
VBB 2 Position Low Gain Engin. mode	15	LMV	VMV	G
VBB 3 Position High Gain Science mode	00	LMW	VMW	G
VBB 3 Position Low Gain Science mode	05	LMW	VMW	G
VBB 3 Position High Gain Engin. mode	10	LMW	VMW	G
VBB 3 Position Low Gain Engin. Mode	15	LMW	VMW	G

**Table 34:** Channel naming of the SEIS and VBB T channels

Temperature channels	Loc ID	1 Hz	0.1–0.5 Hz	0.01–0.05	<0.01	Chan flag
VBB 1 Temperature	00	LKU	VKU	UKU	RKU	H
VBB 2 Temperature	00	LKV	VKV	UKV	RKV	H
VBB 3 Temperature	00	LKW	VKW	UKW	RKW	H
Scientific Temperature A	00	LKI	VKI	UKI	RKI	H
Scientific Temperature B	05	LKI	VKI	UKI	RKI	H

**Table 35:** Channel naming of the APSS channels

APSS channels	Loc ID	10–80 Hz	2–5 Hz	1 Hz	0.1–0.5 Hz	0.01–0.05	<0.01	Chan flag
*Sensor 1*								
Wind horizontal speed	10	Not Applicable		LWS	VWS	UWS	RWS	W
Wind vertical speed	15			LWS	VWS	UWS	RWS	W
Wind direction	10			LWD	VWD	UWD	RWD	W
Atmosphere temperature	10			LKO	VKO	UKO	RKO	W
*Sensor 2*								
Wind horizontal speed	20			LWS	VWS	UWS	RWS	W
Wind vertical speed	25			LWS	VWS	UWS	RWS	W
Wind direction	20			LWD	VWD	UWD	RWD	W
Atmosphere temperature	20			LKO	VKO	UKO	RKO	W
*Composite*								
Wind horizontal speed	30			LWS	VWS	UWS	RWS	W
Wind vertical speed	35			LWS	VWS	UWS	RWS	W
Wind direction	30			LWD	VWD	UWD	RWD	W
Atmosphere temperature	30			LKO	VKO	UKO	RKO	W
Pressure (outside)	00	BDO	MDO	LDO	VDO	UDO	RDO	W
Pressure sensor temperature (inside)	10	BKI	MKI	LKI	VKI	UKI	RKI	H
Magnetomer1	00	BF1	MF1	LF1	VF1	UF1	RF1	G
Magnetomer2	00	BF2	MF2	LF2	VF2	UF2	RF2	G
Magnetomer3	00	BF3	MF3	LF3	VF3	UF3	RF3	G
Magnetometer temperature	00	BKM	MKM	LKM	VKM	UKM	RKM	H

**Table 36:** Channel naming of the SP channels

SP channels	Loc ID	100 Hz	10–80 Hz	2–5 Hz	1 Hz	Chan flag
SP1 (High Gain)	65	EHU	SHU	MHU	LHU	G
SP2 (High Gain)	65	EHV	SHV	MHV	LHV	G
SP3 (High Gain)	65	EHW	SHW	MHW	LHW	G
SP1 (Low Gain)	70	ELU	SLU	MLU	LLU	G
SP2 (Low Gain)	70	ELV	SLV	MLV	LLV	G
SP3 (Low Gain)	70	ELW	SLW	MLW	LLW	G

**Table 37:** Channel naming of the SP channels

Synthesized channels	Loc ID	100 Hz	10–80 Hz	2–5 Hz	1 Hz	Chan flag
SEISVELZ	55	HZC	BZC	MZC	LZC	S
SPZ	75	EZC	SZC	MZC	LZC	S
VBBR	80	HZC	BZC	MZC	LZC	S
SPR	20	HZC	BZC	MZC	LZC	S
ESTAVBB	40				LHZ	S
MAXVBB	45				LYZ	S
ESTASP	85				LLZ	S
MAXSP	90				LYZ	S
MAGZ	20		BFR	MFR	LFR	S
ESTAP1	50				LDO	S
ESTAP2	60				LDO	S
MAXP1	70				LYO	S
MAXP2	80				LYO	S
ESTAM	30				LFA	S
MAXM	40				LYA	S
