# Supplementary material for: SEIS: Insight’s Seismic Experiment for Internal Structure of Mars
Source: Space Sci Rev. 2019 Jan 28;215(1):12. doi: 10.1007/s11214-018-0574-6 (PMC6394762; doi:10.1007/s11214-018-0574-6)
Supplement: Supplementary file 1 — (PDF 2.4 MB) [file 11214_2018_574_MOESM1_ESM.pdf]

ROTATION MATRIX (R)

$$R = \begin{bmatrix} -\sqrt{3}/2 & -0.5 & 0 \\ +0.5 & -\sqrt{3}/2 & 0 \\ 0 & 0 & 1 \end{bmatrix}$$

TRANSLATION VECTOR (T)

$$T = \begin{bmatrix} 31.5 \\ 155 \\ 184.5 \end{bmatrix}$$

$$NODE/SEIS = \begin{bmatrix} R \end{bmatrix} \cdot \begin{bmatrix} NODE/SEIS\_ASM \end{bmatrix} + \begin{bmatrix} T \end{bmatrix}$$

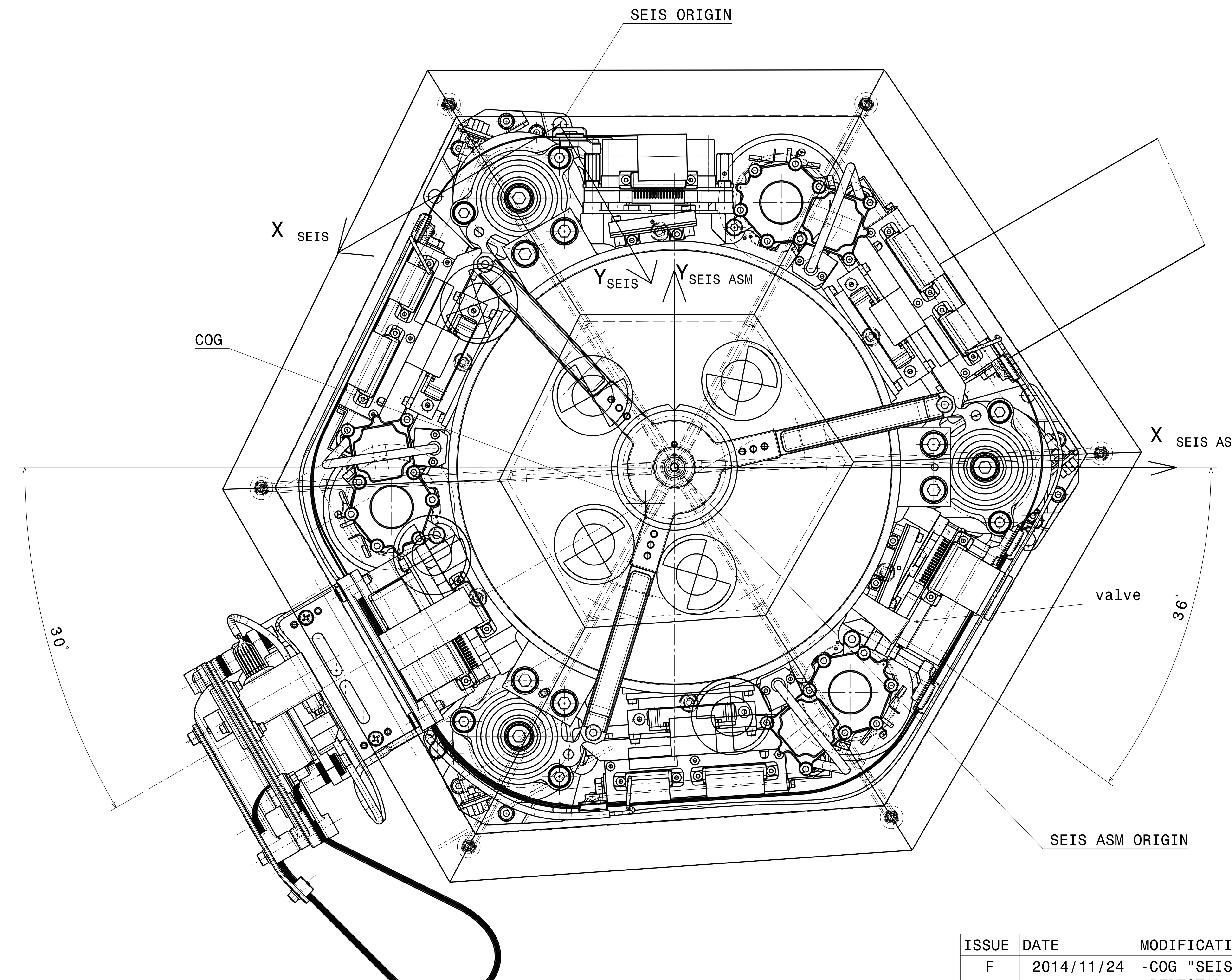

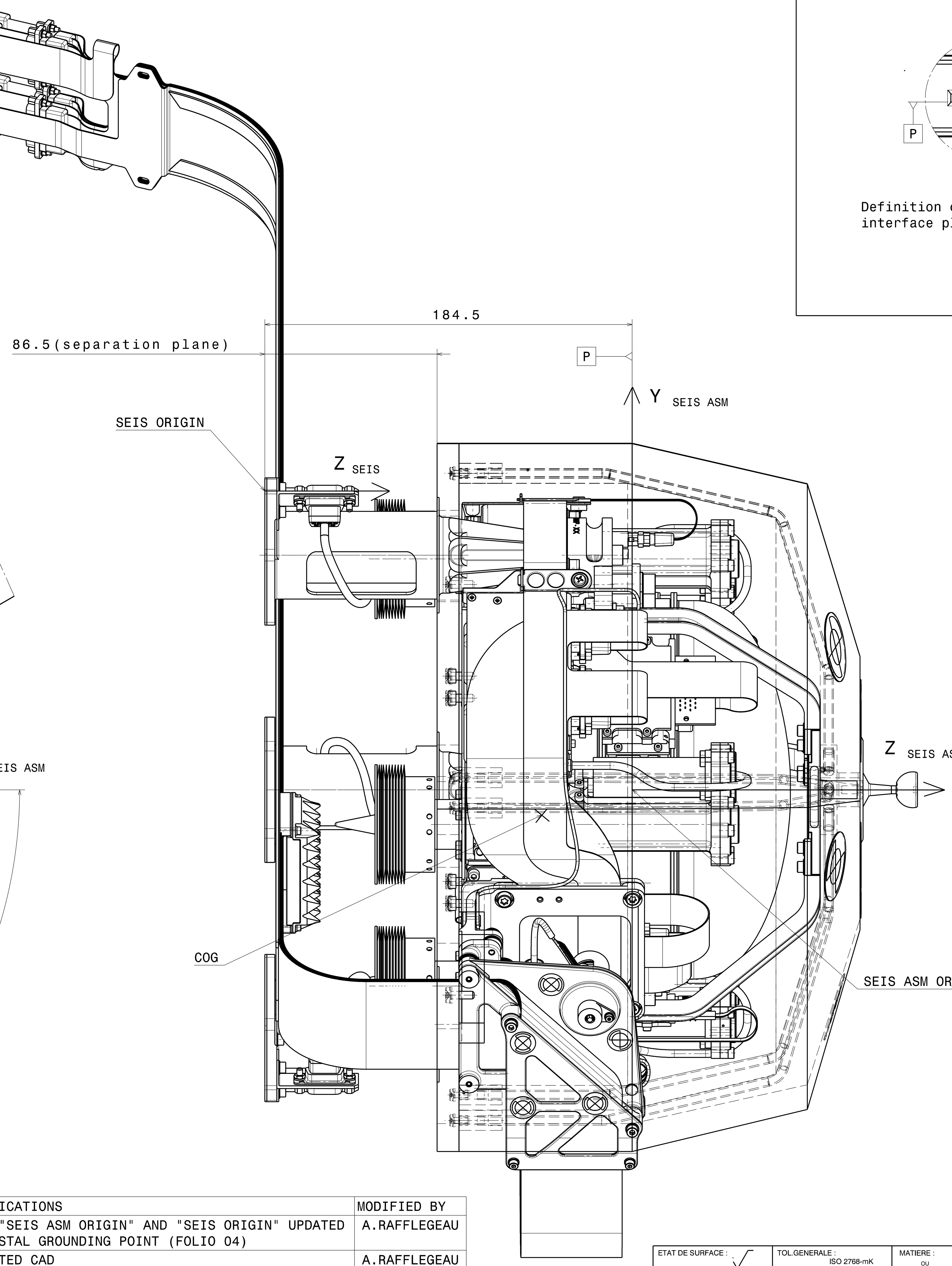

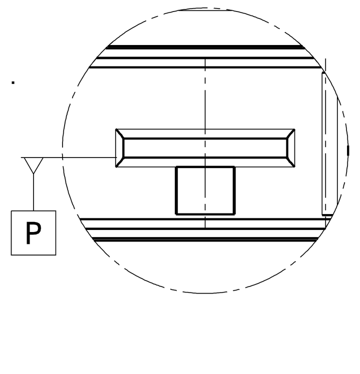

Definition of the sphere interface plane P

	SEIS ASM ORIGIN FRAME	
COG	X	-15.8
	Y	-12.7
	Z	-45.4

	SEIS ORIGIN FRAME	
COG	X	+51.2
	Y	+158.1
	Z	+139.1

SEIS-ASM frame Definition:
Origin goes through the sphere symmetry axis
X-Y plane is on the the sphere interface plane P
X axis oriented from the sphere center to the dampers located at 36° of the sphere valve
Z axis is perpendicular to the plane P oriented from the deck to the sphere
Y axis is oriented such that the reference frame forms a direct and orthogonal three-axis system

ISSUE	DATE	MODIFICATIONS	MODIFIED BY
F	2014/11/24	-COG "SEIS ASM ORIGIN" AND "SEIS ORIGIN" UPDATED<br>-PEDESTAL GROUNDING POINT (FOLIO 04)	A.RAFFLEGEAU
E	2014/09/17	-UPDATED CAD	A.RAFFLEGEAU
D	2014/03/30	-COG "SEIS ASM ORIGIN" AND "SEIS ORIGIN" UPDATED	A.RAFFLEGEAU
C	2014/02/13	-UPDATED CAD<br>-DETAIL B ADDED (folio 2)<br>-ACTUATOR DEPLOYEMENT CONNECTORS ADDED (folio 4)	A.RAFFLEGEAU
B	2013/10/18	-UPDATED CAD<br>-"origin SEIS ASM" ADDED (folio 1,2 and 3)<br>-"origin SEIS" ADDED (folio 1 and 2)<br>-$X_{SEIS\ ASM}$","$Y_{SEIS\ ASM}$" AND "$Z_{SEIS\ ASM}$" ADDED (folio 1,2 and 3)<br>-X_{SEIS}","Y_{SEIS}" AND "Z_{SEIS}" ADDED (folio 1 and 2)<br>-HOLE TABLE ADDED (folio 2)<br>-ROTATION MATRIX AND TRANSLATION VECTOR ADDED (folio 1)	A.RAFFLEGEAU
A	2013/09/23	-CREATION	A.RAFFLEGEAU

ETAT DE SURFACE : √	TOL.GENERALE : ISO 2768-mK	MATIERE : . OU .	TRAITEMENT: .

DESSINE PAR : A.RAFFLEGEAU — LE : 2014/11/24
VERIFIE PAR : C.IMBERT — LE : 2014/11/24
VISE PAR : . — LE : .
SERVICE EMETTEUR : DCT/TV/MT

CENTRE NATIONAL D'ETUDES SPATIALES
CENTRE SPATIAL DE TOULOUSE
18 AVENUE EDOUARD BELIN, 31401 TOULOUSE CEDEX 9
TEL : 05-61-27-31-31 FAX : 05-61-27-31-79

CE DOCUMENT EST LA PROPRIETE DU CNES ET NE PEUT ETRE COMMUNIQUE OU REPRODUIT SANS SON AUTORISATION

ECHELLE : 3/4 ./.
PROJECTION :

DESIGNATION :
SEIS ASSEMBLY
REFERENCE AXIS DEFINITION AND COG
.

FORMAT : A1
INDICE : F
PLANCHE : 1/4

NOM DU PLAN EN BD CATIA : .

N° DE PLAN : 2013-MT-INSIGHT-0002

DETAIL B
SCALE : 1:1
FREE COATING AREA (x3)

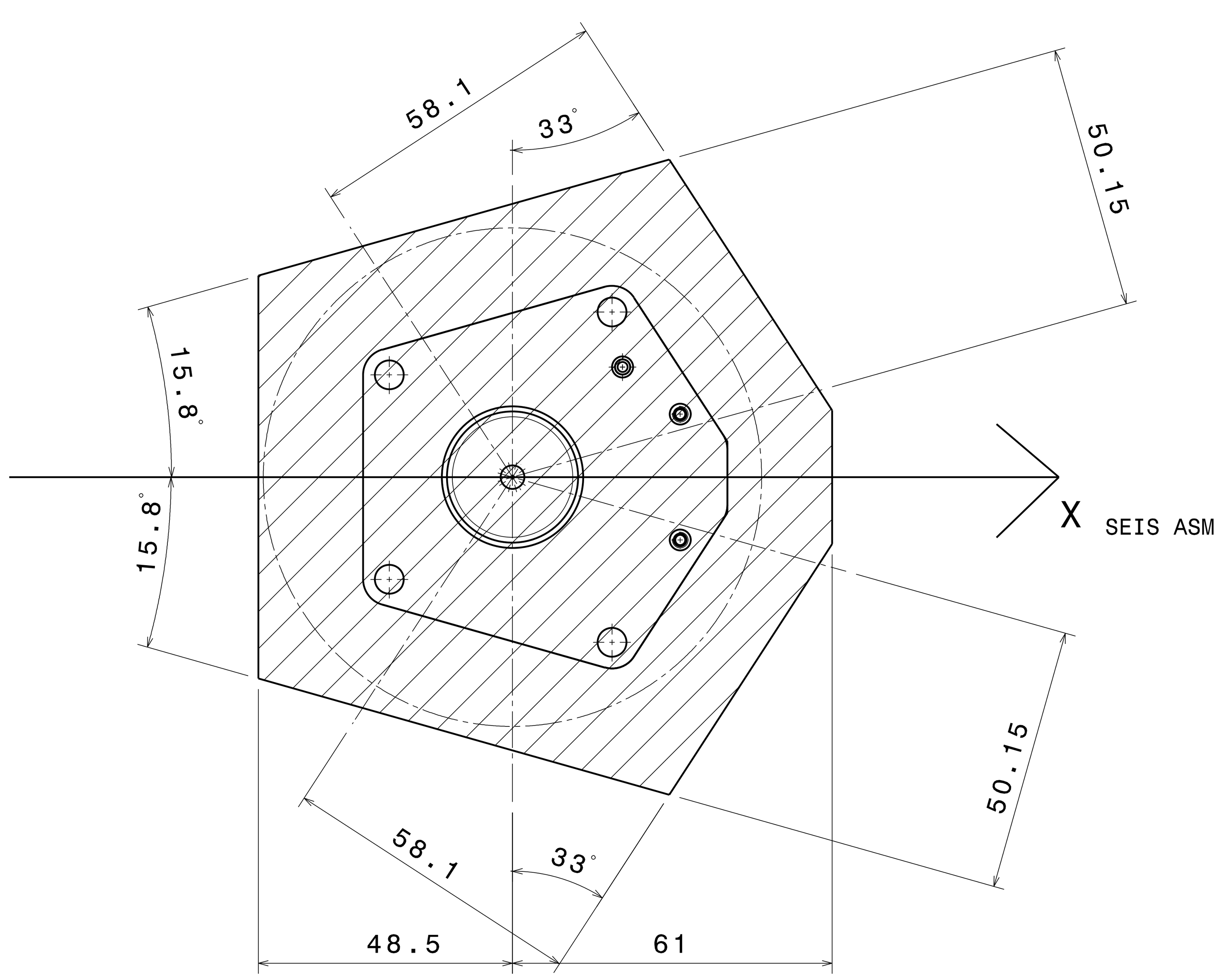

VIEW WITHOUT RWEB AND TETHER

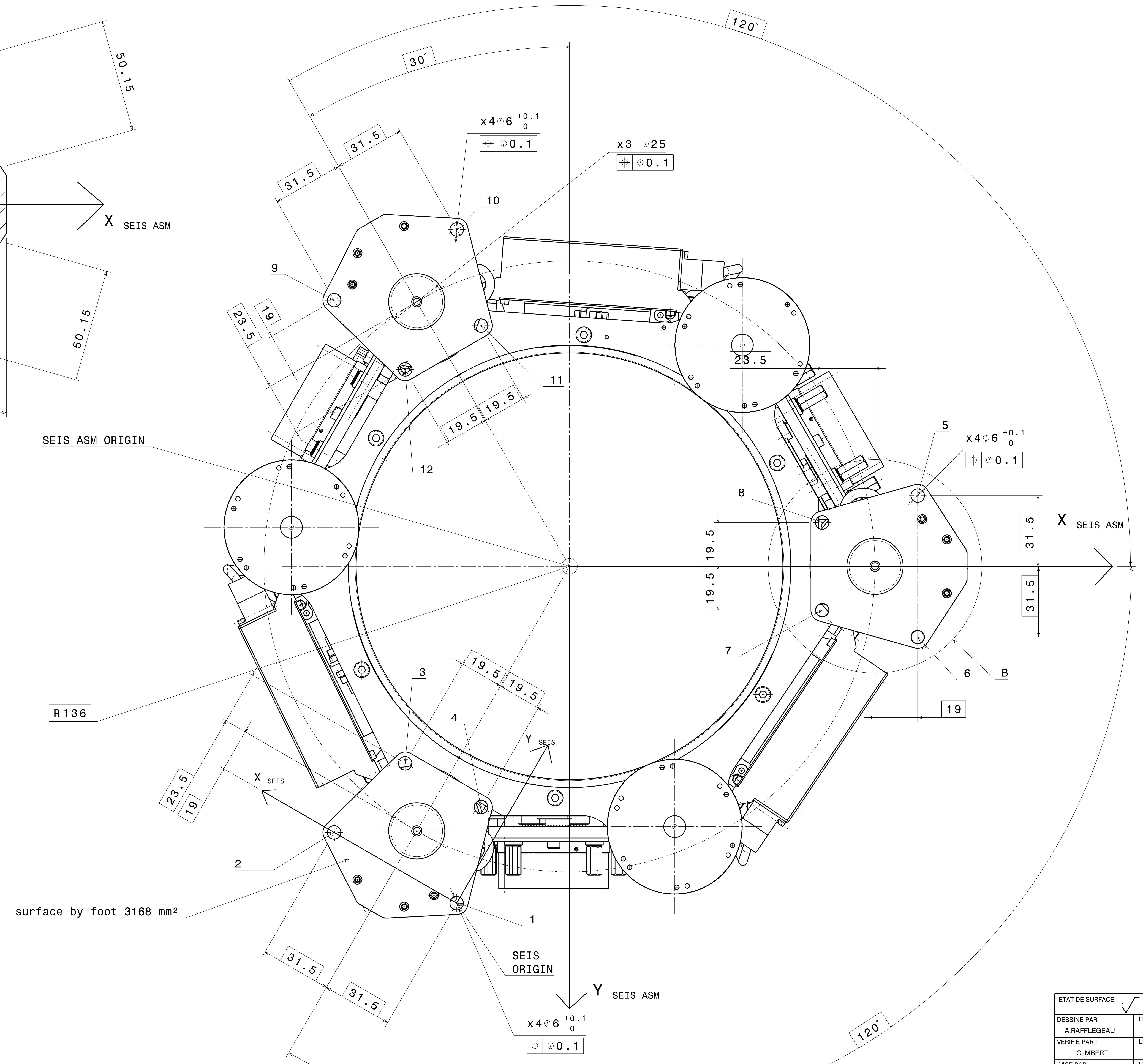

ALL CO-ORDONATES ARE IN SEIS FRAME			
HOLE NUMBER	X(mm)	Y(mm)	Z(mm)
1	0	0	0
2	63	0	0
3	51	42.5	0
4	12	42.5	0
5	-86.984	259.78	0
6	-118.484	205.22	0
7	-75.678	194.363	0
8	-56.178	228.137	0
9	181.484	205.22	0
10	149.984	259.78	0
11	119.178	228.137	0
12	138.678	194.363	0

ETAT DE SURFACE : √
TOL.GENERALE : ISO 2768-mK
MATIERE : . OU .
TRAITEMENT: .

DESSINE PAR : A.RAFFLEGEAU — LE : 2014/11/24
VERIFIE PAR : C.IMBERT — LE : 2014/11/24
VISE PAR : . — LE : .
SERVICE EMETTEUR : DCT/TV/MT

cnes
CENTRE NATIONAL D'ETUDES SPATIALES
CENTRE SPATIAL DE TOULOUSE
18 AVENUE EDOUARD BELIN, 31401 TOULOUSE CEDEX 9
TEL : 05-61-27-31-31 FAX : 05-61-27-31-79

CE DOCUMENT EST LA PROPRIETE DU CNES ET NE PEUT ETRE COMMUNIQUE OU REPRODUIT SANS SON AUTORISATION

ECHELLE : 1/1 ./.
PROJECTION :

DESIGNATION :
SEIS ASSEMBLY
DECK INTERFACE PLANE
.

FORMAT : A1
INDICE : F
PLANCHE : 2/4

NOM DU PLAN EN BD CATIA : .
N° DE PLAN : 2013-MT-INSIGHT-0002

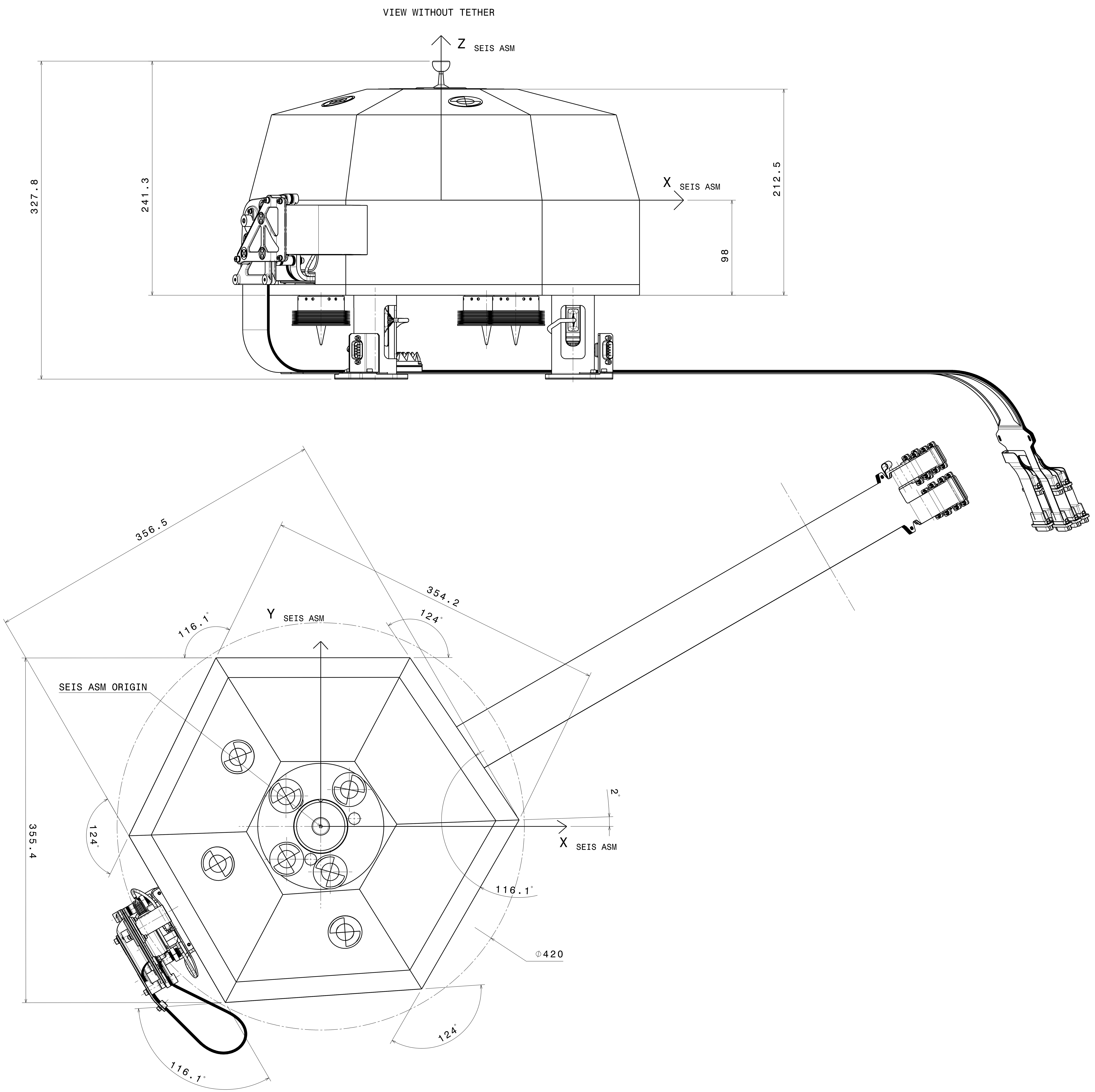

ETAT DE SURFACE : √	TOL.GENERALE : ISO 2768-mK	MATIERE : . OU .	TRAITEMENT: .
DESSINE PAR : A.RAFFLEGEAU	LE : 2014/11/24	CENTRE NATIONAL D'ETUDES SPATIALES	
VERIFIE PAR : C.IMBERT	LE : 2014/11/24	CENTRE SPATIAL DE TOULOUSE	
VISE PAR : .	LE : .	18 AVENUE EDOUARD BELIN, 31401 TOULOUSE CEDEX 9	
SERVICE EMETTEUR : DCT/TV/MT		TEL : 05-61-27-31-31 FAX : 05-61-27-31-79	

CE DOCUMENT EST LA PROPRIETE DU CNES ET NE PEUT ETRE COMMUNIQUE OU REPRODUIT SANS SON AUTORISATION

ECHELLE : 1/2 ./.	DESIGNATION :	FORMAT : A1
PROJECTION :	SEIS ASSEMBLY<br>VOLUME WITHOUT TETHER<br>WITH SHUNT	INDICE : F
NOM DU PLAN EN BD CATIA : .		PLANCHE : 3/4
N° DE PLAN :	2013-MT-INSIGHT-0002	

VIEW ACCORDING TO A

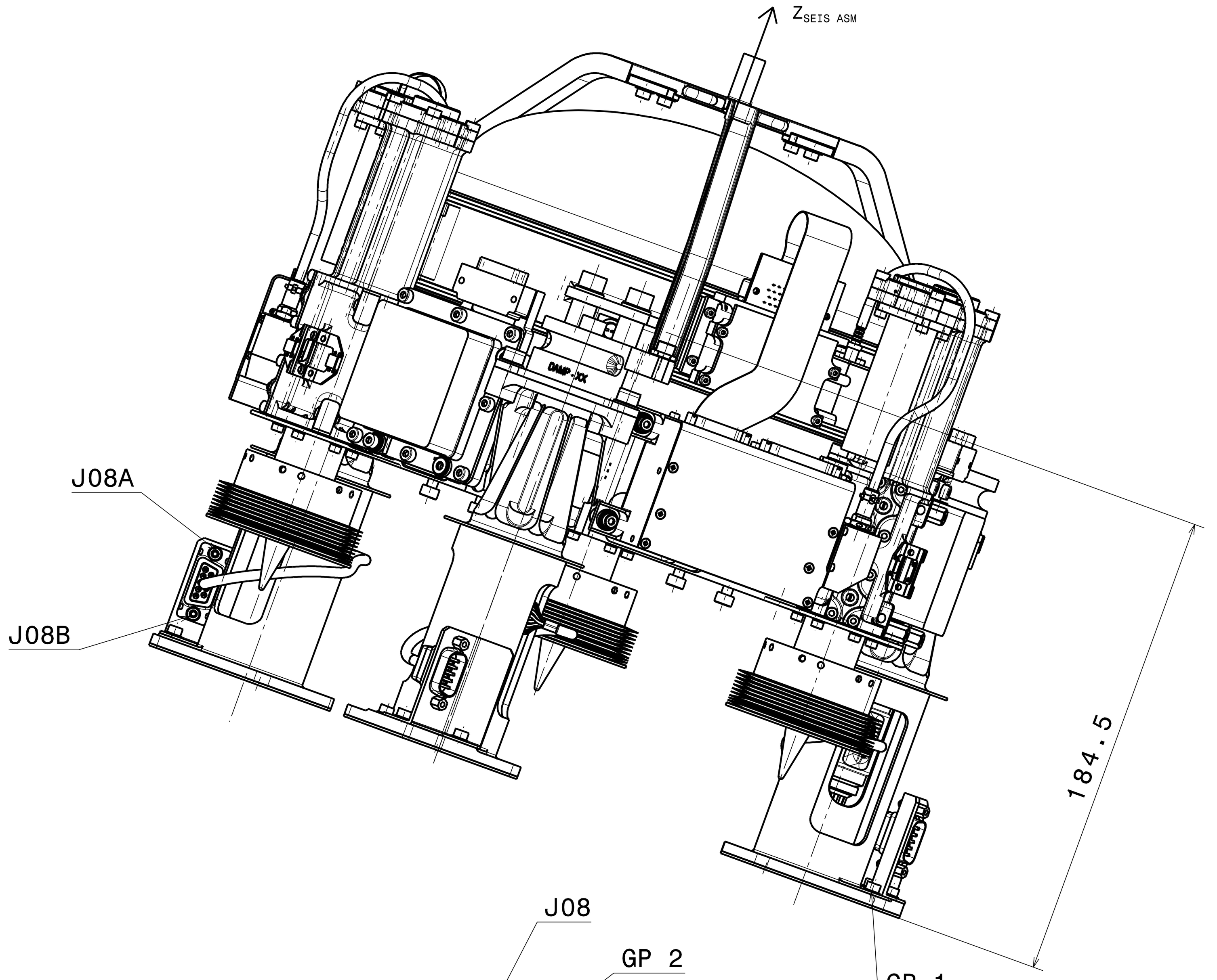

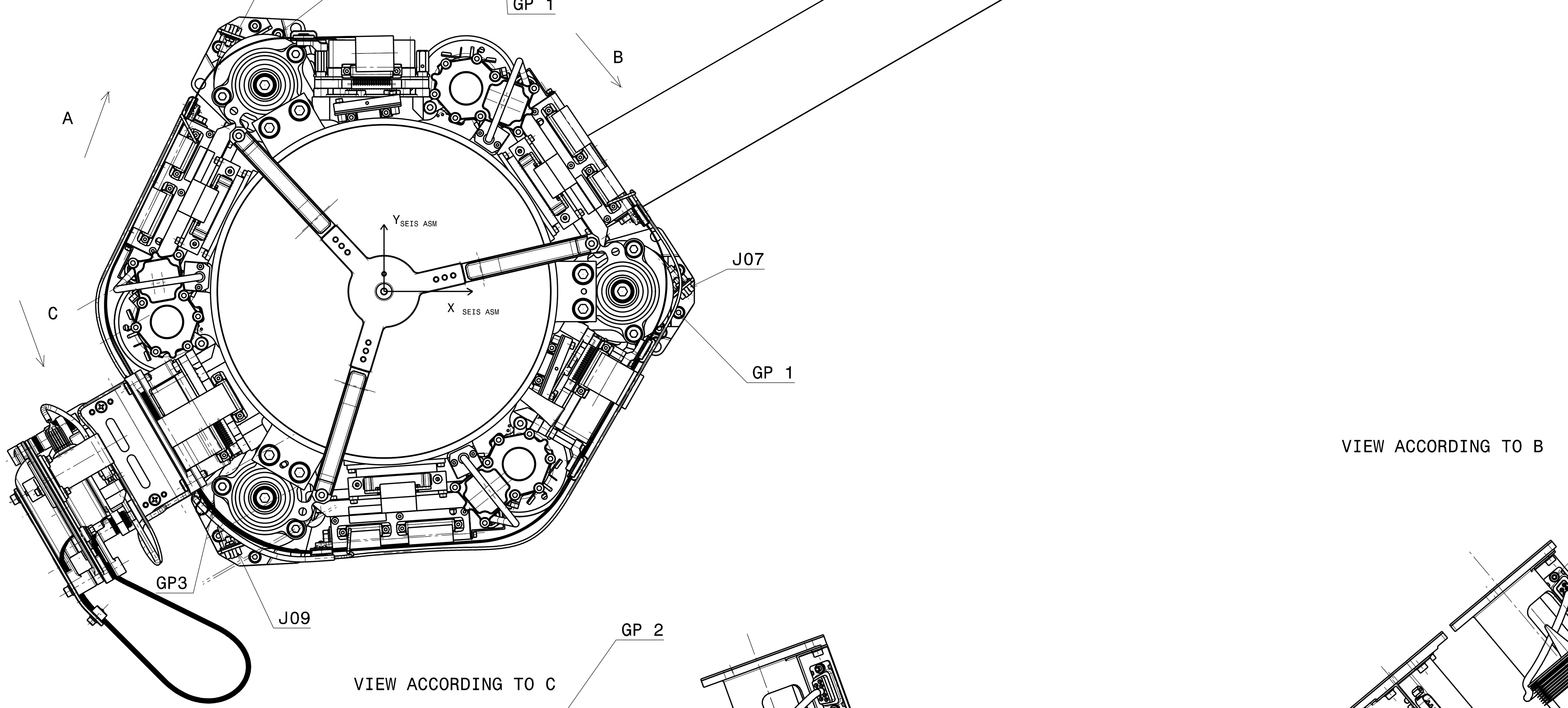

PEDESTAL CONNECTOR		SEIS ASSEMBLY FRAME X (mm)	Y (mm)	Z (mm)
J07A	#1	170.17	2.64	-142.01
J07B	#1	170.17	2.64	-167
J08A	#2	-87.36	146.05	-142.01
J08B	#2	-87.36	146.05	-167
J09A	#3	-87.36	-146.05	-142.01
J09B	#3	-87.36	-146.05	-167

NOTE :
-A CORRESPONDING TO PIN 1 CONNECTOR

PEDESTAL GROUNDING POINTS	SEIS ASSEMBLY FRAME X (mm)	Y (mm)	Z (mm)
GP 1	157	-21	-178.5
GP 2	-60.31	146.47	-178.5
GP 3	-96.69	-125.47	-178.5

VIEW ACCORDING TO C

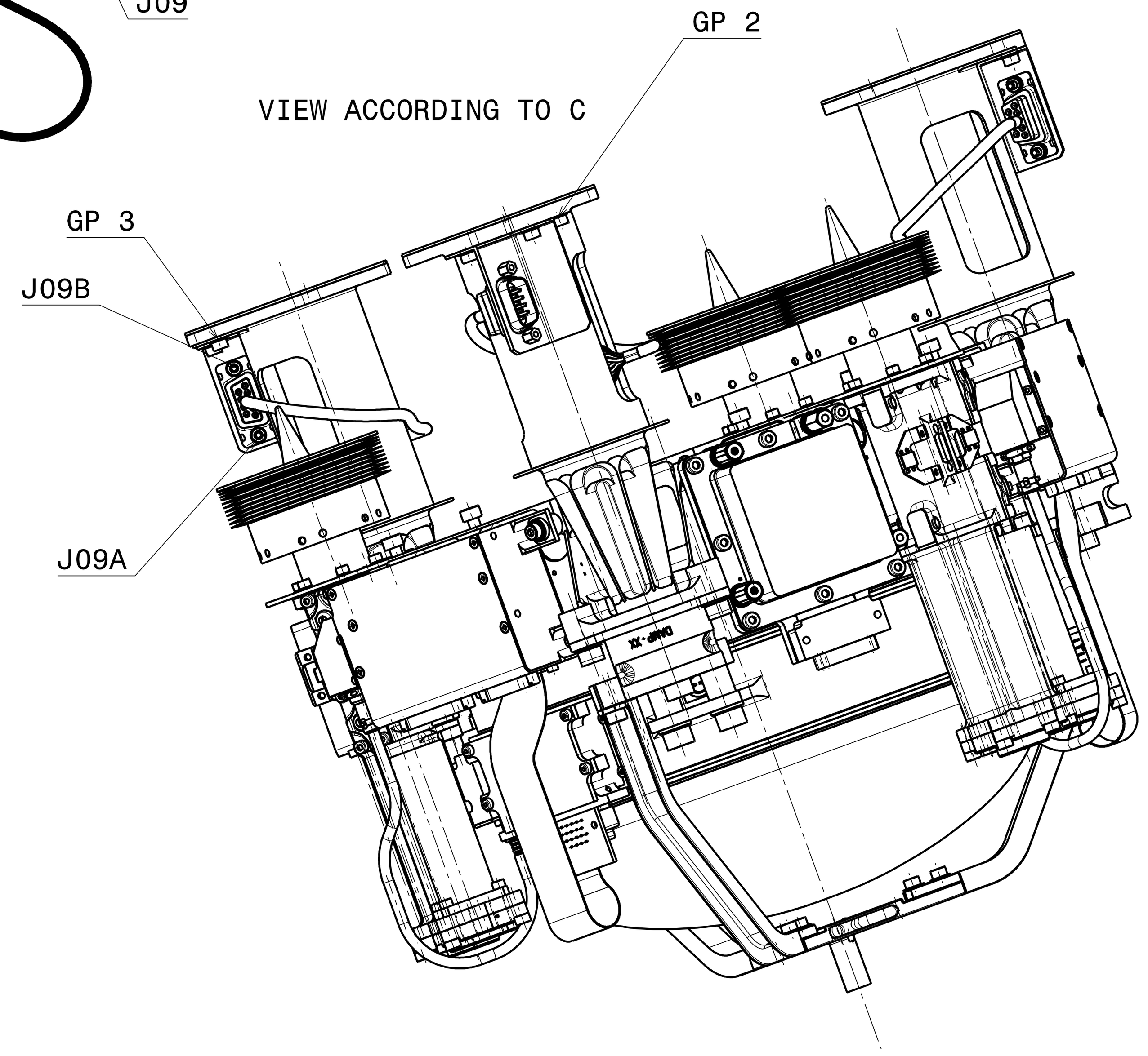

VIEW ACCORDING TO B

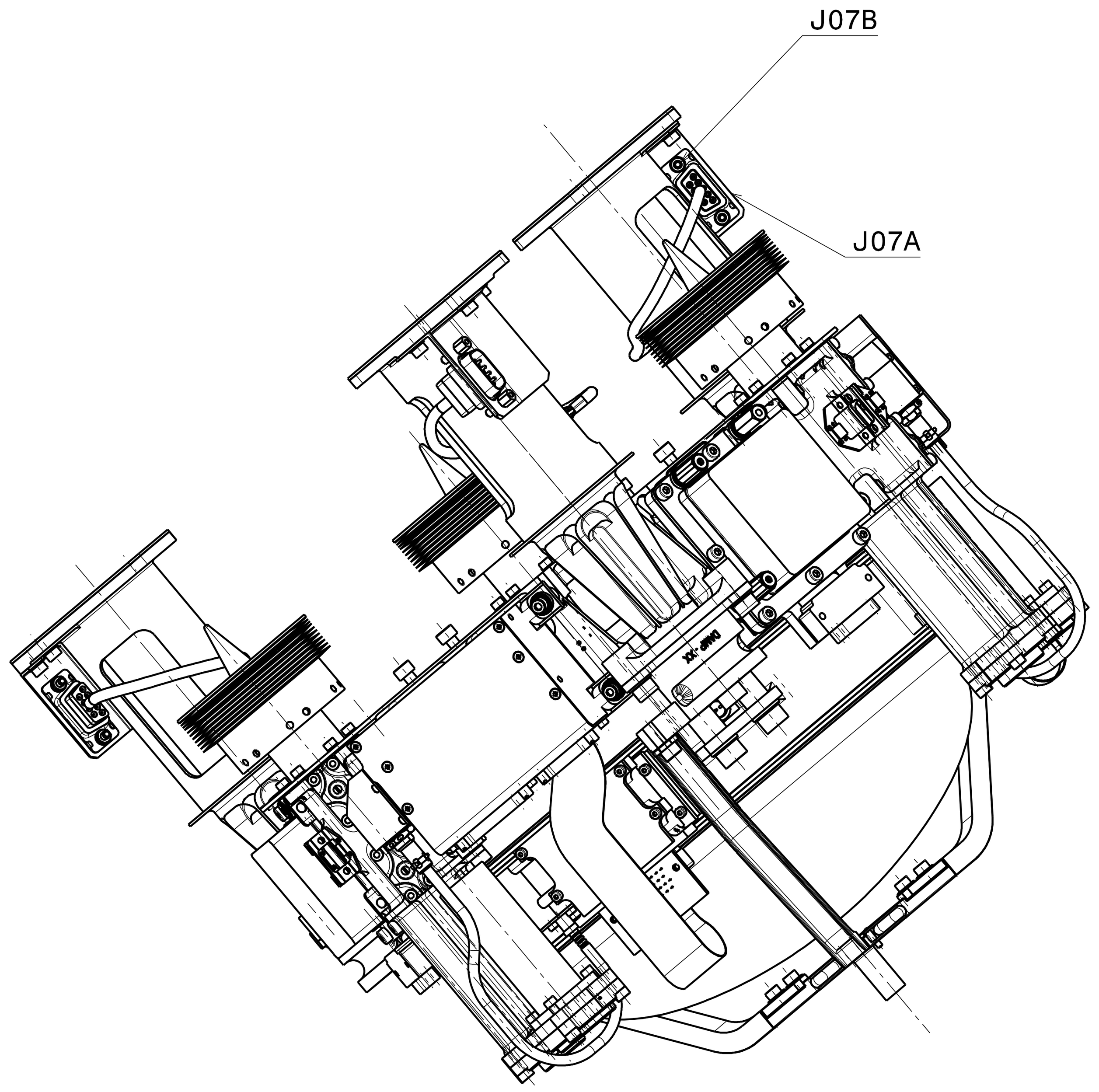

ETAT DE SURFACE :	TOL.GENERALE : ISO 2768-mK	MATIERE : . OU .	TRAITEMENT: .
DESSINE PAR : A.RAFFLEGEAU	LE : 2014/11/24		
VERIFIE PAR : C.IMBERT	LE : 2014/11/24		
VISE PAR : .	LE : .		
SERVICE EMETTEUR : DCT/TV/MT			

CENTRE NATIONAL D'ETUDES SPATIALES
CENTRE SPATIAL DE TOULOUSE
18 AVENUE EDOUARD BELIN, 31401 TOULOUSE CEDEX 9
TEL : 05-61-27-31-31 FAX : 05-61-27-31-79

CE DOCUMENT EST LA PROPRIETE DU CNES ET NE PEUT ETRE COMMUNIQUE OU REPRODUIT SANS SON AUTORISATION

ECHELLE : 1/2 . / .
PROJECTION :

DESIGNATION :
SEIS ASSEMBLY
ACTUATOR DEPLOYMENT CONNECTORS AND GROUNDING POINTS

FORMAT : A1
INDICE : F
PLANCHE : 4/4

NOM DU PLAN EN BD CATIA : .
N° DE PLAN : 2013-MT-INSIGHT-0002
